# Ring-fused cyclobutanes *via* cycloisomerization of alkylidenecyclopropane acylsilanes†

**DOI:** 10.1039/d0sc02224a

**Published:** 2020-05-04

**Authors:** Sarah Eichenberger, Moritz Hönig, Matthieu J. R. Richter, Renana Gershoni-Poranne, Erick M. Carreira

**Affiliations:** Laboratorium für Organische Chemie, Eidgenössische Technische Hochschule Zürich, HCI H335 Vladimir-Prelog-Weg 3 8093 Zürich Switzerland carreira@org.chem.ethz.ch

## Abstract

A novel Lewis acid-catalyzed cycloisomerization of alkylidenecyclopropane acylsilanes is disclosed. The readily available starting materials participate in tandem Prins addition/ring expansion/1,2-silyl shift to grant access to bicyclo[4.2.0]octanes and bicyclo[3.2.0]heptanes, which are common motifs in terpenoid natural products. Notably, the transformation relies on the ability of acylsilanes to act sequentially as acceptors and donors on the same carbon atom.

## Abstract 2

A novel Lewis acid-catalyzed cycloisomerization of alkylidenecyclopropane acylsilanes is disclosed that involves tandem Prins addition/ring expansion/1,2-silyl shift to grant access to bicyclo[4.2.0]octanes and bicyclo[3.2.0]heptanes.

## Graphical abstract

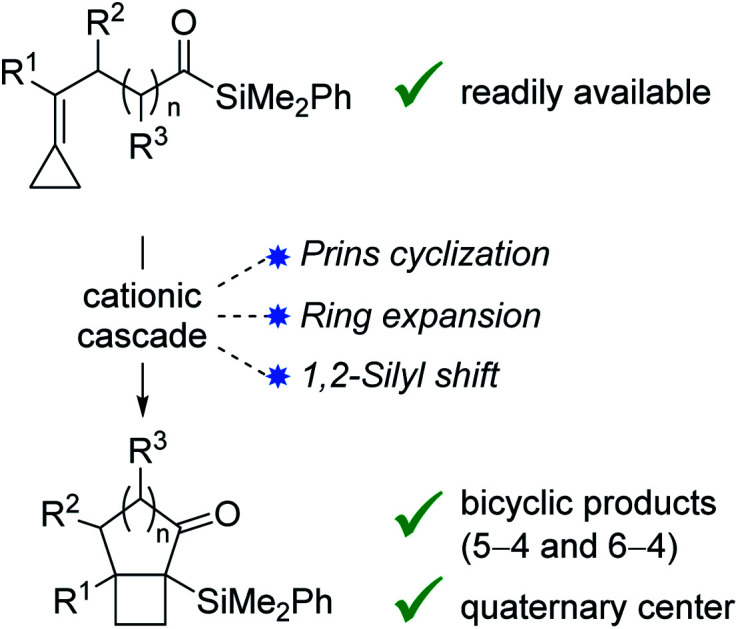

## Introduction

**Fig. 1:**
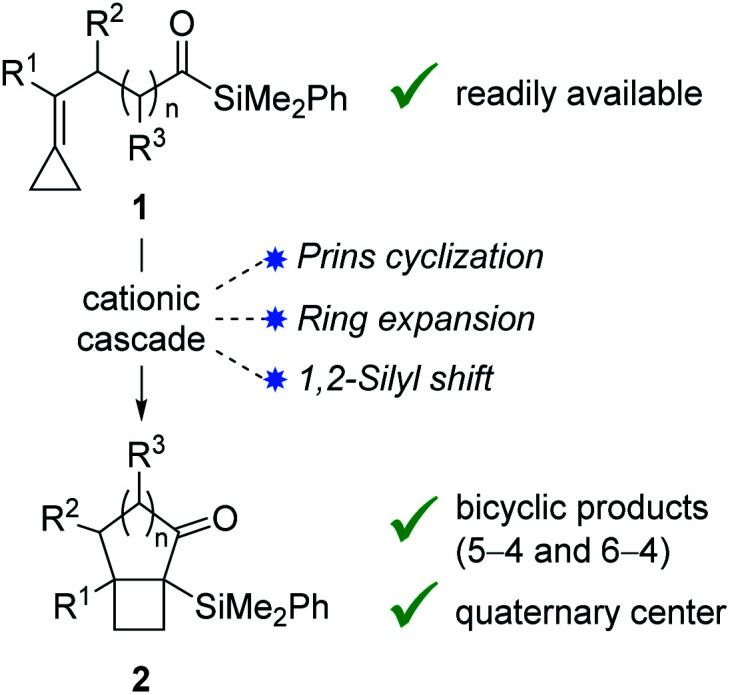
Cycloisomerization cascade.

Bicyclo[4.2.0]octanes and bicyclo[3.2.0]heptanes are common motifs in natural products, such as those belonging to the protoilludane, sterpurane and punctaporonane families.^1^ Additionally, both hydrocarbon and heterocyclic analogues have found interest in the discovery and development of small molecule therapeutics.^2^ Herein, we report a novel Lewis acid-catalysed ring-expanding cycloisomerization of alkylidenecyclopropane acylsilanes **1**. The transformation generates bicyclic products **2** that incorporate fused cyclobutanes with embedded quaternary centers (Fig. 1). Notably, this method offers high diastereocontrol over the newly formed quaternary stereocenters, and the resulting α-silyl ketones offer a key handle for divergent product functionalization.

**Scheme 1:**
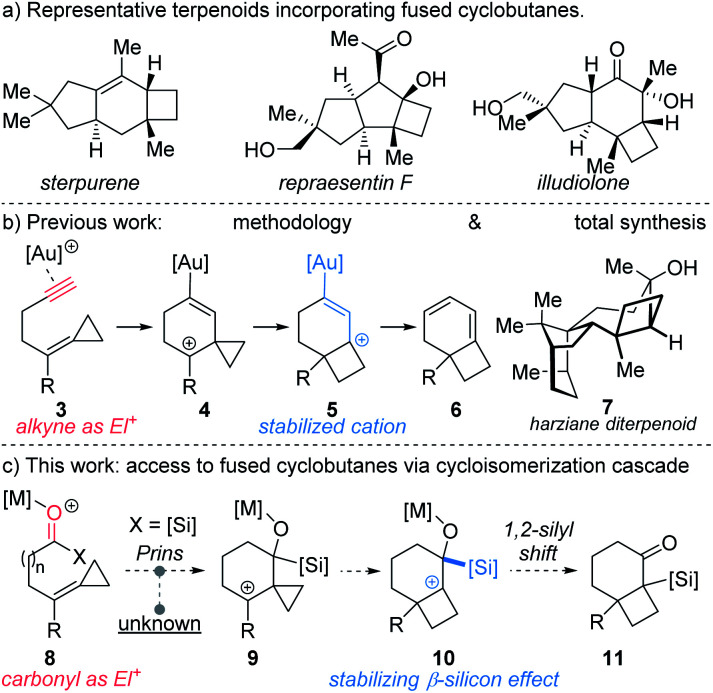
(a) Relevant natural products; (b) prior work; and (c) this work.

Sterpurene, repraesentin F, and illudiolone (Scheme 1a) exemplify natural products that include bicyclo[4.2.0]octane and bicyclo[3.2.0]heptane. The complex scaffolds along with the quaternary centers at the cyclobutane ring junction render these structures challenging targets for synthesis.^3^ Among the most widespread methods to access bicyclo[4.2.0]octane and bicyclo[3.2.0]heptane scaffolds are [2 + 2] photocycloaddition reactions involving cyclic enone acceptors and ethylene.^4^ However, experimental inconvenience associated with the use of ethylene limits the utility of such approaches. Alternative strategies towards these include cobalt-mediated [2 + 2 + 2] cycloaddition,^5^ cyclopropane ring expansion,^6^ and homo-Favorskii rearrangement,^7^ among others. However, cyclobutanes fused to either 5- or 6-membered rings that possess quaternary stereocenters at the ring junction continue to pose considerable challenges for synthesis. In a pioneering study, Fürstner reported the use of alkylidene cyclopropanes for the synthesis of cyclobutenes mediated by platinum-catalysis.^8^ The requisite starting materials were accessed in one step from aldehydes by use of the Julia–Kocienski reaction. Other convenient access routes to alkylidene cyclopropanes from aldehydes include Wittig^9^ and Petasis^10^ olefination approaches. Transformations exploiting alkylidenecyclopropanes or vinyl cyclopropanes as cyclobutane precursors have been developed employing π-acid catalysis.^6*b*–*g*^ For example, acetylenic alkylidene cyclopropanes have been reported to undergo Au-catalyzed rearrangement to furnish cyclobutane-fused 1,3-cyclohexadienes **6** (Scheme 1b).^6*f*^ We recently implemented this method in the first total synthesis and stereochemical revision of harziane diterpenoid **7**, using a key gold-catalyzed cycloisomerization to access the natural product's cyclobutane core.^11^ Despite the power of the transformation involving alkylidene cyclopropanes, expansion to other reactive functionality, such as acylsilanes would considerably expand its utility.

## Results and discussion

Analysis of the enyne isomerization over the course of the sequence of steps reveals that the alkyne and its derivatives display complementary reactivity patterns.^12^ Thus, in the conversion of **3** to **4** the coordinated alkyne is electrophilic; by contrast, the ensuing cation **5** enjoys stabilization by the electron-rich vinyl gold fragment (AuC=C) derived from the alkyne. Accordingly, we became interested in identifying acetylene surrogates that would exhibit similar behavior and trigger analogous reaction cascades. Based on the pioneering work of Kuwajima,^13^ Reich,^14^ and, more recently, Johnson,^15^ we reasoned that acyl acylsilanes could serve as a suitable proxy for the alkyne in cycloisomerization reactions.^16^

Accordingly, we hypothesized that in the presence of a Lewis acid acylsilane **8** might trigger intramolecular Prins-type addition of alkylidenecyclopropane to generate cyclopropyl carbinyl cation **9** (Scheme 1c).^17^ Although addition of substituted vinylidene cyclopropanes to aldehydes have been reported,^18^ additions to acylsilanes are unprecedented. The formation of **9** might subsequently bring about ring expansion to form cyclobutyl cation **10**,^8,19^ which following 1,2-silyl shift would lead to formation of α-silyl ketone **11**.^20^

**Scheme 2:**
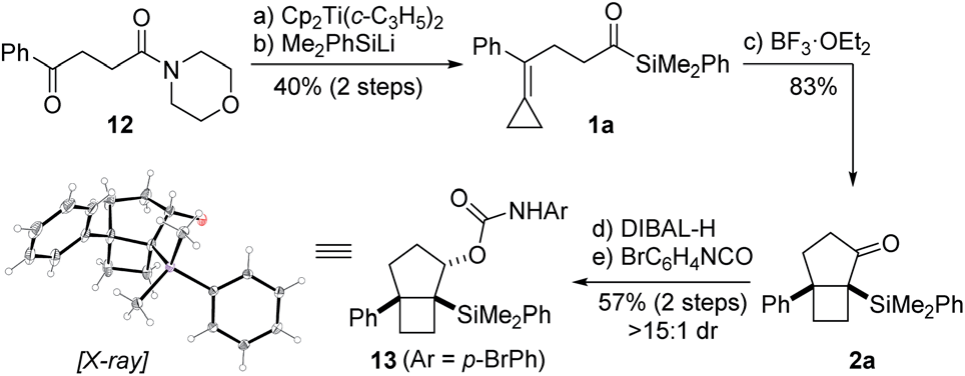
Proof of concept. Reagents and conditions: (a) Cp_2_Ti(*c*-C_3_H_5_)_2_, PhMe, 50 °C; (b) Me_2_PhSiLi, THF, −78 °C, 40% (2 steps); (c) BF_3_·OEt_2_ (20 mol%), CH_2_Cl_2_, −78 to −20 °C, 83%; (d) DIBAL-H, CH_2_Cl_2_, −78 °C; (e) 4-bromophenyl isocyanate, Et_3_N, CH_2_Cl_2_, 37 °C, 57% (2 steps), >15 : 1 dr. The thermal ellipsoids in the X-ray structure of **13** are displayed at 50% probability level. Moreover, the carboxamide on the secondary alcohol was omitted for clarity.

Our synthetic studies commenced with investigation of the cycloisomerization of acylsilane **1a** containing a phenyl-substituted alkylidenecyclopropane (Scheme 2). This substrate was accessed from ketoamide **12** in two steps involving chemoselective olefination using Petasis' dicyclopropyl titanocene reagent,^10^ followed by addition of dimethylphenylsilyllithium.^21^ Treatment of acylsilane **1a** with 20 mol% BF_3_·OEt_2_ in dichloromethane at −78 to −20 °C effected cycloisomerization and yielded bicyclo[3.2.0]heptane **2a** in 83% yield and as a single diastereoisomer.^22^ We also examined other Lewis acids on related substrates, such as TMSOTf, EtAlCl_2_, In(OTf)_3_, but none gave the desired product. The relative configuration of ring fusion was initially, tentatively assigned to be *cis* based on the severe energetic penalty that would otherwise be associated with formation of the alternative *trans* fusion.

This assignment was subsequently corroborated by X-ray crystallographic analysis of carbamate derivative **13**, which was accessed from α-silyl ketone **2a** by diastereoselective carbonyl reduction (DIBAL-H, >15 : 1 dr), followed by treatment of the resulting secondary alcohol with *para*-bromophenyl isocyanate. As a control experiment, it is noteworthy that when the corresponding aldehyde was used *in lieu* of the acyl silane, we did not observe product formation. Having demonstrated the viability of the reaction, we next investigated the scope of the novel cycloisomerization reaction (Table 1). The cycloisomerization proved suitable for the synthesis of fused 5–4 and 6–4 ring systems, as demonstrated by the formation of bicyclo[3.2.0]heptane **2a** (83%) and bicyclo[4.2.0]octane **2b** (84%). The transformation could furthermore be extended to substrates containing alkyl substituents (**2c–2e**). Among these, cyclopropyl-substituted cyclobutane **2e** is an interesting case because it results from regioselective ring expansion of the spirocyclic cyclopropane ring of the putative intermediate cyclopropylcarbinyl cation **14**. The observed preference for migration of a spirocyclic cyclopropane C–C bond may result from the greater stability of the ensuing tertiary cyclobutyl cation (**15**) over the alternative secondary cyclobutyl cation in spirocycle **16**.

**Table 1:** Substrate scope^a^

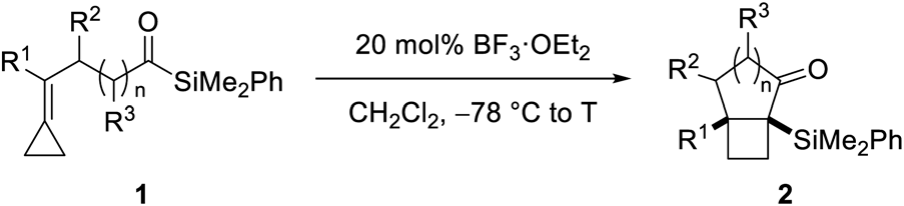
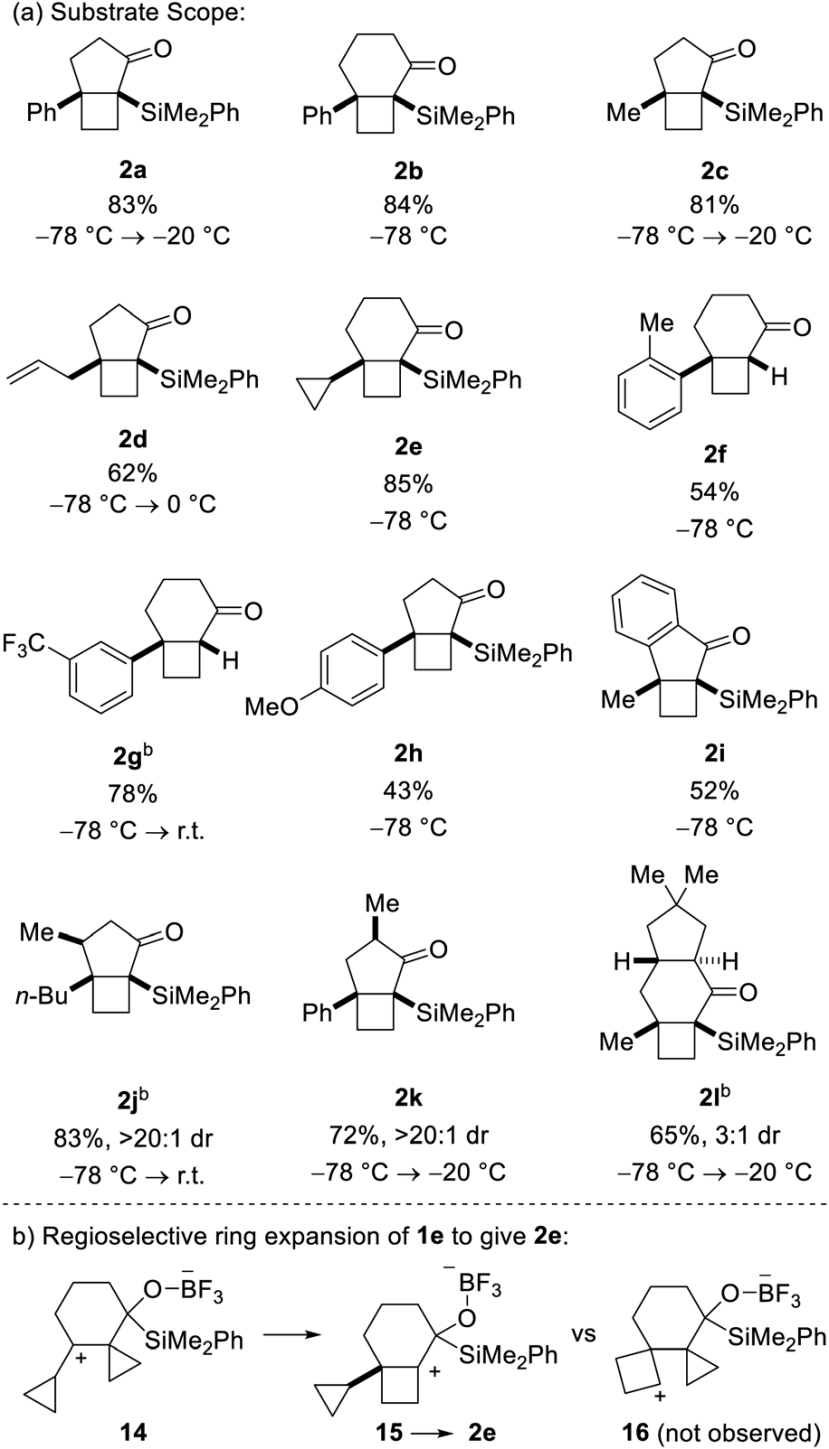

a Yields refer to isolated product after purification.

b 40 mol% BF_3_·OEt_2_ used.

The substrate scope of the novel cycloisomerization reaction was found to extend to substrates incorporating aryl substituents bearing electron-donating and electron-withdrawing groups (**2f–2h**). Interestingly, cycloisomerization of arenes with *ortho*- or *meta*-substitution was accompanied by product lacking the silyl group (*cf.***2f** and **2g**). For these two cases, we hypothesize that the additional steric encumbrance exerted by the substituted arenes results in competing desilylation of intermediate **10** (Scheme 1) thereby producing directly an enolate, which is protonated upon work-up. Intriguingly, incorporation of various substitution patterns along the alkyl chain linking the alkylidene cyclopropanes and acyl silane led to formation of a range of substituted polycyclic products **2i–2l** in moderate to high yield. It is notable that the cyclization takes place in a highly diastereoselective manner. Accordingly, incorporation of a methyl group at the allylic position or at C_α_ in the acylsilane led to highly diastereoselective formation of the all-*cis* isomers of bicyclo[3.2.0]heptanes **2j** and **2k** in >20 : 1 dr. Furthermore, incorporation of a dimethyl-substituted cyclopentane ring in cyclization precursor **1l** led to generation of the corresponding tricyclic product **2l** in 65% yield and 3 : 1 dr. Notably, **2l** incorporates the characteristic carbon skeleton of sterpurane derived natural products, which underscores the significance of the novel cycloisomerization reaction to access complex terpenoids.

**Scheme 3:**
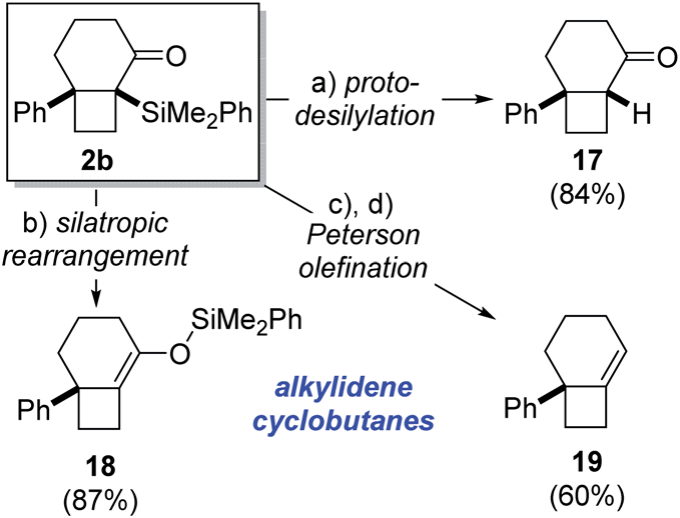
Product functionalization. Reagents and conditions: (a) KF, MeOH, r.t., 84%; (b) PhMe, 90 °C, 87%; (c) DIBAL-H, CH_2_Cl_2_, −78 °C; (d) BF_3_·OEt_2_, CH_2_Cl_2_, 0 °C, 60% (2 steps).

The α-silyl ketone functionality present in the cycloisomerization products provide a suitable handle for further functionalization, as demonstrated for bicyclo[4.2.0]octane **2b** as a representative example (Scheme 3). For instance, protodesilylation of **2b** was effected in high yield upon exposure to potassium fluoride in methanol at room temperature to give bicyclo[4.2.0]octane **17**. Alternatively, heating of **2b** in toluene at 90 °C promoted rearrangement to the isomeric silyl enol ether **18** in high yield.^23^ This silyl enol ether, featuring a strained alkylidenecyclobutane double bond, would be difficult to generate by regioselective enol ether formation of the parent ketone. Marek has recently demonstrated the ability of related tetrasubstituted phenyldimethylsilyl enol ethers to engage in aldol addition reactions for the formation of quaternary stereocenters.^24^ Consequently, this corroborates the synthetic utility associated with phenyldimethylsilyl enol ethers such as **18** and, by extension, their corresponding α-silyl ketones **2**. The exclusive formation of α-silyl ketone products (**2**) from Lewis acid-induced cycloisomerization of acylsilanes **1** likely reflects kinetic preference for 1,2-silyl shift of the intermediate cyclobutyl cation **10** over competing Brook rearrangement to furnish silyl enol ether isomer.

The synthetic utility of the α-silyl ketone motif in the cycloisomerization product **2c** is further demonstrated by a reduction/Peterson olefination^25^ sequence (Scheme 3). Accordingly, treatment of **2b** with DIBAL-H led to formation of a diastereomeric mixture of secondary alcohols (dr 3 : 1), which underwent highly regioselective elimination to generate alkylidenecyclobutane **19** in 60% yield over 2 steps. It is notable that both diastereomeric alcohols proved competent substrates for elimination^26^ and did not lead to formation of regioisomeric olefin products. This demonstrates the strategic advantage associated with the novel cycloisomerization reaction in providing a handle for installation of strained alkylidenecyclobutane double bonds. Additionally, the ability to access cyclobutanes with exocyclic double bonds renders this method complementary to Fürstner's platinum-catalyzed isomerization of alkylidenecyclopropanes, which generates cyclobutenes containing endocyclic double bonds.^7^

**Scheme 4:**
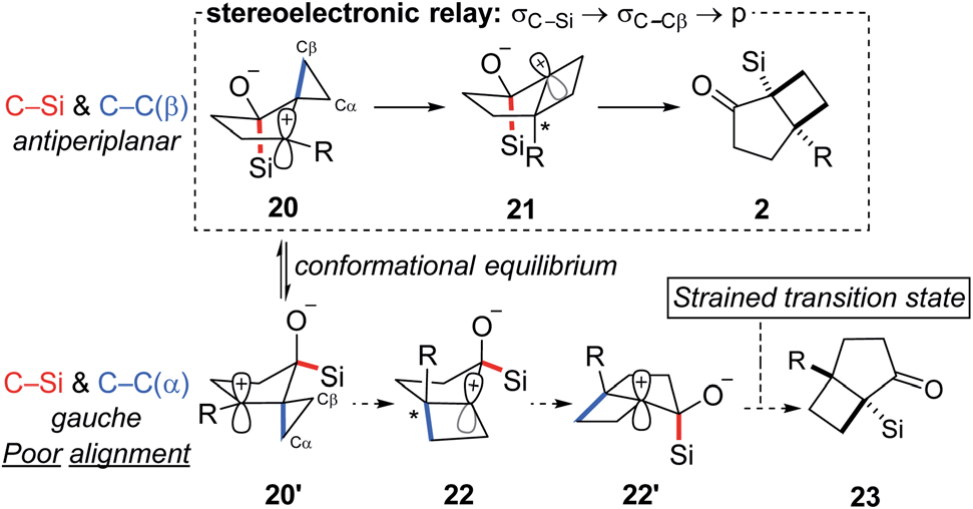
Stereochemical course of the cyclization reaction.

Reflection on the transformation described prompts interesting questions concerning the mechanism. Rearrangement pathways for two limiting conformations that highlight the relationship between the C–Si and the cyclopropyl C–C are outlined in Scheme 4: C–Si and C–C_β_*anti*-periplanar (**20**) along with C–Si and C–C_β_ roughly *syn*-periplanar (**20′**). The former is expected to furnish the observed products (**2**) while products resulting from the latter are not observed because of poor alignment of C–Si relative to the migrating C_α_ cyclopropane carbon. To further probe the origin of the selectivity for the *syn* isomer, we performed a computational analysis.^27,28^

Although **20′** was calculated to be favored thermodynamically by ∼1 kcal mol^−1^, **20** undergoes a facile migration with no meaningful energy barrier, leading to **21**. Conversely, **20′** is unable to undergo an analogous migration, and attempts to obtain structure **22** as a local minimum failed, indicating that not only is the rearrangement disfavored, but the intermediate itself is not a stable compound. Analysis of the geometries of the conformers reveal the underlying reasons for this difference.

**Scheme 5:**
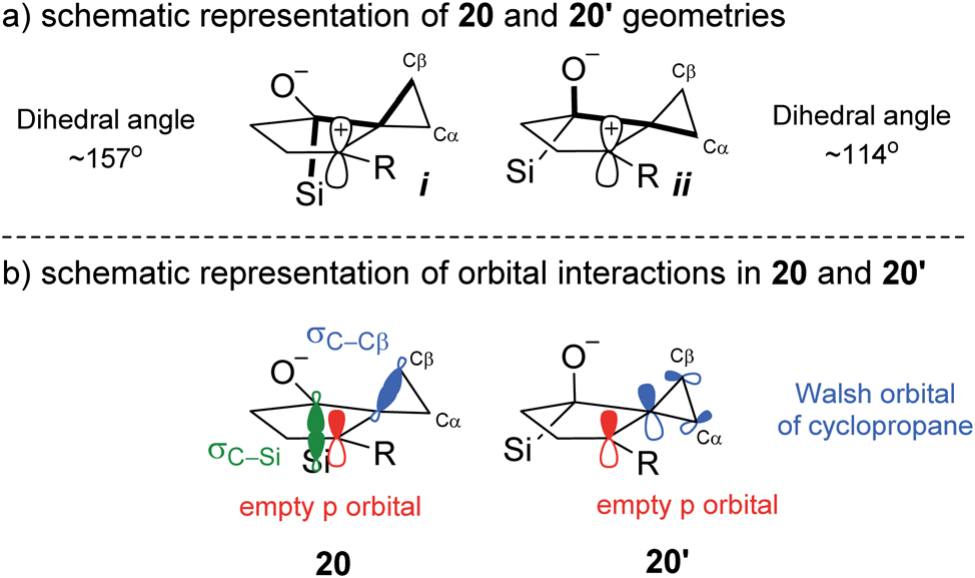
Geometrical and orbital analysis of the stereoelectronic relay based on computational results.

The calculated optimized geometry of **20′** shows the cyclopropyl bisected by the plane of the cyclopentane (**ii** in Scheme 5a). This allows for a stabilizing interaction between the Walsh-like cyclopropane orbital and the neighbouring carbocation. In contrast, the cyclopropyl ring in **20** is forced “upwards” from the plane of the cyclopentane ring, in the direction opposite the pseudo-axial silicon (Scheme 5a structure **i**). This distortion is likely triggered by the destabilizing out-of-phase interaction between the cyclopropyl Walsh-like orbital and the σ_C–Si_ orbital. This results in the orbital becoming more localized and positioned almost parallel to the empty p orbital (Scheme 5b**20**), allowing for facile migration of C_β_ to the neighboring carbon. Importantly, because the cyclopropyl group is distorted as shown in **20**, it is always C_β_ that migrates, leading to the selectivity for the *syn* positioning of the R and Si groups. An analogous interaction with the σ_C–O_ orbital was not identified in **20′**. Following the migration of C_β_ in **20**, the positive charge is located β to the Si group, leading to a rapid 1,2-silyl shift and resulting in the formation of the observed *cis*-fused product **2**, which is found to be 29.5 kcal mol^−1^ lower in energy than **21**. Additional calculations and visualizations of the molecular orbitals supporting this analysis are provided in the ESI.†

## Conclusions

We developed a highly diastereoselective ring-expanding cycloisomerization reaction of alkylidenecyclopropane acylsilanes to bicyclic α-silyl ketones. The products feature cyclobutane rings containing a quaternary stereocenter, which are challenging motifs for synthesis. The α-silyl ketone functionality present in the cyclization products provides a suitable handle for further functionalization of the cyclobutane ring. Notably, all cyclization precursors were synthesized in two steps from simple acyclic ketoamide precursors. More broadly, the cationic annulation of vinylidine cyclopropanes have now been expanded to include acylsilanes as reaction partners. Applications of this method in the context of total syntheses are currently ongoing and will be reported in due course.

## Conflicts of interest

The authors declare no conflict of interest.

## Supplementary Material

SC-011-D0SC02224A-s001

SC-011-D0SC02224A-s002
