# Supplementary material for: Ring-fused cyclobutanes via cycloisomerization of alkylidenecyclopropane acylsilanes
Source: Chem Sci. 2020 May 4;11(20):5294–8. doi: 10.1039/d0sc02224a (PMC8159344; doi:10.1039/d0sc02224a)
Supplement: SC-011-D0SC02224A-s001 [file SC-011-D0SC02224A-s001.pdf]

Electronic Supplementary Material (ESI) for Chemical Science.
This journal is © The Royal Society of Chemistry 2020

SUPPORTING INFORMATION

Table of Contents

1. General Methods 2
2. Synthesis of Cycloisomerization Substrates and Products 3
3. Lewis Acid Screening for Cycloisomerization of **397l** 30
4. Product Functionalization 31
5. Synthesis of Crystalline Derivative **415** 33
6. Control Experiments 35
7. NMR Spectra 38
8. X-Ray Crystallographic Data 103

1. General Methods

Chemicals and Solvents: All chemicals were purchased from ABCR, ACROS, ALDRICH, ALFA AESAR, FLUOROCHEM, MERCK, TCI, OR STREM and used as such unless stated otherwise. Deuterated solvents were obtained from ARMAR chemicals and CAMBRIDGE ISOTOPIC LABORATORIES.

Reaction handling: All reactions involving non-aqueous solvents were carried out in vacuum dried glassware sealed with rubber septa and under a positive pressure of argon unless otherwise stated. Reactions were stirred using magnetic stirring bars and monitored by TLC (using Merck silica gel F254 TLC glass plates) and visualized with UV fluorescence quenching or Seebach's stain, unless otherwise stated. Concentrations under reduced pressure were performed by rotary evaporation at 30 °C at the appropriate pressure. Chromatographic purification was performed as flash chromatography (using Silicycle SiliaFlash® Silica Gel P60). The yields given refer to chromatographically purified and spectroscopically pure compounds, unless otherwise stated.

NMR spectroscopy: Nuclear Magnetic Resonance spectra were recorded on BRUKER ASCEND, BRUKER AV, and BRUKER DRX (400/500 MHz for ^{1}H NMR and 126/101 MHz for ^{13}C NMR) spectrometers. Measurements were carried out at room temperature. Chemical shifts (δ) are reported in ppm with the residual solvent signal as internal standard (chloroform at 7.26 and 77.16 ppm, benzene at 7.16 and 128.06 ppm, dichloromethane at 5.32 and 54.00 ppm, respectively). The data is reported as (s=singlet, d=doublet, t=triplet, q=quadruplet, m=multiplet or unresolved, br=broad signal, coupling constant(s) in Hz, integration). ^{13}C NMR spectra were recorded with complete ^{1}H decoupling.

IR spectroscopy: Infrared spectra were recorded on a PERKIN ELMER TWO-FT-IR spectrometer as thin films. Absorptions are given in wavenumbers (cm^{-1}).

Mass spectrometry: Mass spectrometric analyses were performed as high resolution ESI, EI, and MALDI measurements by the mass spectrometry service of the Laboratorium für Organische Chemie at ETH Zürich by Mr. LOUIS BERTSCHI, Mr. OSWALD GRETER, Mr. DANIEL WIRTZ, and Mr. MICHAEL MEIER under supervision of Dr. BERTRAN GERRITS.

X-Ray diffraction: X-Ray diffraction analysis was performed on a BRUKER Kappa Apex II DUO system equipped with a graphite monochromator by Dr. NILS TRAPP and Mr. MICHAEL SOLAR at the Laboratorium für Organische Chemie at ETH Zürich. The data obtained was deposited at the Cambridge Crystallographic Data Centre.

2. Synthesis of Cycloisomerization Substrates and Products

a) Substrate Synthesis:

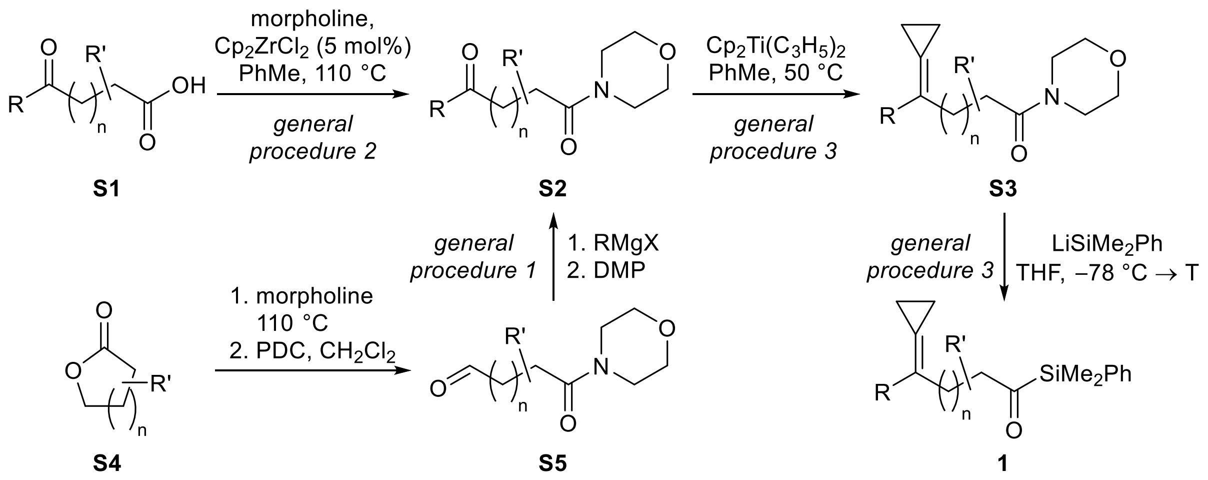

b) Lewis acid-catalyzed Cycloisomerization:

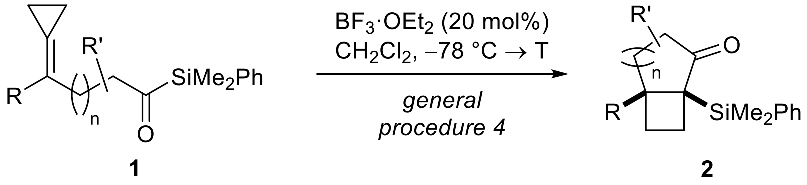

General procedure #1: Synthesis of keto amides **S2** from aldehydes **S5**.

To a solution of the appropriate aldehyde **S5** (1 equiv) in THF (0.1 M) at –78 °C was added dropwise a solution of the appropriate Grignard reagent. The reaction mixture was stirred at the indicated temperature for the indicated time before it was quenched by dropwise addition of sat. aq. $NaHCO_3$ and allowed to warm to room temperature. The aqueous layer was extracted four times with EtOAc and the combined organic layers were dried ($MgSO_4$), filtered, and concentrated *in vacuo*. The crude secondary alcohols thus obtained were generally used in the subsequent reaction without further purification.

To a solution of the appropriate secondary alcohol in CH_2Cl_2 (0.2 M) were added $NaHCO_3$ (2 equiv) and DMP (1.2 equiv). The reaction mixture was stirred at room temperature for 2 hours before it was quenched by addition of sat. aq. $NaHCO_3$ and sat. aq. $Na_2S_2O_3$. The organic layer was separated and the aqueous layer was extracted four times with EtOAc. The combined organic layers were dried ($MgSO_4$), filtered, and concentrated *in vacuo*. Column chromatography yielded ketones **S2**.

General procedure #2: Synthesis of keto amides **S2** from acids **S1**.

Following a literature procedure,[1] a mixture of the appropriate acid **S1** (1 equiv), morpholine (1 equiv), and Cp_2ZrCl_2 (5 mol%) in toluene (0.1 M) were heated at 110 °C for 12 hours. The reaction mixture was allowed to cool to room temperature and concentrated *in vacuo*. Column chromatography yielded morpholine amides **S2**.

General procedure #3: Synthesis of acylsilanes **1** from ketoamides **S2**.

To a solution of ketone **S2** in PhMe (0.1 M) were added $NaHCO_3$ (4.0 equiv) and a solution of dicyclopropane titanocene (prepared according to ref. 2) in toluene (0.55 M, 1.3 equiv). The reaction mixture was heated at 50 °C for 3 hours before it was allowed to cool back to room temperature and filtered through silica gel (eluent: grade from 1:20:180 to 1:200:0 NEt_3–EtOAc–hexane). Concentration yielded the intermediate crude alkylidenecyclopropane **S3**, which was used without further purification in the next step.

Following a literature procedure,[3] to a solution of the crude alkylidenecyclopropane thus obtained in THF (0.1 M) at –78 °C was added a solution of dimethylphenylsilyllithium (prepared according to reference [4]) in THF (0.75 M, 1.5 equiv). The reaction mixture was stirred at this temperature for 2–3 hours before it was quenched by dropwise addition of sat. aq. $NaHCO_3$ and subsequently allowed to warm to room temperature. The aqueous layer was extracted four times with ethyl acetate and the combined organic layers were dried ($MgSO_4$), filtered, and concentrated *in vacuo*. Column chromatography yielded acylsilane **1**.

General procedure #4: Ring-expanding cycloisomerization of acylsilanes **1** to cyclobutanes **2**.

To a solution of acylsilane **1** in CH_2Cl_2 (0.09 M) at –78 °C was added $BF_3{\cdot}OEt_2$ in CH_2Cl_2 (20 mol%, 0.28 M) from a freshly prepared stock solution (the stock solution was prepared by dissolving $BF_3{\cdot}OEt_2$ (16 µL, 0.13 mmol) in CH_2Cl_2 (0.46 mL). The reaction mixture was stirred at the indicated temperature for the indicated time before it was quenched by addition of sat. aq. $NaHCO_3$ and allowed to warm to room temperature. The aqueous layer was extracted four times with CH_2Cl_2 and the combined organic layers were dried ($MgSO_4$), filtered, and concentrated *in vacuo*. Column chromatography yielded cyclobutanes **2**.

[1] Allen, C. L.; Chhatwal, A. R.; Williams, J. M. J., *Chem. Commun.* **2012**, *48*, 666.
[2] Petasis, N. A.; Bzowej, E. I. *Tetrahedron Lett.* **1993**, *34*, 943.
[3] Clark, C. T.; Milgram, B. C.; Scheidt, K. A., *Org. Lett.* **2004**, *6*, 3977.
[4] Fleming, I.; Roberts, R. S.; Smith, S. C., *J. Chem. Soc. Perkin Trans. 1* **1998**, 1209.

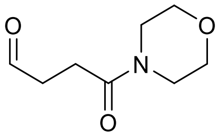

Aldehyde **S5-1**.

Following a literature procedure,[5] a mixture of γ-butyrolactone (2.85 mL, 34.8 mmol) and morpholine (3.04 mL, 34.8 mmol, 1.0 equiv) was heated at 110 °C for 4 hours before it was allowed to cool back to room temperature. The crude hydroxy amide thus obtained was used in the next step without further purification.

To a solution of the crude hydroxy amide thus obtained in CH_2Cl_2 (231 mL) was added PCC (14.9 g, 69.3 mmol, 2 equiv). The reaction mixture was stirred at room temperature for 2 hours before celite (15 g) was added and the reaction mixture was stirred vigorously for another 30 minutes. The reaction mixture was subsequently diluted with diethyl ether and filtered, washing copiously with diethyl ether. The filtrate was concentrated *in vacuo*. Column chromatography (eluent: grade from 10% to 100% EtOAc–hexane) yielded aldehyde **S5-1** (3.2 g, 18.7 mmol, 54%) as a colorless oil.

TLC: R_f = 0.55 (10% MeOH/CH_2Cl_2, $KMnO_4$ stain); ^{1}H-NMR (400 MHz, $CDCl_3$): δ 9.86 (br s, 1H), 3.72 – 3.64 (m, 4H), 3.63 – 3.57 (m, 2H), 3.52 – 3.47 (m, 2H), 2.84 (t, *J* = 6.4 Hz, 2H), 2.63 (t, *J* = 6.4 Hz, 2H) ppm; ^{13}C-NMR (101 MHz, $CDCl_3$): δ 201.1, 169.8, 67.0, 66.6, 45.9, 42.3, 38.7, 25.6 ppm; IR: ν 1717, 1638, 1439, 1114, 1033 cm^{-1}; HRMS (ESI): Exact mass calculated for $C_8H_{13}NNaO_3^+$ [$(M+Na)^+$] 194.0788, found 194.0785.

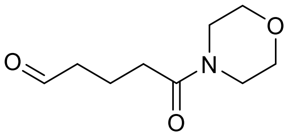

Aldehyde **S5-2**.

Following a literature procedure,[6] a mixture of δ-valerolactone (2.97 mL, 32.0 mmol) and morpholine (3.07 mL, 35.2 mmol, 1.0 equiv) was heated at 110 °C for 4 hours before it was allowed to cool back to room temperature. The crude hydroxy amide thus obtained was used in the next step without further purification.

To a solution of the crude hydroxy amide thus obtained in CH_2Cl_2 (214 mL) was added PCC (10.4 g, 48.1 mmol, 1.5 equiv). The reaction mixture was stirred at room temperature for 2 hours before celite (15 g) was added and the reaction mixture was stirred vigorously for another 30 minutes. The reaction mixture was subsequently diluted with diethyl ether and filtered, washing copiously with diethyl ether. The filtrate was concentrated *in vacuo*. Column chromatography (eluent: grade from 10% to 100% EtOAc–hexane) yielded aldehyde **S5-2** (2.97 g, 16.0 mmol, 50%) as a pale green oil.

TLC: R_f = 0.55 (10% MeOH/CH_2Cl_2, $KMnO_4$ stain); ^{1}H-NMR (400 MHz, $CDCl_3$): δ 9.78 (t, *J* = 1.2 Hz, 1H), 3.69 – 3.63 (m, 3H), 3.64 – 3.57 (m, 2H), 3.49 – 3.44 (m, 2H), 2.58 (td, *J* = 6.8, 1.3 Hz, 1H), 2.36 (t, *J* = 7.2 Hz, 1H), 1.97 (p, *J* = 7.0 Hz, 1H) ppm; ^{13}C-NMR (101 MHz, $CDCl_3$): δ 202.2, 170.9, 67.0, 66.8, 46.0,

[5] Meth-Cohn, O.; Taylor, D. L., *Tetrahedron* **1995**, *51*, 12869.
[6] Labarre-Lainé, J.; Beniazza, R.; Desvergnes, V.; Landais, Y., *Org. Lett.* **2013**, *15*, 4706.

43.3, 42.1, 32.0, 17.6 ppm; IR: ν 1719, 1640, 1436, 1237, 1115, 1034 cm^{-1}; HRMS (ESI): Exact mass calculated for $C_9H_{15}NNaO_3^+$ [(M+Na)$^+$] 208.0944, found 208.0945.

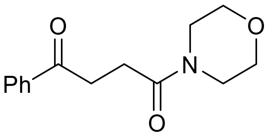

Morpholine amide **12**.

The title compound was prepared according to general procedure #2 from commercially available 4-oxo-4-phenylbutanoic acid (10.0 g, 56.1 mmol). The crude product was purified by flash column chromatography (eluent: grade from 20% to 100% EtOAc–hexane). The title compound was isolated as a pale yellow oil (106. g, 42.9 mmol, 76%). The spectral data was in accordance with the literature.[1]

TLC: R_f = 0.19 (70% EtOAc/hexane, $KMnO_4$ stain); ^{1}H-NMR (400 MHz, $CDCl_3$): δ 8.04 – 7.99 (m, 2H), 7.58 – 7.53 (m, 1H), 7.49 – 7.43 (m, 2H), 3.75 – 3.65 (m, 4H), 3.65 – 3.55 (m, 4H), 3.37 (t, J = 6.5 Hz, 2H), 2.78 (t, J = 6.5 Hz, 2H) ppm.

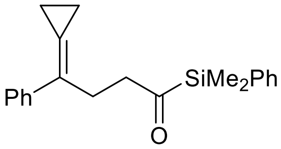

Acylsilane **1a**.

The title compound was prepared according to general procedure #3 from morpholine amide **12** (1.10 g, 4.45 mmol). The crude product was purified by flash column chromatography (eluent: grade from 1:4:196 to 1:60:140 NEt_3–EtOAc–hexane). The title compound was isolated as a pale yellow oil (578 mg, 1.80 mmol, 40% over 2 steps).

TLC: R_f = 0.78 (30% EtOAc/hexane, Seebach stain); ^{1}H-NMR (500 MHz, C_6D_6): δ 7.53 – 7.49 (m, 2H), 7.42 – 7.39 (m, 2H), 7.22 – 7.18 (m, 2H), 7.14 – 7.06 (m, 4H), 3.01 – 2.96 (m, 2H), 2.82 – 2.77 (m, 2H), 1.06 – 1.01 (m, 2H), 0.84 – 0.79 (m, 2H), 0.29 (s, 6H) ppm; ^{13}C-NMR (126 MHz, C_6D_6): δ 243.2, 140.2, 135.0, 134.3, 130.0, 128.6, 128.4, 126.9, 126.8, 126.4, 121.0, 47.5, 26.9, 4.7, 1.4, -4.8 ppm; IR: ν 2958, 1644, 1428, 1249, 1111, 834, 697 cm^{-1}; HRMS (ESI): Exact mass calculated for $C_{21}H_{24}NaOSi^+$ [(M+Na)$^+$] 343.1489, found 343.1489.

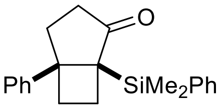

Cyclobutane **2a**.

The title compound was prepared according to general procedure #4 from acylsilane **1a** (100 mg, 0.31 mmol). The reaction mixture was stirred at –78 °C to –20 °C for 3 hours. The crude product was purified by flash column chromatography (eluent: grade from 1:2:198 to 1:20:180 NEt_3/hexane/EtOAc). The title compound was isolated as a colorless oil (83 mg, 0.26 mmol, 83%).

TLC: R_f = 0.35 (10% EtOAc/hexane, Seebach stain); ^{1}H-NMR (400 MHz, C_6D_6): δ 7.41 – 7.36 (m, 2H), 7.20 – 7.10 (m, 3H), 7.06 – 7.00 (m, 3H), 6.86 – 6.81 (m, 2H), 2.44 – 2.37 (m, 2H), 2.37 – 2.29 (m, 1H), 2.17 (ddd, *J* = 11.9, 9.5, 4.4 Hz, 1H), 2.00 (ddd, *J* = 11.9, 8.7, 7.9 Hz, 1H), 1.85 – 1.77 (m, 2H), 1.70 (ddd, *J* = 11.3, 8.7, 4.4 Hz, 1H), 0.32 (s, 3H), 0.10 (s, 3H) ppm; ^{13}C-NMR (101 MHz, C_6D_6) δ 221.4, 147.6, 137.3, 135.3, 129.3, 128.4, 127.7, 126.3, 126.1, 53.2, 52.7, 40.7, 38.0, 30.6, 25.3, -3.5, -4.6 ppm; IR: ν 2957, 1710, 1248, 811, 700, 423 cm^{-1}; HRMS (ESI): Exact mass calculated for $C_{21}H_{24}NaOSi^+$ [$(M+Na)^+$] 343.1489, found 343.1487.

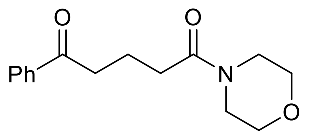

Morpholine amide **S2b**.

The title compound was prepared according to general procedure #2 from commercially available 5-phenyl-5-oxopentanoic acid (8.82 mL, 86.0 mmol). The crude product was purified by flash column chromatography (eluent: grade from 10% to 100% EtOAc–hexane). The title compound was isolated as a colorless oil (2.4 g, 9.2 mmol, 71%).

TLC: R_f = 0.17 (70% EtOAc/hexane, $KMnO_4$ stain); ^{1}H-NMR (400 MHz, $CDCl_3$): δ 7.99 – 7.95 (m, 2H), 7.59 – 7.54 (m, 1H), 7.49 – 7.43 (m, 2H), 3.70 – 3.64 (m, 4H), 3.62 (br s, 2H), 3.52 (br s, 2H), 3.11 (t, *J* = 6.7 Hz, 2H), 2.45 (t, *J* = 7.3 Hz, 2H), 2.09 (p, *J* = 6.8 Hz, 2H) ppm; ^{13}C-NMR (101 MHz, $CDCl_3$): δ 200.1, 171.5, 136.9, 133.3, 128.8, 128.2, 67.0, 66.9, 46.1, 42.1, 37.7, 32.3, 19.7 ppm; IR: ν 1682, 1643, 1448, 1229, 1115, 692 cm^{-1}; HRMS (ESI): Exact mass calculated for $C_{15}H_{20}NO_3^+$ [$(M+H)^+$] 262.1438, found 262.1432.

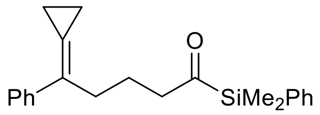

Acylsilane **1b**.

The title compound was prepared according to general procedure #3 from morpholine amide **S2b** (100 mg, 0.383 mmol). The crude product was purified by flash column chromatography (eluent: grade from 1:4:196 to 1:20:180 NEt_3–EtOAc–hexane). The title compound was isolated as a yellow oil (58 mg, 0.17 mmol, 44% over 2 steps).

TLC: R_f = 0.70 (20% EtOAc/hexane, Seebach stain); ^{1}H-NMR (500 MHz, C_6D_6): δ 7.62 – 7.59 (m, 2H), 7.43 – 7.40 (m, 2H), 7.27 – 7.22 (m, 2H), 7.15 – 7.09 (m, 4H), 2.56 (ddt, *J* = 8.9, 6.3, 1.2 Hz, 2H), 2.43 (t, *J* = 6.9 Hz, 2H), 1.88 – 1.81 (m, 2H), 1.12 – 1.06 (m, 2H), 0.88 – 0.83 (m, 2H), 0.29 (s, 6H) ppm; ^{13}C-NMR (126 MHz, C_6D_6) δ 243.4, 140.4, 135.2, 134.3, 130.0, 128.4, 128.2, 127.4, 126.9, 126.5, 121.1, 48.4, 33.5, 21.5, 5.0, 1.3, -4.7 ppm; IR: ν 1642, 1428, 1249, 1110, 696 cm^{-1}; HRMS (ESI): Exact mass calculated for $C_{22}H_{26}NaOSi^+$ [$(M+Na)^+$] 357.1645, found 357.1644.

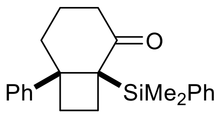

Cyclobutane **2b**.

The title compound was prepared according to general procedure #4 from acylsilane **1b** (58.0 mg, 0.173 mmol). The reaction mixture was stirred at –78 °C for 1 hour. The crude product was purified by flash column chromatography (eluent: grade from 1:2:198 to 1:20:180 NEt_3/hexane/EtOAc). The title compound was isolated as a colorless oil (49.0 mg, 0.146 mmol, 84%).

TLC: R_f = 0.37 (10% EtOAc/hexane, Seebach stain); ^{1}H-NMR (500 MHz, C_6D_6): δ 7.40 – 7.36 (m, 2H), 7.18 – 7.12 (m, 4H), 7.10 – 7.07 (m, 2H), 6.93 – 6.89 (m, 2H), 2.62 (dt, *J* = 11.9, 9.2 Hz, 1H), 2.37 – 2.27 (m, 2H), 2.21 – 2.12 (m, 2H), 1.76 (ddd, *J* = 11.1, 9.2, 3.3 Hz, 1H), 1.68 – 1.59 (m, 2H), 1.53 – 1.44 (m, 1H), 1.27 (ddd, *J* = 8.9, 3.8, 1.5 Hz, 1H), 0.37 (s, 3H), 0.00 (s, 3H) ppm; ^{13}C-NMR (126 MHz, C_6D_6) δ 216.8, 147.9, 137.8, 135.4, 129.6, 128.4, 128.2, 126.8, 126.3, 54.6, 53.3, 38.4, 37.9, 31.6, 27.9, 20.7, -2.6, -4.4 ppm; IR: ν 1674, 1428, 1249, 814, 763, 703 cm^{-1}; HRMS (ESI): Exact mass calculated for $C_{22}H_{26}NaOSi^+$ [$(M+Na)^+$] 357.1645, found 357.1653.

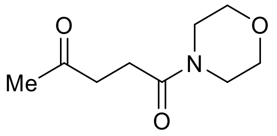

Morpholine amide **S2c**.

The title compound was prepared according to general procedure #2 from commercially available 4-oxopentanoic acid (8.82 mL, 86.0 mmol). The crude product was purified by flash column chromatography (eluent: grade from 10% to 100% EtOAc–hexane). The title compound was isolated as a slightly impure pale yellow solid (10.4 g, 56.1 mmol, 65%) whose spectral data was in accordance with the literature.[7]

^{1}H-NMR (400 MHz, $CDCl_3$): δ 3.67 (t, *J* = 4.8 Hz, 4H), 3.61 – 3.48 (m, 4H), 2.80 (t, *J* = 6.4 Hz, 2H), 2.58 (t, *J* = 6.4 Hz, 2H), 2.22 (d, *J* = 0.4 Hz, 3H) ppm.

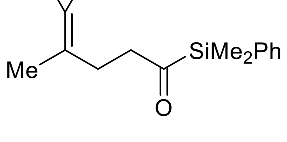

Acylsilane **1c**.

The title compound was prepared according to general procedure #3 from morpholine amide **S2c** (20 mg, 0.11 mmol). The crude product was purified by flash column chromatography (eluent: grade from 1:2:196 to 1:20:180 NEt_3–EtOAc–hexane). The title compound was isolated as a yellow oil (8.0 mg, 31 µmol 28% over 2 steps).

[7] Staveness, D.; Sodano, T. M.; Li, K.; Burnham, E. A.; Jackson, K. D.; Stephenson, C. R. J., *Chem.* **2019**, *5*, 215.

TLC: R$_f$ = 0.75 (30% EtOAc/hexane, Seebach stain); ^{1}H-NMR (500 MHz, C_6D_6): δ 7.48 – 7.44 (m, 2H), 7.19 – 7.11 (m, 3H), 2.74 – 2.67 (m, 2H), 2.44 (tq, *J* = 6.9, 1.6 Hz, 2H), 1.73 – 1.69 (m, 3H), 0.86 – 0.81 (m, 2H), 0.80 – 0.73 (m, 2H), 0.34 (s, 6H) ppm; ^{13}C-NMR (126 MHz, C_6D_6): δ 242.9, 135.2, 134.3, 130.0, 128.4, 123.4, 115.4, 46.9, 29.3, 21.1, 3.3, 1.3, -4.7 ppm; IR: ν 2972, 1644, 1428, 1249, 1110, 835, 817, 781, 670 cm^{-1}; HRMS (ESI): Exact mass calculated for $C_{16}H_{22}NaOSi^+$ [$(M+Na)^+$] 281.1332, found 281.1329.

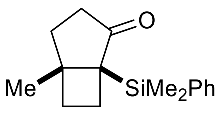

Cyclobutane **2c**.

The title compound was prepared according to general procedure #4 from acylsilane **1c** (60 mg, 2.3 mmol). The reaction mixture was stirred at –78 °C to –20 °C for 3 hours. The crude product was purified by flash column chromatography (eluent: grade from 1:2:198 to 1:20:180 NEt_3/hexane/EtOAc). The title compound was isolated as a colorless oil (48.3 mg, 1.87 mmol, 81%).

TLC: R$_f$ = 0.47 (10% EtOAc/hexane, Seebach stain); ^{1}H-NMR (500 MHz, C_6D_6): δ 7.53 – 7.49 (m, 2H), 7.18 – 7.15 (m, 3H), 2.28 – 2.18 (m, 2H), 2.06 (ddd, *J* = 18.5, 10.5, 4.9 Hz, 1H), 1.91 (ddd, *J* = 11.4, 9.0, 4.2 Hz, 1H), 1.74 (dt, *J* = 11.6, 8.7 Hz, 1H), 1.44 (ddd, *J* = 11.6, 9.3, 4.2 Hz, 1H), 1.32 (ddd, *J* = 13.2, 9.1, 4.9 Hz, 1H), 1.13 (ddd, *J* = 13.2, 10.5, 8.7 Hz, 1H), 0.99 (s, 3H), 0.43 (s, 3H), 0.40 (s, 3H) ppm; ^{13}C-NMR (126 MHz, C_6D_6): δ 222.6, 137.6, 134.6, 129.5, 128.4, 51.3, 48.5, 39.1, 34.8, 33.1, 25.5, 24.9, -3.7, -3.9 ppm; IR: ν 2949, 1711, 1258, 836, 811, 702 cm^{-1}; HRMS (ESI): Exact mass calculated for $C_{16}H_{22}NaOSi^+$ [$(M+Na)^+$] 281.1332, found 281.1335.

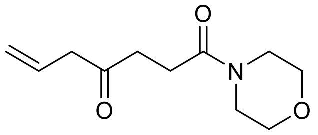

Morpholine amide **S2d**.

The title compound was prepared according to general procedure #1 from aldehyde **S5-1** (500 mg, 2.92 mmol) and allylmagnesium chloride in THF (1.90 mL, 2.0 M, 3.8 mmol, 1.3 equiv). The Grignard addition reaction was stirred at –78 °C for 30 minutes and was then allowed to gradually warm to –30 °C over 2 h. The crude ketoamide **S2d** was purified by flash column chromatography (eluent: grade from 1% to 10% MeOH–CH_2Cl_2). The title compound (252 mg, 1.19 mmol, 41% over 2 steps) was isolated as a colorless oil.

TLC: R$_f$ = 0.52 (10% MeOH/DCM, $KMnO_4$ stain); ^{1}H-NMR (400 MHz, $CDCl_3$): δ 5.95 (ddtd, *J* = 17.2, 10.3, 7.0, 0.4 Hz, 1H), 5.23 – 5.12 (m, 2H), 3.71 – 3.64 (m, 4H), 3.61 – 3.57 (m, 2H), 3.51 (dd, *J* = 5.6, 4.2 Hz, 2H), 3.27 (dt, *J* = 7.0, 1.2 Hz, 2H), 2.83 – 2.77 (m, 2H), 2.59 (t, *J* = 6.4 Hz, 2H) ppm; ^{13}C-NMR (101 MHz, $CDCl_3$): δ 208.0, 170.3, 130.6, 119.1, 67.0, 66.7, 48.0, 45.9, 42.2, 36.9, 26.9 ppm; IR: ν 1714, 1639, 1436, 1230, 1114, 1042 cm^{-1}; HRMS (ESI): Exact mass calculated for $C_{11}H_{17}NNaO_3^+$ [$(M+Na)^+$] 234.1101, found 234.1098.

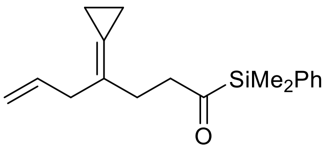

Acylsilane **1d**.

The title compound was prepared according to general procedure #3 from ketoamide **S2d** (126 mg, 0.596 mmol). The crude acylsilane **1d** was purified by flash column chromatography (eluent: grade from 1:2:198 to 1:20:180 NEt_3/hexane/EtOAc). The title compound was isolated as a yellow oil (48.3 mg, 0.170 mmol, 29% over 2 steps).

TLC: R_f = 0.68 (10% EtOAc/hexane, Seebach stain); ^{1}H-NMR (400 MHz, C_6D_6): δ 7.50 – 7.41 (m, 2H), 7.21 – 7.13 (m, 3H), 5.83 (ddtd, *J* = 16.9, 10.0, 6.8, 1.2 Hz, 1H), 5.06 – 4.94 (m, 2H), 2.86 – 2.82 (m, 2H), 2.74 (t, *J* = 7.4 Hz, 2H), 2.52 – 2.44 (m, 2H), 0.79 (app. s, 4H), 0.34 (s, 6H) ppm; ^{13}C-NMR (101 MHz, C_6D_6): δ 242.8, 136.9, 135.2, 134.3, 130.0, 128.4, 125.5, 116.5, 115.5, 46.9, 40.7, 27.4, 2.7, 1.5, -4.7 ppm; IR: ν 2975, 1644, 1429, 1250, 1111, 835, 782, 700 cm^{-1}; HRMS (ESI): Exact mass calculated for $C_{18}H_{24}NaOSi^+$ [$(M+Na)^+$] 307.1489, found 307.1491.

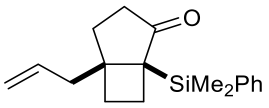

Cyclobutane **2d**.

The title compound was prepared according to general procedure #4 from acylsilane **1d** (29.4 mg, 0.10 mmol). The reaction mixture was stirred at –78 °C to 0 °C to 3 hours before it was stirred at 0 °C for 12 hours. The crude product was purified by flash column chromatography (eluent: grade from 1:2:198 to 1:20:180 NEt_3/hexane/EtOAc). The title compound was isolated as a colorless oil (18.3 mg, 0.064 mmol, 62%).

TLC: R_f = 0.46 (10% EtOAc/hexane, Seebach stain); ^{1}H-NMR (500 MHz, C_6D_6): δ 7.53 – 7.48 (m, 2H), 7.18 – 7.13 (m, 3H), 5.45 (ddt, *J* = 17.2, 10.2, 7.1 Hz, 1H), 4.94 – 4.86 (m, 2H), 2.37 (ddt, *J* = 13.1, 7.1, 1.3 Hz, 1H), 2.27 – 2.18 (m, 2H), 2.11 – 2.02 (m, 2H), 1.94 (ddd, *J* = 11.5, 8.2, 5.3 Hz, 1H), 1.70 – 1.63 (m, 2H), 1.38 (ddd, *J* = 13.3, 9.1, 5.4 Hz, 1H), 1.25 (ddd, *J* = 13.4, 10.5, 8.2 Hz, 1H), 0.42 (s, 3H), 0.40 (s, 3H) ppm; ^{13}C-NMR (126 MHz, C_6D_6): δ 222.2, 137.6, 134.6, 134.5, 129.6, 128.1, 117.2, 51.6, 51.2, 43.4, 38.9, 32.0, 30.5, 25.2, -3.5, -3.8 ppm; IR: ν 1709, 1428, 1249, 836, 811, 702 cm^{-1}; HRMS (ESI): Exact mass calculated for $C_{18}H_{24}NaOSi^+$ [$(M+Na)^+$] 307.1489, found 307.1489.

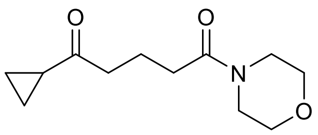

Morpholine amide **S2e**.

The title compound was prepared according to general procedure #1 from aldehyde **S5-2** (500 mg, 2.70 mmol) and cyclopropylmagnesium bromide in 2-methyltetrahydrofuran (3.5 mL, 1.0 M, 3.5 mmol, 1.3 equiv). The Grignard addition reaction was stirred at –78 °C for 30 minutes, then at –78 °C to –30 °C for 1 h and then at room temperature for 1 h. The crude ketoamide **S2e** was purified

by flash column chromatography (eluent: grade from 1% to 10% MeOH–CH_2Cl_2). The title compound (417 mg, 1.85 mmol, 69% over 2 steps) was isolated as a colorless oil.

TLC: R_f = 0.52 (10% MeOH/DCM, $KMnO_4$ stain); ^{1}H-NMR (500 MHz, $CDCl_3$): δ 3.69 – 3.64 (m, 4H), 3.62 – 3.59 (m, 2H), 3.50 – 3.46 (m, 2H), 2.68 (t, *J* = 6.7 Hz, 2H), 2.34 (t, *J* = 7.4 Hz, 2H), 1.97 – 1.89 (m, 3H), 1.03 – 0.98 (m, 2H), 0.89 – 0.84 (m, 2H) ppm; ^{13}C-NMR (126 MHz, $CDCl_3$): δ 210.8, 171.4, 67.1, 66.9, 46.1, 42.5, 42.0, 32.4, 20.6, 19.5, 10.9 ppm; IR: ν 1694, 1641, 1436, 1236, 1114 cm^{-1}; HRMS (ESI): Exact mass calculated for $C_{12}H_{20}NO_3^+$ [(M+H)$^+$] 226.1438, found 226.1441.

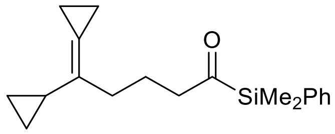

Acylsilane **1e**.

The title compound was prepared according to general procedure #3 from ketoamide **S2e** (209 mg, 0.925 mmol). The crude acylsilane **1e** was purified by flash column chromatography (eluent: grade from 1:2:198 to 1:20:180 NEt_3/hexane/EtOAc). The title compound was isolated as a pale yellow oil (58.8 mg, 0.197 mmol, 21% over 2 steps). It should be noted that the first step (olefination of ketone **S2e**) was plagued by poor conversion and 47% of starting material (**S2e**) was recovered from this reaction.

TLC: R_f = 0.67 (10% EtOAc/hexane, Seebach stain); ^{1}H-NMR (500 MHz, C_6D_6): δ 7.47 – 7.43 (m, 2H), 7.16 – 7.14 (m, 3H), 2.46 (t, *J* = 7.1 Hz, 2H), 2.06 (ddt, *J* = 7.7, 6.1, 1.4 Hz, 2H), 1.92 – 1.83 (m, 2H), 1.32 – 1.25 (m, 1H), 0.93 – 0.86 (m, 2H), 0.77 – 0.71 (m, 2H), 0.62 – 0.58 (m, 2H), 0.53 – 0.49 (m, 2H), 0.33 (s, 6H) ppm; ^{13}C-NMR (126 MHz, C_6D_6): δ 243.6, 135.3, 134.3, 130.0, 128.4, 128.4, 113.6, 48.8, 34.9, 21.2, 15.6, 5.5, 2.3, 0.5, -4.7 ppm; IR: ν 1643, 1428, 1249, 1111, 815, 700 cm^{-1}; HRMS (ESI): Exact mass calculated for $C_{19}H_{26}NaOSi^+$ [(M+Na)$^+$] 321.1645, found 321.1646.

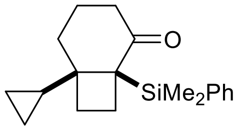

Cyclobutane **2e**.

The title compound was prepared according to general procedure #4 from acylsilane **1e** (39.7 mg, 0.133 mmol). The reaction mixture was stirred at –78 °C for 1 hour. The crude product was purified by flash column chromatography (eluent: grade from 1:2:198 to 1:20:180 NEt_3/hexane/EtOAc). The title compound was isolated as a colorless oil (33.8 mg, 0.113 mmol, 85%).

TLC: R_f = 0.23 (10% EtOAc/hexane, Seebach stain); ^{1}H-NMR (400 MHz, C_6D_6): δ 7.50 – 7.44 (m, 2H), 7.20 – 7.15 (m, 3H), 2.56 (dddd, *J* = 10.1, 8.8, 2.9, 1.1 Hz, 1H), 2.24 (ddd, *J* = 13.2, 4.8, 3.6 Hz, 1H), 2.11 (ddd, *J* = 10.8, 9.5, 8.3 Hz, 1H), 1.83 – 1.73 (m, 1H), 1.61 – 1.51 (m, 1H), 1.51 – 1.43 (m, 2H), 1.23 – 1.14 (m, 1H), 1.08 (dtd, *J* = 14.0, 4.7, 1.2 Hz, 1H), 0.95 – 0.82 (m, 2H), 0.41 (s, 3H), 0.37 (s, 3H), 0.31 – 0.24 (m, 2H), 0.16 – 0.10 (m, 1H), -0.15 – -0.22 (m, 1H) ppm; ^{13}C-NMR (101 MHz, C_6D_6): δ 214.5, 138.0, 134.8, 129.4, 128.0, 58.1, 51.5, 41.5, 34.7, 27.8, 25.5, 22.7, 19.6, 5.0, 2.4, -2.2, -3.4 ppm; IR: ν 1675, 1428, 1250, 812, 703 cm^{-1}; HRMS (ESI): Exact mass calculated for $C_{19}H_{26}NaOSi^+$ [(M+Na)$^+$] 321.1645, found 321.1644.

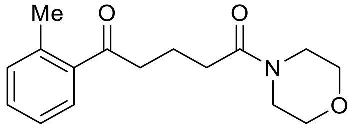

Morpholine amide **S2f**.

The title compound was prepared according to general procedure #1 from aldehyde **S5-2** (500 mg, 2.70 mmol) and *o*-tolylmagnesium chloride in THF (3.9 mL, 0.9 M, 3.5 mmol, 1.3 equiv). The Grignard addition reaction was stirred at –78 °C for 30 minutes and was then allowed to gradually warm to –30 °C over 1 h 30 minutes. The crude ketoamide **S2f** was purified by flash column chromatography (eluent: grade from 1% to 10% MeOH–CH_2Cl_2). The title compound (508 mg, 1.85 mmol, 69% over 2 steps) was isolated as a colorless oil.

TLC: R_f = 0.52 (10% MeOH/CH_2Cl_2, $KMnO_4$ stain); ^{1}H-NMR (500 MHz, $CDCl_3$): δ 7.66 (dd, *J* = 7.5, 1.0 Hz, 1H), 7.36 (td, *J* = 7.5, 1.4 Hz, 1H), 7.25 – 7.22 (m, 2H), 3.69 – 3.64 (m, 4H), 3.63 – 3.59 (m, 2H), 3.52 – 3.46 (m, 2H), 3.01 (t, *J* = 6.8 Hz, 2H), 2.49 (s, 3H), 2.42 (t, *J* = 7.2 Hz, 2H), 2.05 (p, *J* = 7.0 Hz, 2H) ppm; ^{13}C-NMR (126 MHz, $CDCl_3$): δ 204.1, 171.3, 138.1, 137.9, 132.1, 131.5, 128.7, 125.9, 67.0, 66.8, 46.1, 42.0, 40.6, 32.3, 21.5, 19.9 ppm; IR: ν 1683, 1641, 1432, 1231, 1115, 757 cm^{-1}; HRMS (ESI): Exact mass calculated for $C_{16}H_{21}NNaO_3^+$ [$(M+Na)^+$] 298.1414, found 298.1413.

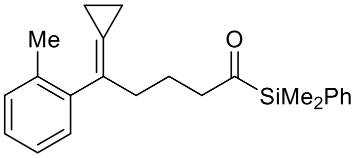

Acylsilane **1f**.

The title compound was prepared according to general procedure #3 from ketoamide **S2f** (254 mg, 0.922 mmol). The crude acylsilane **1f** was purified by flash column chromatography (eluent: grade from 1:2:198 to 1:20:180 NEt_3/hexane/EtOAc). The title compound was isolated as a yellow oil (28.0 mg, 80 µmol, 9% over 2 steps). It should be noted that the first step (olefination of ketone **S2f**) was plagued by poor conversion and 72% of starting material (**S2f**) was recovered from this reaction.

TLC: R_f = 0.63 (30% EtOAc–hexane, Seebach stain); ^{1}H-NMR (500 MHz, CD_2Cl_2): δ 7.52 – 7.49 (m, 2H), 7.41 – 7.34 (m, 3H), 7.17 – 7.07 (m, 3H), 7.02 – 7.00 (m, 1H), 2.56 (dd, *J* = 7.6, 7.0 Hz, 2H), 2.39 (ddt, *J* = 9.1, 6.4, 1.4 Hz, 2H), 2.15 (s, 3H), 1.60 – 1.53 (m, 2H), 1.14 – 1.09 (m, 2H), 0.95 – 0.90 (m, 2H), 0.43 (s, 6H) ppm; ^{13}C-NMR (126 MHz, CD_2Cl_2): δ 246.0, 142.6, 136.2, 135.3, 134.5, 130.6, 130.3, 129.3, 128.9, 128.6, 127.0, 125.8, 121.3, 49.1, 36.6, 21.0, 20.2, 3.2, 3.1, -4.5 ppm; IR: ν 1643, 1428, 1249, 1111, 835, 701 cm^{-1}; HRMS (ESI): Exact mass calculated for $C_{23}H_{28}NaOSi^+$ [$(M+Na)^+$] 371.1802, found 371.1802.

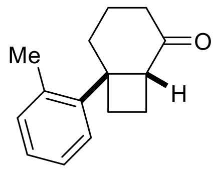

Cyclobutane **2f**.

The title compound was prepared according to general procedure #4 from acylsilane **1f** (28.0 mg, 0.080 mmol). The reaction mixture was stirred at –78 °C for 17 hours. The crude product was purified by flash column chromatography (eluent: grade from 1:2:198 to 1:20:180 NEt_3/hexane/EtOAc). The title compound was isolated as a colorless oil (9.3 mg, 0.043 mmol, 54%).

TLC: R_f = 0.29 (10% EtOAc–hexane, Seebach stain); ^{1}H-NMR (400 MHz, C_6D_6): δ 7.03 – 6.92 (m, 3H), 6.87 – 6.82 (m, 1H), 3.23 (t, J = 9.5 Hz, 1H), 2.28 – 2.20 (m, 1H), 2.18 – 2.07 (m, 1H), 1.99 (s, 3H), 1.93 – 1.72 (m, 4H), 1.64 – 1.56 (m, 2H), 1.35 – 1.27 (m, 2H) ppm; ^{13}C-NMR (101 MHz, C_6D_6): δ 210.0, 146.7, 134.5, 131.7, 127.1, 126.6, 126.0, 51.1, 49.3, 38.9, 33.7, 33.5, 21.6, 20.9, 20.1 ppm; IR: ν 2937, 1698, 1457, 755, 730 cm^{-1}; HRMS (ESI): Exact mass calculated for $C_{15}H_{18}NaO^+$ [(M+Na)$^+$] 237.1250, found 237.1255.

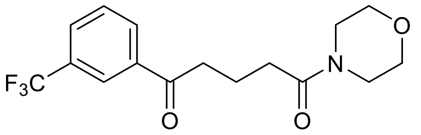

Morpholine amide **S2g**.

The title compound was prepared according to general procedure #1 from aldehyde **S5-2** (501 mg, 2.70 mmol) and Grignard reagent prepared from metalation of commercially available 3-iodobenzotrifluoride (0.60 mL, 4.1 mmol, 1.5 equiv) and a solution of Turbo Grignard in THF (3.11 mL, 1.3 M, 4.04 mmol, 1.5 equiv) at –10 °C for 32 minutes. The Grignard addition reaction was stirred at –10 °C for 1 hour 30 minutes. The crude ketoamide **S2g** was purified by flash column chromatography (eluent: 80% EtOAc–hexane). The title compound (512 mg, 1.55 mmol, 57% over 2 steps) was isolated as a pale yellow solid.

TLC: R_f = 0.64 (10%MeOH/CH_2Cl_2, $KMnO_4$ stain); ^{1}H-NMR (400 MHz, $CDCl_3$): δ 8.23 (s, 1H), 8.17 (d, J = 7.8 Hz, 1H), 7.82 (d, J = 7.8 Hz, 1H), 7.61 (t, J = 7.8 Hz, 1H), 3.70 – 3.66 (m, 4H), 3.64 – 3.60 (m, 2H), 3.53 – 3.48 (m, 2H), 3.15 (t, J = 6.7 Hz, 2H), 2.45 (t, J = 7.1 Hz, 2H), 2.10 (p, J = 6.9 Hz, 2H) ppm; ^{13}C-NMR (101 MHz, $CDCl_3$):[8] δ 198.7, 171.1, 137.4, 131.4, 129.7 (q, J = 3.6 Hz), 129.5, 125.0 (q, J = 3.9 Hz), 67.0, 66.8, 46.0, 42.0, 37.9, 32.1, 19.4 ppm; ^{19}F-NMR (376 MHz, $CDCl_3$): δ -62.8 ppm; IR: ν 2858, 1691, 1645, 1330, 1117, 696 cm^{-1}; HRMS (ESI): Exact mass calculated for $C_{16}H_{19}F_3NO_3^+$ [(M+H)$^+$] 330.1312, found 330.1311.

[8] The ^{13}C peaks of the trifluoromethyl group and the quaternary aromatic carbon atom adjacent to the trifluoromethyl group could not be identified due to their low intensities.

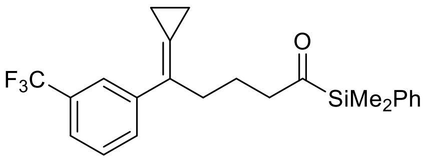

Acylsilane **1g**.

The title compound was prepared according to general procedure #3 from morpholine amide **S2g** (249 mg, 757 µmol). The crude product was purified by flash column chromatography (eluent: grade from 1:2:196 to 1:20:180 NEt_3–EtOAc–hexane). The title compound was isolated as a yellow oil (46.4 mg, 115 µmol, 15% over 2 steps).

TLC: R_f = 0.83 (30% EtOAc/hexane, Seebach stain); ^{1}H-NMR (500 MHz, C_6D_6): δ 8.00 (s, 1H), 7.53 (d, *J* = 9.3 Hz, 1H), 7.43 – 7.40 (m, 2H), 7.30 (d, *J* = 7.8 Hz, 1H), 7.23 – 7.19 (m, 1H), 7.17 – 7.14 (m, 2H), 7.03 (t, *J* = 7.8 Hz, 1H), 2.42 (ddt, *J* = 9.0, 6.3, 1.2 Hz, 2H), 2.38 (t, *J* = 6.8 Hz, 2H), 1.78 – 1.70 (m, 2H), 0.99 – 0.94 (m, 2H), 0.82 – 0.76 (m, 2H), 0.30 (s, 6H) ppm; ^{13}C-NMR (126 MHz, C_6D_6)[8]: δ 243.3, 141.3, 135.1, 134.3, 130.1, 129.4, 129.1, 128.4, 126.2, 123.4, 123.4 (q, *J* = 3.7 Hz), 123.1 (q, *J* = 3.9 Hz), 48.2, 33.1, 21.2, 4.7, 1.4, -4.8 ppm; ^{19}F-NMR (376 MHz, C_6D_6): δ -62.2 ppm; IR: ν 2957, 1642, 1333, 1249, 1123, 698 cm^{-1}; HRMS (ESI): Exact mass calculated for $C_{23}H_{25}F_3NaOSi^+$ [$(M+Na)^+$] 425.1519, found 425.1520.

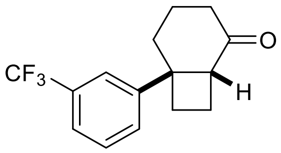

Cyclobutane **2g**.

The title compound was prepared according to general procedure #4 from acylsilane **1g** (42.3 mg, 105 µmol) using 40 mol% $BF_3{\cdot}OEt_2$. The reaction mixture was stirred at –78 °C for 16 hours. The crude product was purified by flash column chromatography (eluent: grade from 1:2:198 to 1:10:190 NEt_3/hexane/EtOAc). The title compound was isolated as a colorless oil (22 mg, 82 µmol, 78%).

TLC: R_f = 0.25 (10% EtOAc/hexane, Seebach stain); ^{1}H-NMR (400 MHz, C_6D_6): δ 7.30 (d, *J* = 2.0 Hz, 1H), 7.23 (d, *J* = 7.7 Hz, 1H), 6.88 (t, *J* = 7.7 Hz, 1H), 6.81 (d, *J* = 7.8 Hz, 1H), 2.97 (t, *J* = 9.1 Hz, 1H), 2.12 – 2.05 (m, 2H), 1.85 – 1.60 (m, 4H), 1.45 (ddd, *J* = 14.4, 10.9, 2.8 Hz, 1H), 1.34 – 1.16 (m, 3H) ppm; ^{13}C-NMR (101 MHz, C_6D_6): δ 209.6, 151.3, 131.0 (q, *J* = 31.8 Hz), 129.1, 128.9, 125.0 (d, *J* = 272.2 Hz), 122.9 (q, *J* = 3.8 Hz), 122.1 (q, *J* = 3.7 Hz), 49.8, 47.9, 38.7, 36.8, 31.9, 21.2, 20.6 ppm; ^{19}F-NMR (377 MHz, C_6D_6): δ -62.2 ppm; IR: ν 2924, 1700, 1322, 1121, 802, 704 cm^{-1}; HRMS (ESI): Exact mass calculated for $C_{15}H_{15}F_3NaO^+$ [$(M+Na)^+$] 291.0967, found 291.0969.

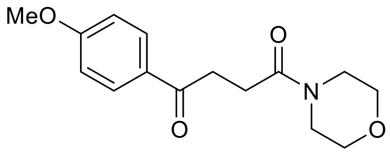

Morpholine amide **S2h**.

The title compound was prepared according to general procedure #1 from aldehyde **S5-1** (500 mg, 2.92 mmol) and *para*-methoxyphenylmagnesium bromide in THF (3.80 mL, 1.0 M, 3.8 mmol,

1.3 equiv). The Grignard addition reaction was stirred at –78 °C for 30 minutes and was then allowed to gradually warm to 0 °C over 2 h. The crude ketoamide **S2h** was purified by flash column chromatography (eluent: grade from 1% to 10% MeOH–CH_2Cl_2). The title compound (434 mg, 1.57 mmol, 74% over 2 steps) was isolated as a colorless oil.

TLC: R_f = 0.53 (10% MeOH/CH_2Cl_2, $KMnO_4$ stain); ^{1}H-NMR (500 MHz, $CDCl_3$): δ 8.00 (d, *J* = 9.0 Hz, 2H), 6.94 (d, *J* = 9.0 Hz, 2H), 3.87 (s, 3H), 3.74 – 3.70 (m, 2H), 3.69 – 3.66 (m, 2H), 3.64 – 3.61 (m, 2H), 3.60 – 3.57 (m, 2H), 3.32 (t, *J* = 6.6 Hz, 2H), 2.76 (t, *J* = 6.6 Hz, 2H) ppm; ^{13}C-NMR (126 MHz, $CDCl_3$): δ 197.7, 170.8, 163.7, 130.5, 130.0, 113.9, 67.0, 66.8, 55.6, 46.0, 42.3, 33.3, 27.1 ppm; IR: ν 1642, 1428, 1250, 1116, 829 cm^{-1}; HRMS (ESI): not found.

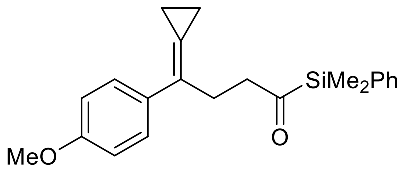

Acylsilane **1h**.

The title compound was prepared according to general procedure #3 from ketoamide **S2h** (217 mg, 0.783 mmol). The crude acylsilane **1h** was purified by flash column chromatography (eluent: grade from 1:2:198 to 1:20:180 NEt_3/hexane/EtOAc). The title compound was isolated as a yellow oil (30.9 mg, 0.138 mmol, 18% over 2 steps). It should be noted that the first step (olefination of ketone **S2h**) was plagued by poor conversion and 39% of starting material **S2h** was recovered from this reaction.

TLC: R_f = 0.39 (10% EtOAc/hexane, Seebach stain); ^{1}H-NMR (500 MHz, C_6D_6): δ 7.47 (d, *J* = 8.8 Hz, 2H), 7.44 – 7.39 (m, 2H), 7.15 – 7.11 (m, 3H), 6.81 (d, *J* = 8.9 Hz, 2H), 3.34 (s, 3H), 3.04 – 2.98 (m, 2H), 2.87 – 2.82 (m, 2H), 1.07 (ddt, *J* = 9.0, 4.9, 1.5 Hz, 2H), 0.86 (ddt, *J* = 8.0, 5.6, 1.3 Hz, 2H), 0.30 (s, 6H) ppm; ^{13}C-NMR (126 MHz, C_6D_6): δ 243.4, 159.1, 135.1, 134.3, 132.7, 130.0, 128.4, 127.5, 126.3, 118.7, 114.1, 54.8, 47.7, 27.0, 4.7, 1.3, -4.8 ppm; IR: ν 1511, 1248, 1179, 1111, 830, 701 cm^{-1}; HRMS (ESI): Exact mass calculated for $C_{22}H_{26}NaO_2Si^+$ [$(M+Na)^+$] 373.1594, found 373.1600.

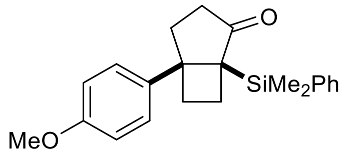

Cyclobutane **2h**.

The title compound was prepared according to general procedure #4 from acylsilane **1h** (24.1 mg, 0.069 mmol). The reaction mixture was stirred at –78 °C for 17 hours. The crude product was purified by flash column chromatography (eluent: grade from 1:2:198 to 1:20:180 NEt_3/hexane/EtOAc). The title compound was isolated as a colorless oil (10.3 mg, 0.029 mmol, 43%).

TLC: R_f = 0.30 (10% EtOAc/hexane, Seebach stain); ^{1}H-NMR (500 MHz, C_6D_6): δ 7.45 – 7.39 (m, 2H), 7.17 – 7.11 (m, 3H), 6.79 (d, *J* = 8.8 Hz, 2H), 6.68 (d, *J* = 8.9 Hz, 2H), 3.35 (s, 3H), 2.46 – 2.40 (m, 2H), 2.40 – 2.34 (m, 1H), 2.21 (ddd, *J* = 11.9, 9.5, 4.8 Hz, 1H), 2.02 (ddd, *J* = 11.8, 8.7, 7.5 Hz, 1H), 1.91 – 1.79 (m, 2H), 1.75 (ddd, *J* = 11.4, 8.7, 4.7 Hz, 1H), 0.34 (s, 3H), 0.13 (s, 3H) ppm; ^{13}C-NMR (126 MHz,

C_6D_6): δ 221.6, 158.6, 139.4, 137.4, 135.3, 129.3, 127.7, 127.3, 113.8, 54.8, 52.8, 52.8, 40.7, 38.0, 30.8, 25.3, -3.5, -4.6 ppm; IR: ν 1709, 1514, 1250, 1180, 829, 812, 703 cm^{-1}; HRMS (ESI): Exact mass calculated for $C_{22}H_{26}NaO_2Si^+$ [$(M+Na)^+$] 373.1594, found 373.1588.

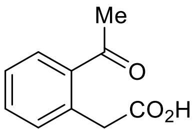

2-(2-Acetylphenyl)acetic acid (**S1i**).

A 250 mL one-necked round bottom flask equipped with a magnetic stir bar was charged with 1-methylindene[9] (2.1 g, 16 mmol, 1.0 equiv) in hexane-MeCN-H_2O (2:2:3, 96 mL) at room temperature. $NaIO_4$ (14 g, 65 mmol, 4.1 equiv) and $RuCl_3{\cdot}H_2O$ (60 mg, 0.27 mmol, 1.7 mol%) were successively added and the biphasic mixture was stirred vigorously for 12 hours. The reaction mixture was poured into CH_2Cl_2 (200 mL) and 6M aq HCl (100 mL). The aqueous phase was extracted twice with CH_2Cl_2. The organic layers were basified with 5% aq NaOH solution. The aqueous layers were then acidified with conc HCl (25 mL, final pH < 1) and extracted twice with CH_2Cl_2 (2 x 200 mL). The organic layers were washed with brine, combined and dried over $MgSO_4$. Concentration *in vacuo* afforded a light brown solid (1.2 g, 6.5 mmol, 41%). The spectroscopic data matched those reported in the literature.[10]

^{1}H NMR (400 MHz, $CDCl_3$) δ 7.84 (dd, *J* = 7.8, 1.4 Hz, 1H), 7.53 (td, *J* = 7.5, 1.4 Hz, 1H), 7.46 – 7.38 (m, 2H), 3.87 (s, 2H), 2.68 (s, 3H).

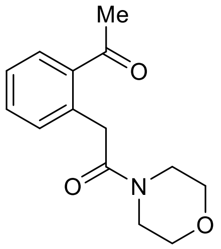

Morpholine amide **S2i**.

A 100 mL one-necked round bottom flask equipped with a magnetic stir bar was charged with carboxylic acid **S1i** (1.2 g, 6.5 mmol, 1.0 equiv) under N_2 atmosphere. Dry DMF (22 mL) was added and the pale brown solution was cooled to 0 °C. DIPEA (2.3 mL, 13 mmol, 2.0 equiv), morpholine (0.74 mL, 8.5 mmol, 1.3 equiv) and HATU (3.2 g, 8.5 mmol, 1.3 equiv) were successively added and the dark brown solution was stirred for 7.5 hours. The ice bath was removed after 3 minutes. Water and EtOAc were added at 0 °C. The aqueous phase was extracted twice with EtOAc. The organic layers were washed three times with water-brine (2:1), combined and dried over $MgSO_4$. Concentration *in vacuo* afforded a dark brown oil. Flash column chromatography (eluent: 1:3 Hexane-EtOAc) furnished the title compound as a dark red oil (0.85 g, 3.4 mmol, 53%).

TLC: R_f = 0.10 (Hexane–EtOAc 1:2, $KMnO_4$/UV); ^{1}H-NMR (500 MHz, $CDCl_3$) δ 7.80 (dd, *J* = 7.7, 1.4 Hz, 1H), 7.46 (td, *J* = 7.5, 1.4 Hz, 1H), 7.40 – 7.34 (m, 1H), 7.24 (ddd, *J* = 7.6, 1.4, 0.6 Hz, 1H), 4.01 (d, *J* = 1.1

[9] Phatake, R. S.; Ramana, C. V. *Tetrahedron Lett.* **2015**, *56*, 3868–3871.
[10] Masuda, Y.; Ishida, N.; Murakami, M. *J. Am. Chem. Soc.* **2015**, *137*, 14063–14066.

Hz, 2H), 3.71 (s, 4H), 3.62 (t, J = 4.8 Hz, 4H), 2.59 (s, 3H); ^{13}C-NMR (126 MHz, $CDCl_3$) δ 201.6, 169.8, 137.1, 135.5, 132.4, 132.0, 130.0, 127.1, 66.8, 66.7, 46.2, 42.3, 38.6, 29.0; IR (thin film): 2965, 2920, 2856, 1681, 1645, 1574, 1432, 1357, 1299, 1258, 1230, 1114, 1069, 1036, 960, 848, 762, 723, 597, 581 cm^{-1}; HRMS (ESI): exact mass calculated for $C_{14}H_{17}NNaO_3$ [$(M+Na)^+$] 270.1101, found 270.1099.

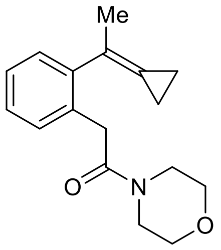

Morpholine amide **S3i**.

A 50 mL one-necked pear-shaped flask equipped with a magnetic stir bar was charged with ketone **S2i** (0.25 g, 1.0 mmol, 1.0 equiv) under Ar atmosphere. $NaHCO_3$ (0.42 g, 5.0 mmol, 5.0 equiv), dry PhMe (10 mL) and $Cp_2Ti(C_3H_5)_2$ in PhMe (0.60 M, 3.7 mL, 2.2 mmol, 2.2 equiv) were successively added and the red mixture was stirred for 30 minutes; during this time it became a dark red/maroon suspension. The reaction mixture was allowed to cool to r.t. Flash column chromatography (eluent: 3:200:400 Et_3N–Hexane–EtOAc) furnished the title compound as a yellow oil (0.14 g, 0.53 mmol, 53%).

TLC: R_f = 0.24 (Hexane–EtOAc 1:1, $KMnO_4$/UV); ^{1}H-NMR (400 MHz, C_6D_6) δ 7.50 – 7.43 (m, 1H), 7.15 – 7.11 (m, 1H), 7.13 – 7.06 (m, 2H), 3.54 (s, 2H), 3.46 (t, J = 4.9 Hz, 2H), 3.24 (t, J = 4.9 Hz, 2H), 2.98 (t, J = 4.8 Hz, 2H), 2.73 (t, J = 4.9 Hz, 2H), 2.06 (p, J = 1.7 Hz, 3H), 0.98 – 0.92 (m, 2H), 0.81 (tt, J = 6.4, 2.5 Hz, 2H); ^{13}C-NMR (101 MHz, C_6D_6) δ 169.4, 143.0, 133.3, 129.5, 128.6, 127.4, 127.0, 125.0, 121.9, 66.8, 66.3, 46.2, 42.2, 38.8, 23.0, 4.3, 2.6; IR (thin film): 2972, 2916, 2854, 1647, 1430, 1362, 1272, 1229, 1167, 1115, 1069, 1036, 966, 848, 760, 729, 584 cm^{-1}; HRMS (ESI): exact mass calculated for $C_{17}H_{22}NO_2$ [$(M+H)^+$] 272.1645, found 272.1644.

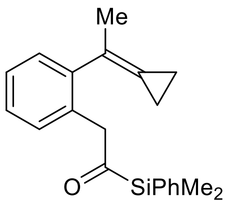

Acylsilane **1i**.

A 25 mL one-necked pear-shaped flask equipped with a magnetic stir bar was charged with morpholine amide **S3i** (0.10 g, 0.38 mmol, 1.0 equiv) under Ar atmosphere. Dry THF (3.8 mL) was added and the pale yellow solution was cooled to –78 °C. $PhMe_2SiLi$ in THF (1.49 M, 0.51 mL, 0.76 mmol, 2.0 equiv) was added dropwise and the dark red solution was stirred for 1 hour. Sat aq $NaHCO_3$ solution and EtOAc were successively added at –78 °C. The aqueous phase was extracted twice with EtOAc. The organic layers were combined and dried over $MgSO_4$. Concentration *in vacuo* afforded a yellow oil. Flash column chromatography (eluent: 3:4:600 Et_3N–Hexane-EtOAc) furnished the title compound as a colorless oil (37 mg, 0.11 mmol, 30%). The material is unstable on silica gel (rearrangement to α-silyl ketone **2i**, which was isolated as a colorless oil (0.021 g, 0.066 mmol, 17%)).

TLC: R_f = 0.53 (Hexane–EtOAc 10:1, $KMnO_4$/UV); ^{1}H-NMR (500 MHz, C_6D_6) δ 7.56 – 7.43 (m, 1H), 7.40 – 7.37 (m, 1H), 7.27 – 7.11 (m, 4H), 7.09 (td, J = 7.4, 1.5 Hz, 1H), 7.03 (td, J = 7.4, 1.5 Hz, 1H), 6.96 (ddd, J = 7.6, 1.5, 0.6 Hz, 1H), 3.83 (s, 2H), 2.04 (p, J = 1.7 Hz, 3H), 0.89 – 0.82 (m, 2H), 0.82 – 0.75 (m, 2H), 0.25 (s, 6H); ^{13}C-NMR (126 MHz, C_6D_6) δ 239.7, 144.1, 135.1, 134.3, 131.8, 131.4, 130.0, 128.6, 128.4, 127.0, 126.9, 125.4, 121.3, 53.8, 23.1, 4.4, 2.4, -4.5; IR (thin film): 3049, 2971, 2912, 1690, 1652, 1634, 1488, 1428, 1302, 1249, 1157, 1110, 1068, 992, 833, 817, 779, 762, 700, 650, 581, 466, 414 cm^{-1}; HRMS (ESI): exact mass calculated for $C_{21}H_{24}NaOSi$ [(M+Na)$^+$] 343.1489, found 343.1490.

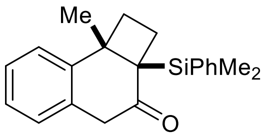

Cyclobutane **2i**.

The title compound was prepared according to general procedure #4 from acylsilane **1i** (12.6 mg, 0.039 mmol). The reaction mixture was stirred at –78 °C for 100 minutes. The crude product was purified by flash column chromatography on silica gel (eluent: 80:1 hexane–EtOAc). The title compound was isolated as a colorless oil (6.5 mg, 0.020 mmol, 52%).

TLC: R_f = 0.34 (Hexane–EtOAc 10:1, $KMnO_4$/UV); ^{1}H-NMR (500 MHz, C_6D_6) δ 7.54 – 7.49 (m, 2H), 7.19 – 7.12 (m, 3H), 7.07 (tt, J = 7.5, 1.2 Hz, 1H), 6.97 – 6.91 (m, 2H), 6.70 (dddd, J = 7.5, 1.9, 1.2, 0.5 Hz, 1H), 3.27 (dt, J = 13.4, 1.2 Hz, 1H), 3.24 – 3.20 (m, 1H), 2.29 – 2.16 (m, 2H), 2.07 (ddd, J = 11.3, 9.1, 3.0 Hz, 1H), 1.91 (ddd, J = 11.3, 10.1, 9.3 Hz, 1H), 1.29 (s, 3H), 0.48 (s, 3H), 0.43 (s, 3H); ^{13}C-NMR (126 MHz, C_6D_6) δ 210.4, 146.1, 136.9, 134.9, 133.9, 129.8, 128.8, 128.2, 127.7, 127.4, 125.4, 53.1, 51.5, 47.0, 34.0, 29.1, 26.8, -3.2, -3.7; IR (thin film): 3069, 3022, 2954, 2869, 1689, 1486, 1453, 1427, 1273, 1250, 1235, 1110, 1038, 1004, 823, 762, 739, 704, 678, 603, 468, 415 cm^{-1}; HRMS (ESI): exact mass calculated for $C_{21}H_{24}NaOSi$ [(M+Na)$^+$] 343.1489, found 343.1489.

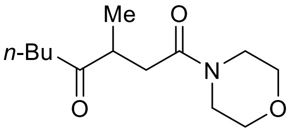

Morpholine amide **S2j**.

A 50 mL two-necked round bottom flask equipped with a magnetic stir bar, gas inlet and rubber septum was heated *in vacuo* and backfilled with Ar three times. $AlCl_3$ (2.1 g, 16 mmol, 1.3 equiv) and 1,2-dichloroethane (6.1 mL) were successively added and the suspension was cooled to 0 °C. Morpholine (2.7 mL, 30 mmol, 2.5 equiv) in 1,2-dichloroethane (3.7 mL) was added dropwise. The ice bath was removed and the thick suspension was stirred for 50 minutes. Then, whiskey lactone (2.0 mL, 12 mmol, 1.0 equiv) was added in one portion and the mixture was stirred for 19 hours. As the mixture initially became a thick slurry, it was shortly stirred with a spatula. The yellow suspension was poured into sat aq $NaHCO_3$ / sat aq Rochelle's salt solution (1:1). EtOAc was added and the biphasic mixture was stirred vigorously for 3 hours at r.t. The aqueous phase was extracted twice with EtOAc. The organic layers were combined and dried over $MgSO_4$. Concentration *in vacuo* afforded a yellow oil. The material was used in the next step without further purification.

A 500 mL one-necked pear shaped flask equipped with a magnetic stir bar was charged with the crude alcohol (2.9 g, 12 mmol, 1.0 equiv), CH_2Cl_2 (120 mL) and *t*-BuOH (1.1 mL, 11 mmol, 1.0 equiv). DMP (5.9 g, 13 mmol, 1.1 equiv) was added in one portion and the reaction mixture was stirred for 16 minutes at r.t. The suspension was poured into aq $Na_2S_2O_3$/$NaHCO_3$ solution (ca. 20 g $Na_2S_2O_3$ in 200 mL sat aq NaHCO3) and the biphasic mixture was stirred vigorously for 10 minutes. The aqueous phase was extracted twice with CH_2Cl_2. The organic layers were combined and dried over $MgSO_4$. Concentration *in vacuo* afforded a yellow oil with white particles. Flash column chromatography (eluent: 2:1 Hexane–EtOAc) furnished the title compound as a faint yellow oil (2.7 g, 11 mmol, 92% over 2 steps).

TLC: R_f = 0.30 (Hexane–EtOAc 1:2, $KMnO_4$); ^{1}H-NMR (500 MHz, $CDCl_3$) δ 3.71 – 3.40 (m, 8H), 3.12 (ddddd, *J* = 12.7, 10.3, 7.2, 4.2, 1.2 Hz, 1H), 2.88 – 2.79 (m, 1H), 2.57 (dddd, *J* = 8.9, 7.4, 2.8, 1.4 Hz, 2H), 2.19 (dddd, *J* = 16.3, 4.2, 2.6, 1.0 Hz, 1H), 1.56 (pdd, *J* = 7.9, 7.1, 2.9, 1.6 Hz, 2H), 1.34 – 1.25 (m, 2H), 1.09 (ddd, *J* = 7.3, 3.0, 1.2 Hz, 3H), 0.89 (tdd, *J* = 7.4, 3.0, 1.1 Hz, 3H); ^{13}C-NMR (126 MHz, $CDCl_3$) δ 214.2 (d, *J* = 1.4 Hz), 170.1, 66.8, 66.5, 45.8, 41.9, 41.8, 41.4, 36.2, 25.6, 22.4, 17.1, 13.9; IR (thin film): 2960, 2932, 2859, 1711, 1645, 1457, 1436, 1410, 1378, 1300, 1275, 1230, 1115, 1070, 1039, 969, 920, 851, 577 cm^{-1}; HRMS (ESI): exact mass calculated for $C_{13}H_{23}NNaO_3$ [$(M+Na)^+$] 264.1570, found 264.1570.

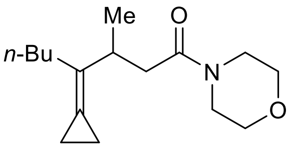

Morpholine amide **S3j**.

A 100 mL one-necked pear shaped flask equipped with a magnetic stir bar was charged with ketone **S2j** (0.50 g, 2.1 mmol, 1.0 equiv) under Ar atmosphere. $NaHCO_3$ (0.88 g, 10 mmol, 5.0 equiv), dry PhMe (21 mL) and $Cp_2Ti(C_3H_5)_2$ in PhMe (0.60 M, 7.7 mL, 4.6 mmol, 2.2 equiv) were successively added and the red/maroon mixture was heated to 50 °C and stirred for 2 hours. It was allowed to cool to r.t. Flash column chromatography (eluent: 1:50:150 Et_3N–Hexane–EtOAc) furnished the title compound as a yellow oil (0.097 g, 0.37 mmol, 17%).

TLC: R_f = 0.50 (Hexane–EtOAc 1:1, $KMnO_4$); ^{1}H-NMR (500 MHz, C_6D_6) δ 3.51 – 3.39 (m, 2H), 3.31 – 3.21 (m, 2H), 3.21 – 3.13 (m, 2H), 3.11 (dddt, *J* = 7.7, 6.9, 6.0, 0.9 Hz, 1H), 2.82 (t, *J* = 4.8 Hz, 2H), 2.45 (dd, *J* = 15.1, 6.1 Hz, 1H), 2.28 (ddt, *J* = 9.2, 7.1, 1.4 Hz, 2H), 2.13 (dd, *J* = 15.2, 8.0 Hz, 1H), 1.65 – 1.56 (m, 2H), 1.39 – 1.30 (m, 2H), 1.24 (d, *J* = 6.9 Hz, 3H), 1.02 – 0.92 (m, 2H), 0.95 (t, *J* = 7.4 Hz, 3H), 0.89 (dddd, *J* = 8.7, 6.3, 1.6, 1.0 Hz, 2H); ^{13}C-NMR (126 MHz, C_6D_6) δ 170.1, 133.0, 113.8, 67.0, 66.6, 45.9, 42.1, 39.1, 35.9, 34.7, 30.8, 23.2, 20.2, 14.3, 2.8, 0.9; IR (thin film): 2958, 2928, 2856, 1644, 1456, 1424, 1361, 1299, 1278, 1225, 1115, 1069, 1035, 1019, 967, 850, 574 cm^{-1}; HRMS (ESI): exact mass calculated for $C_{16}H_{27}NNaO_2$ [$(M+Na)^+$] 288.1934, found 288.1932.

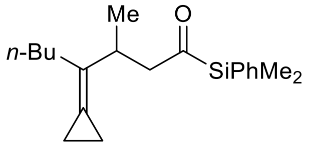

Acylsilane **1j**.

A 25 mL one-necked pear shaped flask equipped with a magnetic stir bar was charged with morpholine amide **S3j** (76 mg, 0.29 mmol, 1.0 equiv) under N_2 atmosphere. Dry THF (2.9 mL) was added and the solution was cooled to –78 °C. $PhMe_2SiLi$ in THF (1.49 M, 0.39 mL, 0.58 mmol, 2.0 equiv) was added dropwise and the dark red/brown mixture was stirred for 1 hour. Sat aq $NaHCO_3$ solution was added and the mixture was allowed to warm to r.t. The aqueous phase was extracted twice with EtOAc. The organic layers were combined and dried over $MgSO_4$. Concentration *in vacuo* afforded a yellow oil. Flash column chromatography (eluent: 1:1:200 Et_3N–Hexane–EtOAc) gave a faint yellow oil. The material was further purified by preparative TLC (TLC plate pretreated with Et_3N; eluent: 10:1 Hexane–EtOAc) to give the title compound as a colorless oil (46 mg, 0.15 mmol, 51%).

TLC: R_f = 0.63 (Hexane–EtOAc 10:1, $KMnO_4$/UV); ^{1}H-NMR (500 MHz, C_6D_6) δ 7.48 – 7.44 (m, 2H), 7.18 – 7.12 (m, 3H), 3.12 – 3.03 (m, 1H), 2.96 (dd, *J* = 16.7, 5.9 Hz, 1H), 2.60 (dd, *J* = 16.7, 7.8 Hz, 1H), 2.20 – 2.09 (m, 2H), 1.55 – 1.47 (m, 2H), 1.33 – 1.24 (m, 2H), 1.05 (d, *J* = 6.9 Hz, 3H), 0.92 (t, *J* = 7.4 Hz, 3H), 0.90 – 0.86 (m, 1H), 0.84 – 0.76 (m, 3H), 0.35 (s, 6H); ^{13}C-NMR (126 MHz, C_6D_6) δ 243.4, 135.2, 134.3, 132.6, 130.0, 128.4, 113.8, 55.0, 34.6, 33.2, 30.7, 23.1, 20.0, 14.3, 2.7, 0.8, -4.7, -4.7; IR (thin film): 3047, 2958, 2930, 2872, 1644, 1457, 1429, 1376, 1249, 1110, 999, 835, 815, 779, 734, 699, 650, 544, 466, 428 cm^{-1}; HRMS (ESI): exact mass calculated for $C_{20}H_{30}NaOSi$ [$(M+Na)^+$] 337.1958, found 337.1957.

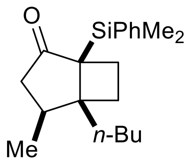

Cyclobutane **2j**.

The title compound was prepared according to general procedure #4 from acylsilane **1j** (28 mg, 0.089 mmol) with 40 mol% $BF_3{\cdot}OEt_2$. The reaction mixture was stirred at –78 °C to r.t. for 3 hours. The crude product was purified by flash column chromatography on silica gel (eluent: 100:1 hexane–EtOAc). The title compound was isolated as a faint yellow oil and as a single diastereomer (23 mg, 0.073 mmol, 82%).

TLC: R_f = 0.40 (Hexane–EtOAc 10:1, $KMnO_4$/UV); ^{1}H-NMR (500 MHz, C_6D_6) δ 7.59 – 7.55 (m, 2H), 7.21 – 7.17 (m, 3H), 2.33 (dd, *J* = 18.5, 10.0 Hz, 1H), 2.30 – 2.23 (m, 1H), 1.97 – 1.87 (m, 3H), 1.68 (tq, *J* = 9.8, 6.6 Hz, 1H), 1.49 – 1.41 (m, 2H), 1.37 – 1.31 (m, 1H), 1.13 – 1.00 (m, 3H), 0.98 – 0.88 (m, 1H), 0.82 (t, *J* = 7.1 Hz, 3H), 0.62 (d, *J* = 6.7 Hz, 3H), 0.45 (s, 3H), 0.44 (s, 3H); ^{13}C-NMR (101 MHz, C_6D_6) δ 221.8, 138.2, 134.8, 129.4, 128.3, 55.5, 54.1, 47.5, 38.3, 35.3, 27.7, 25.8, 25.8, 23.9, 15.5, 14.2, -3.2, -3.4; IR (thin film): 3070, 2955, 2932, 2872, 1709, 1458, 1428, 1408, 1378, 1249, 1169, 1107, 1082, 1017, 941, 838, 811, 773, 737, 702, 656, 545, 470, 418 cm^{-1}; HRMS (ESI): exact mass calculated for $C_{20}H_{30}NaOSi$ [$(M+Na)^+$] 337.1958, found 337.1967.

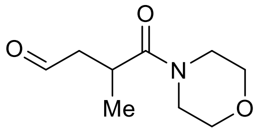

3-Methyl-4-morpholino-4-oxobutanal (**S5-3**).

A 50 mL two-necked round bottom flask equipped with a magnetic stir bar, gas inlet and rubber septum was heated *in vacuo* and backfilled with Ar three times. $AlCl_3$ (2.2 g, 16 mmol, 1.3 equiv) and 1,2-DCE (6.5 mL) were successively added and the suspension was cooled to 0 °C. Morpholine (2.8 mL, 32 mmol, 2.5 equiv) in 1,2-DCE (3.9 mL) was added dropwise. The ice bath was removed and the thick suspension was stirred for 35 minutes. Then, α-methyl-γ-butyrolactone (1.2 mL, 12 mmol, 1.0 equiv) was added in one portion and the mixture was stirred for 12 hours at r.t. The yellow suspension was poured into sat aq $NaHCO_3$/sat aq Rochelle's salt solution (1:1). EtOAc was added and the biphasic mixture was stirred vigorously for 30 minutes at r.t. The aqueous phase was extracted twice with EtOAc. The organic layers were combined and dried over $MgSO_4$. Concentration *in vacuo* afforded an orange oil. The material was used in the next step without further purification.
A 500 mL one-necked pear shaped flask equipped with a magnetic stir bar was charged with the crude alcohol in CH_2Cl_2 (83 mL). PCC (6.5 g, 30 mmol, 2.4 equiv) was added in one portion and the dark mixture was stirred vigorously for 1 hour. Celite (7 g) was added and the suspension was stirred for another 30 minutes. Et_2O was added and the mixture was filtered over Celite. The residue was washed throroughly with Et_2O. Concentration *in vacuo* afforded a brown liquid. Flash column chromatography (eluent: 1:3 Hexane–EtOAc) afforded the title compound as a pale yellow liquid (0.68 g, 3.7 mmol, 30% over 2 steps).

TLC: R_f = 0.42 (CH_2Cl_2–MeOH 10:1, $KMnO_4$); ^{1}H-NMR (500 MHz, $CDCl_3$) δ 9.75 (d, *J* = 1.6 Hz, 1H), 3.82 – 3.49 (m, 8H), 3.26 – 3.16 (m, 1H), 3.11 (ddd, *J* = 18.4, 8.7, 2.1 Hz, 1H), 2.53 – 2.45 (m, 1H), 1.15 – 1.12 (m, 3H); ^{13}C-NMR (126 MHz, $CDCl_3$) δ 200.9, 173.9, 67.0, 66.9, 48.0, 46.3, 42.5, 29.8, 17.7; IR (thin film): 2969, 2899, 2856, 2727, 1719, 1635, 1466, 1435, 1360, 1270, 1236, 1113, 1068, 1032, 943, 909, 846, 746, 615, 565 cm^{-1}; HRMS (ESI): exact mass calculated for $C_9H_{15}NNaO_3$ [$(M+Na)^+$] 208.0944, found 208.0963.

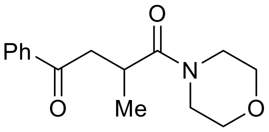

Morpholine amide **S2k**.

A 100 mL one-necked pear shaped flask equipped with a magnetic stir bar was charged with aldehyde **S5-3** (0.67 g, 3.6 mmol, 1.0 equiv) under Ar atmosphere. Dry THF (36 mL) was added and the colorless solution was cooled to –78 °C. PhMgBr in Et_2O (3.0 M, 1.6 mL, 5.7 mmol, 1.3 equiv) in dry THF (4.0 mL) was added over 5 minutes. The yellow solution was stirred for 80 minutes at –78 °C. Sat aq $NaHCO_3$ solution was added and the aqueous phase was extracted four times with EtOAc. The organic layers were combined and dried over $MgSO_4$. Concentration *in vacuo* afforded a cloudy, pale yellow oil, which was used in the subsequent reaction without further purification.
A 50 mL one-necked pear shaped flask equipped with a magnetic stir bar was charged with the crude alcohol in CH_2Cl_2 (18 mL). $NaHCO_3$ (0.61 g, 7.2 mmol, 2.0 equiv) and DMP (1.9 g, 4.3 mmol, 1.2 equiv) were successively added and the yellow suspension was stirred for 2 hours at r.t. A solution of $NaHCO_3$/$Na_2S_2O_3$ was added. The aqueous phase was extracted four times with EtOAc. The organic

layers were combined and dried over $MgSO_4$. Concentration *in vacuo* afforded a yellow oil. Flash column chromatography (Hexane–EtOAc, 1:1) furnished the title compound as a white, amorphous solid (0.54 g, 2.1 mmol, 57% over 2 steps).

TLC: R_f = 0.35 (Hexane–EtOAc 1:3, $KMnO_4$/UV); ^{1}H-NMR (500 MHz, $CDCl_3$) δ 7.99 – 7.96 (m, 2H), 7.55 (ddt, *J* = 8.6, 6.9, 1.3 Hz, 1H), 7.47 – 7.42 (m, 2H), 3.88 – 3.55 (m, 9H), 3.41 (dddd, *J* = 11.5, 8.7, 7.1, 4.5 Hz, 1H), 2.94 (dd, *J* = 17.9, 4.4 Hz, 1H), 1.21 (d, *J* = 7.1 Hz, 3H); ^{13}C-NMR (126 MHz, $CDCl_3$) δ 199.1, 174.6, 136.8, 133.3, 128.7, 128.2, 67.1, 67.1 – 66.9 (m), 46.4, 43.0, 42.4, 31.0, 18.0; IR (thin film): 2967, 2900, 2857, 1684, 1641, 1598, 1467, 1448, 1435, 1362, 1269, 1237, 1216, 1188, 1115, 1069, 1030, 1004, 886, 847, 763, 692, 577 cm^{-1}; HRMS (ESI): exact mass calculated for $C_{15}H_{19}NNaO_3$ [$(M+Na)^+$] 284.1257, found 284.1258.

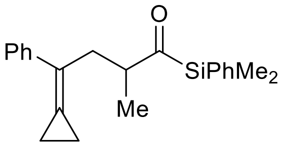

Acylsilane **1k**.

A 50 mL one-necked, pear-shaped flask equipped with a magnetic stir bar was charged with ketone **S2k** (0.31 g, 1.2 mmol, 1.0 equiv) under Ar atmosphere. Dry PhMe (11.8 mL), $NaHCO_3$ (0.50 g, 5.9 mmol, 5.0 equiv) and $Cp_2Ti(C_3H_5)_2$ in PhMe (0.60 M, 4.4 mL, 2.6 mmol, 2.2 equiv) were successively added and the red/maroon solution was heated to 50 °C and stirred for 25 minutes. The dark brown suspension was allowed to cool to r.t. Flash column chromatography (eluent: gradient from 1:18:180 to 1:50:150 Et_3N–hexane–EtOAc) furnished the intermediate morpholine amide **S3k** as a yellow oil (0.25 g). It contained impurities and the material was used as such in the next step.
A 25 mL one-necked, pear-shaped flask equipped with a magnetic stir bar was charged with crude morpholine amide **S3k** (76% of the material; 0.19 g, 0.66 mmol, 1.0 equiv) under Ar atmosphere. Dry THF (6.6 mL) was added and the yellow solution was cooled to –78 °C. $PhMe_2SiLi$ in THF (1.49 M, 0.88 mL, 1.3 mmol, 2.0 equiv) was added dropwise and the dark brown mixture was stirred for 20 minutes. Sat aq $NaHCO_3$ solution was quickly added and the aqueous phase was extracted twice with EtOAc. The organic layers were combined and dried over $MgSO_4$. Concentration *in vacuo* afforded a yellow oil. Flash column chromatography (eluent: 3:4:600 Et_3N–Hexane–EtOAc) furnished the title compound as a faint yellow oil (0,16 g, 0,47 mmol, 53% over 2 steps).

TLC: R_f = 0.48 (Hexane–EtOAc 10:1, $KMnO_4$/UV); ^{1}H-NMR (400 MHz, C_6D_6) δ 7.47 – 7.35 (m, 4H), 7.25 – 7.07 (m, 6H), 3.33 – 3.22 (m, 1H), 3.22 – 3.12 (m, 1H), 2.46 (ddt, *J* = 13.9, 8.9, 0.9 Hz, 1H), 1.16 – 1.07 (m, 1H), 1.07 – 0.98 (m, 1H), 0.88 (d, *J* = 6.8 Hz, 3H), 0.33 (s, 3H), 0.32 (s, 3H); ^{13}C-NMR (126 MHz, C_6D_6) δ 246.8, 140.1, 135.4, 134.4, 130.0, 128.6, 128.4, 126.9, 126.6, 125.6, 122.9, 49.0, 35.7, 14.7, 5.3, 1.7, -4.3, -4.4; IR (thin film): 3049, 2969, 1726, 1638, 1598, 1495, 1452, 1428, 1370, 1248, 1110, 1071, 1030, 984, 833, 814, 780, 735, 696, 651, 470, 433 cm^{-1}; HRMS (ESI): exact mass calculated for $C_{22}H_{27}OSi$ [$(M+H)^+$] 335.1826, found 335.1824.

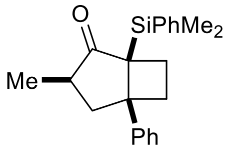

Cyclobutane **2k**.

The title compound was prepared according to general procedure #4 from acylsilane **1k** (48 mg, 0.14 mmol). The reaction mixture was stirred at –78 °C to –20 °C for 3 hours. The crude product was purified by flash column chromatography on silica gel (eluent: 160:1 hexane–EtOAc). The title compound was isolated as a faint yellow oil and as a single diastereomer (34 mg, 0.10 mmol, 72%).

TLC: R_f = 0.44 (Hexane–EtOAc 10:1, $KMnO_4$/UV); ^{1}H-NMR (400 MHz, C_6D_6) δ 7.53 – 7.47 (m, 2H), 7.17 (dd, *J* = 4.5, 3.3 Hz, 3H), 7.11 – 7.02 (m, 3H), 7.01 – 6.97 (m, 2H), 2.61 (dddt, *J* = 9.9, 7.2, 4.9, 3.6 Hz, 1H), 2.51 (dddd, *J* = 12.5, 10.4, 5.7, 1.1 Hz, 1H), 2.20 (dddd, *J* = 12.1, 10.4, 7.0, 0.7 Hz, 1H), 1.99 – 1.86 (m, 2H), 1.75 (ddd, *J* = 12.0, 9.4, 5.7 Hz, 1H), 1.62 (ddd, *J* = 13.5, 11.6, 1.0 Hz, 1H), 1.08 (d, *J* = 7.0 Hz, 3H), 0.29 (s, 3H), -0.00 (s, 3H); ^{13}C-NMR (101 MHz, C_6D_6) δ 222.2, 146.3, 137.3, 135.1, 128.9, 128.0, 127.3, 126.7, 126.0, 52.4, 50.4, 47.1, 41.7, 29.0, 24.5, 14.7, -3.8, -4.8; IR (thin film): 3053, 2960, 2870, 1709, 1600, 1498, 1446, 1428, 1305, 1247, 1167, 1110, 1026, 1002, 941, 836, 814, 762, 736, 700, 652, 473, 439 cm^{-1}; HRMS (ESI): exact mass calculated for $C_{22}H_{26}NaOSi$ [$(M+Na)^+$] 357.1645, found 357.1643.

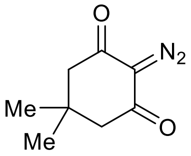

Diazodimedone (**S6**).

A 250 mL one-necked pear shaped flask equipped with a magnetic stir bar was charged with *p*-TsN_3[11] (19.1 g, 97.0 mmol, 1.00 equiv) and MeCN (56 mL). Dimedone (13.5 g, 96.0 mmol, 1.00 equiv) and K_2CO_3 (14.8 g, 107 mmol, 1.11 equiv) were successively added. The suspension was stirred vigorously for 12 hours at r.t. The brown reaction mixture was filtered over a short silica plug. The filtrate was concentrated *in vacuo* to afford a red/brown solid. Flash column chromatography (eluent: grade from 10:1 to 5:1 Hexane–EtOAc) furnished the title compound as a yellow solid (13.0 g, 78.0 mmol, 81%). The spectroscopic data matched those reported in the literature.[Error! Bookmark not defined.]

TLC: R_f = 0.29 (Hexane–EtOAc 3:1, Seebach's stain/UV); ^{1}H-NMR (400 MHz, $CDCl_3$) δ 2.44 (d, *J* = 0.7 Hz, 4H), 1.12 (s, 6H).

[11] Presset, M.; Mailhol, D.; Coquerel, Y.; Rodriguez, J. *Synthesis* **2011**, *16*, 2549–2552.

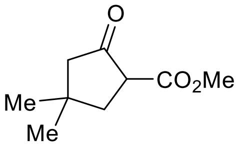

Methyl 4,4-dimethyl-2-oxocyclopentane-1-carboxylate (**S7**).

A 50 mL one-necked pear shaped flask equipped with a magnetic stir bar was charged with diazodimedone (**S6**) (2.0 g, 12 mmol, 1.0 equiv), dry PhMe (24 mL) and dry MeOH (0.97 mL, 24 mmol, 2.0 equiv) under N_2 atmosphere. The flask was equipped with a reflux condenser. The yellow solution was heated to 110 °C and stirred for 21 hours. The yellow/orange solution was allowed to cool to r.t. and concentrated *in vacuo*. Flash column chromatography (eluent: grade from 40:1 to 16:1 Pentane–Et_2O) furnished the title compound as a colorless liquid (1.75 g, 10.3 mmol, 85%). The spectroscopic data matched those reported in the literature.[12]

TLC: R_f = 0.54 (Hexane–EtOAc 3:1, Seebach's stain/UV); ^{1}H-NMR (400 MHz, $CDCl_3$) δ 3.74 (d, *J* = 0.6 Hz, 3H), 3.43 – 3.34 (m, 1H), 2.36 – 2.02 (m, 5H), 1.23 (s, 3H), 1.05 (s, 3H).

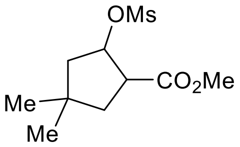

Methyl 4,4-dimethyl-2-((methylsulfonyl)oxy)cyclopentane-1-carboxylate (**S8**).

A 100 mL one-necked pear shaped flask equipped with a magnetic stir bar was charged with ketoester **S7** (1.75 g, 10.3 mmol, 1.00 equiv) and MeOH (41 mL). The colorless solution was cooled to 0 °C. $NaBH_4$ (0.778 g, 20.6 mmol, 2.00 equiv) was added in one portion. The mixture was stirred for 30 minutes. Sat aq NH_4Cl solution and Et_2O were added. The aqueous phase was extracted twice with Et_2O. The organic layers were washed with water, combined and dried over $MgSO_4$. Concentration *in vacuo* afforded a colorless oil.

A 250 mL one-necked round bottom flask equipped with a magnetic stir bar was charged with the intermediate alcohol under N_2 atmosphere. Dry CH_2Cl_2 (95 mL) and freshly distilled Et_3N (1.6 mL, 12 mmol, 1.2 equiv) were added and the colorless solution was cooled to 0 °C. MsCl (0.84 mL, 11 mmol, 1.1 equiv) was added dropwise and the mixture was stirred for 65 minutes. The ice bath was removed after 5 minutes. Sat aq $NaHCO_3$ solution and CH_2Cl_2 were added. The aqueous phase was extracted twice with CH_2Cl_2. The organic layers were combined and dried over $MgSO_4$. Concentration *in vacuo* afforded a colorless oil. Flash column chromatography (eluent: grade from 20:1 to 6:1) furnished the title compound as an inconsequential mixture of diastereomers (d.r. = 9:1, 1.83 g, 7.31 mmol, 82%).

TLC: R_f = 0.30 (Hexane–EtOAc 3:1, Seebach's stain); ^{1}H-NMR (400 MHz, $CDCl_3$, major diasteromer) δ 5.35 – 5.27 (m, 1H), 3.72 (s, 3H), 3.18 (td, *J* = 9.6, 6.4 Hz, 1H), 3.02 (s, 3H), 2.07 (dd, *J* = 14.0, 7.9 Hz, 1H), 1.96 (ddd, *J* = 13.1, 9.3, 1.4 Hz, 1H), 1.81 (ddd, *J* = 14.0, 5.6, 1.3 Hz, 1H), 1.63 (dd, *J* = 13.1, 9.9 Hz, 1H), 1.12 (s, 3H), 1.05 (s, 3H). ^{13}C-NMR (126 MHz, $CDCl_3$, major diasteromer) δ 174.0, 84.3, 52.3, 50.2, 47.2, 42.8, 38.3, 38.0, 29.5, 29.3. IR (thin film): 3024, 2956, 2871, 1732, 1464, 1437, 1354, 1265, 1172, 1017, 956, 904, 855, 819, 779, 525 cm^{-1}. HRMS (ESI): exact mass calculated for $C_{10}H_{22}NO_5S$ [$(M+NH_4)^+$] 268.1213, found 268.1215.

[12] Presset, M.; Coquerel, Y.; Rodriguez, J. *J. Org. Chem.* **2009**, *74*, 415–418.

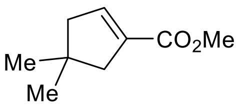

Methyl 4,4-dimethylcyclopent-1-ene-1-carboxylate (**S9**).

A 250 mL two-necked round bottom flask equipped with a magnetic stir bar was heated *in vacuo* and backfilled with N2 three times. Mesylate **S8** (1.83 g, 7.31 mmol, 1.00 equiv) in dry CH_2Cl_2 (73 mL) was added and the colorless solution was cooled to 0 °C. DBU (1.65 mL, 11.0 mmol, 1.50 equiv) was added dropwise and the solution was stirred for 95 minutes. The ice bath was removed after 5 minutes. 1M aq HCl solution (20 mL) and pentane were successively added and the aqueous phase was extracted twice with pentane. The organic layers were washed with brine, combined and dried over $MgSO_4$. Concentration *in vacuo* (p = 800 mbar, T = 42 °C) afforded a pale yellow liquid. Flash column chromatography (eluent: grade from 200:0 to 100:1 Pentane–Et_2O) furnished the title compound as a pale yellow oil (1.1 g, 7.1 mmol, 98%). The spectroscopic data matched those reported in the literature.[13]

TLC: R_f = 0.73 (Hexane–EtOAc 3:1, $KMnO_4$/UV); ^{1}H-NMR (500 MHz, $CDCl_3$) δ 6.68 – 6.64 (m, 1H), 3.72 (d, J = 0.7 Hz, 3H), 2.39 (qd, J = 2.3, 0.6 Hz, 2H), 2.30 (qd, J = 2.5, 0.6 Hz, 2H), 1.10 (d, J = 0.7 Hz, 6H). ^{13}C-NMR (126 MHz, $CDCl_3$) δ 166.0, 142.7, 135.1, 51.5, 48.4, 46.4, 38.9, 29.7.

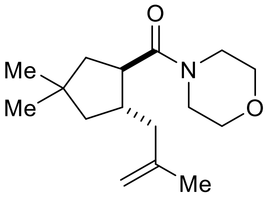

Morpholine amide **S10**.

A 25 mL Schlenk flask equipped with a magnetic stir bar was heated *in vacuo* and backfilled with N_2 three times. TBAF in THF (1.0 M, 1.4 mL, 1.4 mmol, 0.50 equiv) was added and the volatiles were removed *in vacuo*. The colorless oil was dried for 30 minutes under high vacuum. Dry DMF (34 mL) was added to give a faint yellow solution. A 250 mL two-necked round bottom flask equipped with a magnetic stir bar, rubber septum and gas inlet was charged with 4Å molecular sieves (10 g). The flask was heated *in vacuo* and backfilled with N_2 three times. The TBAF/DMF solution was added and the suspension was stirred for 30 minutes at r.t. Dry HMPA (2.4 mL, 14 mmol, 6.1 equiv) was added followed by dropwise addition of a mixture of enoate **S9** (0.35 mg, 2.3 mmol, 1.0 equiv) and methallyltrimethylsilane (2.3 mL, 14 mmol, 6.0 equiv) in dry DMF (22 mL) over 15 minutes. The resulting yellow supsension was stirred for 12 hours at r.t. MeOH and water (each ca 20 mL) were added and the mixture was filtered over Celite and the residue was washed thoroughly with Et_2O. The aqueous phase was extracted twice with Et_2O. The organic layers were washed twice with water. The organic layers were combined and dried over $MgSO_4$. Concentration *in vacuo* afforded a red/brown liquid. Flash column chromatography (eluent: grade from 200:0 to 100:1 Pentane–Et_2O) afforded a yellow liquid (0.30 g).

A 10 mL Schlenk flask equipped with a magnetic stir bar and rubber septum was heated *in vacuo* and backfilled with N2 three times. Morpholine (84 µL, 0.96 mmol, 4.1 equiv) and dry CH_2Cl_2 (1.8 mL) was

[13] Magnus, P.; Quagliato, D. *J. Org. Chem.* **1985**, *50*, 1621–1626.

added at r.t. $AlMe_3$ in hexane (2.0 M, 0.48 mL, 0.96 mmol, 4.0 equiv) was added dropwise and the colorless solution was stirred for 30 minutes at r.t. Then, the intermediate enamide (17% of the material; 50 mg, 0.24 mmol, 1.0 equiv) in dry CH_2Cl_2 (0.60 mL) was added dropwise and the yellow reaction mixture was stirred for 37 hours. The reaction mixture was heated to 34 °C after 19 hours. The mixture was allowed to cool to r.t. Sat aq $NaHCO_3$ solution and an aqueous solution of Rochelle's salt were successively added. The aqueous phase was extracted twice with EtOAc. The organic layers were combined and dried over $MgSO_4$. Concentration *in vacuo* afforded an intense yellow/orange oil. Flash column chromatography (eluent: grade from 20:1 to 6:1 Hexane–EtOAc) furnished the title compound as a yellow oil (25 mg, 0.094 mmol, 25% over 2 steps).

TLC: R_f = 0.50 (Hexane–EtOAc 1:1, $KMnO_4$); ^{1}H-NMR (500 MHz, $CDCl_3$) δ 4.65 – 4.61 (m, 2H), 3.71 – 3.51 (m, 6H), 3.46 (p, *J* = 4.0 Hz, 2H), 2.89 – 2.79 (m, 1H), 2.60 (q, *J* = 9.3 Hz, 1H), 2.10 (dd, *J* = 13.2, 6.2 Hz, 1H), 1.90 (ddd, *J* = 13.3, 8.6, 1.0 Hz, 1H), 1.74 – 1.67 (m, 2H), 1.70 – 1.69 (m, 3H), 1.50 (dd, *J* = 12.7, 9.4 Hz, 1H), 1.10 (dd, *J* = 12.7, 10.4 Hz, 1H), 1.04 (s, 3H), 1.03 (d, *J* = 0.8 Hz, 3H). ^{13}C-NMR (126 MHz, $CDCl_3$) δ 174.0, 145.5, 110.9, 67.2, 66.9, 47.4, 47.2, 46.2, 46.0, 44.6, 42.4, 40.5, 38.5, 30.8, 30.2, 22.3. IR (thin film): 3071, 2952, 2860, 1643, 1431, 1366, 1269, 1239, 1212, 1116, 1039, 980, 885, 573 cm^{-1}. HRMS (ESI): exact mass calculated for $C_{16}H_{27}NNaO_2$ [$(M+Na)^+$] 288.1934, found 288.1933.

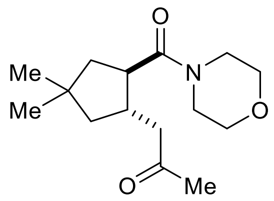

Morpholine amide **S2l**.

Olefin **S10** (0.10 g, 0.38 mmol, 1.0 equiv) was dissolved in CH_2Cl_2–MeOH (10:1, 15 mL) and cooled to –78°C. A mixture of ozone and oxygen was passed through the faint yellow solution until it turned blue. Pure oxygen was subsequently bubbled through the reaction mixture for 30 seconds (causing complete decoloration of the solution), followed by nitrogen. Dimethylsulfide (0.50 mL, 6.8 mmol, 18 equiv) was added and the reaction mixture was stirred for 20 hours. During this time, it was allowed to warm to r.t. Concentration *in vacuo* afforded a pale yellow, cloudy oil. Flash column chromatography (eluent: grade from 3:1 to 1:1 Hexane–EtOAc) furnished the title compound as a colorless oil (99 mg, 0.37 mmol, 96%).

TLC: R_f = 0.19 (Hexane–EtOAc 1:1, $KMnO_4$); ^{1}H-NMR (500 MHz, $CDCl_3$) δ 3.75 – 3.51 (m, 6H), 3.47 (dd, *J* = 16.2, 7.7 Hz, 2H), 3.07 – 2.95 (m, 1H), 2.69 (q, *J* = 9.5 Hz, 1H), 2.49 (dd, *J* = 14.5, 5.9 Hz, 1H), 2.32 (dd, *J* = 14.5, 8.2 Hz, 1H), 2.12 (d, *J* = 0.5 Hz, 3H), 1.82 (dd, *J* = 12.6, 7.6 Hz, 1H), 1.73 (dd, *J* = 12.8, 9.2 Hz, 1H), 1.51 – 1.43 (m, 1H), 1.14 (dd, *J* = 12.7, 11.0 Hz, 1H), 1.06 (s, 3H), 1.04 (s, 3H); ^{13}C-NMR (126 MHz, $CDCl_3$) δ 208.9, 173.2, 67.1, 66.9, 49.2, 47.1, 46.9, 46.1, 45.6, 42.4, 38.4, 38.4, 30.9, 30.3, 29.9; IR (thin film): 2953, 2929, 2863, 1710, 1639, 1433, 1360, 1300, 1269, 1243, 1217, 1116, 1070, 1039, 995, 582 cm^{-1}; HRMS (ESI): exact mass calculated for $C_{15}H_{25}NNaO_3$ [$(M+Na)^+$] 290.1727, found 290.1731.

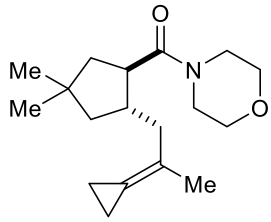

Morpholine amide **S3l**.

A 10 mL one-necked pear shaped flask equipped with a magnetic stir bar was charged with ketone **S2l** (21 mg, 0.080 mmol, 1.0 equiv) and $NaHCO_3$ (34 mg, 0.40 mmol, 5.0 equiv) under Ar atmosphere. Dry PhMe (0.80 mL) and $Cp_2Ti(C_3H_5)_2$ in PhMe (0.55 M, 0.36 mL, 0.20 mmol, 2.5 equiv) were successively added and the dark red/brown suspension was heated to 50 °C and stirred for 90 minutes. The reaction mixture was allowed to cool to r.t. It was directly purified by flash column chromatography (eluent: grade from 1:10:200 to 3:40:600 Et_3N–Hexane–EtOAc). The title compound was obtained as a yellow oil (13 mg, 0.045 mmol, 56%).

TLC: R_f = 0.55 (Hexane–EtOAc 1:1, $KMnO_4$); ^{1}H-NMR (400 MHz, C_6D_6) δ 3.56 – 3.30 (m, 3H), 3.23 (d, J = 16.2 Hz, 4H), 2.87 (s, 2H), 2.46 – 2.34 (m, 2H), 2.20 (dd, J = 13.0, 8.1 Hz, 1H), 1.99 (q, J = 1.6 Hz, 3H), 1.73 (dd, J = 12.4, 7.4 Hz, 1H), 1.59 – 1.43 (m, 2H), 1.15 (dd, J = 12.5, 10.9 Hz, 1H), 1.09 (s, 3H), 1.02 (s, 3H), 1.06 – 0.89 (m, 4H); ^{13}C-NMR (126 MHz, C_6D_6) δ 173.2, 124.7, 115.9, 67.1, 66.9, 48.3, 47.5, 45.9, 45.9, 43.5, 42.5, 40.9, 38.7, 30.8, 30.0, 20.9, 3.3, 2.5; IR (thin film): 2952, 2926, 2859, 1644, 1430, 1366, 1300, 1269, 1213, 1117, 1038, 993 cm^{-1}; HRMS (ESI): exact mass calculated for $C_{18}H_{30}NO_2$ [(M+H)$^+$] 292.2271, found 292.2272.

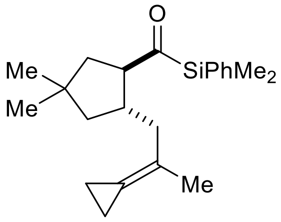

Acylsilane **1l**.

A 10 mL one-necked round bottom flask equipped with a magnetic stir bar was charged with morpholine amide **S3l** (40 mg, 0.14 mmol, 1.0 equiv) under Ar atmosphere. Dry THF (1.4 mL) was added and the yellow solution was cooled to –78 °C. $PhMe_2SiLi$ in THF (1.49 M, 0.28 mL, 0.42 mmol, 3.0 equiv) was added dropwise and the dark red/brown mixture was stirred for 50 minutes at –78 °C. Sat aq $NaHCO_3$ solution (2 mL) was added and the mixture was allowed to warm to r.t. EtOAc was added and the aqueous phase was extracted twice with EtOAc. The organic layers were combined and dried over MgSO4. Concentration *in vacuo* afforded a yellow oil. Flash column chromatography (Hexane–EtOAc +0.5% Et_3N, 200:1) afforded the title compound as a faint yellow oil (44 mg, 0.12 mmol, 93%).

TLC: R_f = 0.60 (Hexane–EtOAc 10:1, $KMnO_4$/UV); ^{1}H-NMR (500 MHz, $CDCl_3$) δ 7.53 – 7.48 (m, 2H), 7.18 – 7.16 (m, 2H), 7.16 – 7.16 (m, 1H), 3.19 (dp, J = 9.8, 7.8 Hz, 1H), 3.05 (dt, J = 10.3, 8.0 Hz, 1H), 2.22 – 2.08 (m, 2H), 1.85 (p, J = 1.7 Hz, 3H), 1.60 (ddd, J = 12.4, 7.8, 1.1 Hz, 1H), 1.52 (dd, J = 12.7, 10.3 Hz, 1H), 1.33 (ddd, J = 12.6, 7.6, 1.1 Hz, 1H), 1.09 (dd, J = 12.4, 9.8 Hz, 1H), 0.91 (s, 3H), 1.01 – 0.80 (m, 4H), 0.88 (s, 3H), 0.37 (s, 3H), 0.37 (s, 3H); ^{13}C-NMR (126 MHz, $CDCl_3$) δ 244.2, 135.5, 134.4, 130.0,

128.3, 124.5, 116.3, 63.1, 48.3, 43.8, 43.2, 39.2, 37.1, 30.2, 29.0, 20.6, 3.2, 2.4, -4.0, -4.2; IR (thin film): 3046, 2952, 2933, 2864, 1637, 1445, 1428, 1368, 1248, 1110, 998, 832, 780, 735, 701, 650, 539, 467, 424 cm^{-1}; HRMS (ESI): exact mass calculated for $C_{22}H_{32}NaOSi$ $[(M+Na)^+]$ 363.2115, found 363.2116.

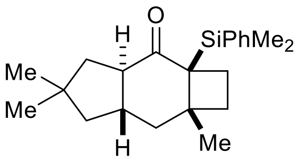

Cyclobutane **2l**.

The title compound was prepared according to general procedure #4 from acylsilane **1l** (11.1 mg, 33 µmol) using 40 mol% $BF_3{\cdot}OEt_2$. The reaction mixture was stirred at –78 °C to 0 °C for 2 hours. The crude product was purified by preparative thin layer chromatography (eluent: 20:1 hexane–EtOAc). The title compound was isolated as a colorless oil and as a mixture of diastereomers (d.r. = 3.0:1, 7.2 mg, 0.021 mmol, 65%).

TLC: R_f = 0.51 (Hexane–EtOAc 10:1, $KMnO_4$/UV); ^{1}H-NMR (500 MHz, C_6D_6) δ 7.69 – 7.64 (m, 2H), 7.44 – 7.41 (m, 2H, minor), 7.24 – 7.17 (m, 3H), 2.68 (ddd, J = 10.6, 8.9, 1.8 Hz, 1H, minor), 2.30 – 2.12 (m, 3H), 2.09 – 2.00 (m, 2H, minor), 1.96 – 1.80 (m, 2H, minor), 1.75 – 1.57 (m, 3H), 1.56 – 1.44 (m, 3H), 1.41 (dd, J = 13.2, 6.7 Hz, 1H, minor), 1.37 (dd, J = 12.2, 6.5 Hz, 1H, major), 1.29 (t, J = 12.8 Hz, 1H), 1.23 (d, J = 0.7 Hz, 3H, minor), 1.19 – 1.14 (m, 1H, minor), 1.10 (d, J = 0.5 Hz, 3H, major), 0.97 (s, 3H, minor), 0.95 (s, 3H, major), 0.89 (t, J = 11.8 Hz, 1H), 0.87 (s, 3H, minor), 0.84 (s, 3H, major), 0.57 (s, 3H, major), 0.55 (s, 3H, major), 0.36 (s, 1H, minor), 0.33 (s, 1H, minor); ^{13}C-NMR (126 MHz, C_6D_6), major diastereomer: δ 215.8, 139.6, 135.4, 129.2, 128.0, 55.6, 52.6, 48.0, 47.0, 45.7, 41.7, 40.9, 36.7, 35.9, 32.1, 31.7, 28.5, 26.8, -0.4, -1.5; minor diastereomer: 212.9, 137.7, 134.7, 129.5, 128.1, 57.3, 57.2, 51.7, 49.0, 44.4, 43.8, 39.5, 37.0, 32.9, 32.3, 31.7, 28.8, 25.8, -1.9, -3.8.; IR (thin film): 2950, 2865, 1678, 1461, 1427, 1364, 1249, 1173, 1108, 815, 779, 737, 703, 652, 544, 476, 421 cm^{-1}; HRMS (ESI): exact mass calculated for $C_{22}H_{32}NaOSi$ $[(M+Na)^+]$ 363.2115, found 363.2114.

Determination of relative stereochemistry via protodesilylation to ketone **S11**:

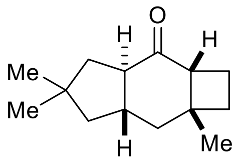

A 5 mL one-necked cone shaped flask equipped with a magnetic stir bar was charged with α-silyl ketone **2l** (6.9 mg, 0.020 mmol, 1.0 equiv) under N_2 atmosphere. Dry MeOH (0.41 mL) and KF (12 mg, 0.20 mmol, 10 equiv) were successively added and the reaction mixture was stirred for 24 hours at r.t. The volatiles were removed under a stream of N_2. Hexane was added and the suspension was filtered and concentrated *in vacuo*. Flash column chromatography (Pentane–Et_2O, 100:1) allowed isolation of the major diasteromer as a colorless oil (2.4 mg, 0.012 mmol, 57%). The spectroscopic data matched those reported in the literature.[14]

^{1}H-NMR (600 MHz, $CDCl_3$) δ 2.96 – 2.88 (m, 1H), 2.80 (dddd, J = 11.6, 9.3, 8.3, 0.9 Hz, 1H), 2.61 (ddq, J = 9.6, 7.2, 1.2 Hz, 1H), 2.23 – 2.10 (m, 2H), 1.89 – 1.77 (m, 2H), 1.68 – 1.62 (m, 3H), 1.46 (ddd, J = 13.2, 8.4, 2.2 Hz, 1H), 1.34 (d, J = 0.6 Hz, 3H), 1.22 (t, J = 13.1 Hz, 1H), 1.07 (s, 3H), 1.06 – 1.03 (m, 1H), 0.94 (d, J = 0.8 Hz, 3H); ^{13}C-NMR (151 MHz, $CDCl_3$) δ 218.4, 50.8, 48.7, 48.5, 44.0, 41.4, 40.3, 39.3, 38.5, 32.8, 29.0, 27.5, 27.0, 17.8; IR (thin film): 2951, 2932, 2866, 1706, 1463, 1376, 1367, 1324, 1280, 1225,

[14] Moens, L.; Baizer, M. M.; Little, R. D. *J. Org. Chem.* **1986**, *51*, 4497–4498.

1177, 1119, 1062, 938, 806 cm^{-1}; HRMS (ESI): exact mass calculated for $C_{14}H_{23}O$ [$(M+H)^+$] 207.1743, found 207.1745.

3. Lewis Acid Screening for Cycloisomerization of **1l**

The effect of various Lewis acids on the diastereoselectivity for cycloisomerization of acylsilane **1l** was investigated (Table 1).

Table 1. Condition screening for cycloisomerization of **1l**.

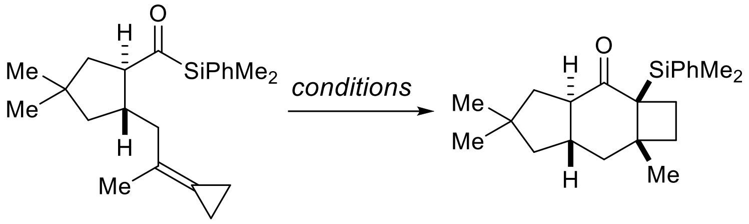

1l **2l**

Entry	Conditions	*dr*[a]	Yield[b]
1	$BF_3 \cdot OEt_2$ (40 mol%), CH_2Cl_2, –78 °C	3.0 : 1	65%
2	MAD[c] (120 mol%), PhMe, –78 °C to r.t.	–	–[d]
3	TMSOTf (40 mol%), CH_2Cl_2, –78 °C	5.4 : 1	73%
4	$EtAlCl_2$ (80 mol%), CH_2Cl_2, –78 to –30 °C	4.2 : 1	33%
5	$In(OTf)_3$, CH_2Cl_2, –78 to 10 °C	1.7 : 1	62%

[a]The diastereomeric ratio was determined by peak integration of the ^1H NMR spectrum of the crude material. [b]Yields were obtained after preparative thin layer chromatography on silica gel. [c]MAD = bis(2,6-di-tert-butyl-4-methylphenoxy)(methyl)aluminum. [d]No conversion of the starting material.

4. Product Functionalization

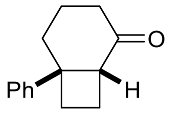

Cyclohexanone **17**.

To a solution of α-silyl cyclohexanone **2b** (9.0 mg, 27 µmol) in MeOH (0.27 mL) was added KF (7.8 mg, 0.14 mmol, 5 equiv). The reaction mixture was stirred at room temperature for 1 hour. Column chromatography of the crude reaction mixture yielded cyclohexanone (4.5 mg, 22 µmol, 84%) as a colorless oil.

<u>TLC</u>: R_f = 0.52 (20% EtOAc/hexane, Seebach stain); <u>^{1}H-NMR</u> (500 MHz, C_6D_6): δ 7.13 – 7.08 (m, 2H), 7.04 – 6.99 (m, 1H), 6.88 (dd, *J* = 8.3, 1.3 Hz, 2H), 3.16 (t, *J* = 8.9 Hz, 1H), 2.18 – 2.12 (m, 2H), 1.96 – 1.81 (m, 3H), 1.80 – 1.74 (m, 1H), 1.61 (ddd, *J* = 13.7, 9.9, 4.0 Hz, 1H), 1.49 – 1.43 (m, 1H), 1.40 – 1.33 (m, 2H) ppm; <u>^{13}C-NMR</u> (126 MHz, C_6D_6): δ 210.2, 150.3, 128.6, 126.0, 125.4, 50.1, 48.2, 38.9, 37.1, 32.3, 21.3, 20.8 ppm; <u>IR</u>: ν 2935, 1698, 1494, 760, 701 cm^{-1}; <u>HRMS</u> (ESI): Exact mass calculated for $C_{14}H_{16}NaO^+$ [$(M+Na)^+$] 223.1093, found 223.1097.

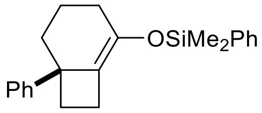

Silyl enol ether **18**.

A solution of α-silyl ketone **2b** (9.6 mg, 0.29 mmol) in d^8-toluene (0.4 mL) was heated at 90 °C for 15 hours. The reaction mixture was allowed to cool back to room temperature. Concentration *in vacuo* yielded an 87:13 mixture (9.6 mg, 0.29 mmol) of silyl enol ether **18** (87%) and recovered starting material (13%) as a pale yellow oil.

<u>TLC</u>: R_f = 0.83 (10% EtOAc/hexane, Seebach stain); <u>^{1}H-NMR</u> (500 MHz, C_6D_6): δ 7.70 – 7.65 (m, 2H), 7.32 – 7.28 (m, 2H), 7.27 – 7.21 (m, 4H), 7.18 – 7.15 (m, 1H), 7.13 – 7.09 (m, 1H), 2.60 – 2.54 (m, 1H), 2.53 – 2.44 (m, 1H), 2.18 – 1.96 (m, 4H), 1.75 (dt, *J* = 11.9, 3.2 Hz, 1H), 1.48 (ddd, *J* = 13.2, 11.9, 4.0 Hz, 1H), 1.37 – 1.27 (m, 2H), 0.46 (s, 3H), 0.46 (s, 3H) ppm; <u>^{13}C-NMR</u> (126 MHz, C_6D_6): δ 148.3, 142.3, 138.3, 133.9, 130.0, 128.4, 128.2, 128.1, 126.1, 118.9, 54.1, 37.5, 37.4, 29.9, 28.6, 20.1, -0.6, -0.6 ppm; <u>IR</u>: ν 2932, 1345, 1251, 1187, 960, 828, 620 cm^{-1}; <u>HRMS</u> (ESI): Exact mass calculated for $C_{14}H_{16}NaO^+$ [$(M+Na)^+$] 357.1645, found 357.1647.

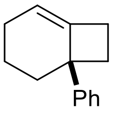

Olefin **19**.

A 5 mL one-necked cone shaped flask equipped with a magnetic stir bar was charged with α-silyl ketone **2b** (44 mg, 0.13 mmol, 1.0 equiv) under N_2 atmosphere. Dry PhMe (2.65 mL) was added and the colorless solution was cooled to –78 °C. DIBAL-H in PhMe (1.0 M, 0.27 mL, 0.27 mmol, 2.0 equiv)

was added and the reaction mixture was stirred for 40 minutes. Sat aq solution of Rochelle's salt was added. The aqueous phase was extracted twice with EtOAc. The organic layers were combined and dried over $MgSO_4$. Concentration *in vacuo* afforded a colorless oil. The material was used in the next step without further purification.

A 25 mL one-necked pear shaped flask equipped with a magnetic stir bar was charged with the crude alcohol under N_2 atmosphere. Dry CH_2Cl_2 (2.65 mL) was added and the colorless solution was cooled to 0 °C. $BF_3{\cdot}OEt_2$ (22 µL, 0.17 mmol, 1.3 equiv) was added dropwise and the reaction mixture was stirred for 15 minutes. Sat aq $NaHCO_3$ solution was added and the aqueous phase was extracted twice with pentane. The organic layers were combined and dried over $MgSO_4$. Concentration *in vacuo* afforded a pale yellow oil. Flash column chromatography (eluent: 1:200 Et_3N–hexane) afforded the desired product as a colorless oil with minor impurities (15 mg, 0.080 mmol, 60%).

TLC: R_f = 0.67 (Hexane, $KMnO_4$/UV); ^{1}H-NMR (500 MHz, C_6D_6) δ 7.39 – 7.35 (m, 2H), 7.25 – 7.21 (m, 2H), 7.13 – 7.08 (m, 1H), 5.50 – 5.47 (m, 1H), 2.80 – 2.69 (m, 1H), 2.42 (dddt, *J* = 14.0, 9.0, 2.3, 1.2 Hz, 1H), 2.13 (td, *J* = 9.3, 2.1 Hz, 1H), 2.10 – 2.01 (m, 2H), 1.94 (dddt, *J* = 11.4, 7.2, 6.4, 2.7 Hz, 1H), 1.87 (dt, *J* = 11.8, 3.3 Hz, 1H), 1.51 (td, *J* = 12.1, 4.2 Hz, 1H), 1.38 – 1.28 (m, 2H); ^{13}C-NMR (126 MHz, C_6D_6) δ 147.7, 142.2, 128.1, 128.0, 125.8, 115.5, 37.0, 36.6, 31.1, 24.6, 17.9; IR (thin film): 3057, 3022, 2932, 2838, 1601, 1493, 1445, 1077, 1030, 948, 902, 787, 758, 702, 559 cm^{-1}; HRMS (EI): exact mass calculated for $C_{14}H_{16}$ [$M^{\cdot+}$] 184.1247, found 184.1244.

5. Synthesis of Crystalline Derivative **13**

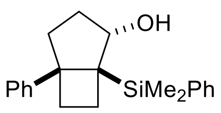

Cyclobutane **S12**.

To a solution of cyclopentanone **2a** (26.0 mg, 0.081 mmol) in CH_2Cl_2 (0.81 mL) at –78 °C was added dropwise a solution of DIBAL–H in hexane (0.12 mL, 1.0 M, 0.12 mmol, 1.5 equiv). The reaction mixture was stirred at this temperature for 1 hour before it was quenched by addition of sat. aq. $NaHCO_3$ and allowed to warm to room temperature. The organic layer was separated and the aqueous layer was extracted four times with ethyl acetate. The combined organic layers were dried ($MgSO_4$), filtered, and concentrated *in vacuo* to give cyclopentanol **S12** (22.1 mg, 0.069 mmol, 84%) as a colorless oil.

TLC: R_f = 0.30 (10% EtOAc/hexane, Seebach stain); ^{1}H-NMR (500 MHz, C_6D_6): δ 7.48 – 7.45 (m, 2H), 7.23 – 7.19 (m, 3H), 7.11 – 7.07 (m, 2H), 7.06 – 7.02 (m, 1H), 6.89 – 6.86 (m, 2H), 4.45 (dt, *J* = 10.1, 5.9 Hz, 1H), 2.66 – 2.59 (m, 1H), 2.02 (ddd, *J* = 13.6, 10.4, 8.0 Hz, 1H), 1.97 – 1.90 (m, 1H), 1.90 – 1.83 (m, 1H), 1.80 – 1.72 (m, 2H), 1.67 – 1.56 (m, 2H), 0.86 (d, *J* = 5.8 Hz, 1H), 0.20 (s, 3H), 0.03 (s, 3H) ppm; ^{13}C-NMR (126 MHz, C_6D_6) δ 149.4, 138.4, 135.3, 129.1, 128.5, 127.8, 127.1, 125.8, 78.3, 54.9, 46.4, 42.8, 33.7, 29.1, 16.8, -3.3, -5.0 ppm; IR: ν 3447, 2952, 1249, 1109, 810, 702 cm^{-1}; HRMS (ESI): Exact mass calculated for $C_{21}H_{26}NaOSi^+$ $[(M+Na)^+]$ 345.1645, found 345.1647.

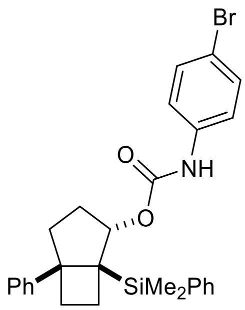

Cyclobutane **13**.

To a solution of alcohol **S12** (5.0 mg, 16 µmol) in CH_2Cl_2 (0.16 mL) were added Et_3N (6.5 µL, 47 µmol, 3 equiv) and 4-bromophenyl isocyanate (9.2 mg, 47 µmol, 3 equiv). The reaction mixture was stirred at room temperature for 2 hours and then at 37 °C for 2 h before it was quenched by addition of sat. aq. $NaHCO_3$. The aqueous layer was extracted four times with EtOAc and the combined organic layers were dried over $MgSO_4$, filtered, and concentrated *in vacuo*. Column chromatography (eluent: grade from 1:4:195 to 1:100:100 NEt_3/hexane/EtOAc) yielded carbamate **13** (5.5 mg, 11 µmol, 68%) as a colorless crystalline solid. Crystals amenable for X-Ray analysis were obtained by slow evaporation from ethyl acetate.

TLC: R_f = 0.36 (10% EtOAc/hexane, Seebach stain); ^{1}H-NMR (500 MHz, CD_2Cl_2): δ 7.45 – 7.41 (m, 2H), 7.34 – 7.15 (m, 10H), 7.09 – 7.03 (m, 2H), 6.34 (s, 1H), 5.53 (dd, *J* = 9.9, 6.4 Hz, 1H), 2.85 – 2.76 (m, 1H), 2.45 (dtd, *J* = 12.5, 6.0, 2.5 Hz, 1H), 2.25 (tdd, *J* = 12.0, 10.4, 9.2 Hz, 1H), 2.19 – 2.10 (m, 1H), 2.04 – 1.97 (m, 2H), 1.96 – 1.90 (m, 2H), 0.11 (s, 3H), 0.01 (s, 3H) ppm; ^{13}C-NMR (126 MHz, CD_2Cl_2) δ 153.4, 148.8, 138.4, 138.0, 135.2, 132.4, 129.1, 128.5, 127.7, 127.4, 126.3, 120.6, 115.9, 80.9, 54.5, 45.3,

43.1, 31.0, 29.1, 18.1, -4.0, -4.5 ppm; IR: ν 3329, 2960, 2927, 2281, 1704, 1518, 1219, 1054, 810, 702 cm^{-1}; HRMS (ESI): Exact mass calculated for $C_{28}H_{30}BrNNaO_2Si^+$ [$(M+Na)^+$] 542.1121, found 542.1121.

6. Control Experiments

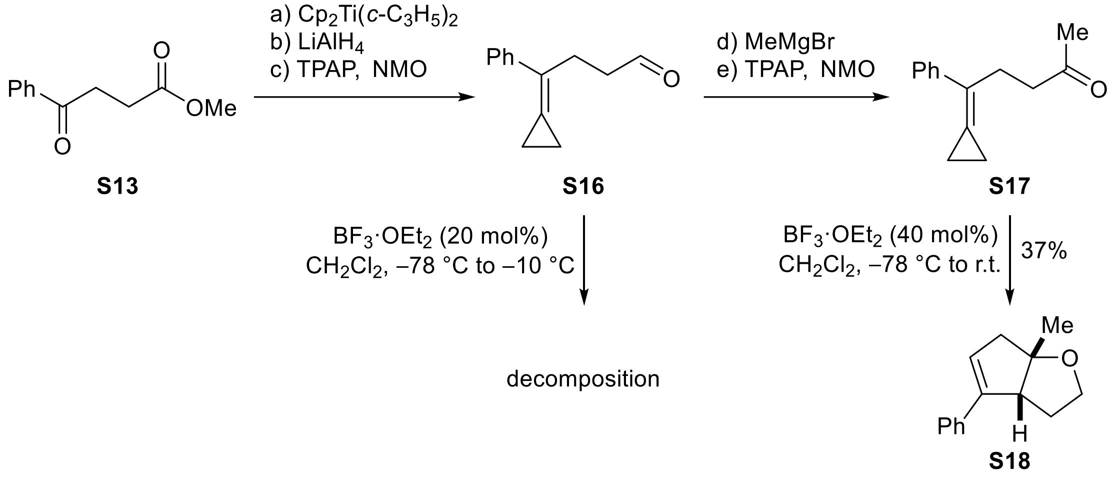

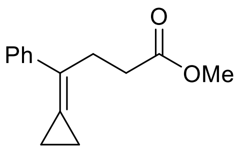

Cyclopropylidene **S14**.

To a solution of commercially available methyl 4-oxo-4-phenylbutanoate (**S13**; 1.0 g, 5.20 mmol) in PhMe (52 mL) was added a solution of dicyclopropane titanocene (prepared according to reference 2) in toluene (0.55 M, 1.3 equiv). The reaction mixture was heated at 50°C for 3 hours before it was allowed to cool back to room temperature and filtered through silica gel (eluent: grade from 1:20:180 to 1:200:0 NEt_3–EtOAc–hexane). Concentration yielded alkylidenecyclopropane **S14** (533 mg, 2.46 mmol, 47%) as a colorless oil.

TLC: R_f = 0.69 (30% EtOAc/hexane, Seebach stain); ^{1}H-NMR (500 MHz, $CDCl_3$): δ 7.60 – 7.57 (m, 2H), 7.37 – 7.31 (m, 2H), 7.25 – 7.21 (m, 1H), 3.66 (s, 3H), 2.99 (ddp, *J* = 9.3, 6.5, 1.4 Hz, 2H), 2.64 – 2.57 (m, 2H), 1.41 – 1.35 (m, 2H), 1.23 – 1.16 (m, 2H) ppm; ^{13}C-NMR (126 MHz, $CDCl_3$): δ 174.1, 139.7, 128.4, 126.9, 126.0, 125.8, 121.6, 51.7, 33.2, 29.3, 4.6, 1.6 ppm; IR: ν 2974, 1737, 1437, 1167, 696 cm^{-1}; HRMS (ESI): Exact mass calculated for $C_{14}H_{16}NaO_2^+$ [$(M+Na)^+$] 239.1043, found 239.1042.

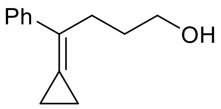

Alcohol **S15**.

To a solution of methyl ester **S14** (100 mg, 0.462 mmol) in THF (9.3 mL) at 0 °C was added dropwise a solution of $LiAlH_4$ in THF (0.19 mL, 2.4 M, 0.46 mmol, 1 equiv). The reaction mixture was stirred at 0 °C for 1 h before it was quenched by dropwise addition of H_2O (0.05 mL), 10% aq. NaOH (0.05 mL), and H_2O (0.05 mL). To the reaction mixture was added $MgSO_4$ and the reaction mixture was stirred

vigorously at room temperature for 30 minutes. Filtration and concentration of the filtrate *in vacuo* yielded alcohol **S15** (85 mg, 0.45 mmol, 98%) as a colorless oil.

TLC: R_f = 0.39 (30% EtOAc/hexane, Seebach stain); ^{1}H-NMR (400 MHz, $CDCl_3$): δ 7.63 – 7.57 (m, 2H), 7.34 (dd, *J* = 8.4, 7.0 Hz, 2H), 7.22 (tt, *J* = 6.9, 1.2 Hz, 1H), 3.67 (t, *J* = 6.6 Hz, 2H), 2.76 (ddt, *J* = 7.6, 6.4, 1.3 Hz, 2H), 1.90 – 1.78 (m, 2H), 1.57 (s, 1H), 1.41 (ddt, *J* = 7.7, 5.1, 1.4 Hz, 2H), 1.27 (s, 1H), 1.21 – 1.12 (m, 2H) ppm; ^{13}C-NMR (101 MHz, $CDCl_3$): δ 140.0, 128.4, 126.8, 126.7, 126.1, 121.2, 62.9, 31.5, 30.2, 5.0, 1.4 ppm; IR: ν 3339, 2944, 1495, 1060, 757, 695 cm^{-1}; HRMS (ESI): not found.

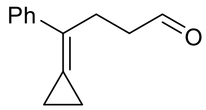

Cyclopropylidene **S16**.

To a solution of alcohol **S15** (85 mg, 0.45 mmol) in CH_2Cl_2 (4.5 mL) over crushed 4 Å molecular sieves (33 mg) were added NMO (79 mg, 0.68 mmol, 1.5 equiv) and TPAP (16 mg, 45 µmol, 10 mol%). The resulting black reaction mixture was stirred at room temperature for 1 h. Column chromatography of the crude reaction mixture (eluent: grade from 1:10:190 to 1:40:160 NEt_3/hexane/EtOAc) yielded aldehyde **S16** (78 mg, 0.42 mmol, 93%) as a colorless oil.

TLC: R_f = 0.72 (30% EtOAc/hexane, Seebach stain); ^{1}H-NMR (500 MHz, $CDCl_3$): δ 9.81 (t, *J* = 1.6 Hz, 1H), 7.60 – 7.55 (m, 2H), 7.39 – 7.32 (m, 2H), 7.26 – 7.22 (m, 1H), 3.01 (ddt, *J* = 7.8, 6.5, 1.4 Hz, 2H), 2.72 (tdd, *J* = 7.5, 1.7, 0.5 Hz, 2H), 1.39 (ddt, *J* = 9.0, 5.1, 1.5 Hz, 2H), 1.23 – 1.16 (m, 2H) ppm; ^{13}C-NMR (126 MHz, $CDCl_3$): δ 202.6, 139.6, 128.5, 127.0, 126.0, 125.5, 121.8, 42.6, 26.6, 4.7, 1.7 ppm; IR: ν 2925, 1722, 1495, 761, 694 cm^{-1}; HRMS (ESI): Exact mass calculated for $C_{13}H_{14}NaO^+$ [$(M+Na)^+$] 209.0937, found 209.0940.

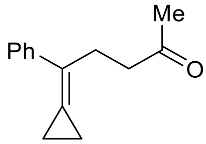

Cyclopropylidene **S17**.

To a solution of aldehyde **S16** (25 mg, 0.13 mmol) in diethyl ether (1.3 mL) at 0 °C was added dropwise a solution of MeMgBr in diethyl ether (54 µL, 3.0 M, 0.16 mmol, 1.2 equiv). The reaction mixture was stirred at this temperature for 1 hour before it was quenched by dropwise addition of sat. aq. $NaHCO_3$. The aqueous layer was extracted four times with ethyl acetate and the combined organic layers were dried ($MgSO_4$), filtered, and concentrated *in vacuo*. The resulting crude secondary alcohol was used in the next step without further purification.

To a solution of the crude secondary alcohol thus obtained in CH_2Cl_2 (1.3 mL) over crushed 4 Å molecular sieves (15 mg) were added NMO (23 mg, 0.20 mmol, 1.5 equiv) and TPAP (4.7 mg, 13 µmol, 10 mol%). The resulting black reaction mixture was stirred at room temperature for 1 h. Column chromatography of the crude reaction mixture (eluent: grade from 1:6:194 to 1:60:140 NEt_3/hexane/EtOAc) yielded ketone **S17** (19 mg, 95 µmol, 73% over 2 steps) as a pale yellow oil.

TLC: R_f = 0.67 (30% EtOAc/hexane, Seebach stain); ^{1}H-NMR (500 MHz, $CDCl_3$): δ 7.59 – 7.55 (m, 2H), 7.37 – 7.32 (m, 2H), 7.25 – 7.21 (m, 1H), 2.97 – 2.91 (m, 2H), 2.72 – 2.66 (m, 2H), 2.14 (d, *J* = 0.4 Hz, 3H), 1.40 – 1.35 (m, 2H), 1.19 – 1.14 (m, 2H) ppm; ^{13}C-NMR (126 MHz, $CDCl_3$): δ 208.8, 139.8, 128.5, 126.9, 126.0, 126.0, 121.4, 42.5, 30.1, 28.1, 4.7, 1.5 ppm; IR: ν 2971, 2926, 1717, 1496, 1359, 1161, 765, 696 cm^{-1}; HRMS (ESI): Exact mass calculated for $C_{14}H_{16}NaO^+$ $[(M+Na)^+]$ 223.1093, found 223.1097.

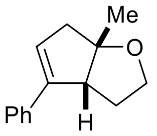

Cyclopentene **S18**.

To a solution of ketone **S17** (3.0 mg, 15 µmol) in CH_2Cl_2 (0.1 mL) at –78 °C was added $BF_3·OEt_2$ in CH_2Cl_2 (10.7 µL, 0.28 M, 3.0 µmol, 20 mol%) from a freshly prepared stock solution (the stock solution was prepared by dissolving $BF_3·OEt_2$ (16 µL, 0.13 mmol) in CH_2Cl_2 (0.46 mL). The reaction mixture was stirred at the indicated temperature for the indicated time before it was quenched by addition of sat. aq. $NaHCO_3$ and allowed to warm to room temperature. The aqueous layer was extracted four times with CH_2Cl_2 and the combined organic layers were dried ($MgSO_4$), filtered, and concentrated *in vacuo*. Column chromatography yielded cyclopentene **S18** (1.1 mg, 5.5 µmol, 20 mol%) as a colorless oil.

TLC: R_f = 0.55 (15% EtOAc/hexane, Seebach stain); ^{1}H-NMR (500 MHz, $CDCl_3$): δ 7.44 – 7.41 (m, 2H), 7.36 – 7.30 (m, 2H), 7.26 – 7.21 (m, 1H), 6.07 (td, *J* = 2.7, 1.6 Hz, 1H), 3.90 (ddd, *J* = 8.7, 7.2, 3.6 Hz, 1H), 3.72 – 3.65 (m, 1H), 3.32 (d, *J* = 9.2 Hz, 1H), 2.72 (dt, *J* = 18.6, 2.5 Hz, 1H), 2.61 (dd, *J* = 18.6, 2.3 Hz, 1H), 2.24 – 2.16 (m, 1H), 1.82 – 1.76 (m, 1H), 1.47 (s, 3H) ppm; ^{13}C-NMR (126 MHz, $CDCl_3$)[15]: δ 135.8, 128.6, 127.3, 126.2, 124.7, 90.0, 67.1, 55.9, 47.0, 32.0, 26.6 ppm; IR: ν 2961, 2925, 1261, 1044, 754, 693 cm^{-1}; HRMS (EI): Exact mass calculated for $C_{14}H_{16}O^+$ $[M^+]$ 200.1196, found 200.1196.

[15] One of the two all-carbon substituted C(sp^2)-C atoms could not be assigned due to its weak intensity.

7. NMR Spectra

^{1}H NMR (400 MHz, $CDCl_3$) of **S5-1**:

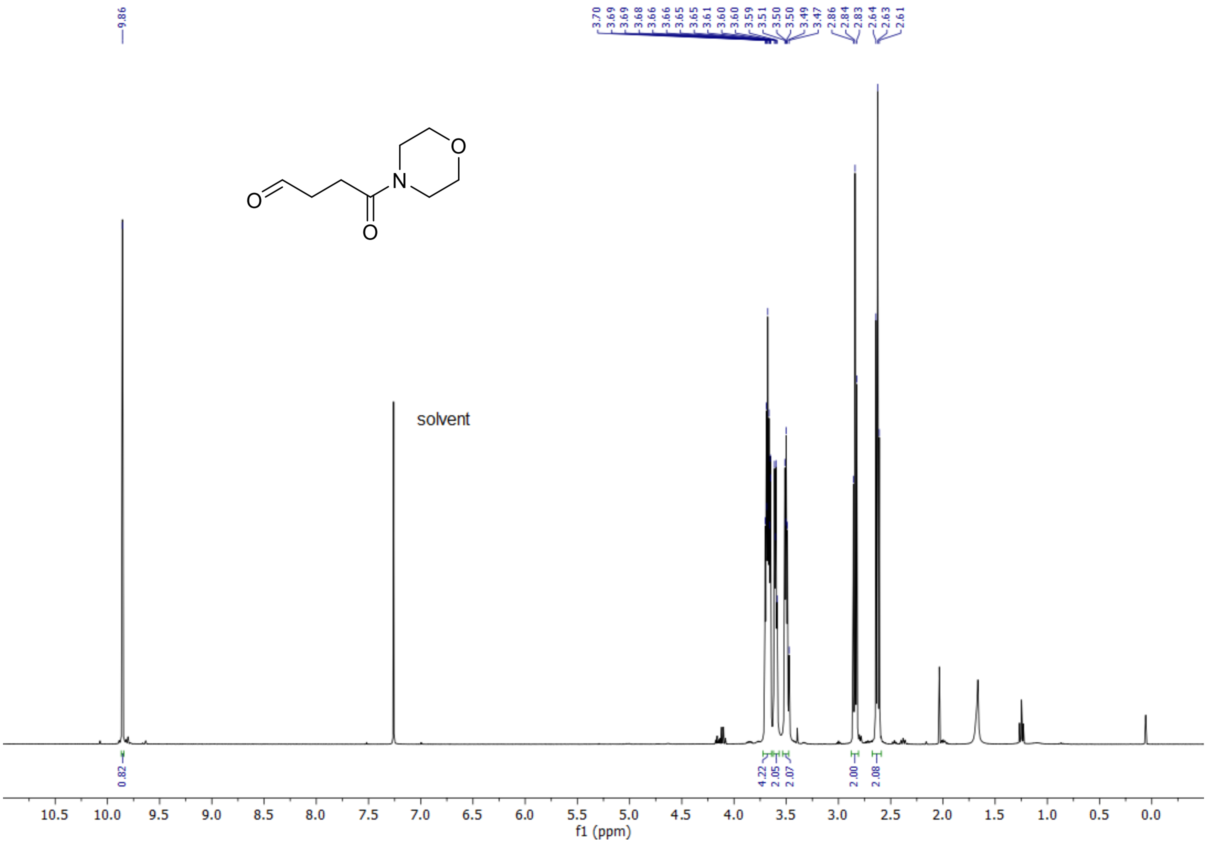

^{13}C NMR (101 MHz, $CDCl_3$) of **S5-1**:

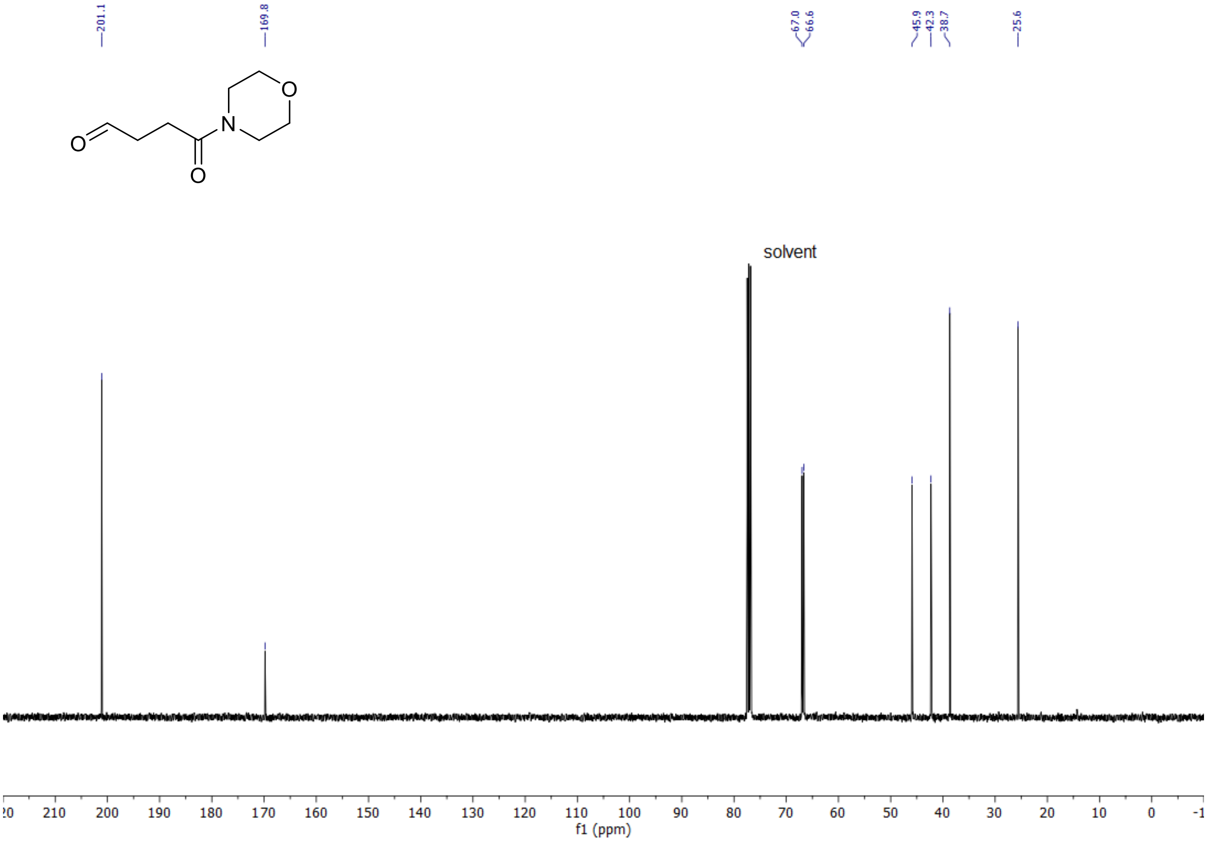

^{1}H NMR (400 MHz, $CDCl_3$) of **S5-2**:

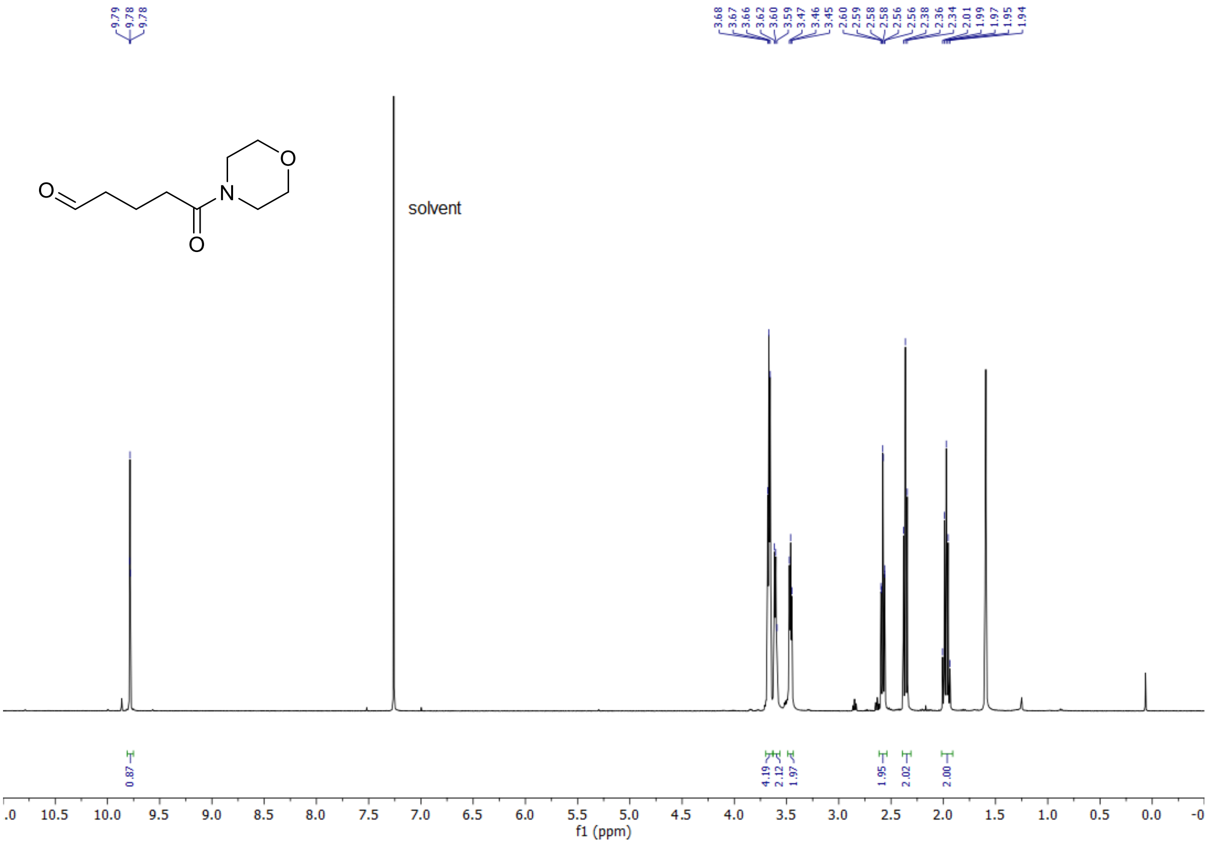

^{13}C NMR (101 MHz, $CDCl_3$) of **S5-2**:

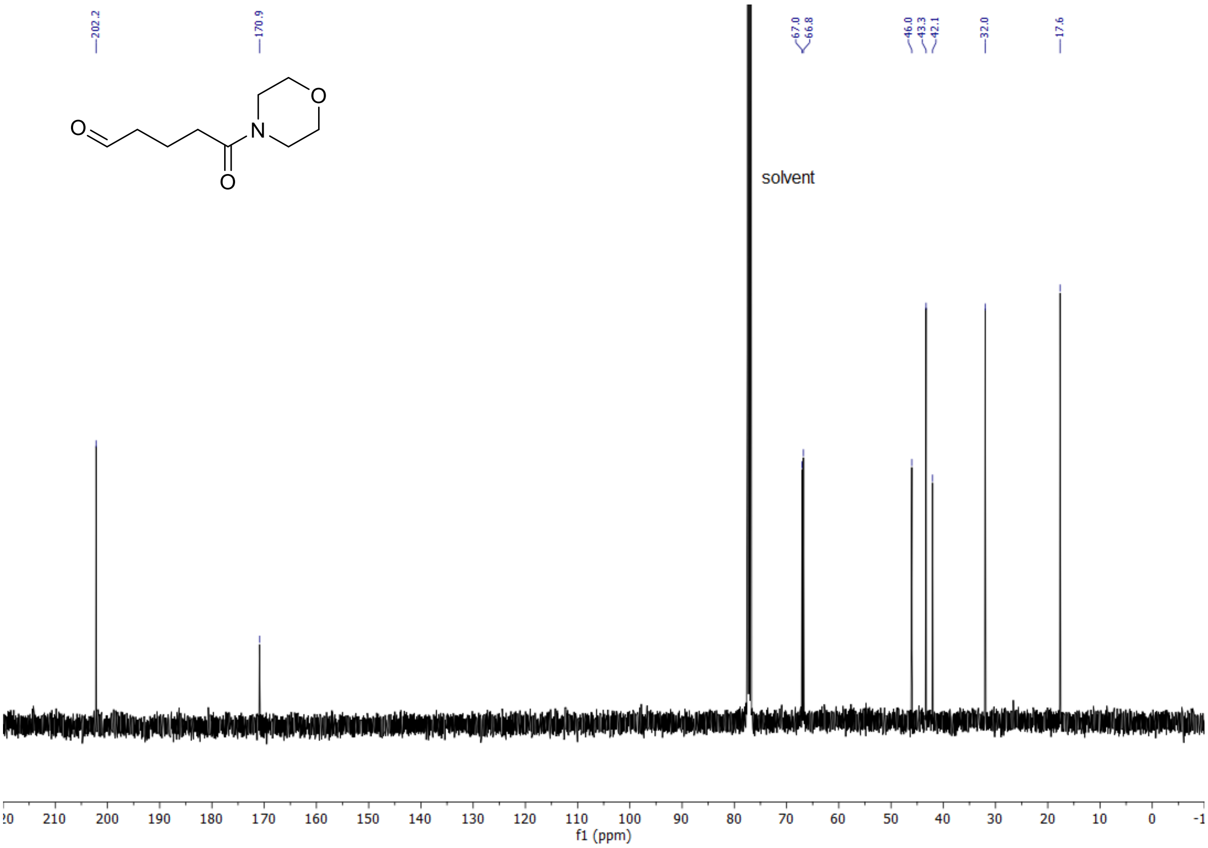

^{1}H NMR (400 MHz, $CDCl_3$) of **12**:

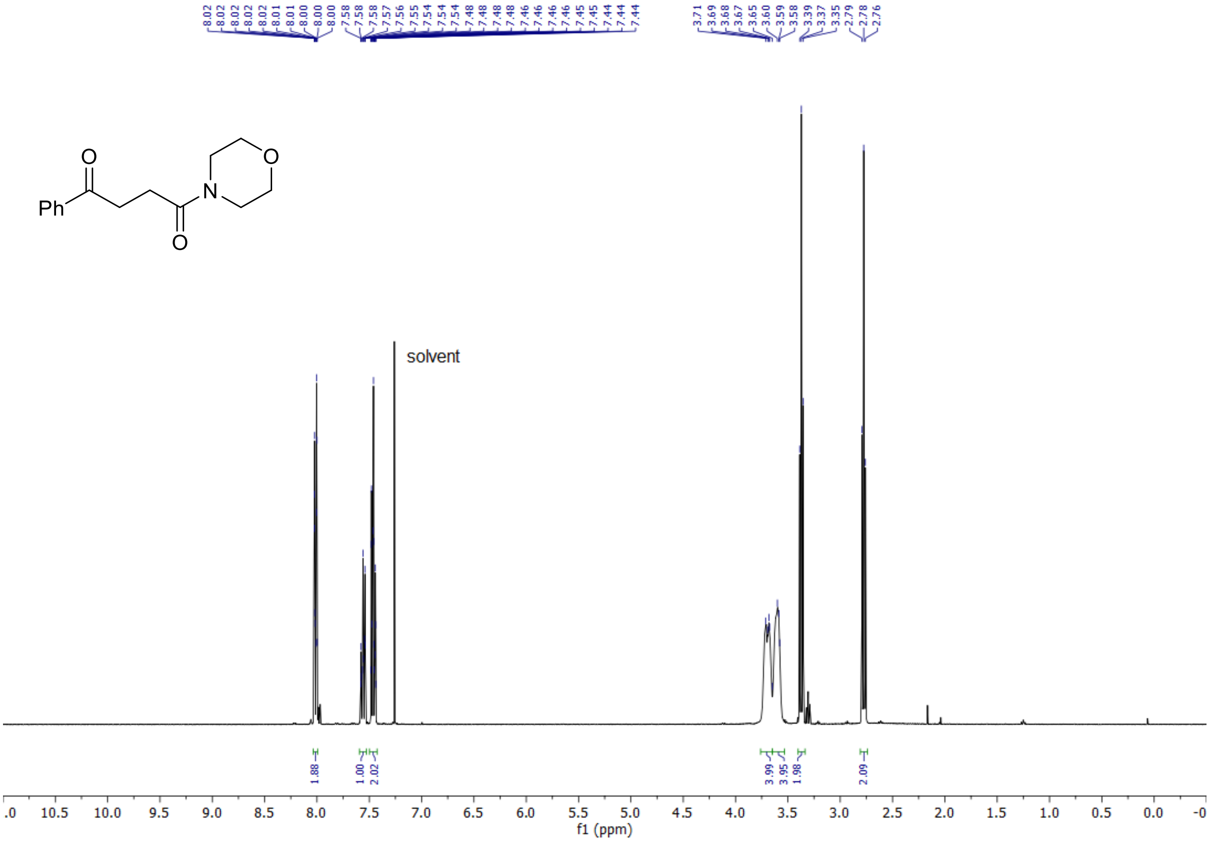

^1H NMR (500 MHz, C_6D_6) of **1a**:

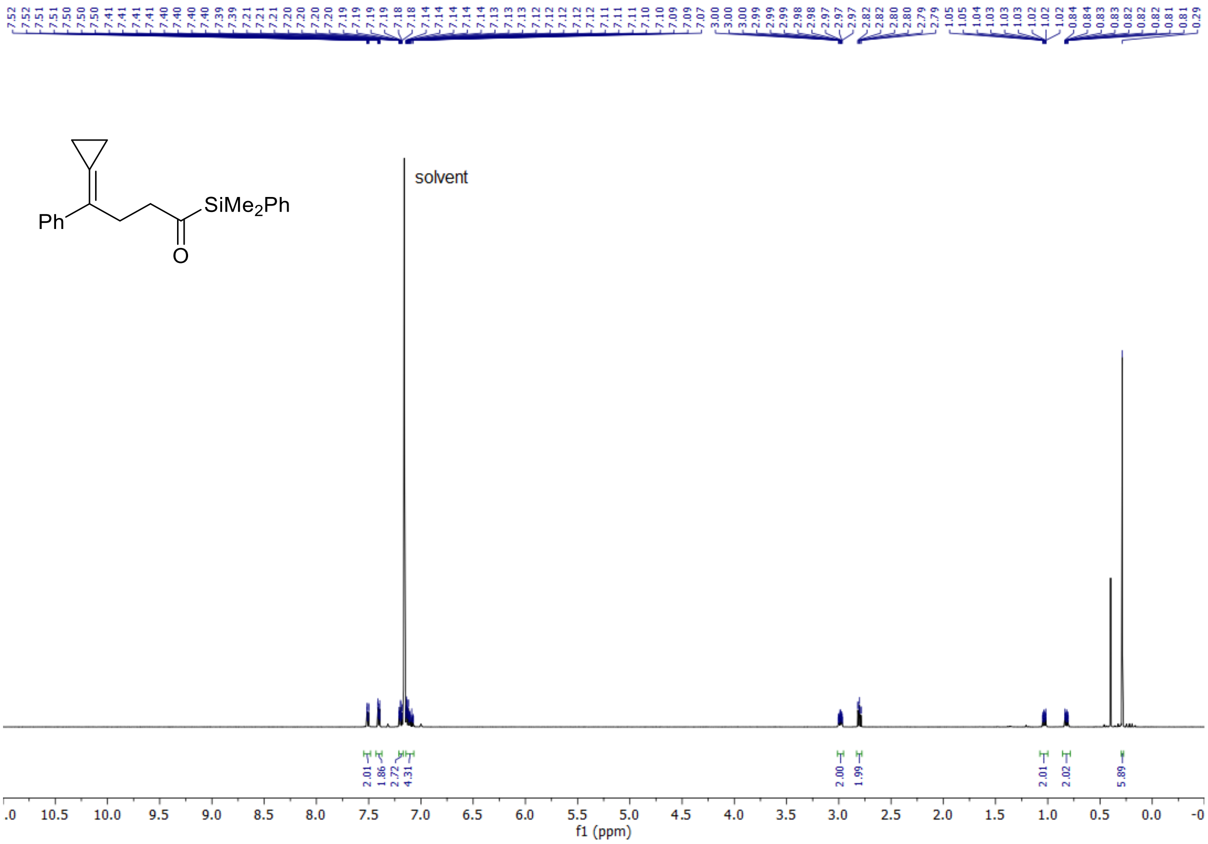

^{13}C NMR (126 MHz, C_6D_6) of **1a**:

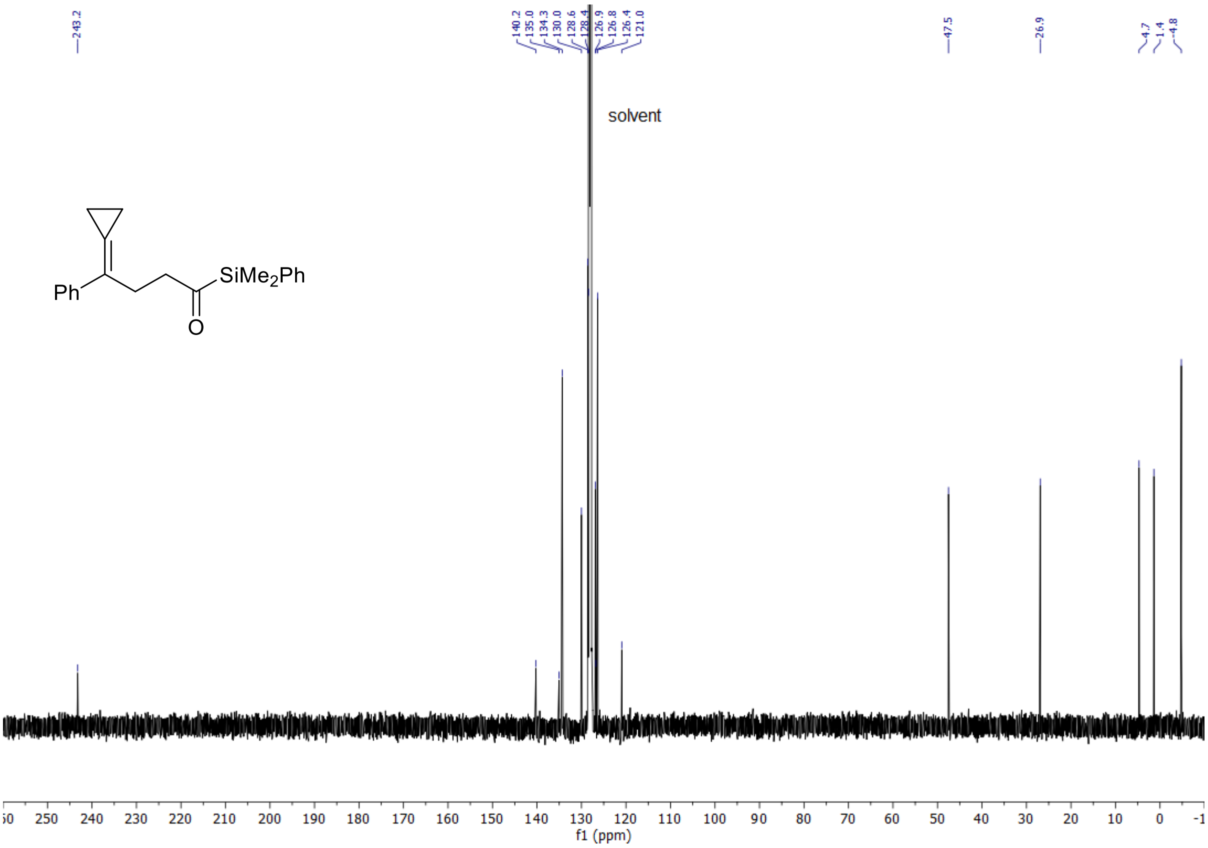

^{1}H NMR (400 MHz, C_6D_6) of **2a**:

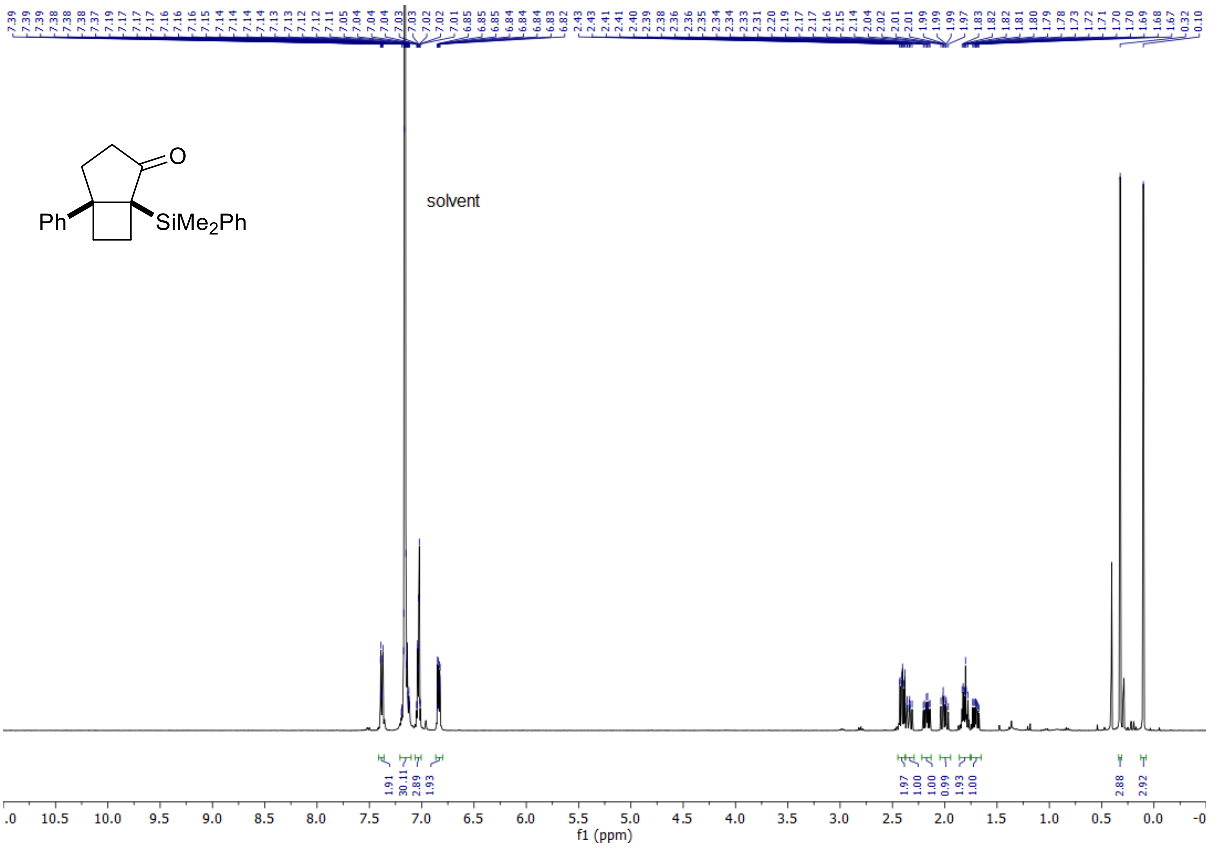

^{13}C NMR (101 MHz, C_6D_6) of **2a**:

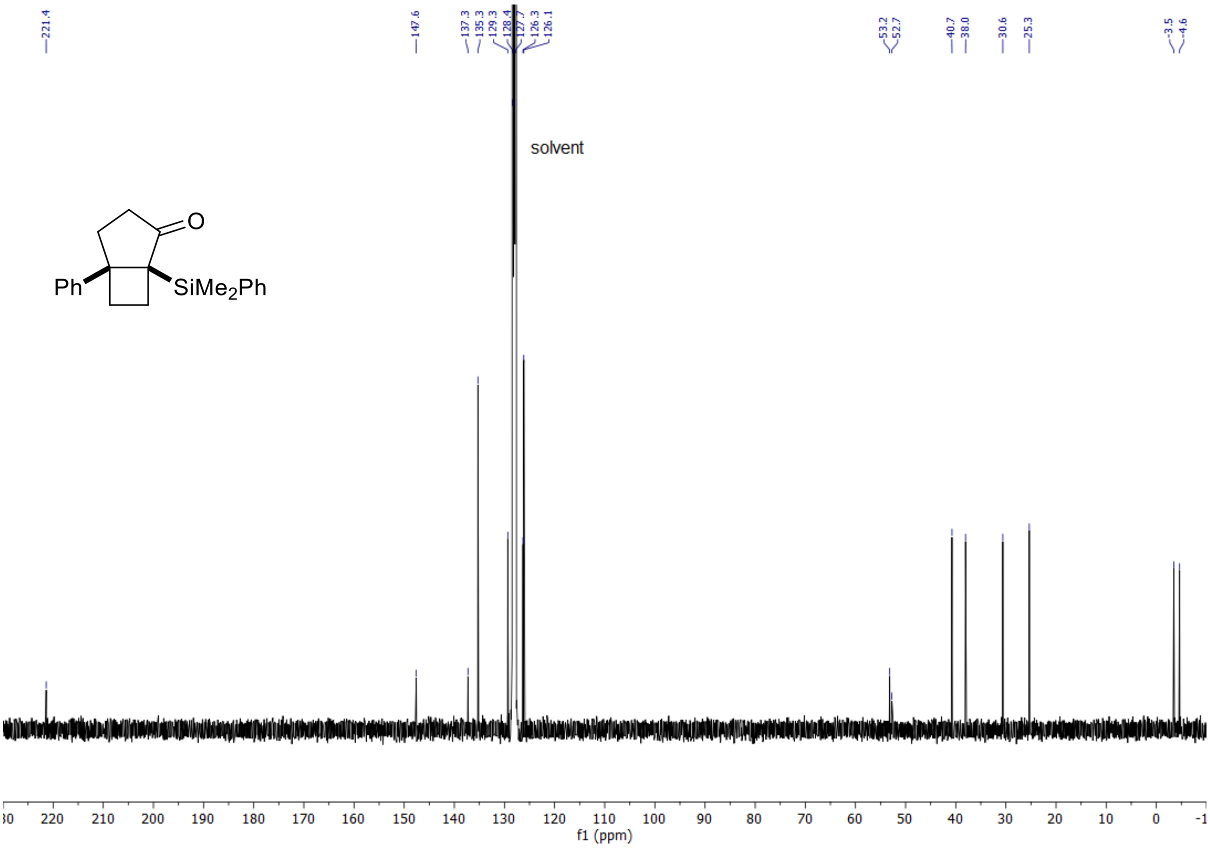

^{1}H NMR (400 MHz, $CDCl_3$) of **S2b**:

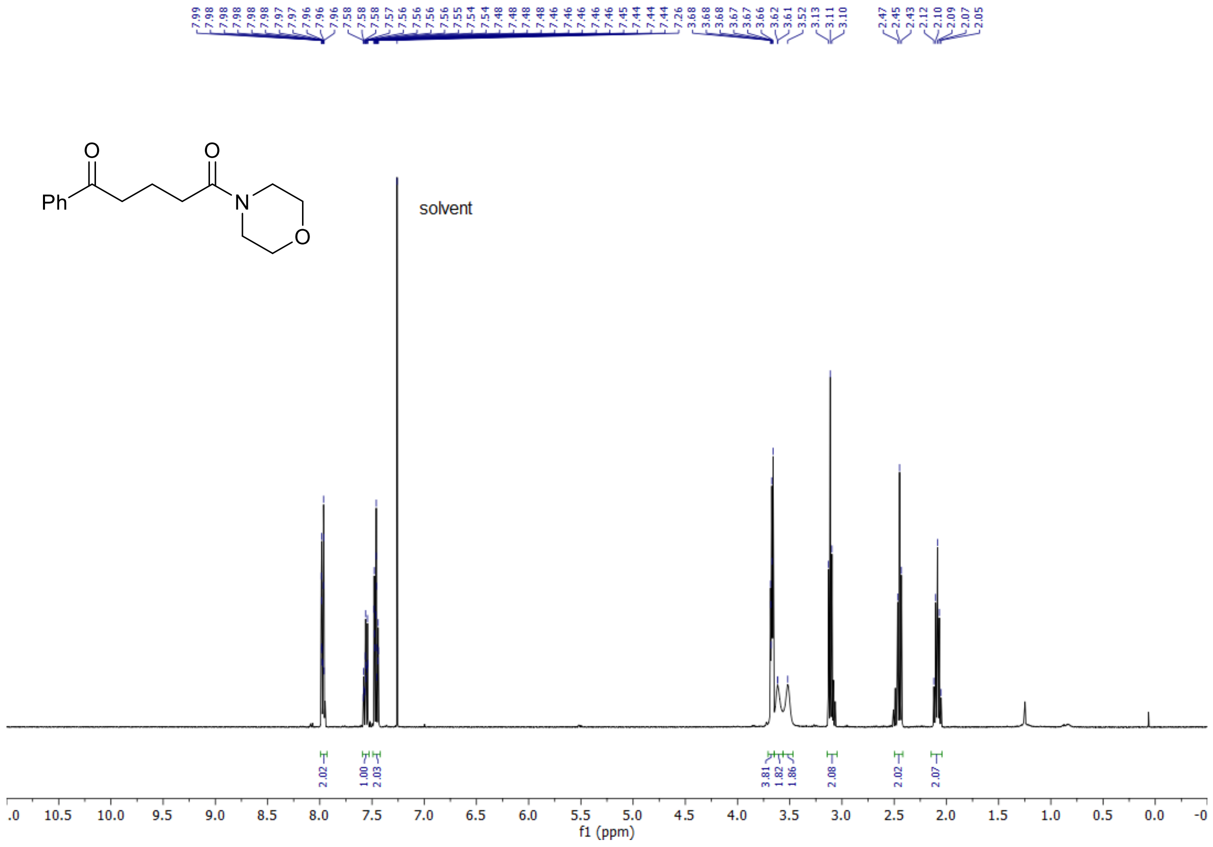

^{13}C NMR (101 MHz, $CDCl_3$) of **S2b**:

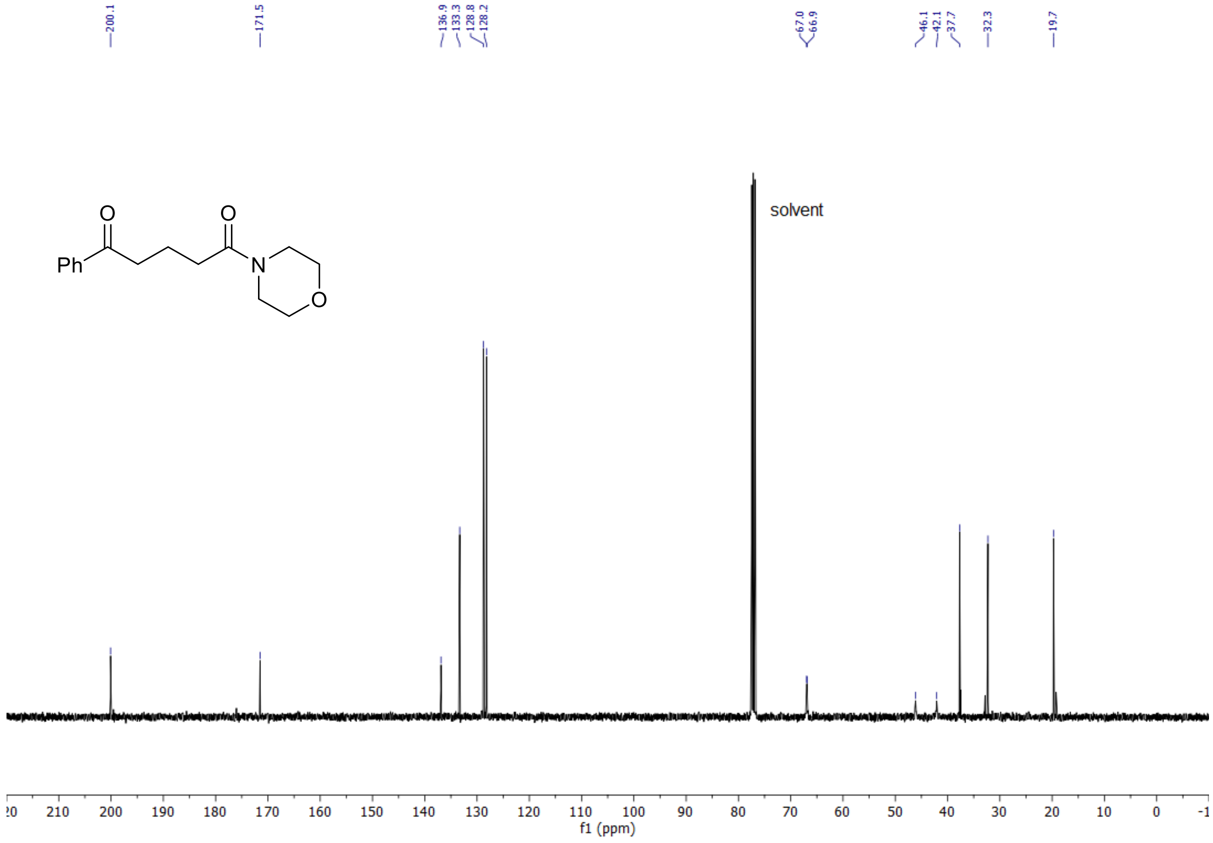

^{1}H NMR (500 MHz, C_6D_6) of **1b**:

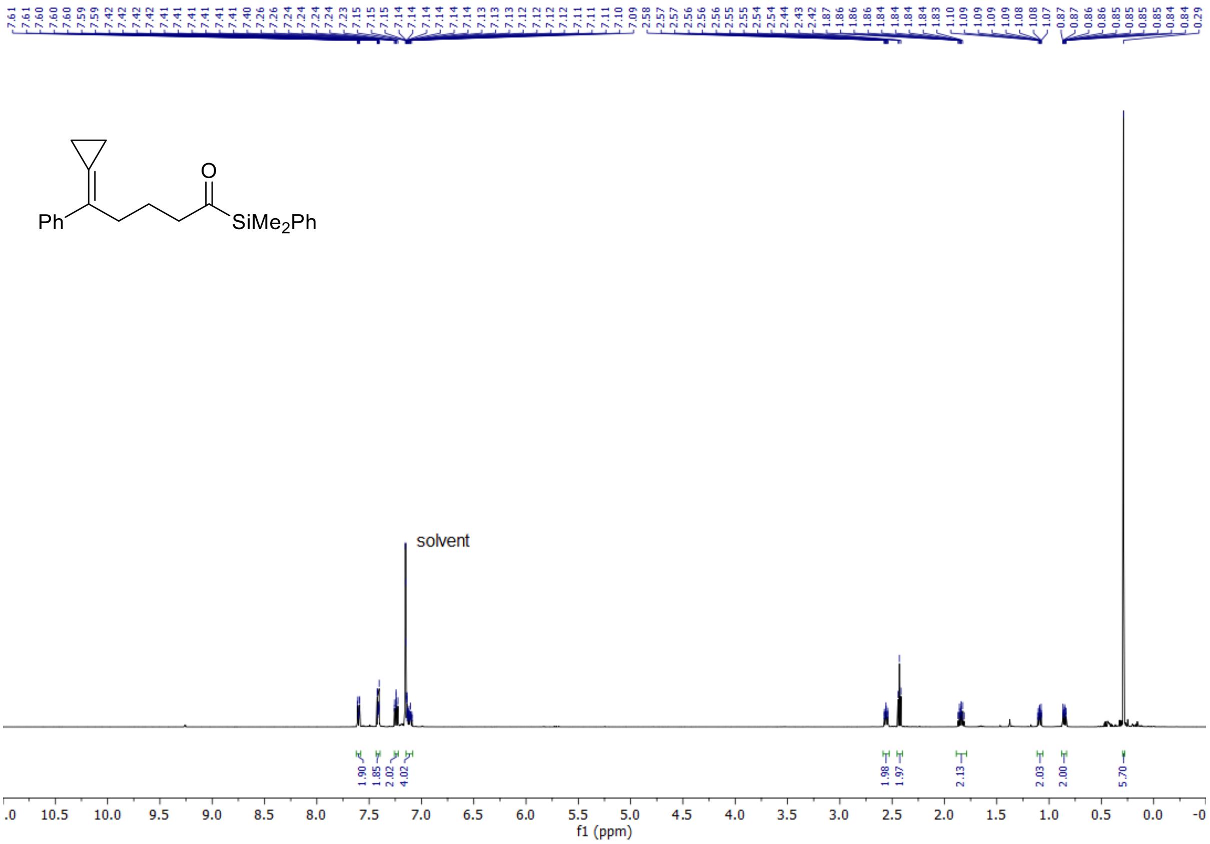

^{13}C NMR (126 MHz, C_6D_6) of **1b**:

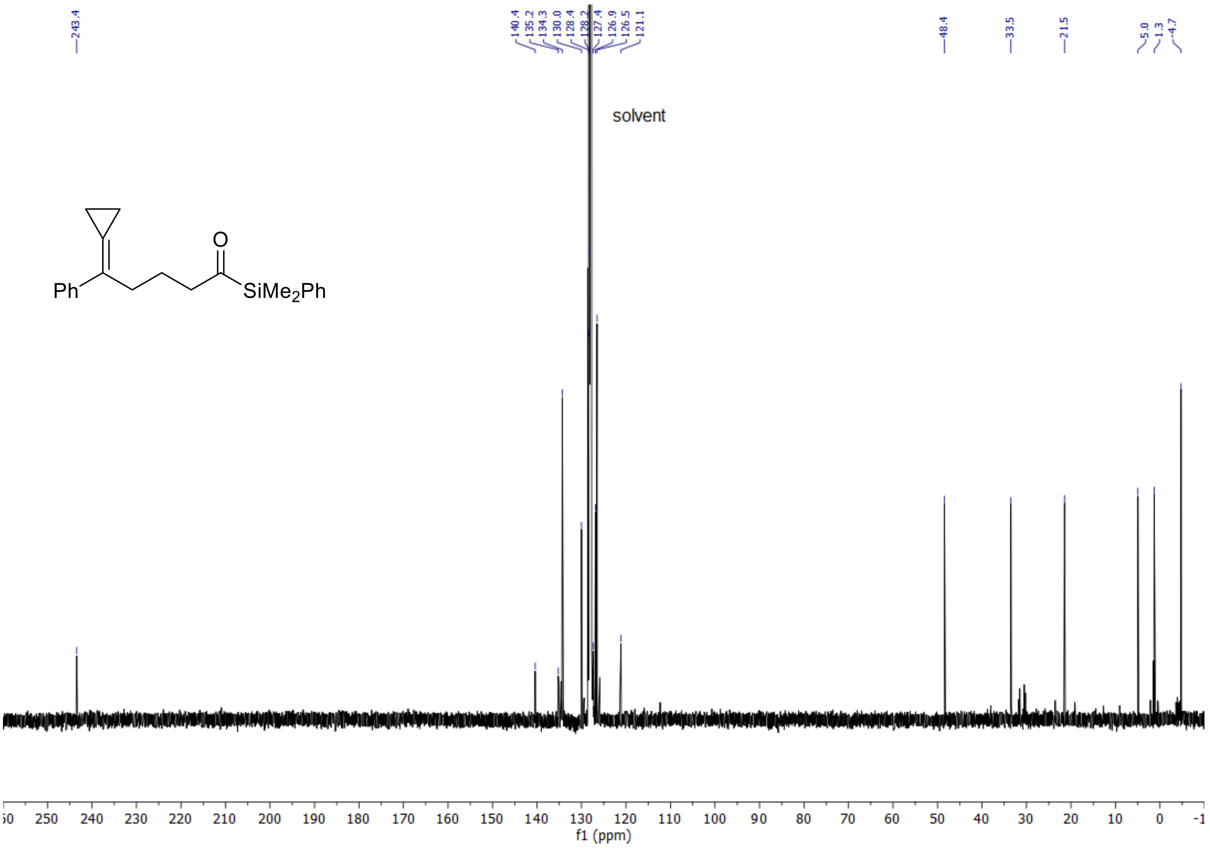

^1H NMR (500 MHz, C_6D_6) of **2b**:

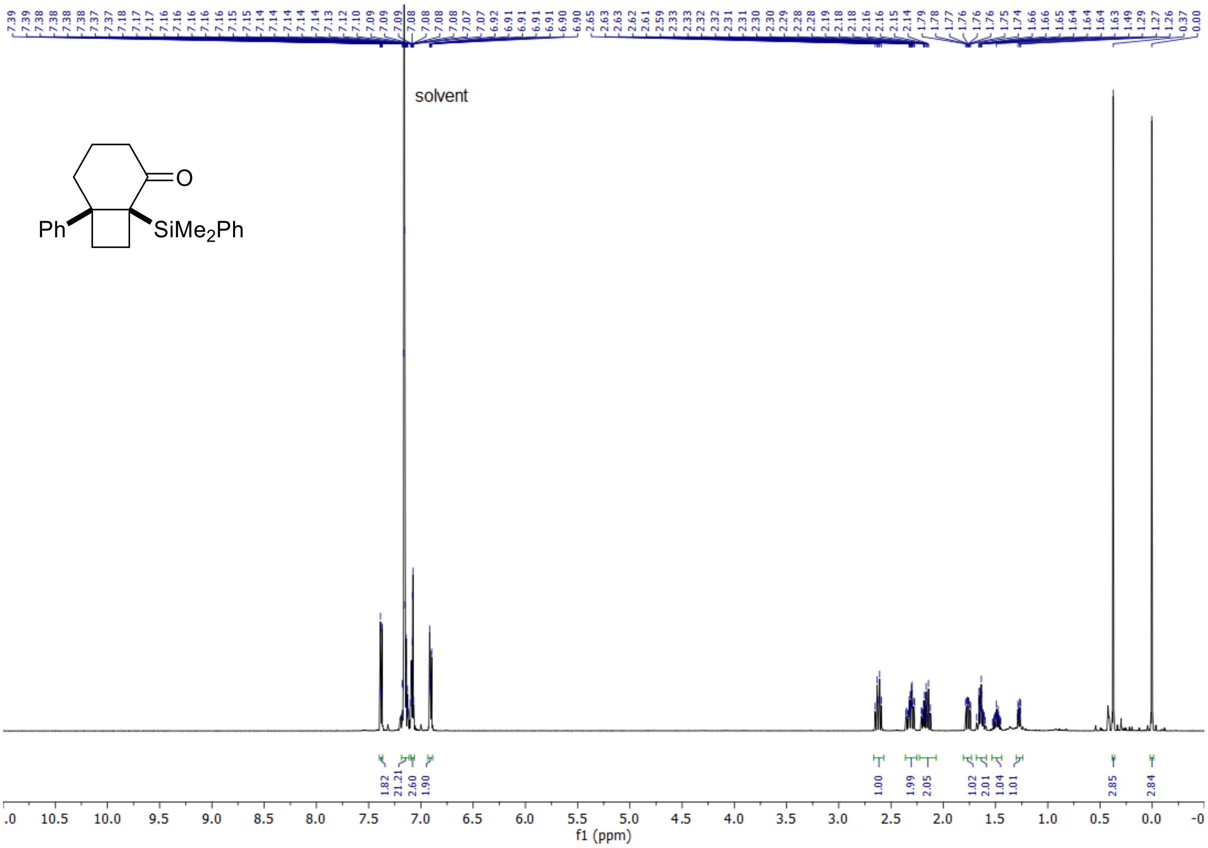

^{13}C NMR (126 MHz, C_6D_6) of **2b**:

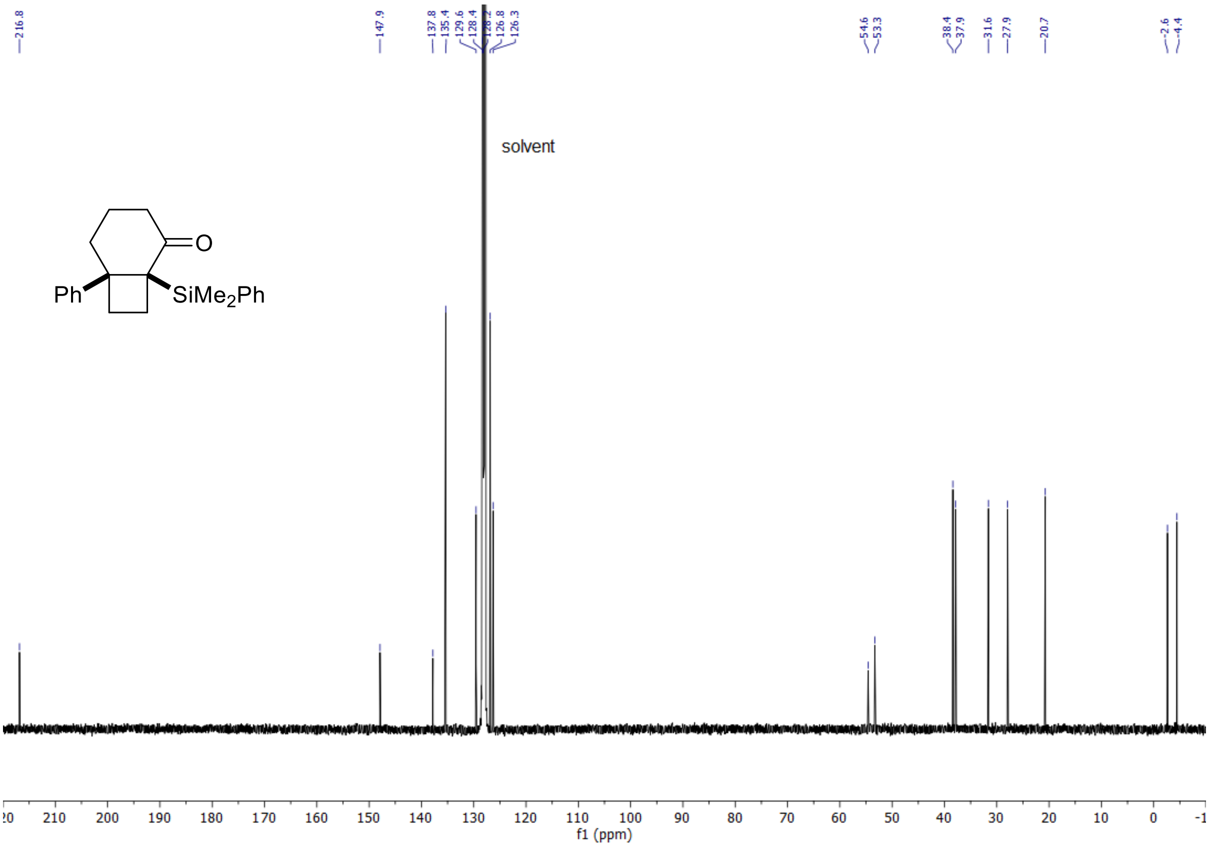

^{1}H NMR (400 MHz, $CDCl_3$) of **S2c**:

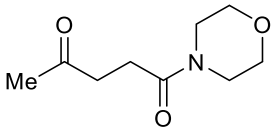

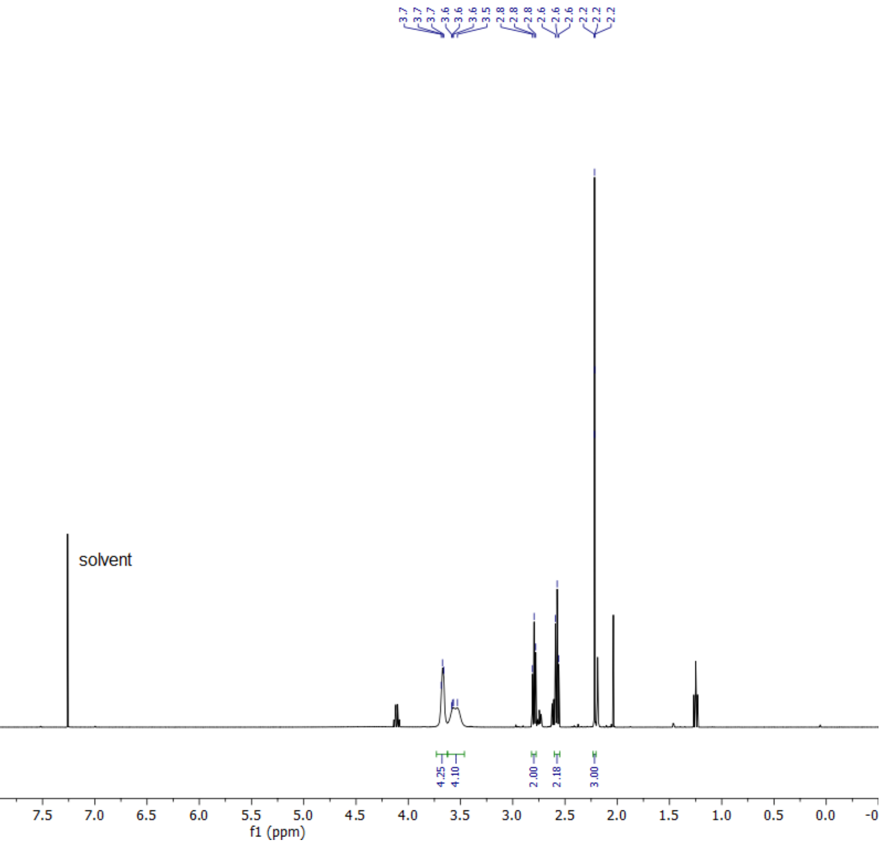

^{1}H NMR (500 MHz, C_6D_6) of **1c**:

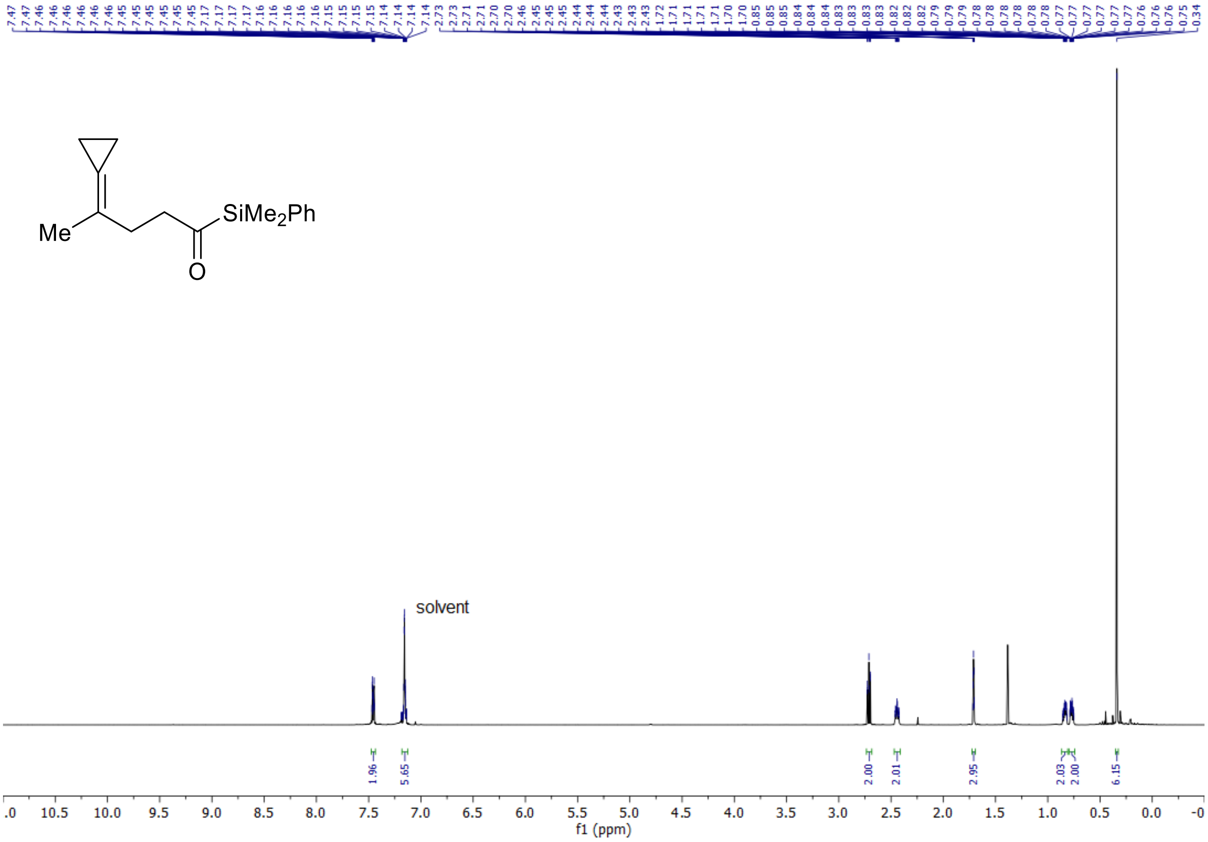

^{13}C NMR (126 MHz, C_6D_6) of **1c**:

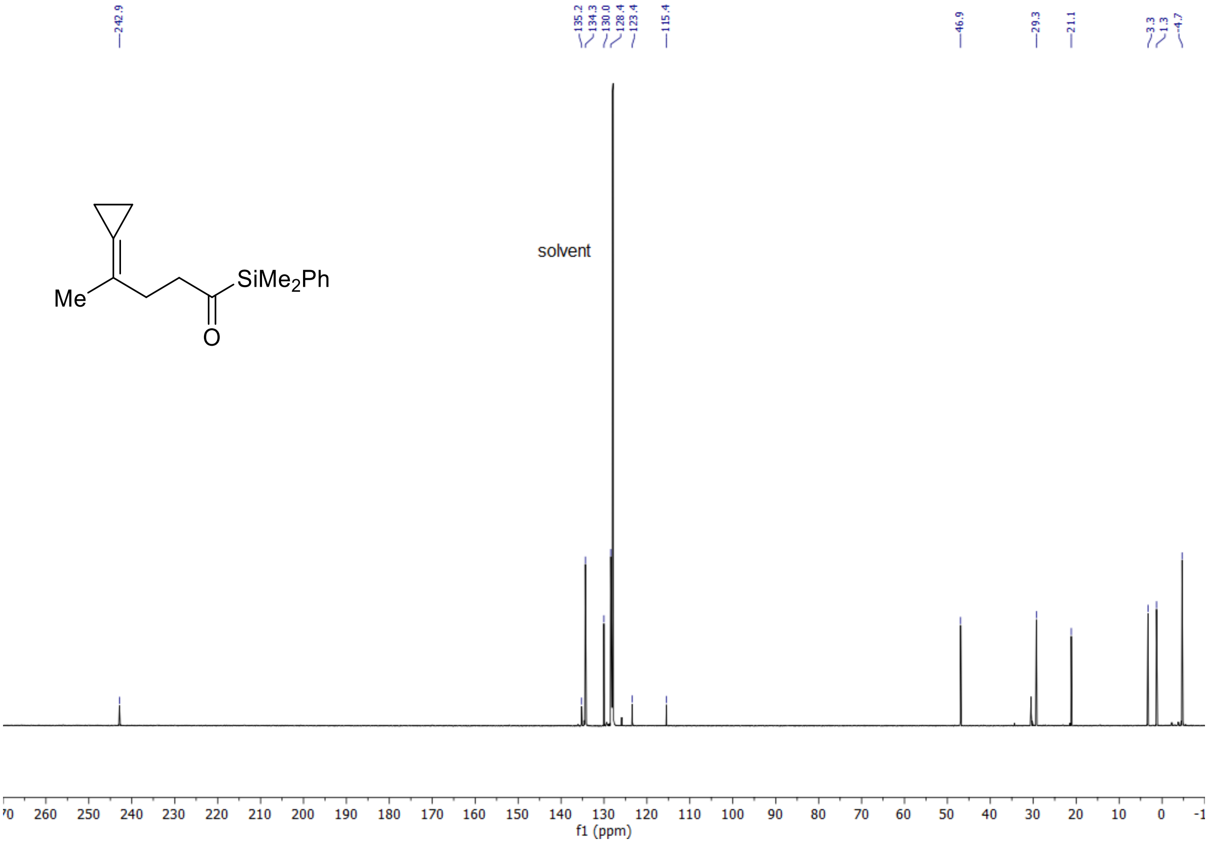

^{1}H NMR (500 MHz, C_6D_6) of **2c**:

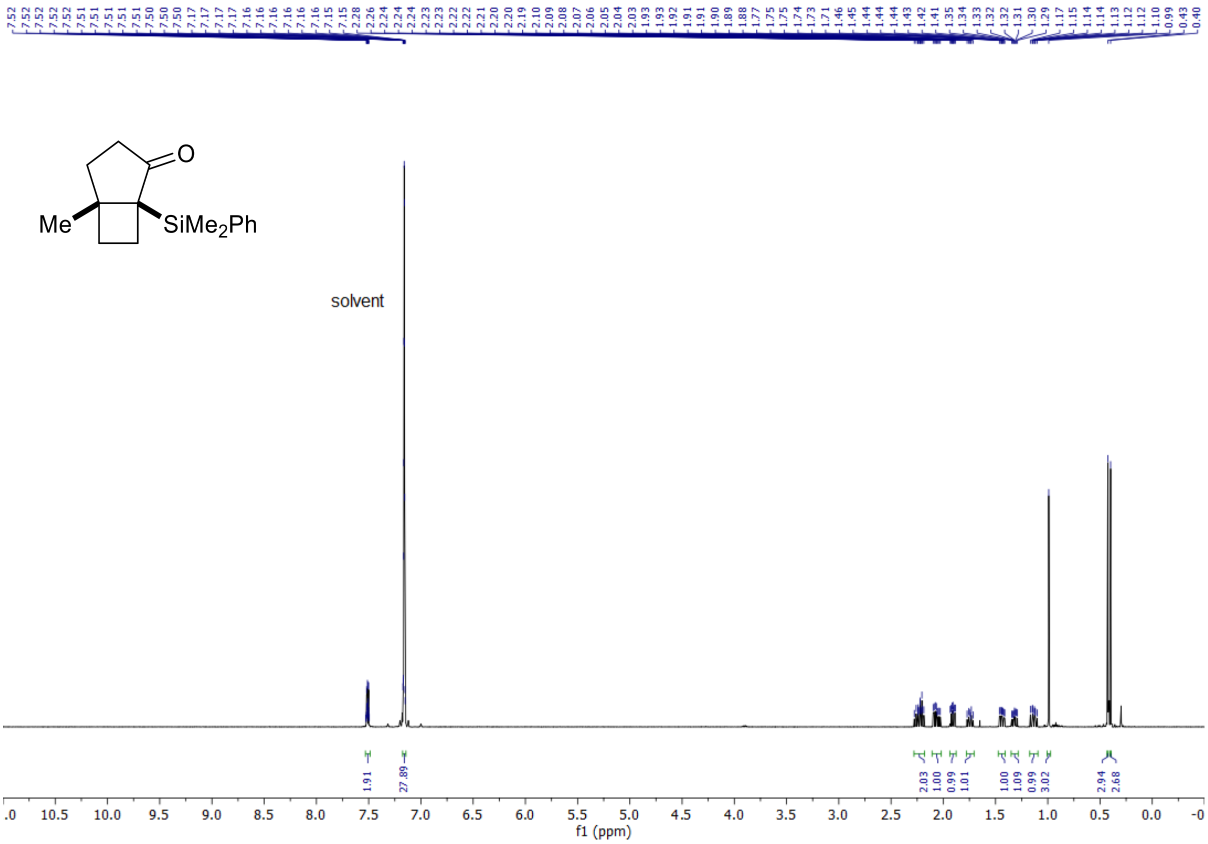

^{13}C NMR (126 MHz, C_6D_6) of **2c**:

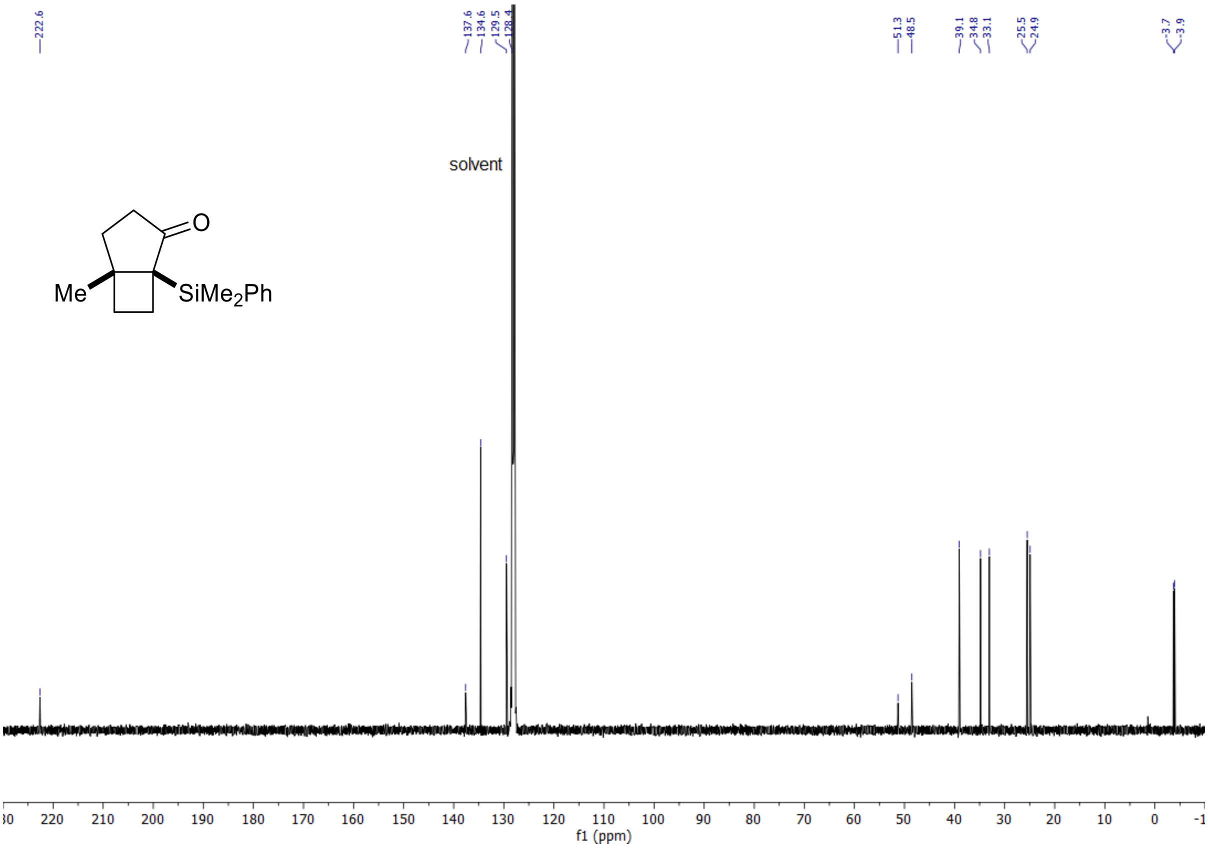

^{1}H NMR (400 MHz, $CDCl_3$) of **S2d**:

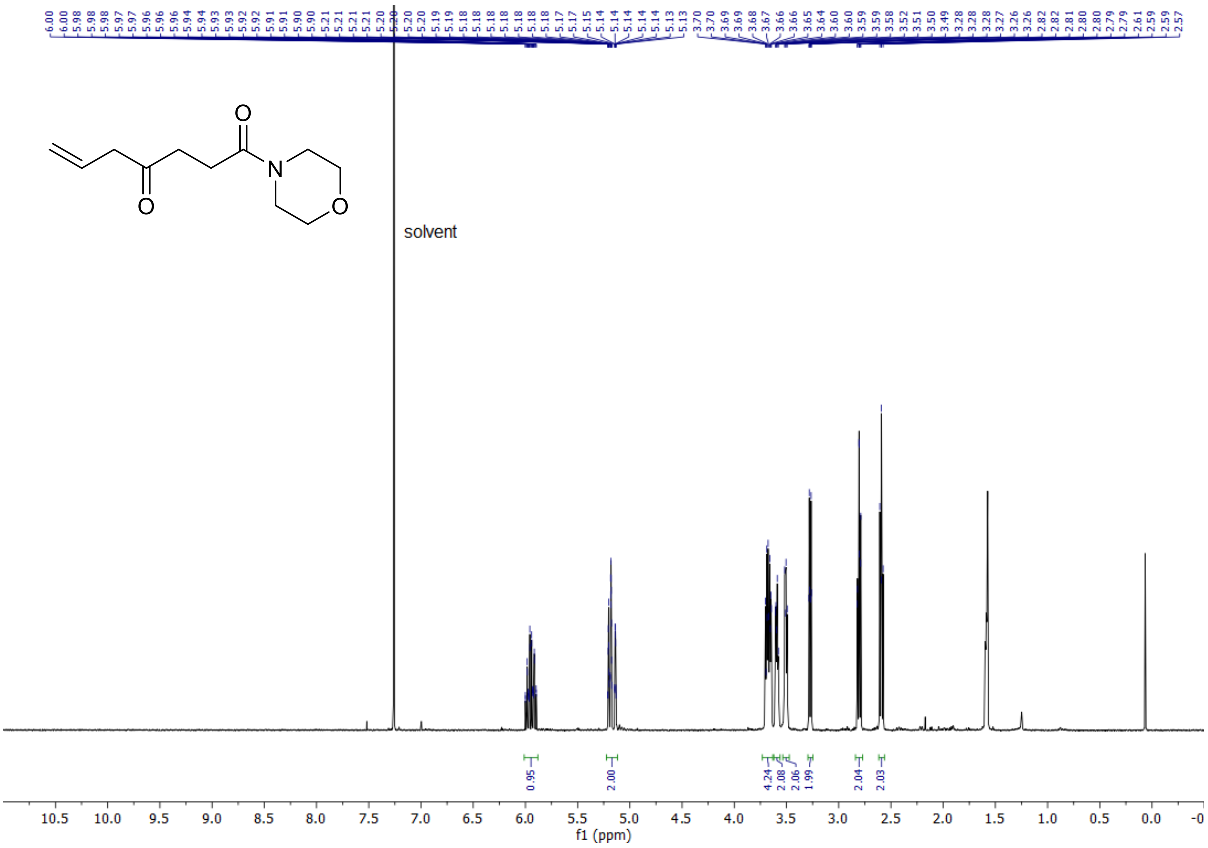

^{13}C NMR (101 MHz, $CDCl_3$) of **S2d**:

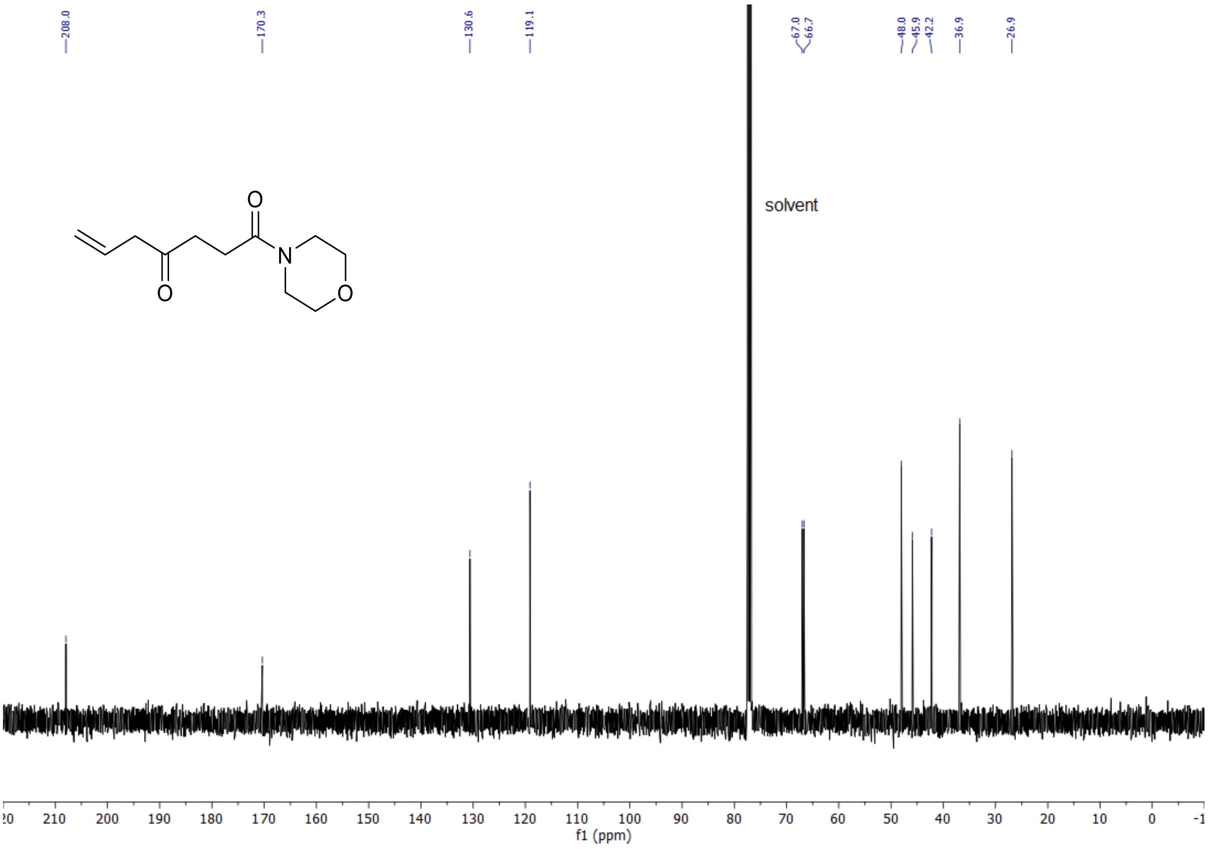

^{1}H NMR (400 MHz, C_6D_6) of **1d**:

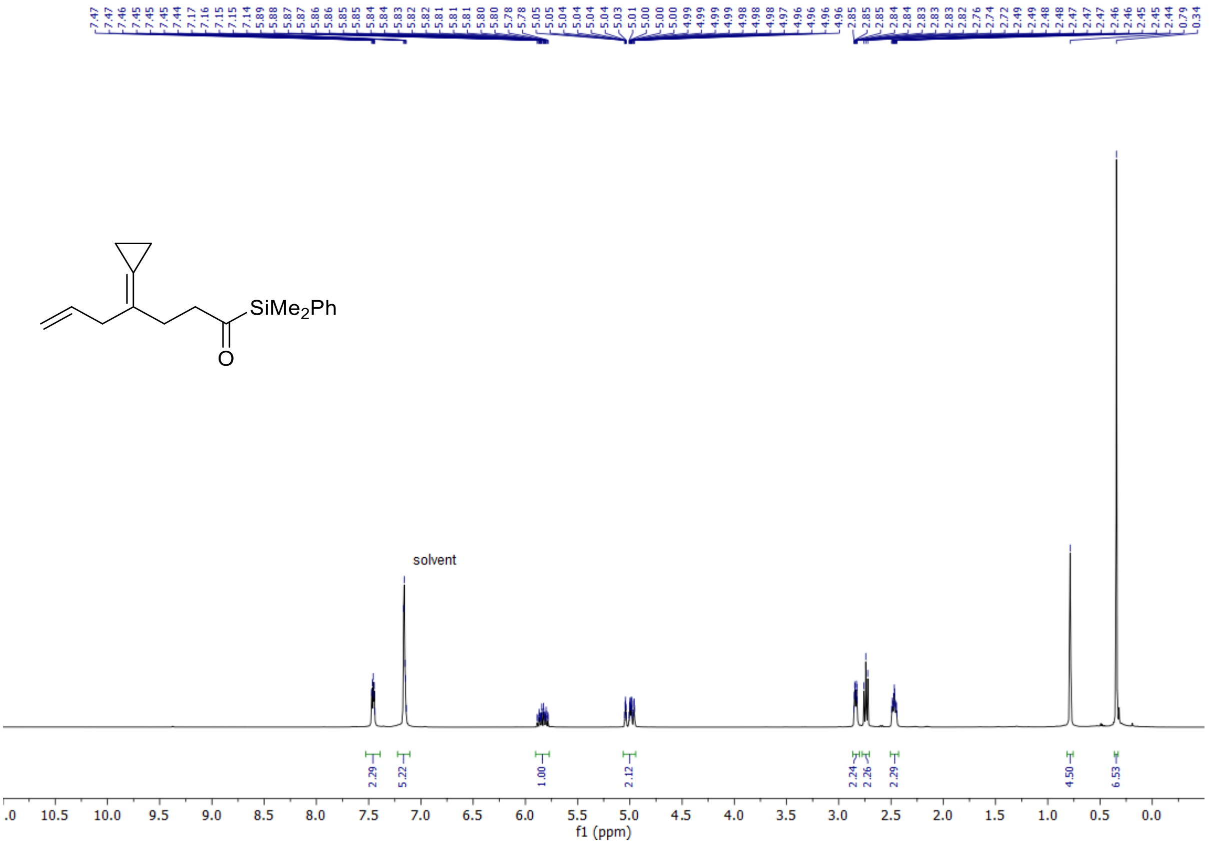

^{13}C NMR (101 MHz, C_6D_6) of **1d**:

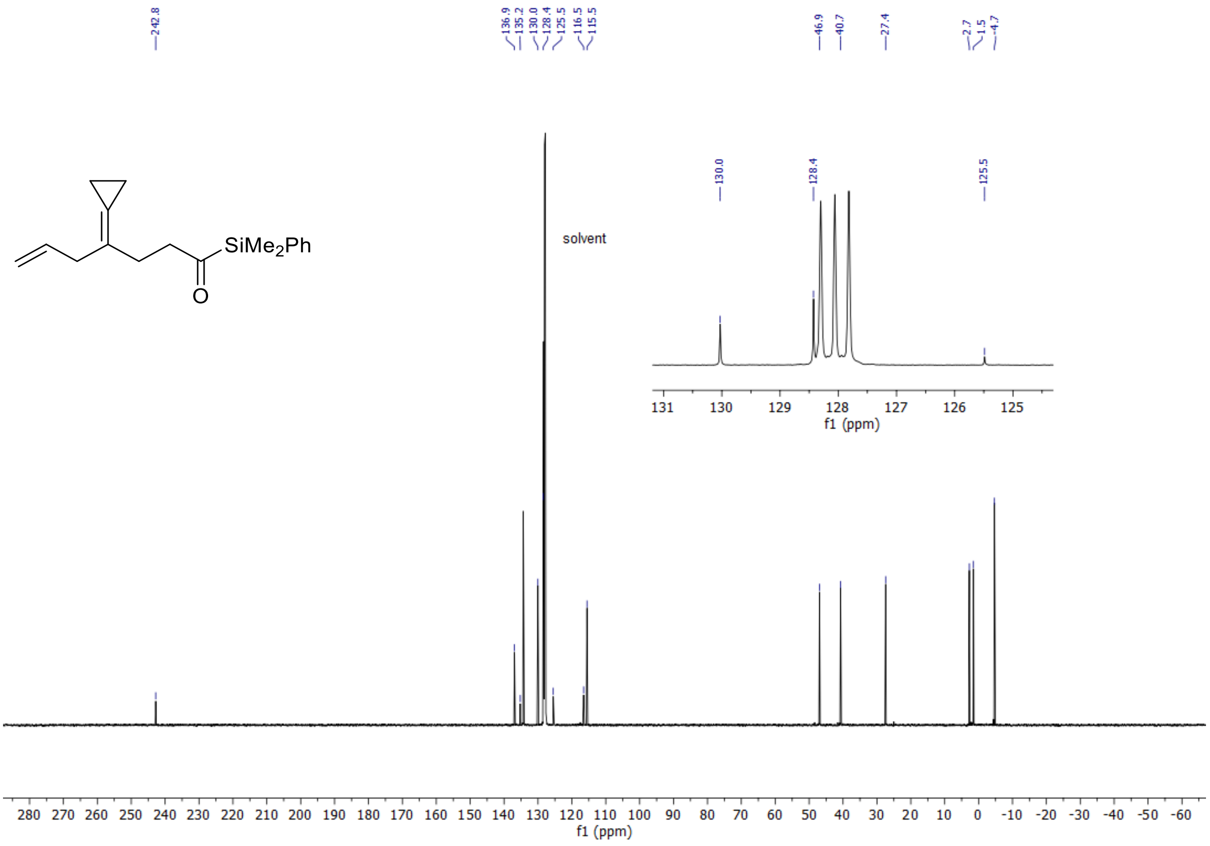

^{1}H NMR (500 MHz, C_6D_6) of **2d**:

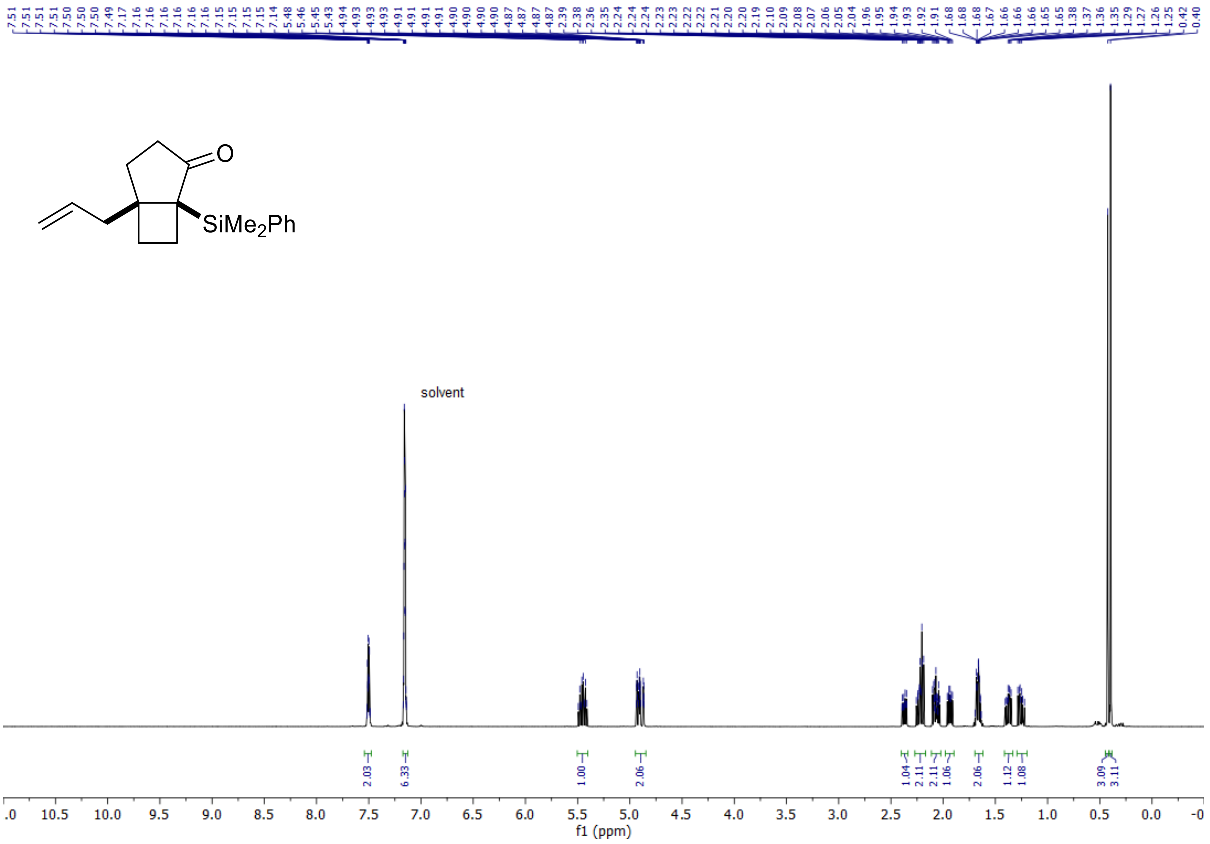

^{13}C NMR (126 MHz, C_6D_6) of **2d**:

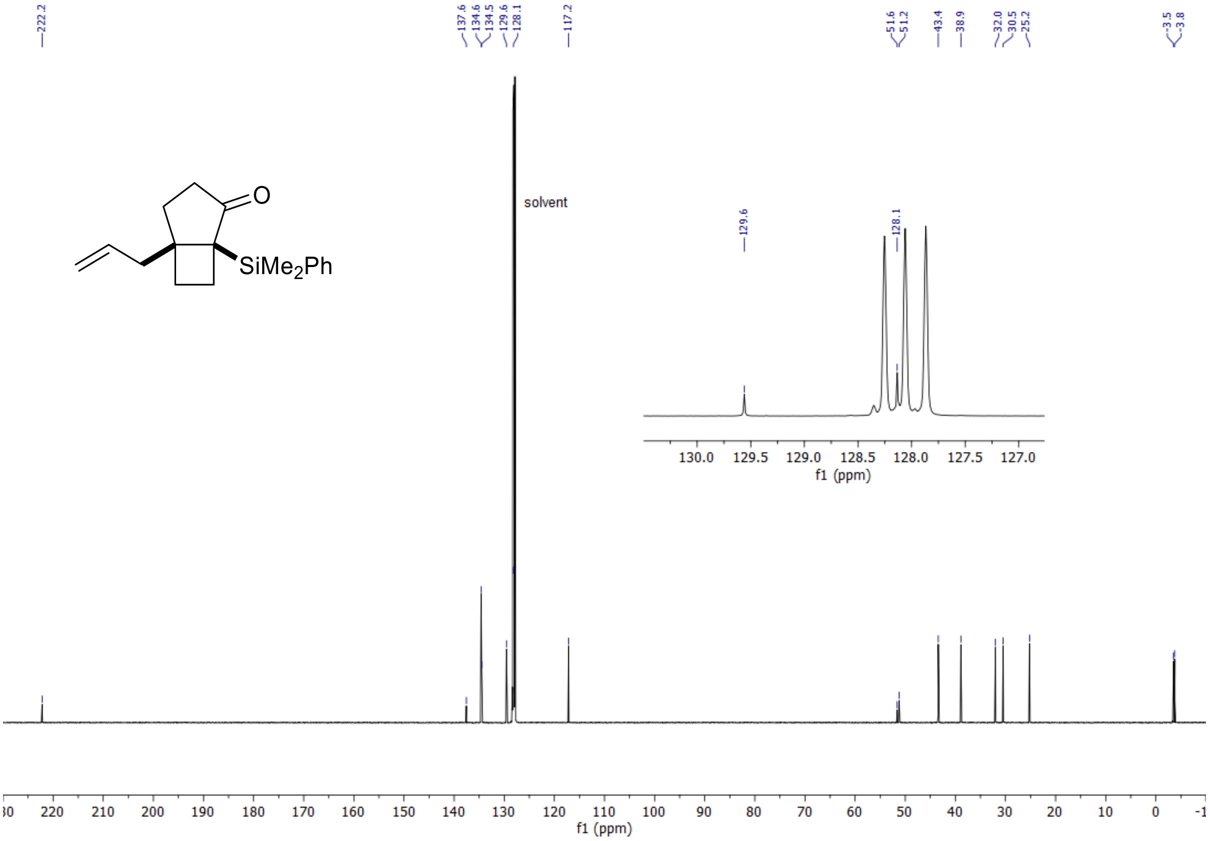

^1H NMR (500 MHz, $CDCl_3$) of **S2e**:

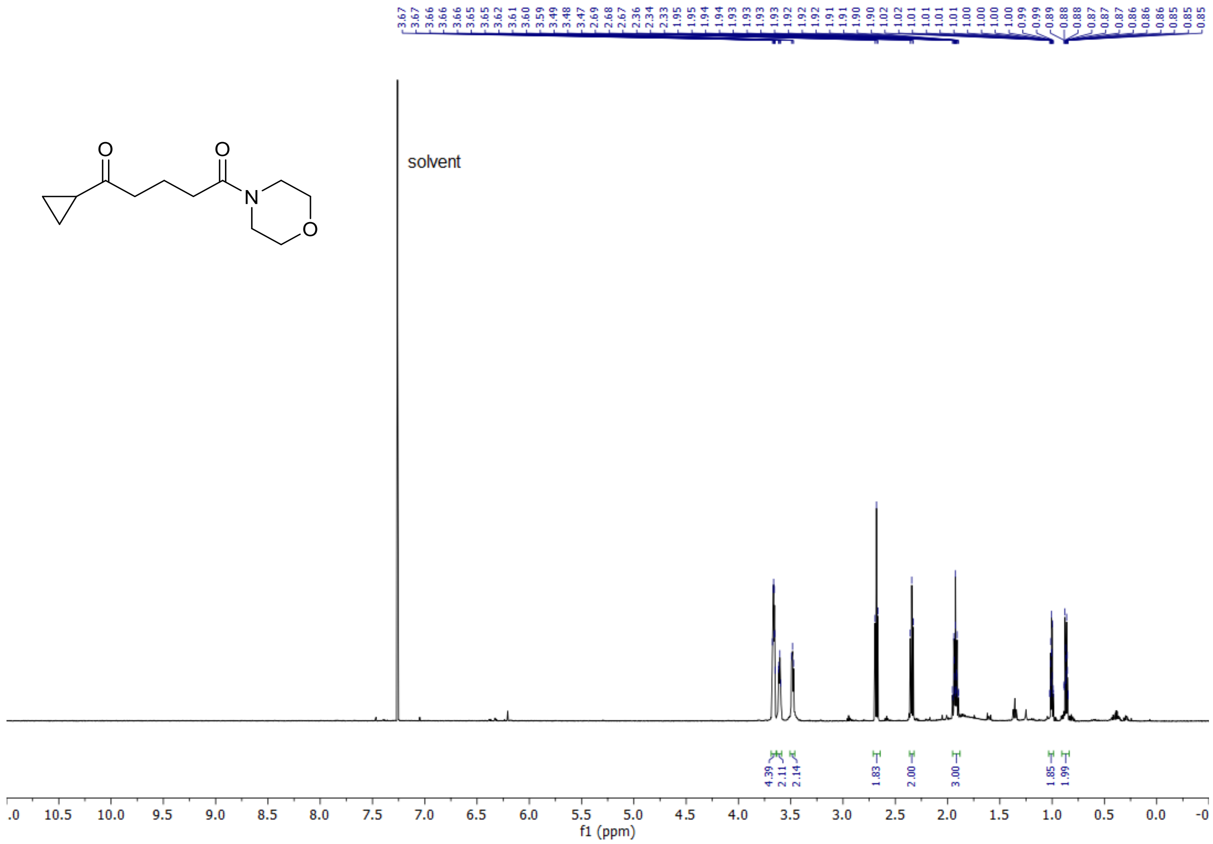

^{13}C NMR (126 MHz, $CDCl_3$) of **S2e**:

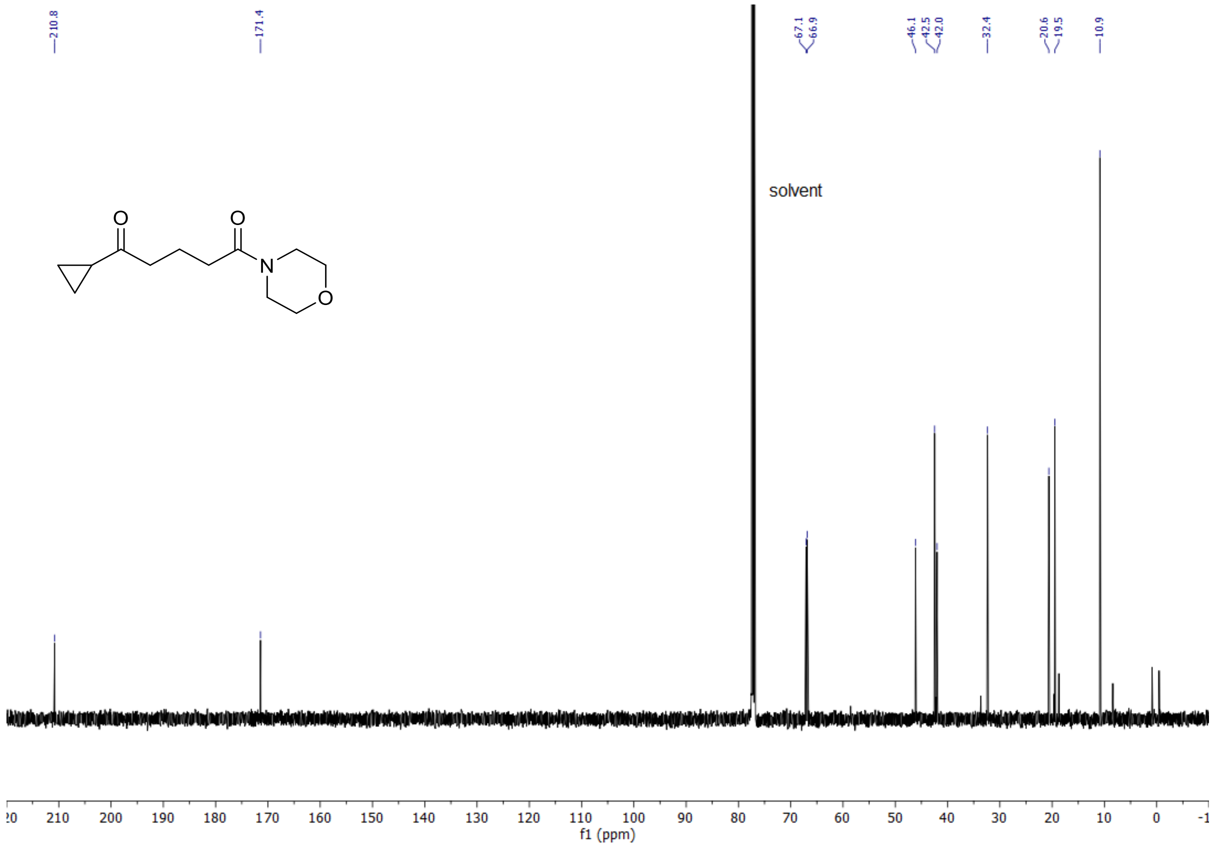

^1H NMR (500 MHz, C_6D_6) of **1e**:

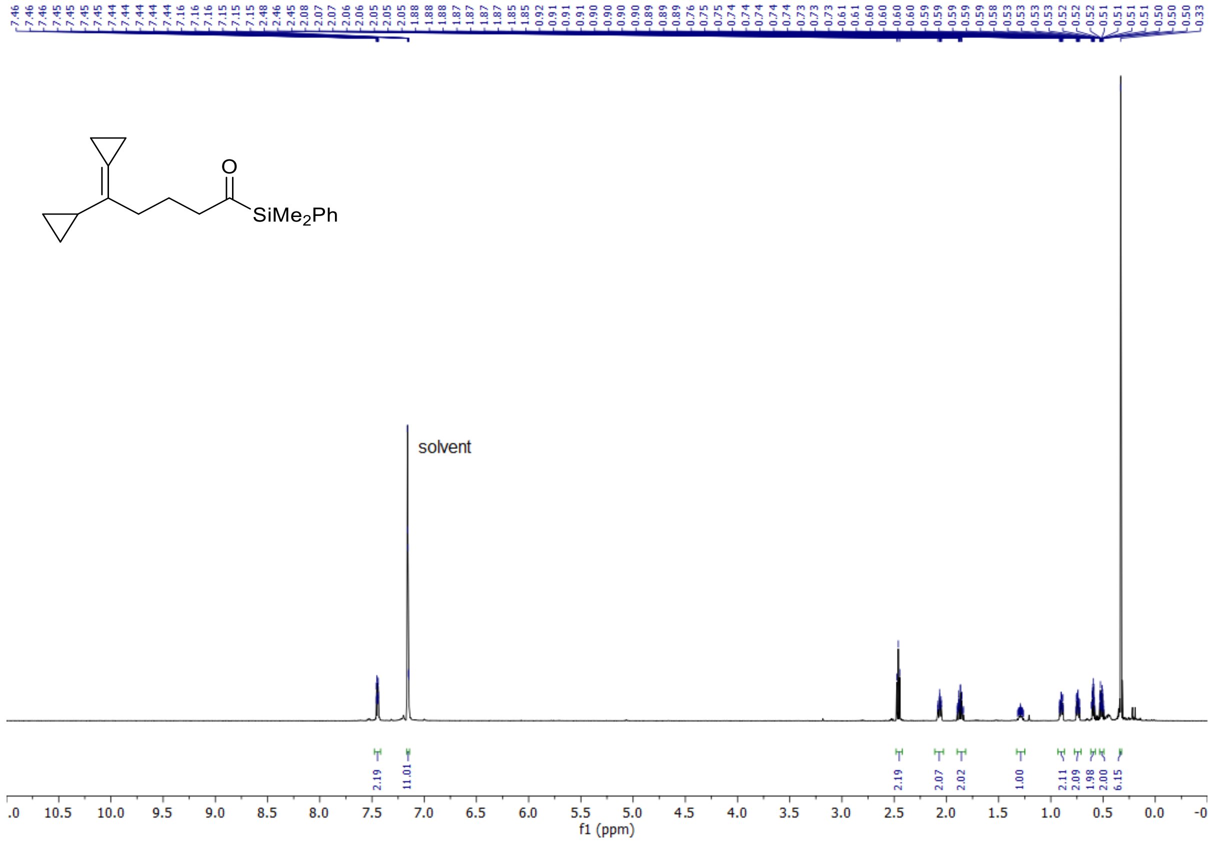

^{13}C NMR (126 MHz, C_6D_6) of **1e**:

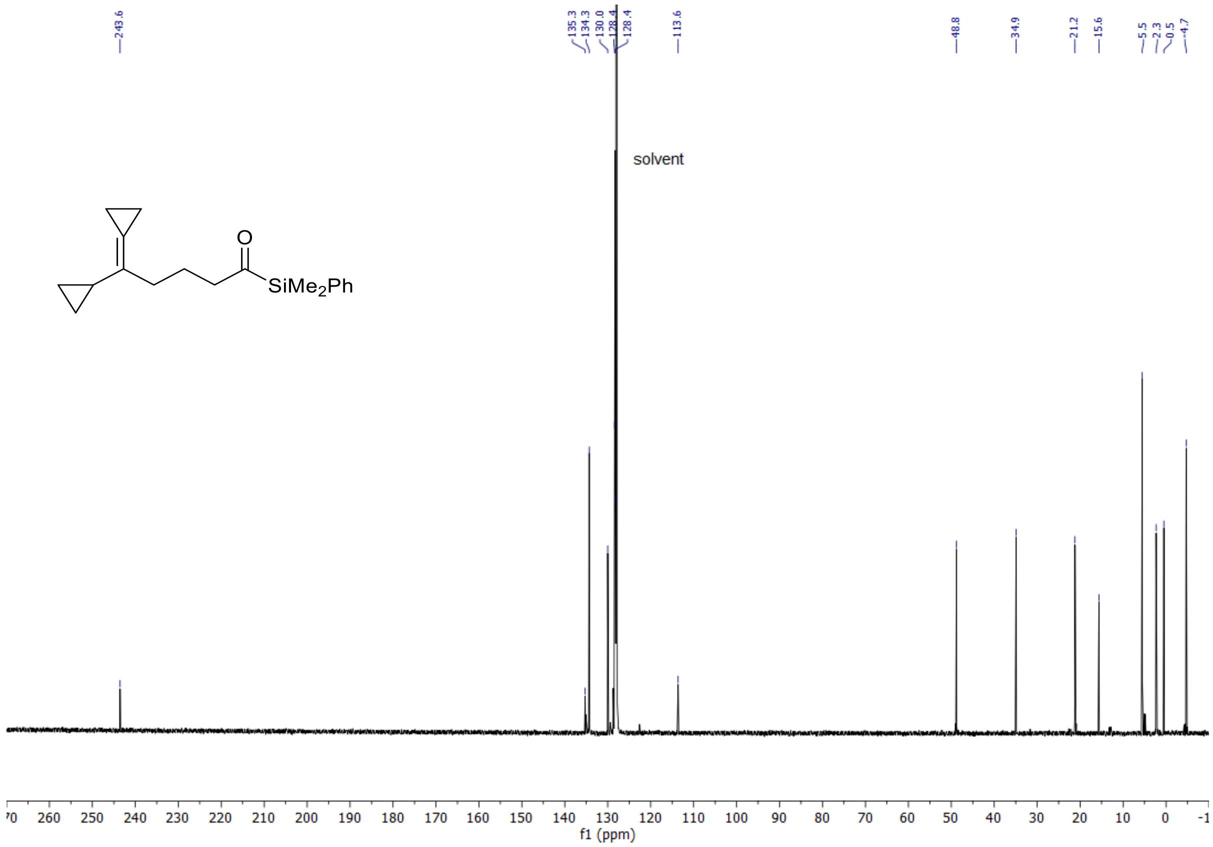

^{1}H NMR (400 MHz, C_6D_6) of **2e**:

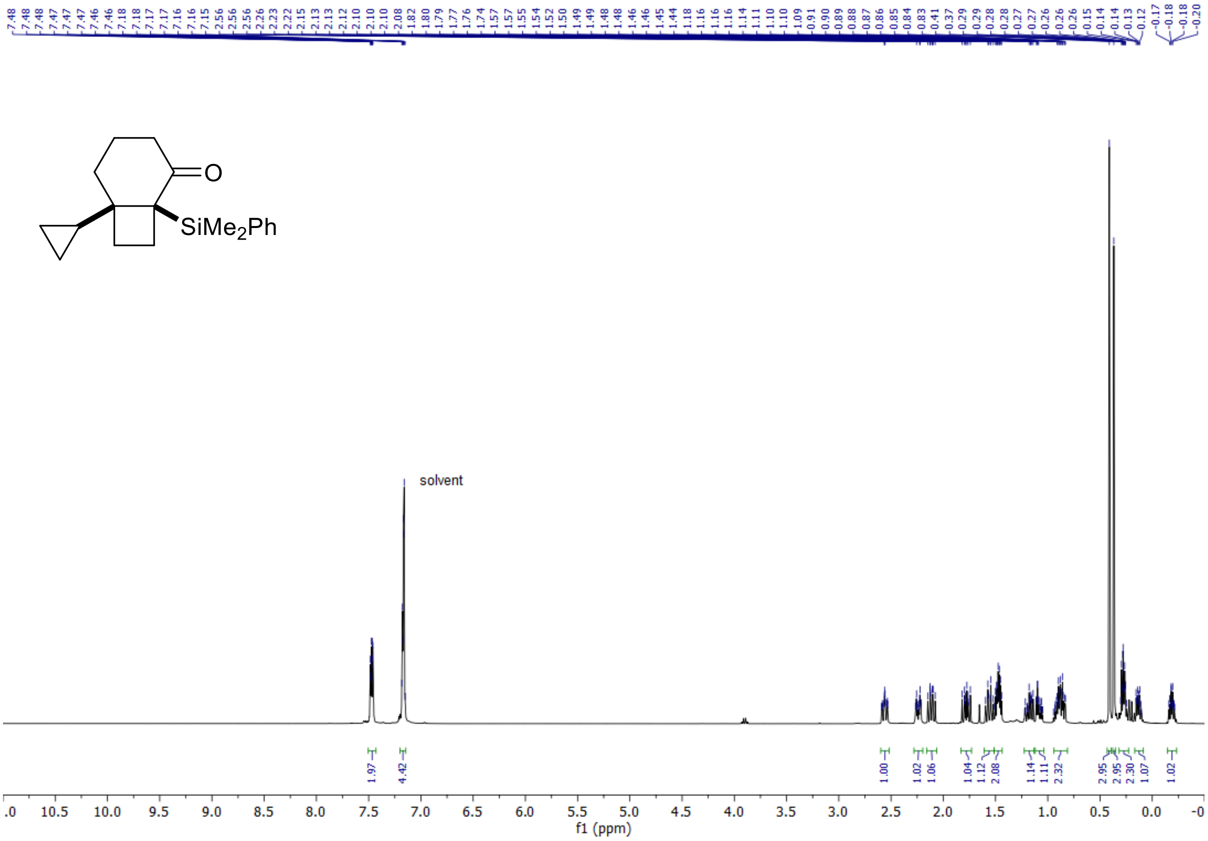

^{13}C NMR (101 MHz, C_6D_6) of **2e**:

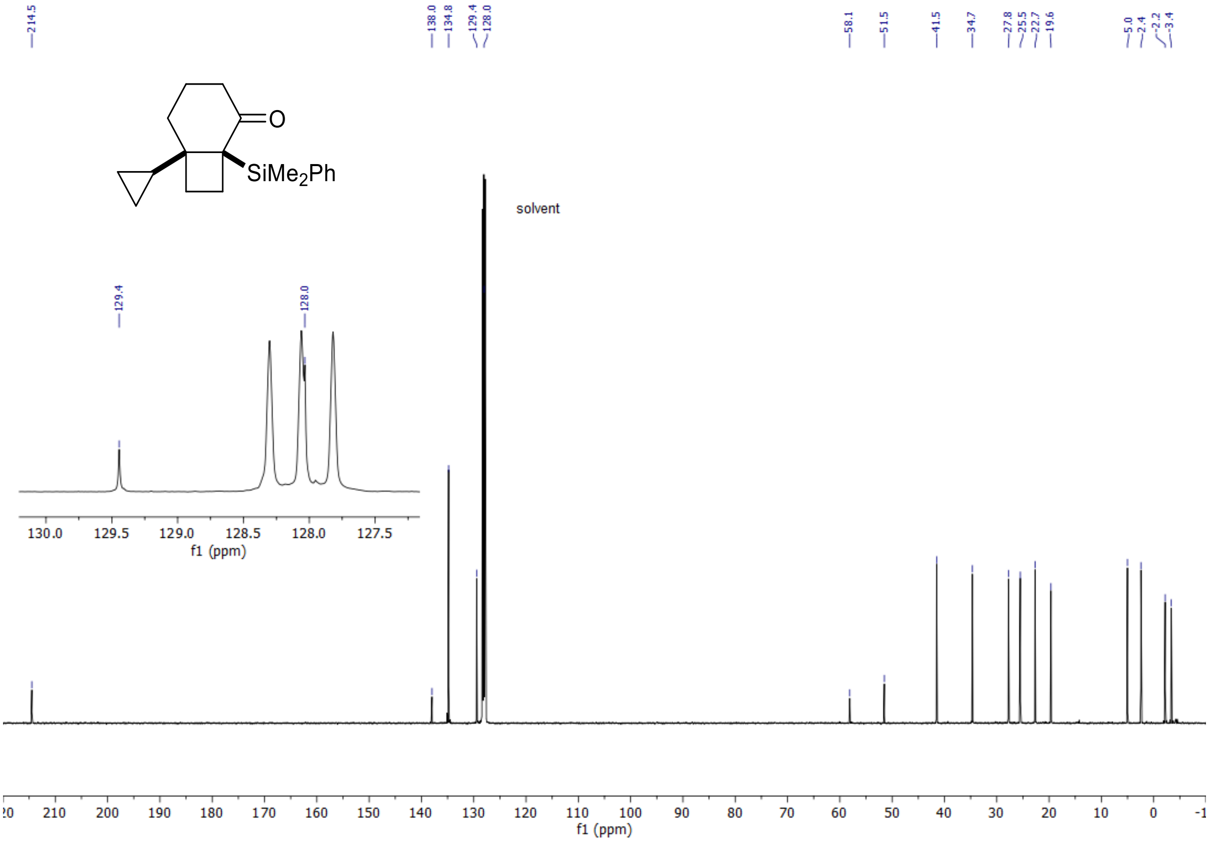

^{1}H NMR (500 MHz, $CDCl_3$) of **S2f**:

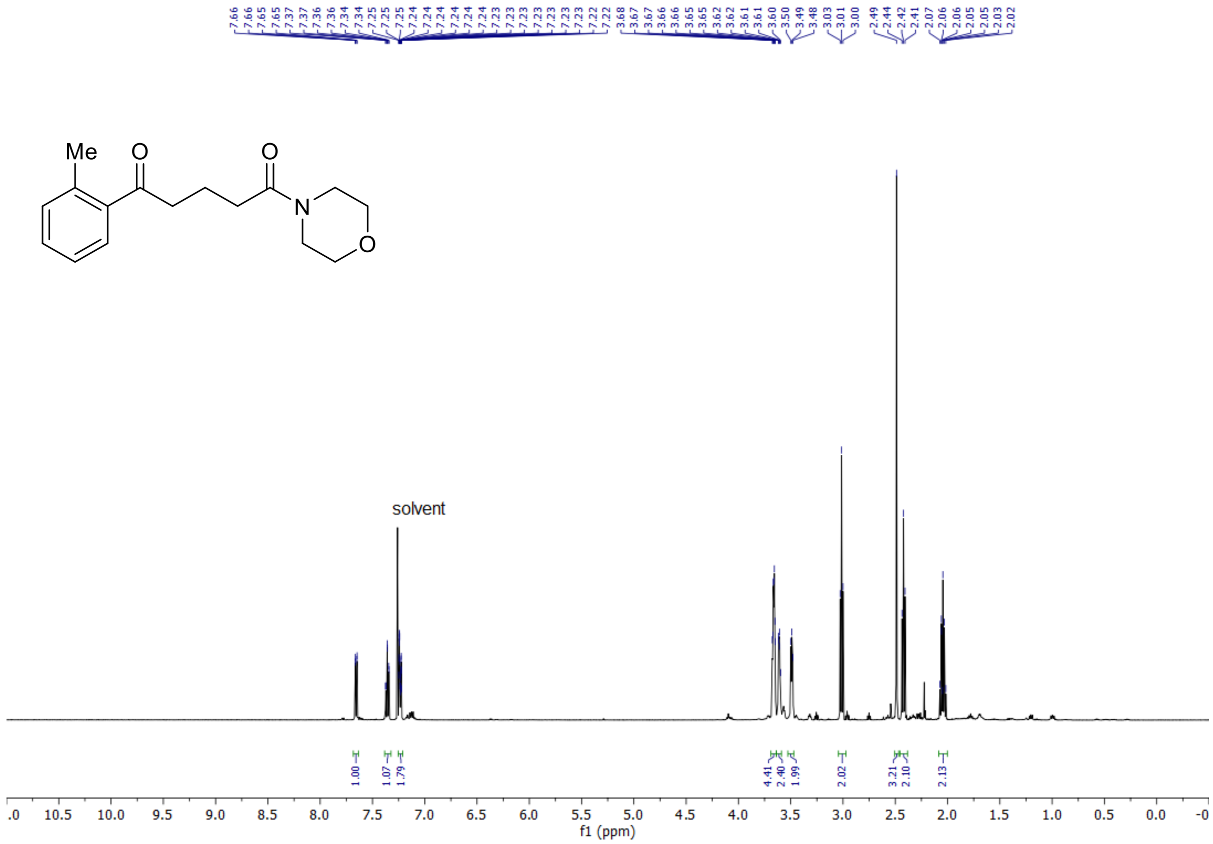

^{13}C NMR (126 MHz, $CDCl_3$) of **S2f**:

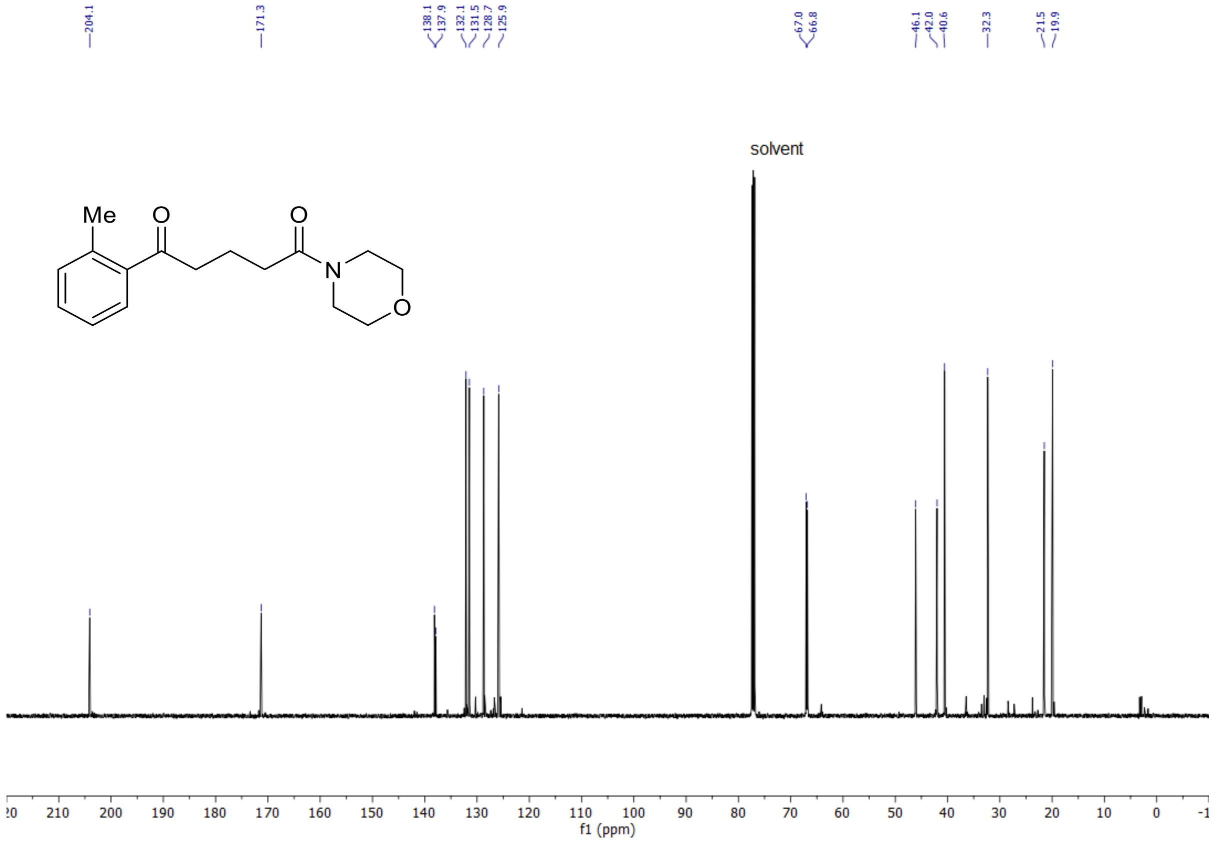

^{1}H NMR (500 MHz, CD_2Cl_2) of **1f**:

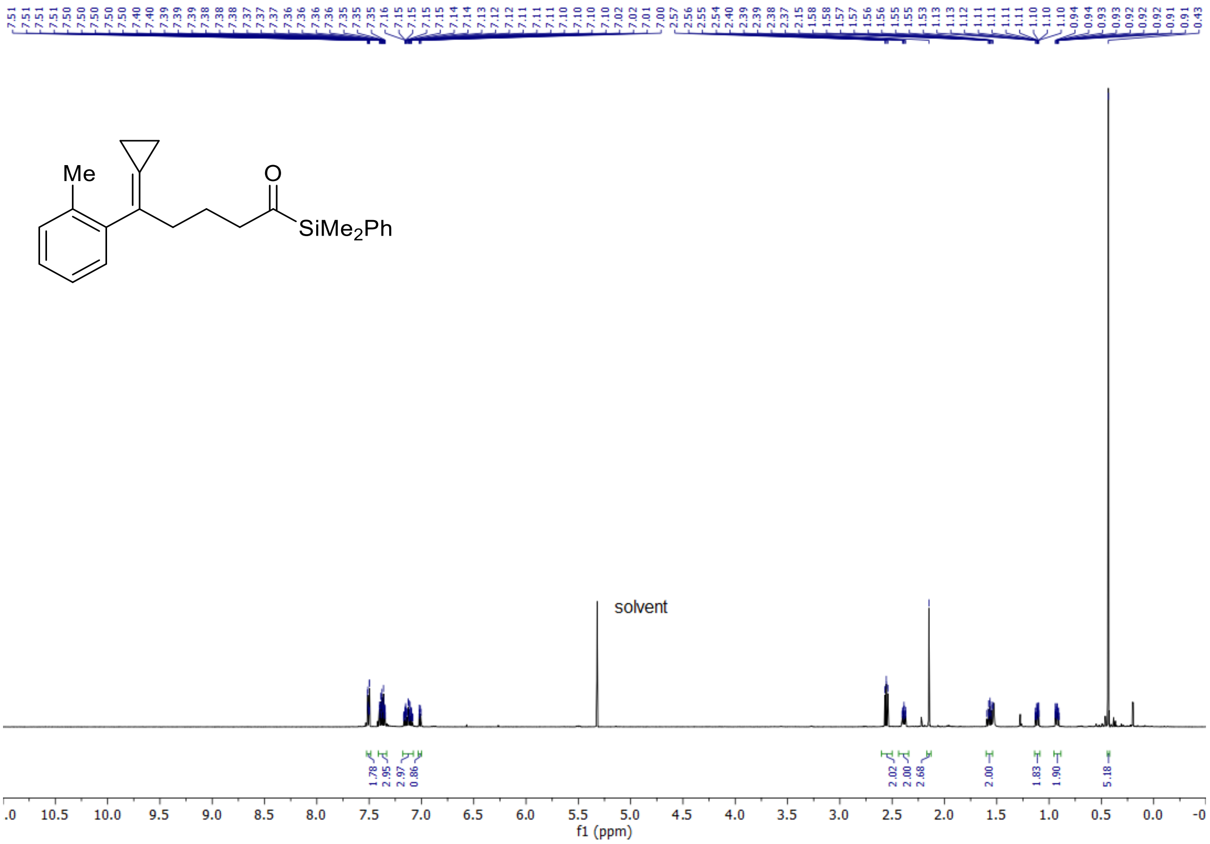

^{13}C NMR (126 MHz, CD_2Cl_2) of **1f**:

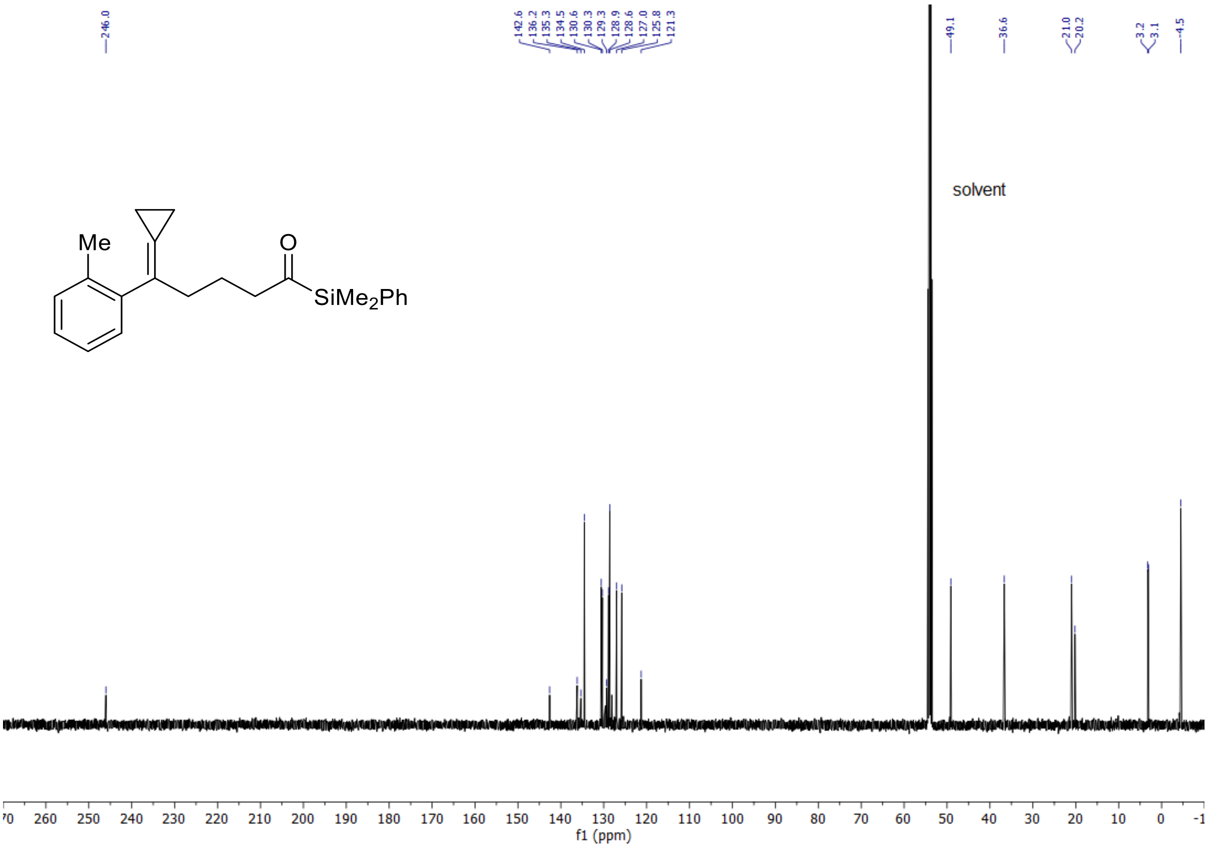

^{1}H NMR (400 MHz, C_6D_6) of **2f**:

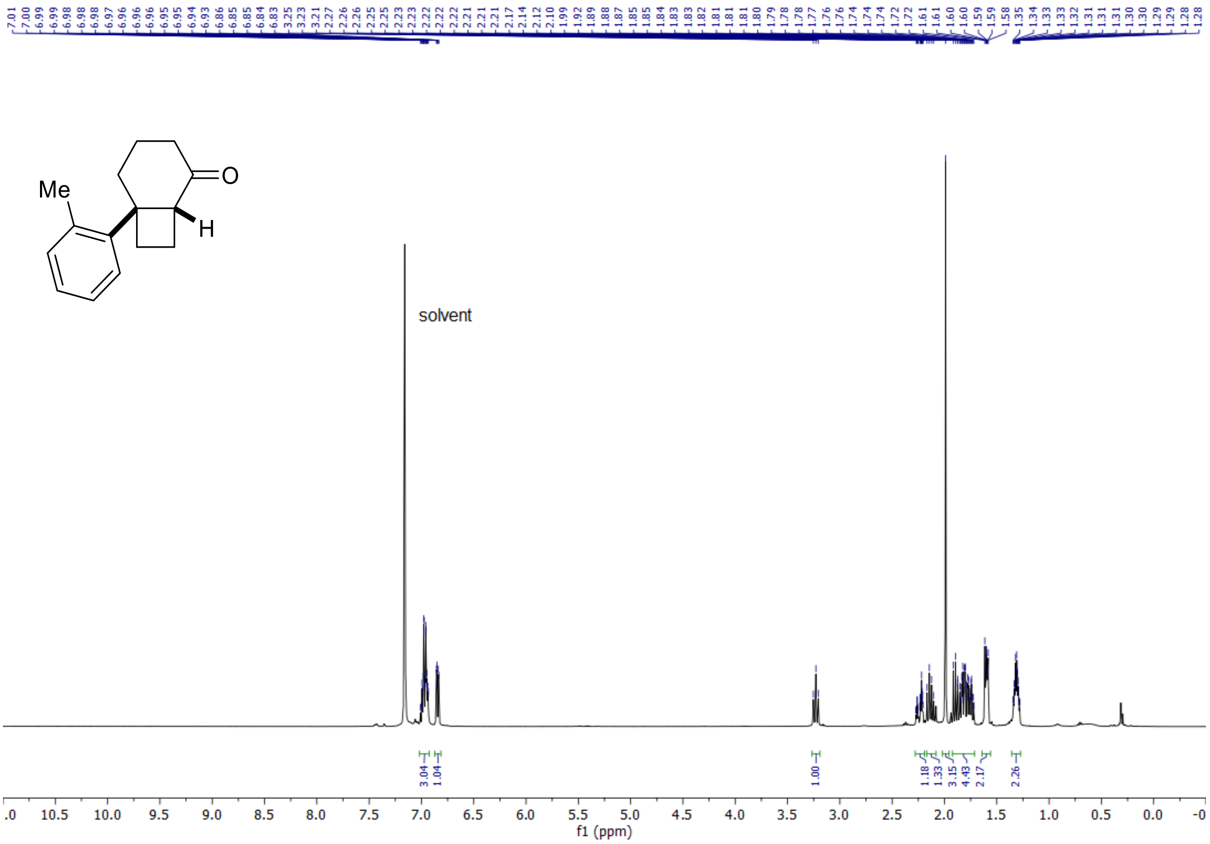

^{13}C NMR (101 MHz, C_6D_6) of **2f**:

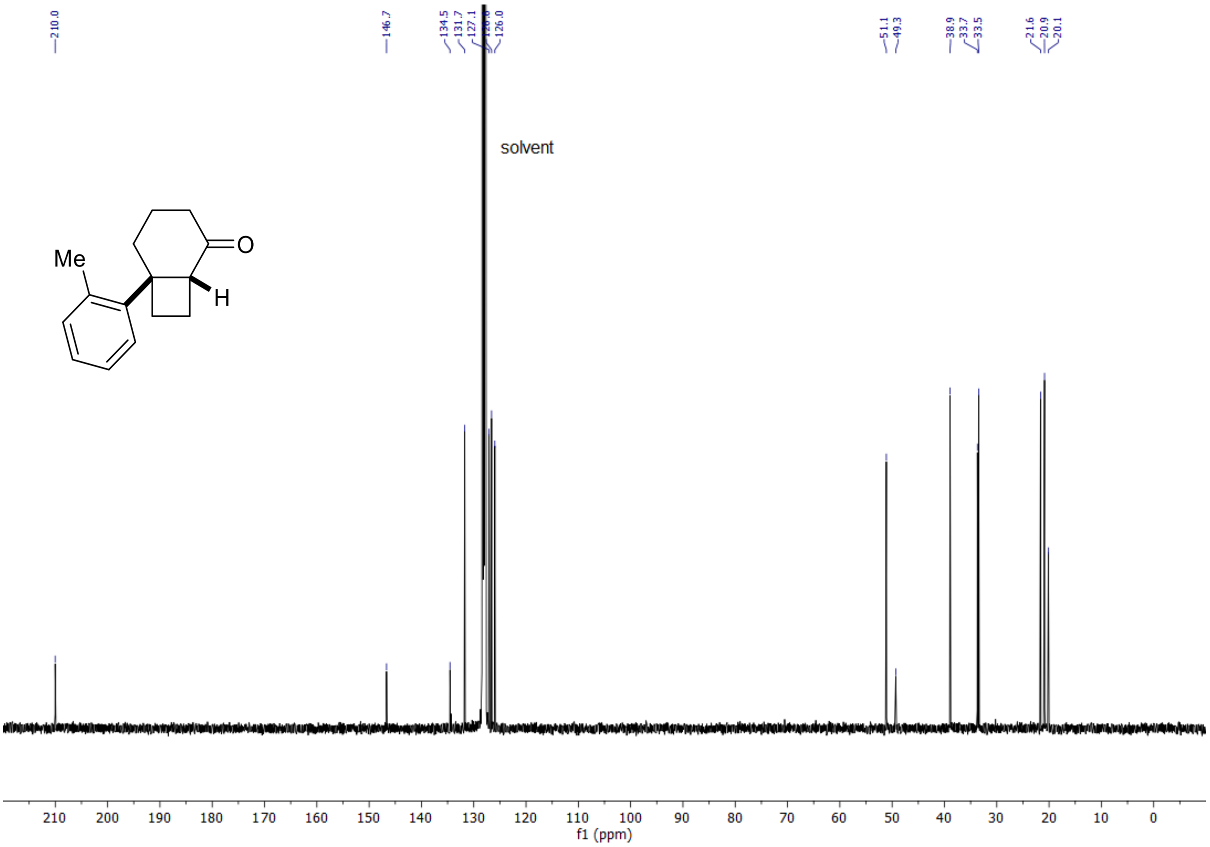

^{1}H NMR (400 MHz, $CDCl_3$) of **S2g**:

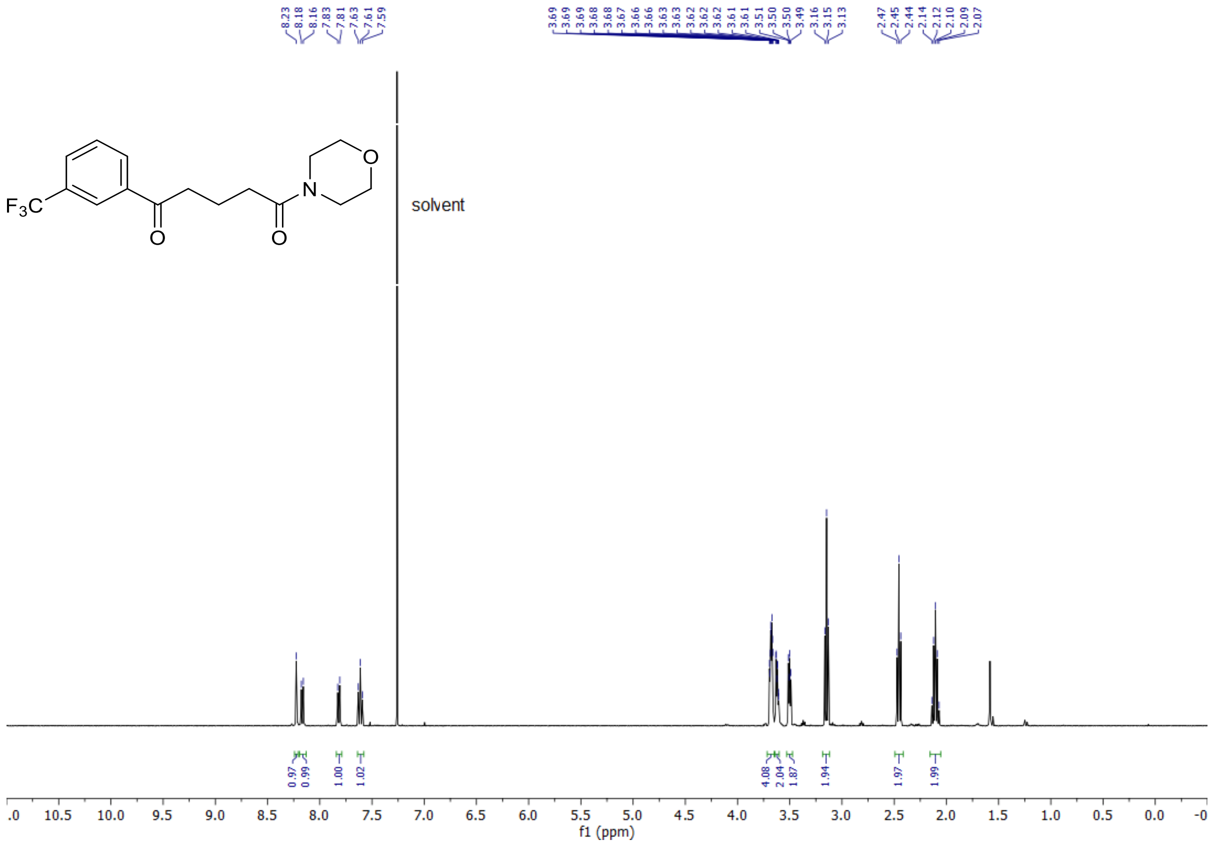

^{13}C NMR (101 MHz, $CDCl_3$) of **S2g**:

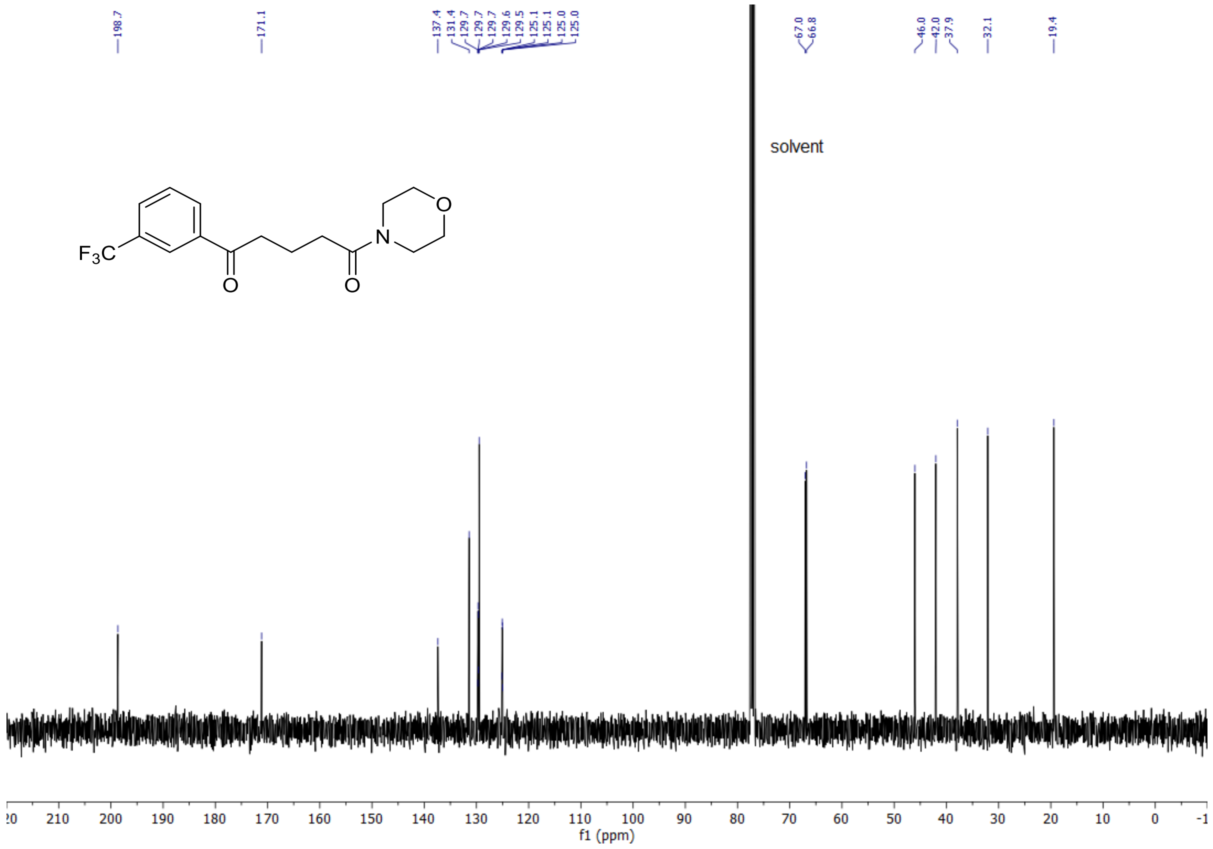

^{19}F NMR (376 MHz, $CDCl_3$) of **S2g**:

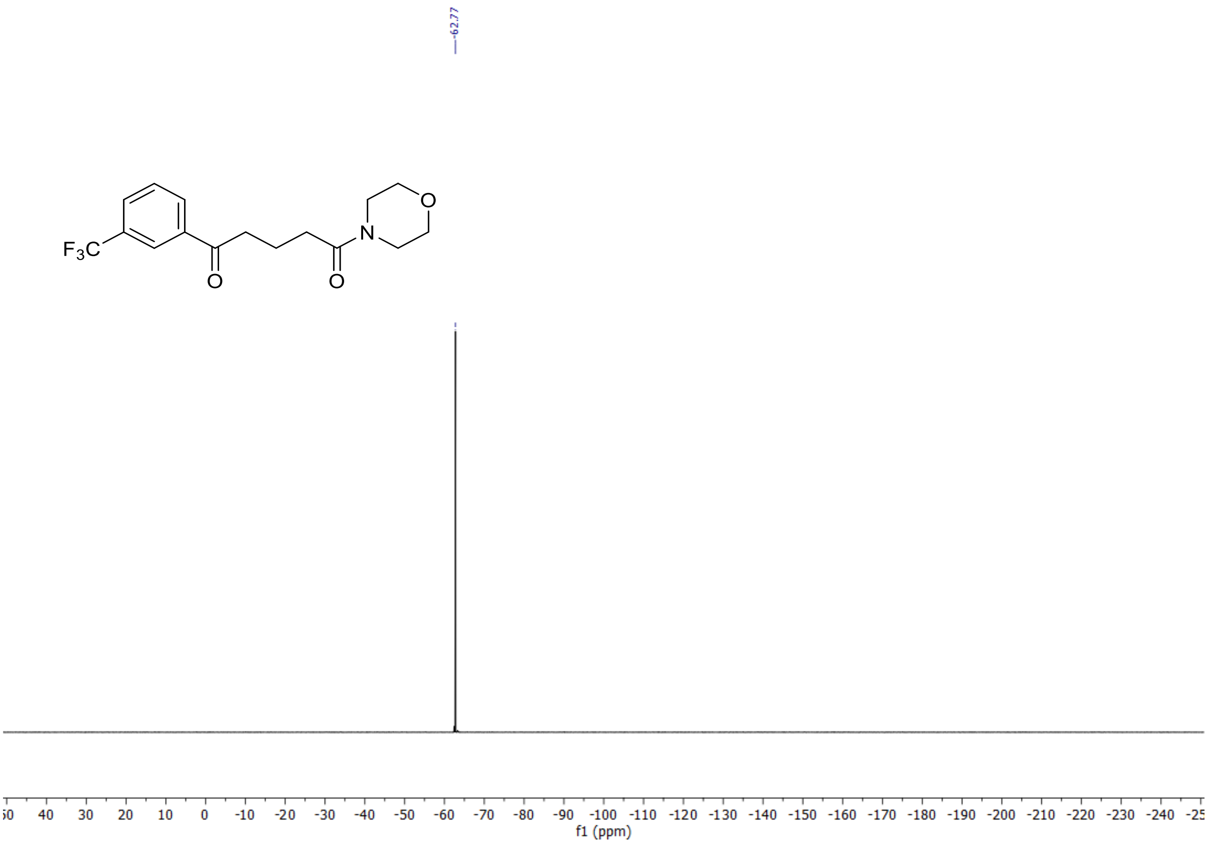

^{1}H NMR (500 MHz, C_6D_6) of **1g**:

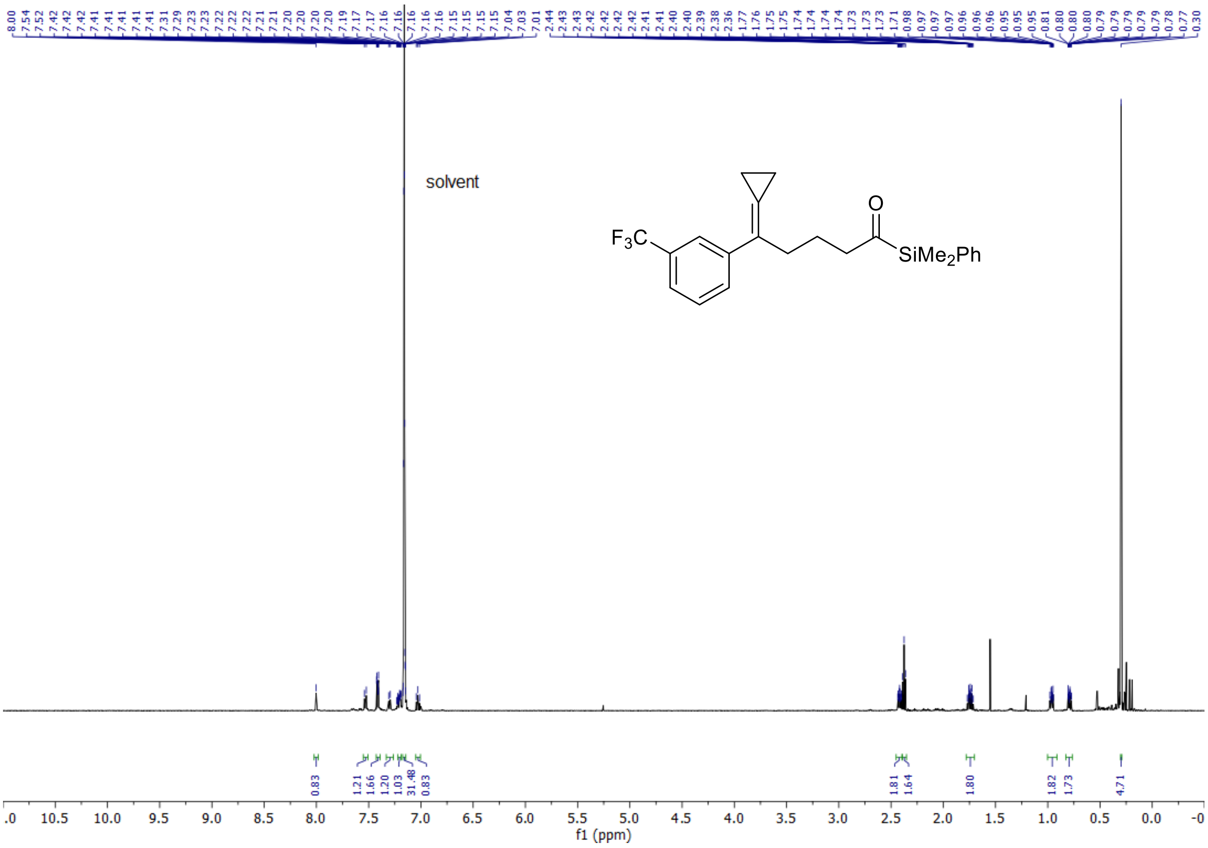

^{13}C NMR (126 MHz, C_6D_6) of **1g**:

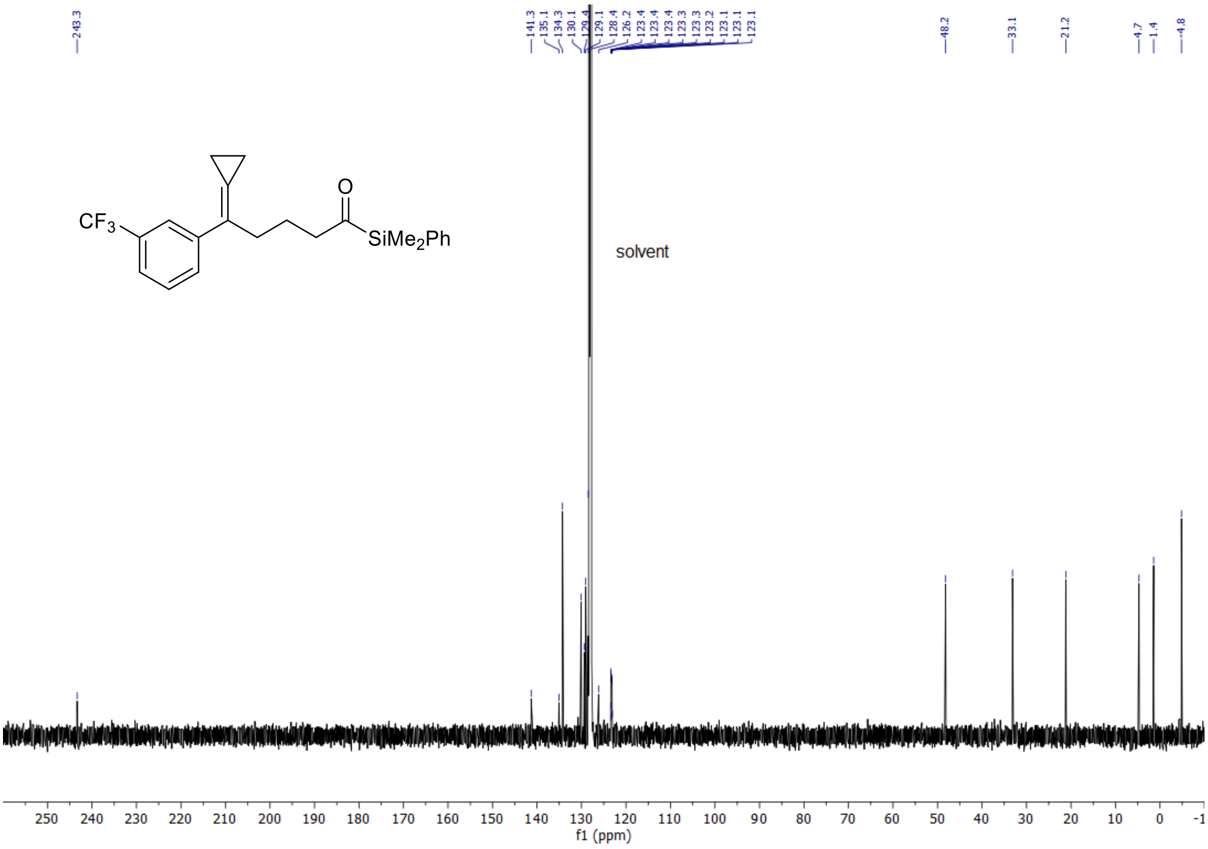

^{19}F NMR (376 MHz, $CDCl_3$) of **1g**:

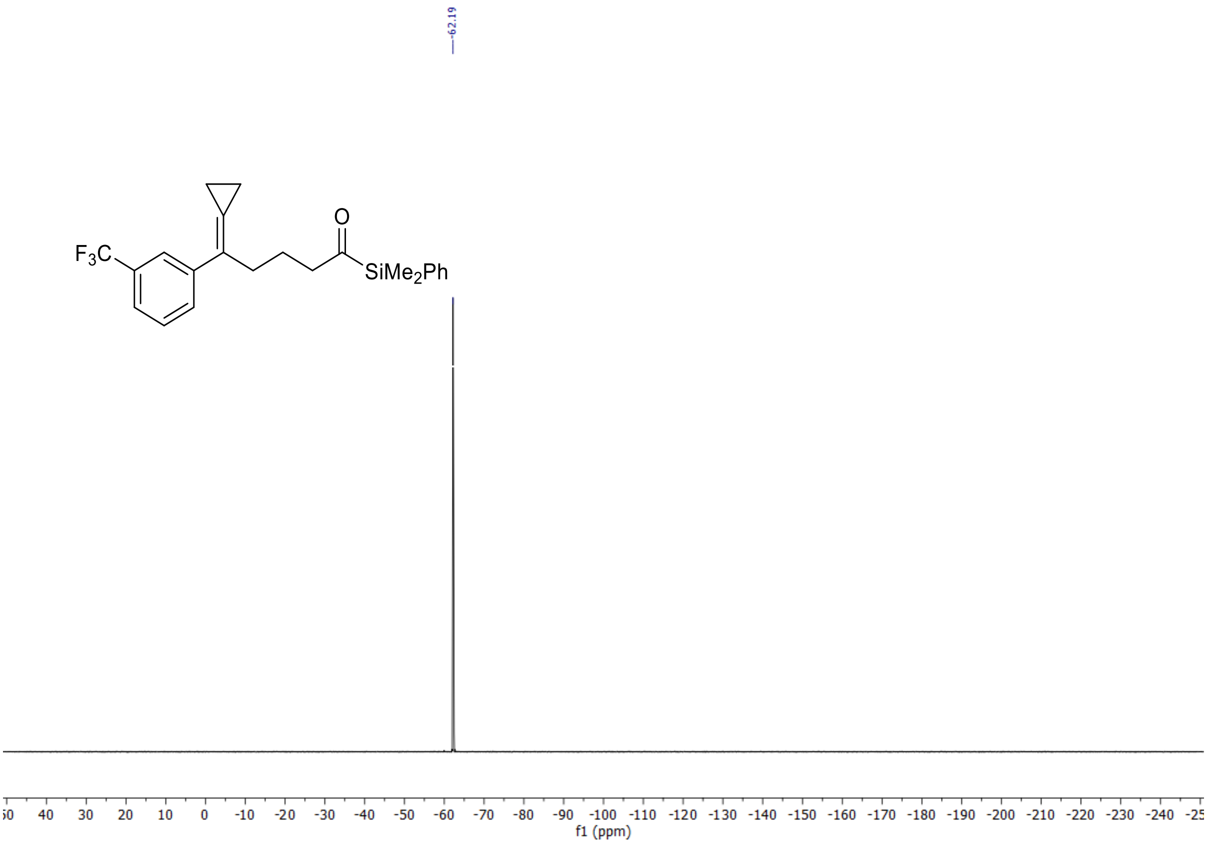

^{1}H NMR (400 MHz, C_6D_6) of **2g**:

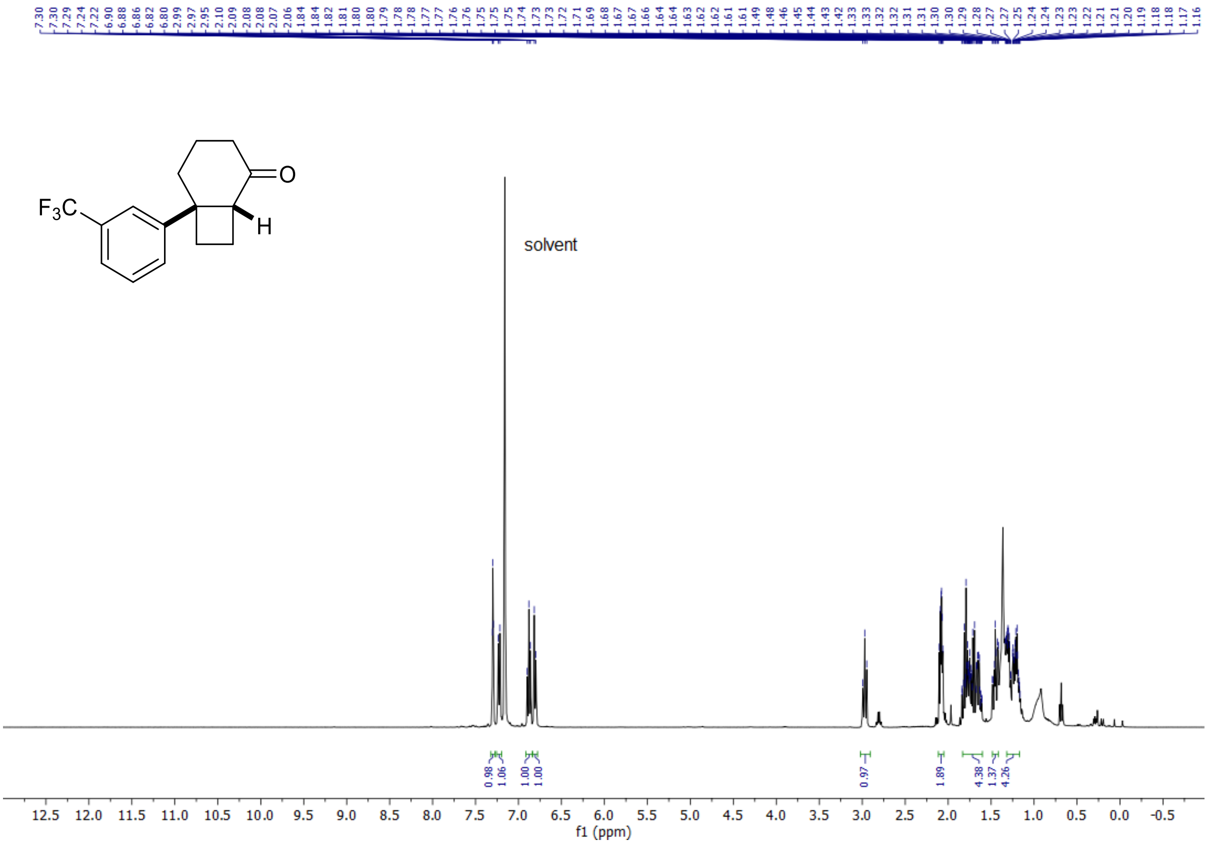

^{13}C NMR (101 MHz, C_6D_6) of **2g**:

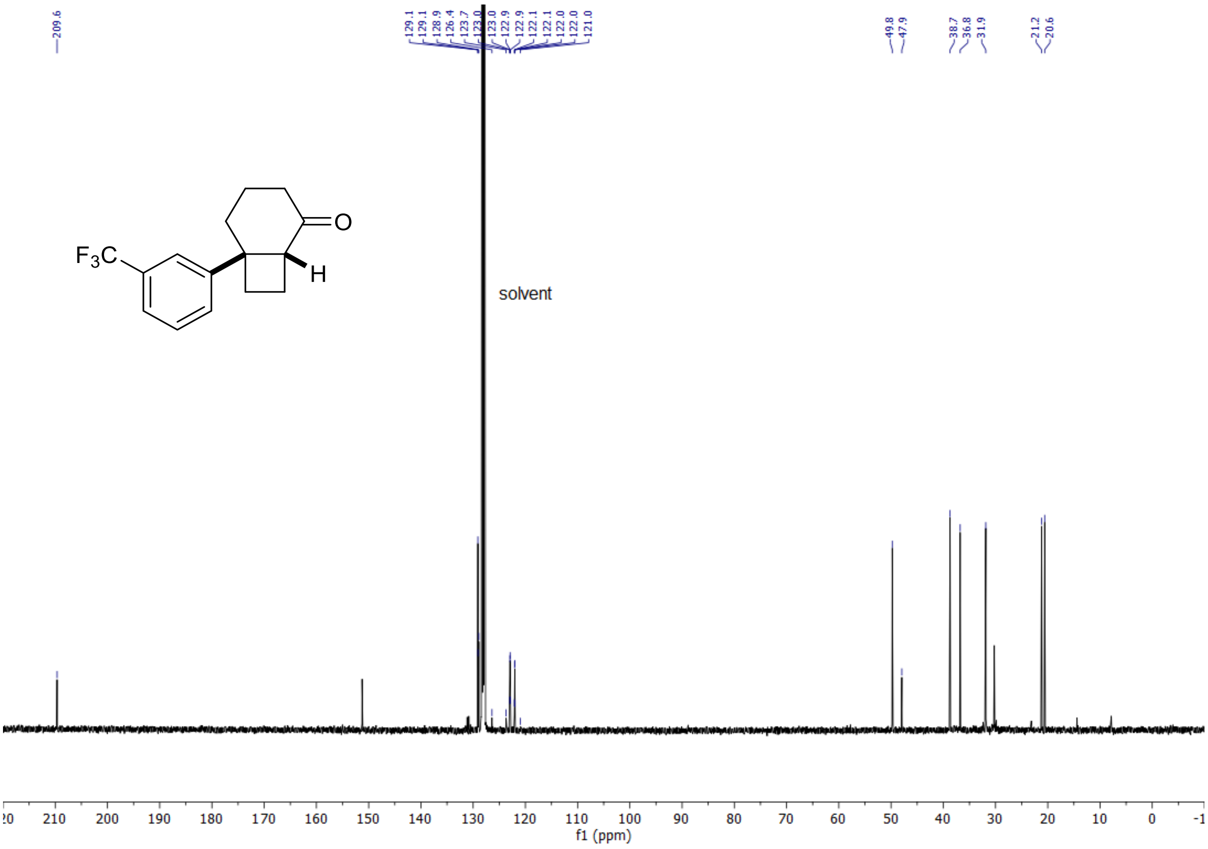

^{19}F NMR (377 MHz, C_6D_6) of **2g**:

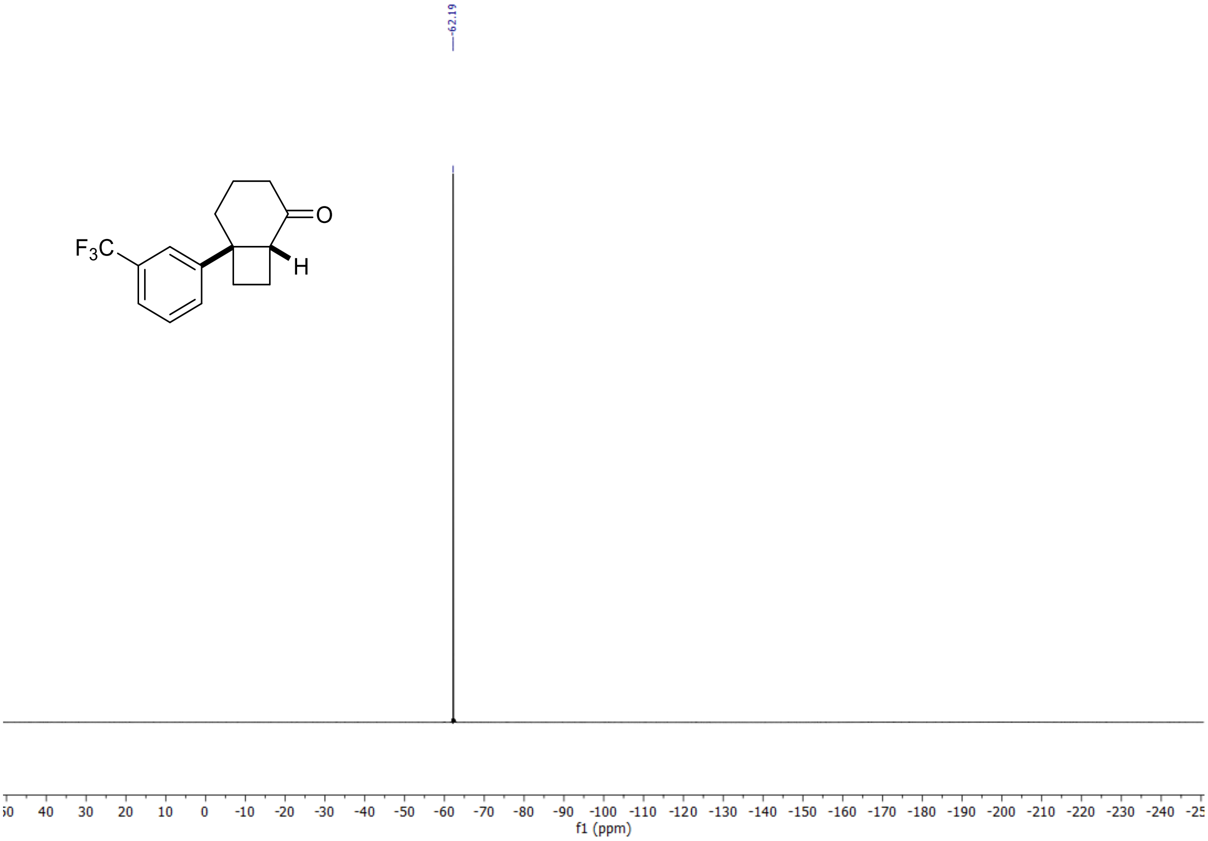

^{1}H NMR (500 MHz, $CDCl_3$) of **S2h**:

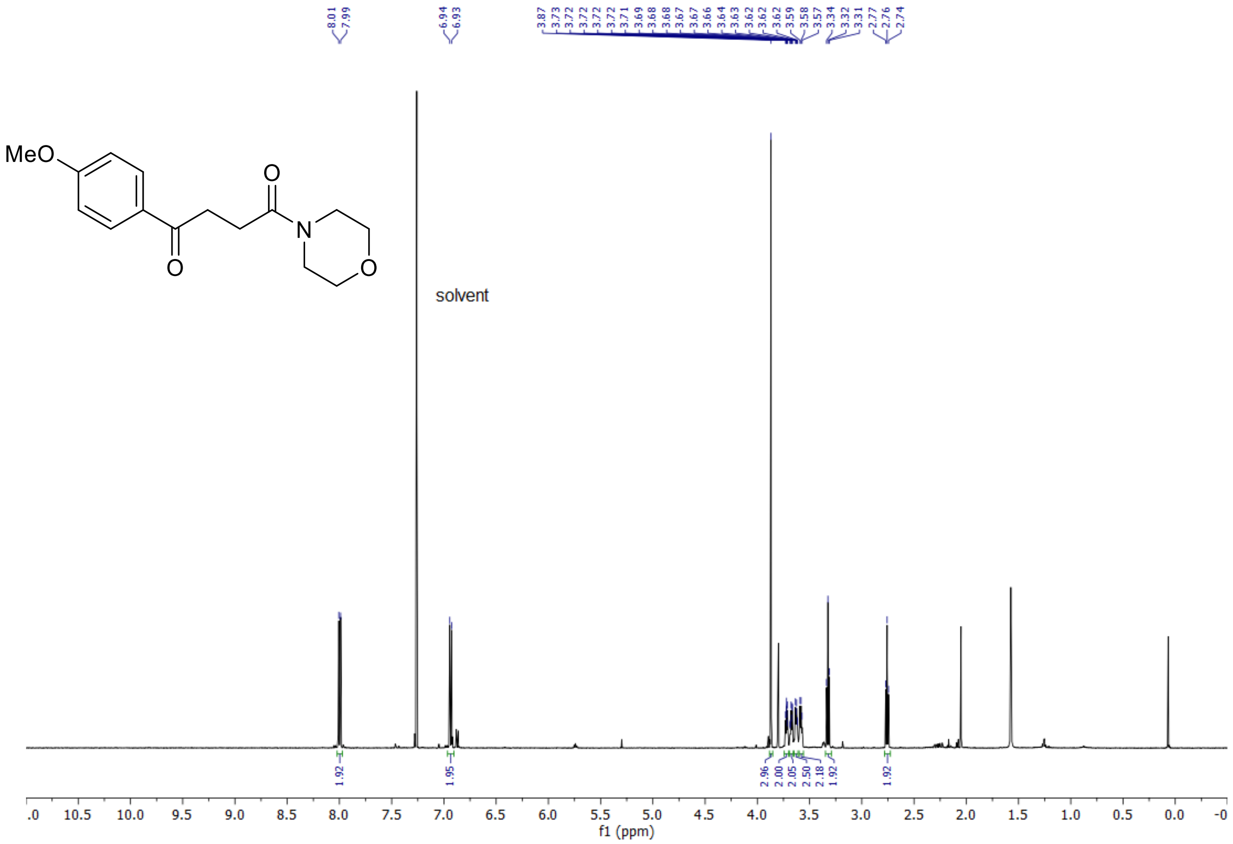

^{13}C NMR (126 MHz, $CDCl_3$) of **S2h**:

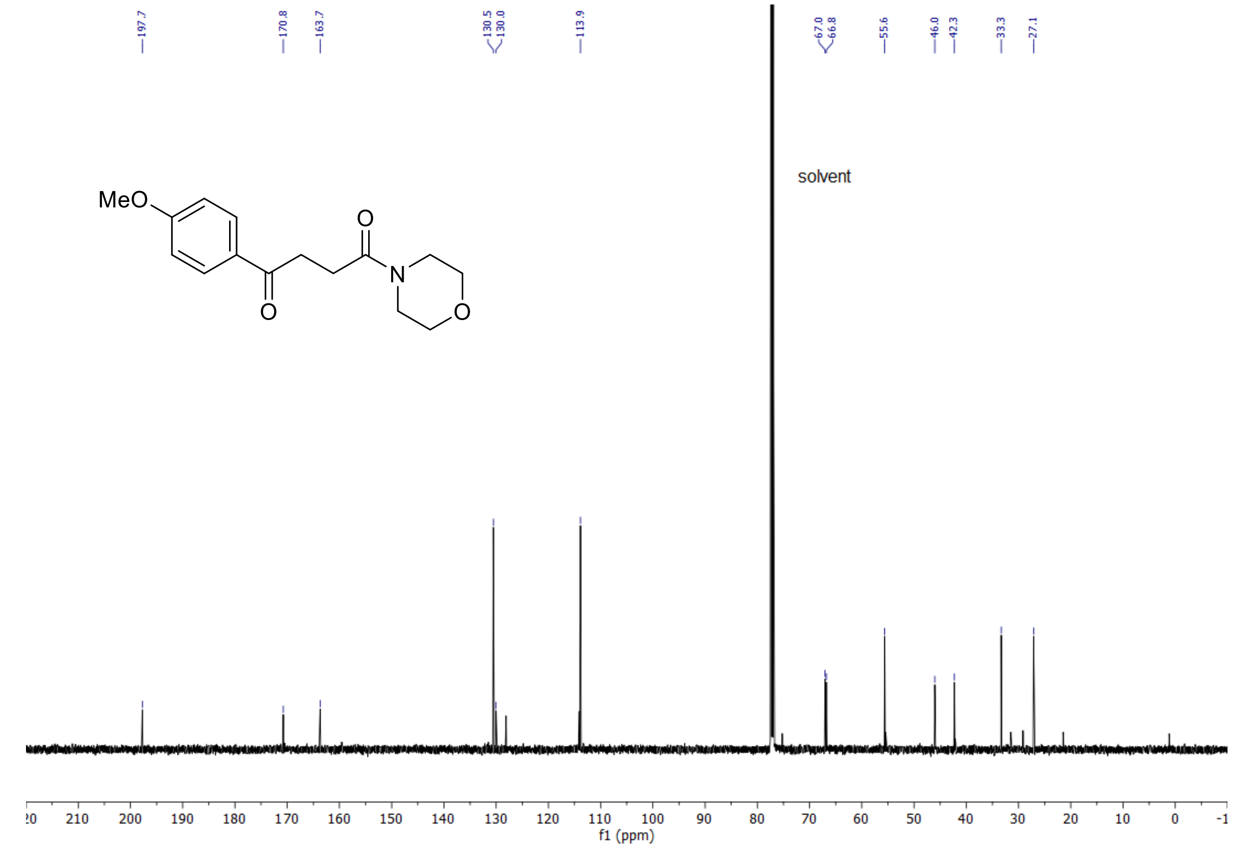

^{1}H NMR (500 MHz, C_6D_6) of **1h**:

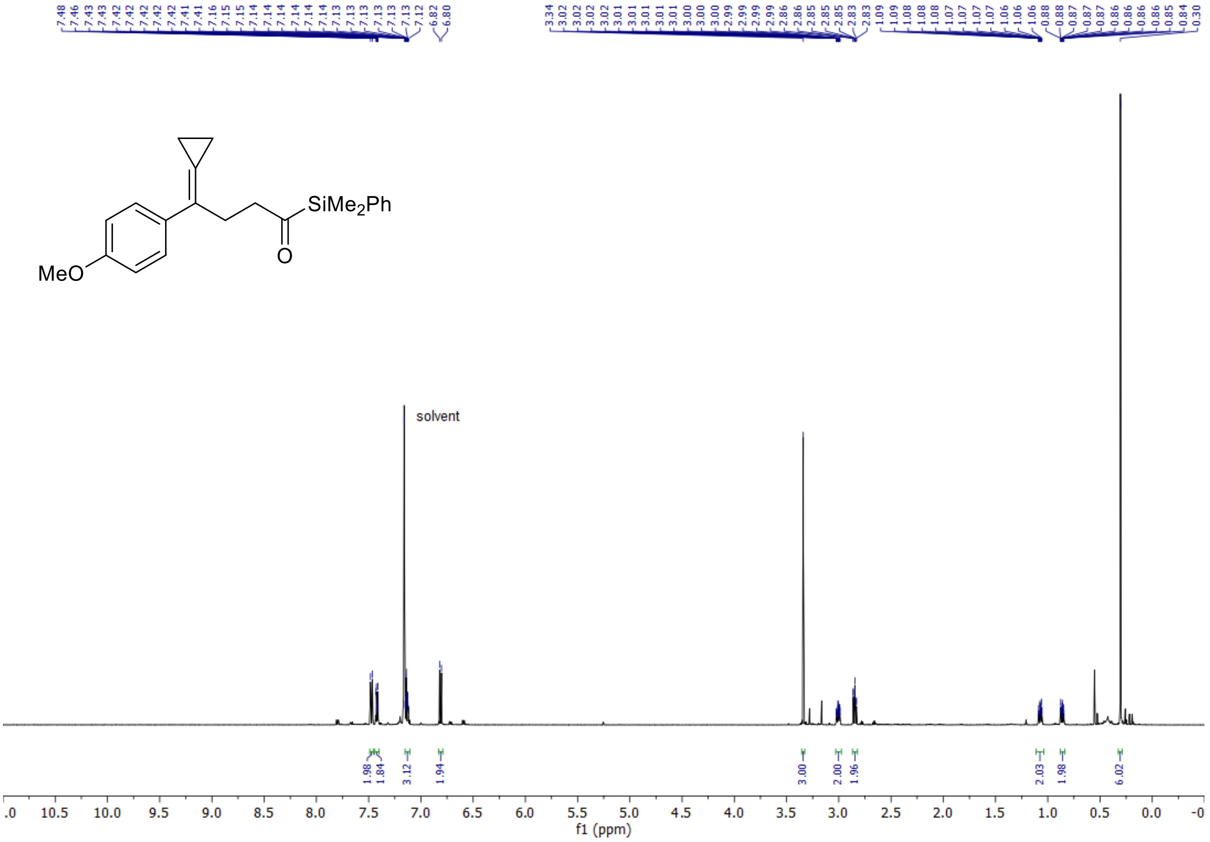

^{13}C NMR (126 MHz, C_6D_6) of **1h**:

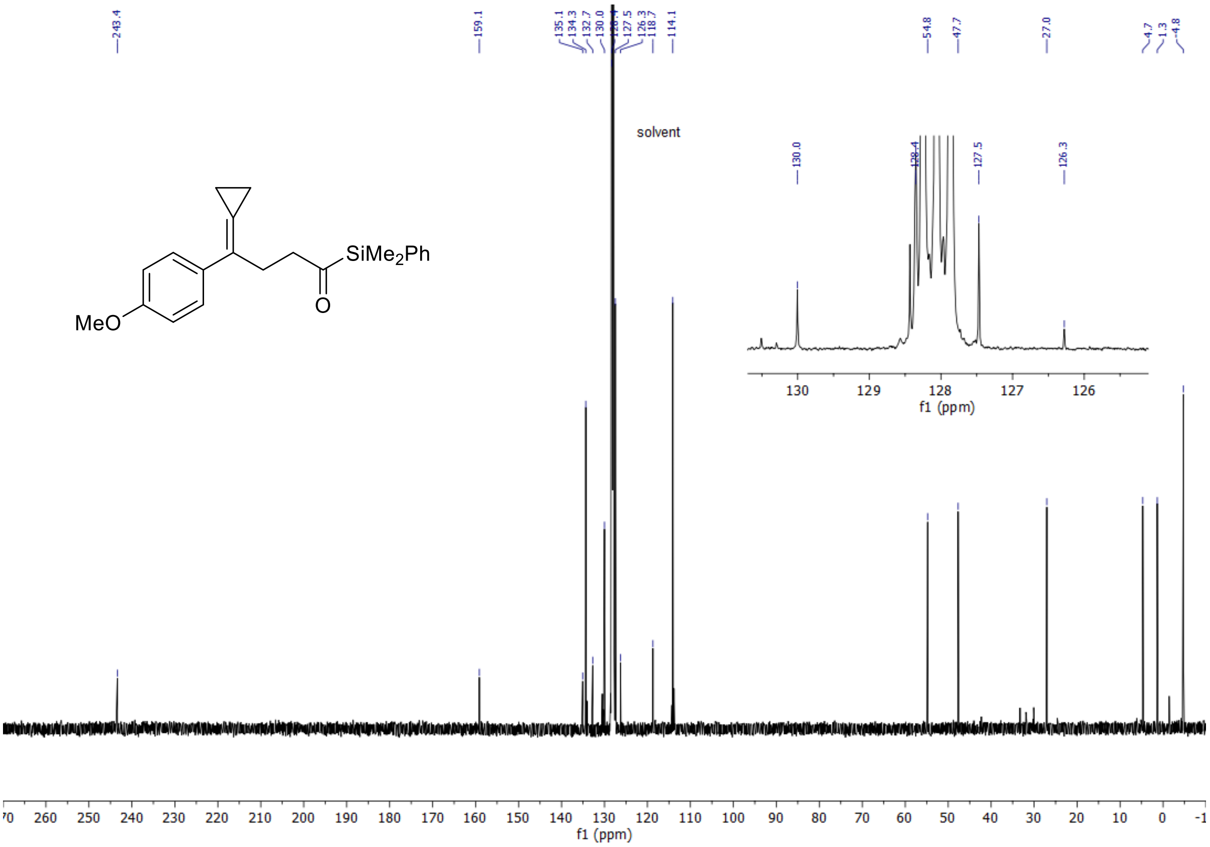

^1H NMR (500 MHz, C_6D_6) of **2h**:

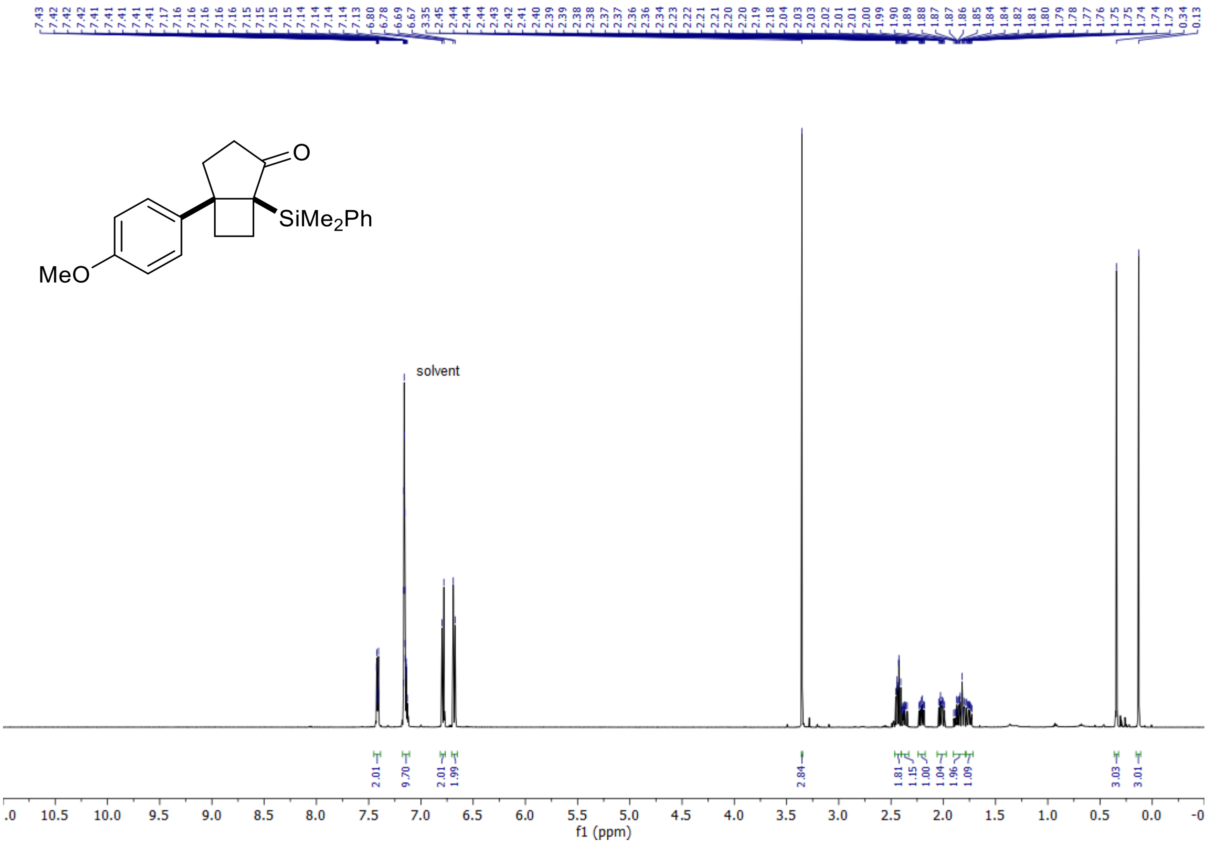

^{13}C NMR (126 MHz, C_6D_6) of **2h**:

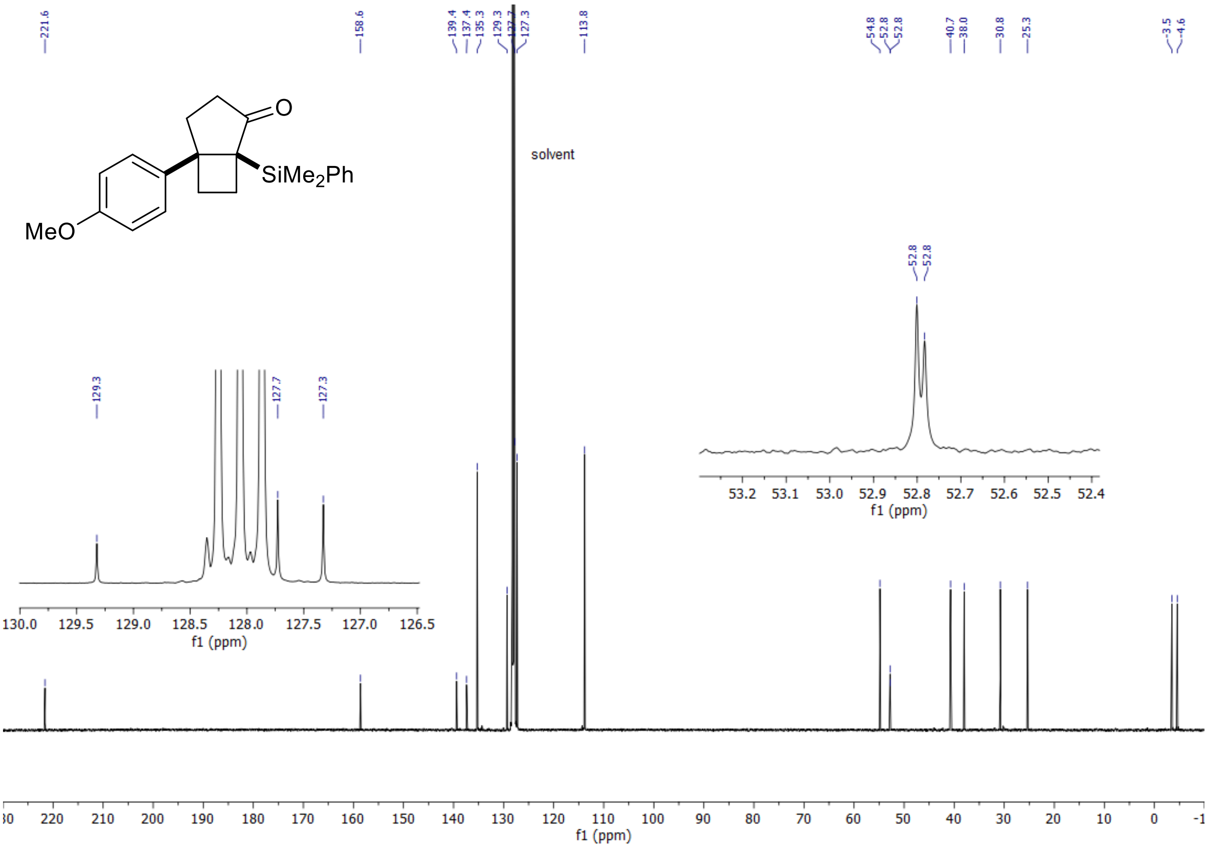

^1H NMR (400 MHz, $CDCl_3$) of **S1i**

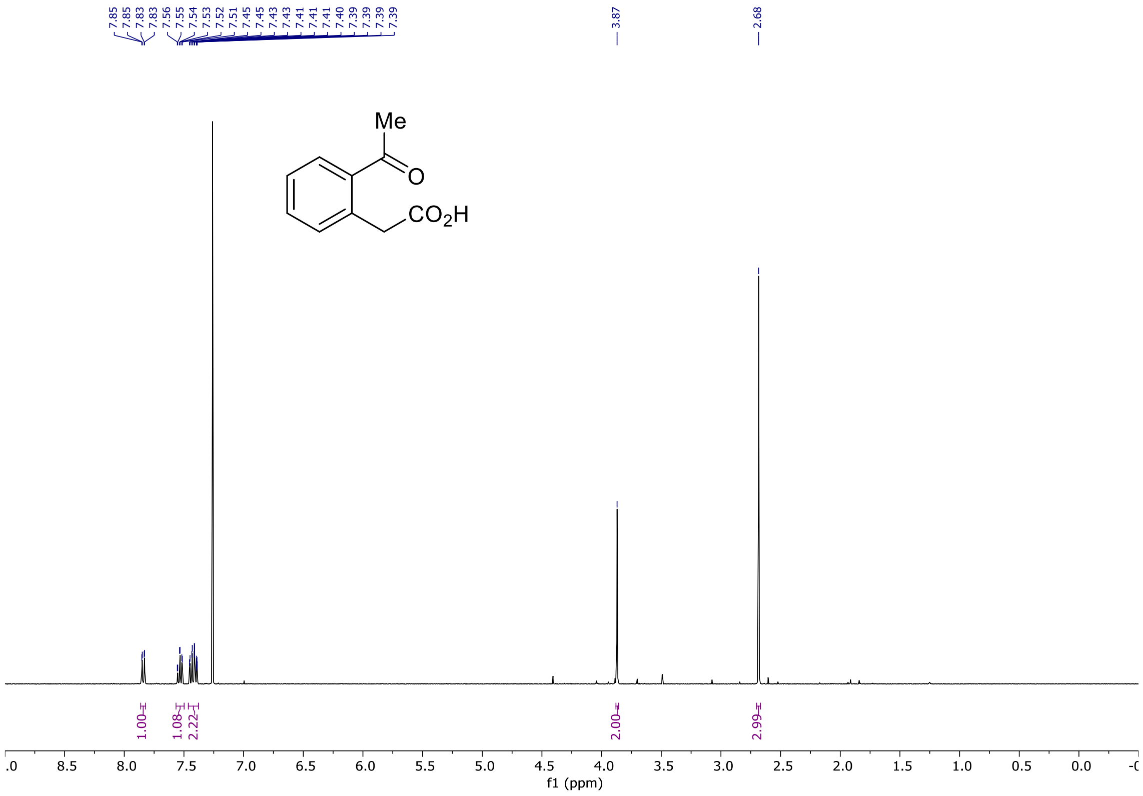

^{1}H NMR (500 MHz, $CDCl_3$) of **S2i**

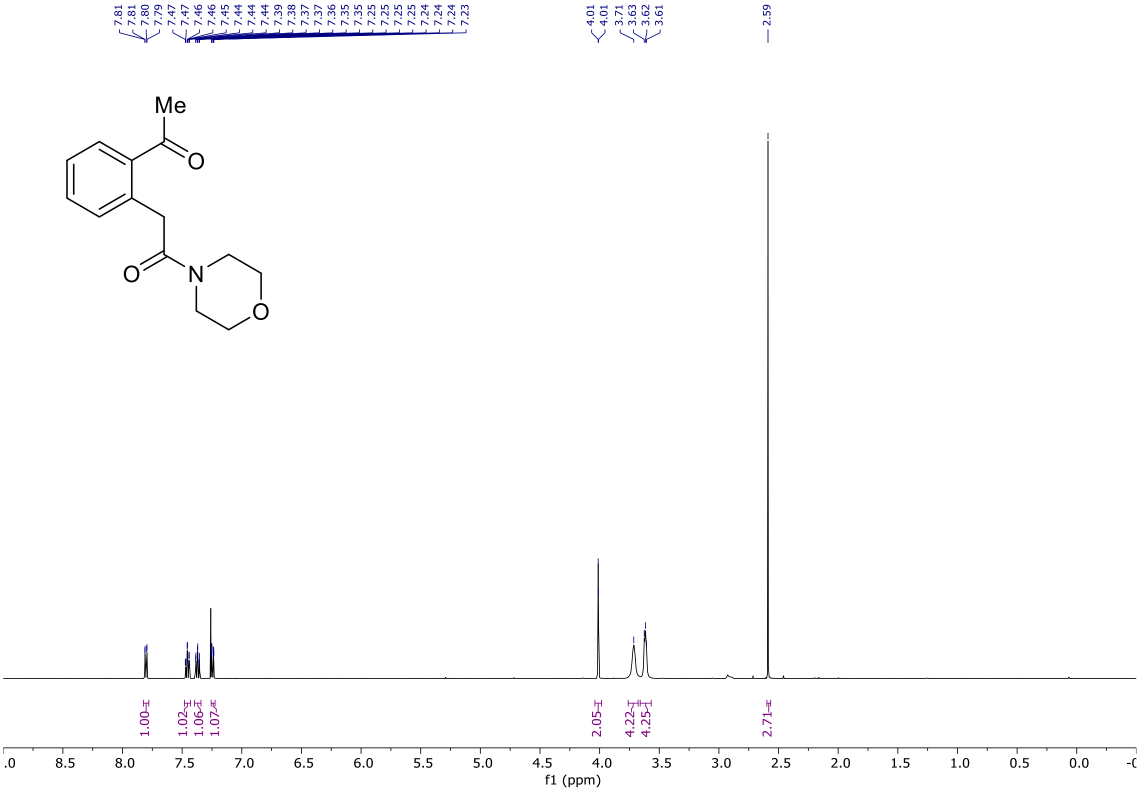

^{13}C NMR (126 MHz, $CDCl_3$) of **S2i**

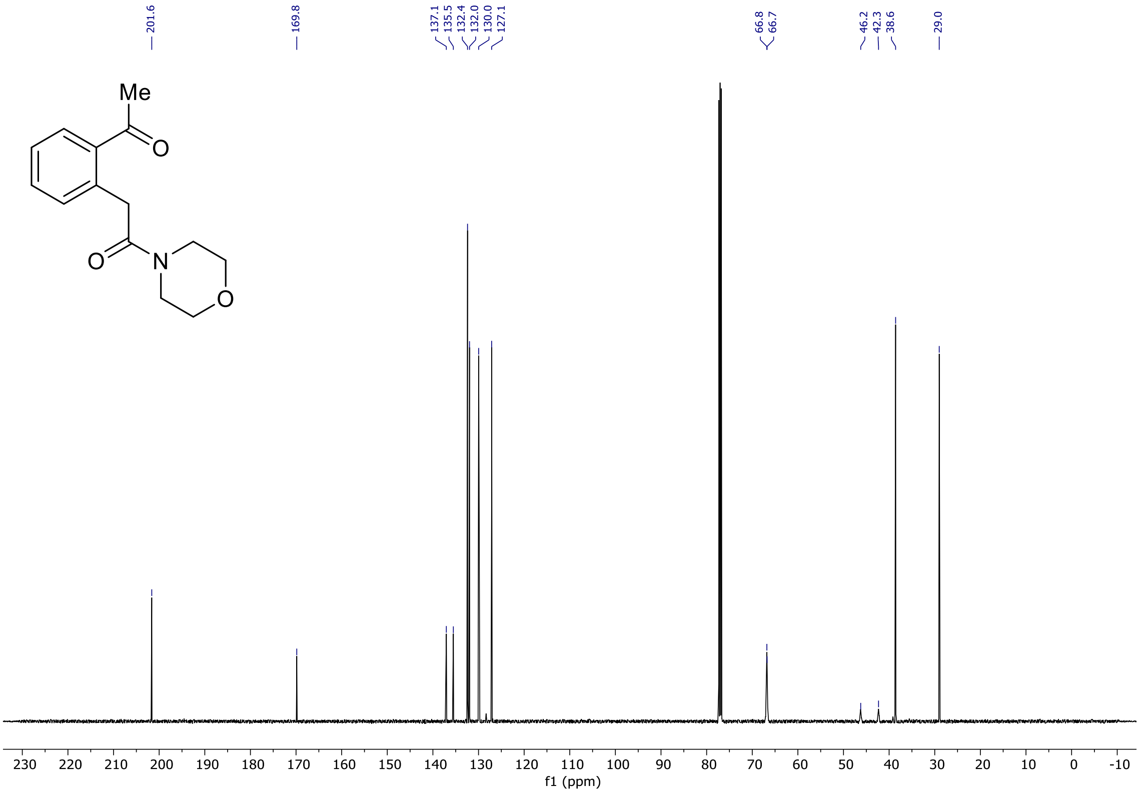

^{1}H NMR (500 MHz, C_6D_6) of **S3i**

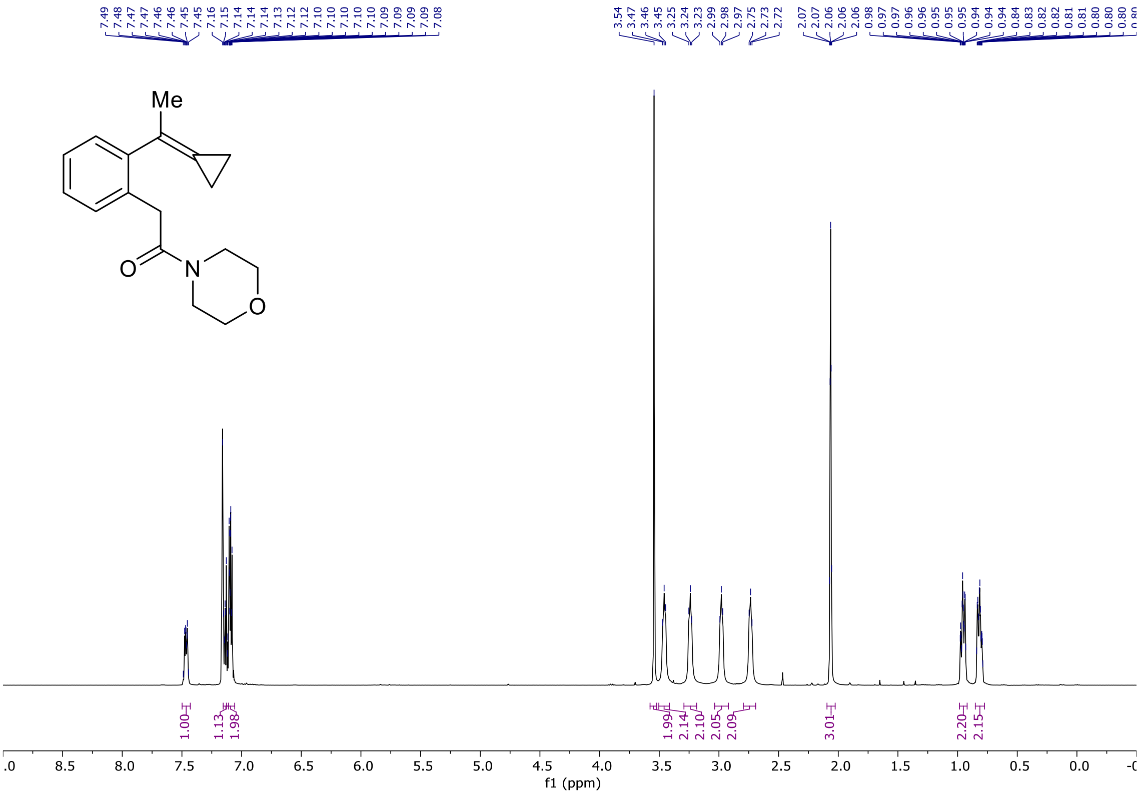

^{13}C NMR (126 MHz, C_6D_6) of **S3i**

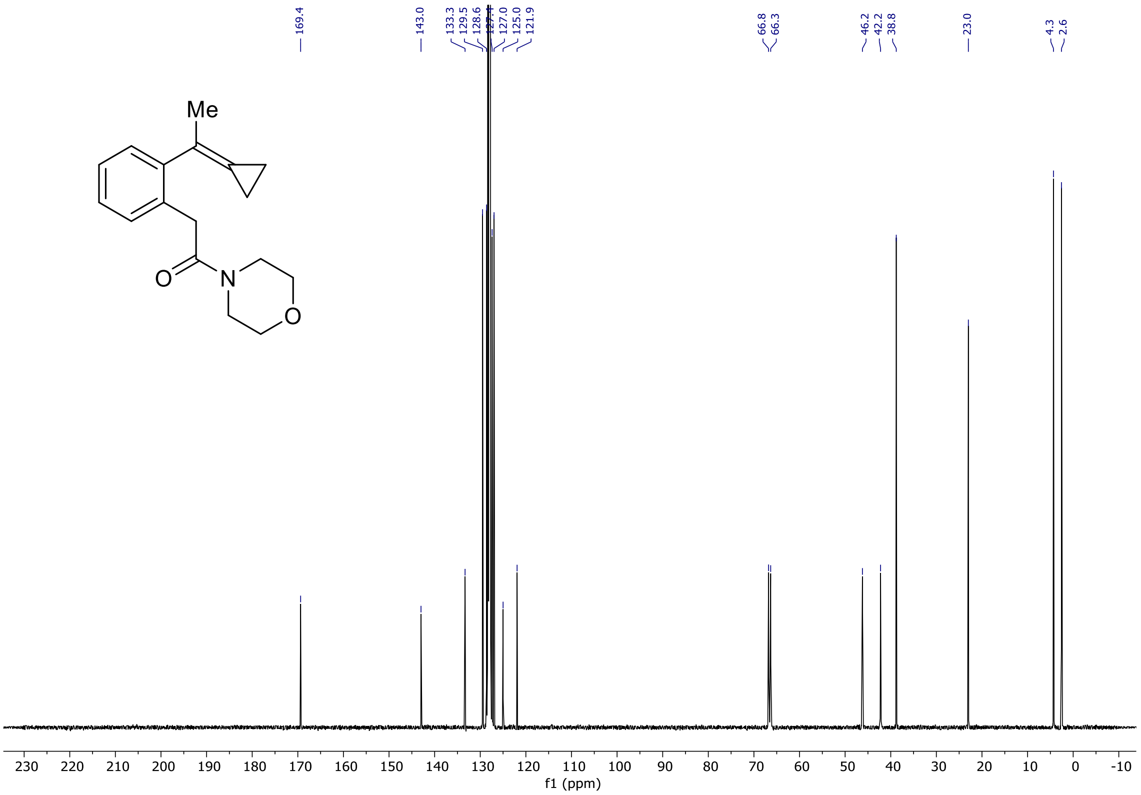

^{1}H NMR (500 MHz, C_6D_6) of **1i**

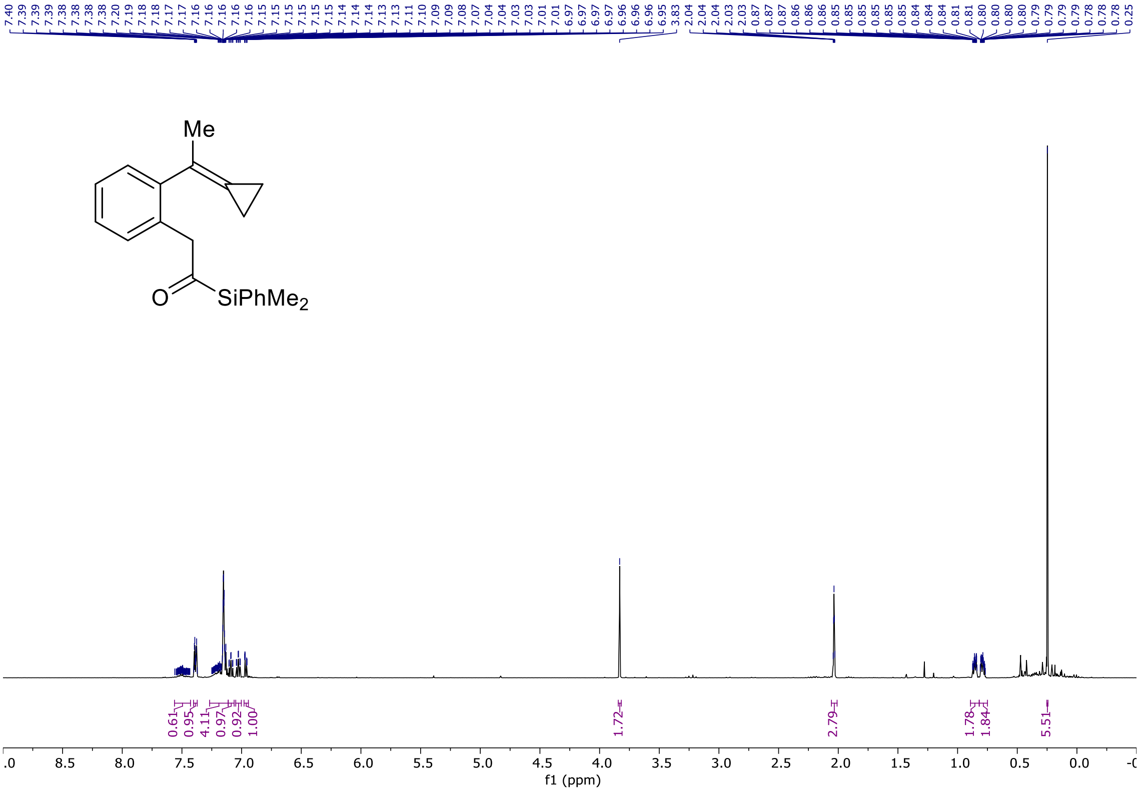

^{13}C NMR (126 MHz, C_6D_6) of **1i**

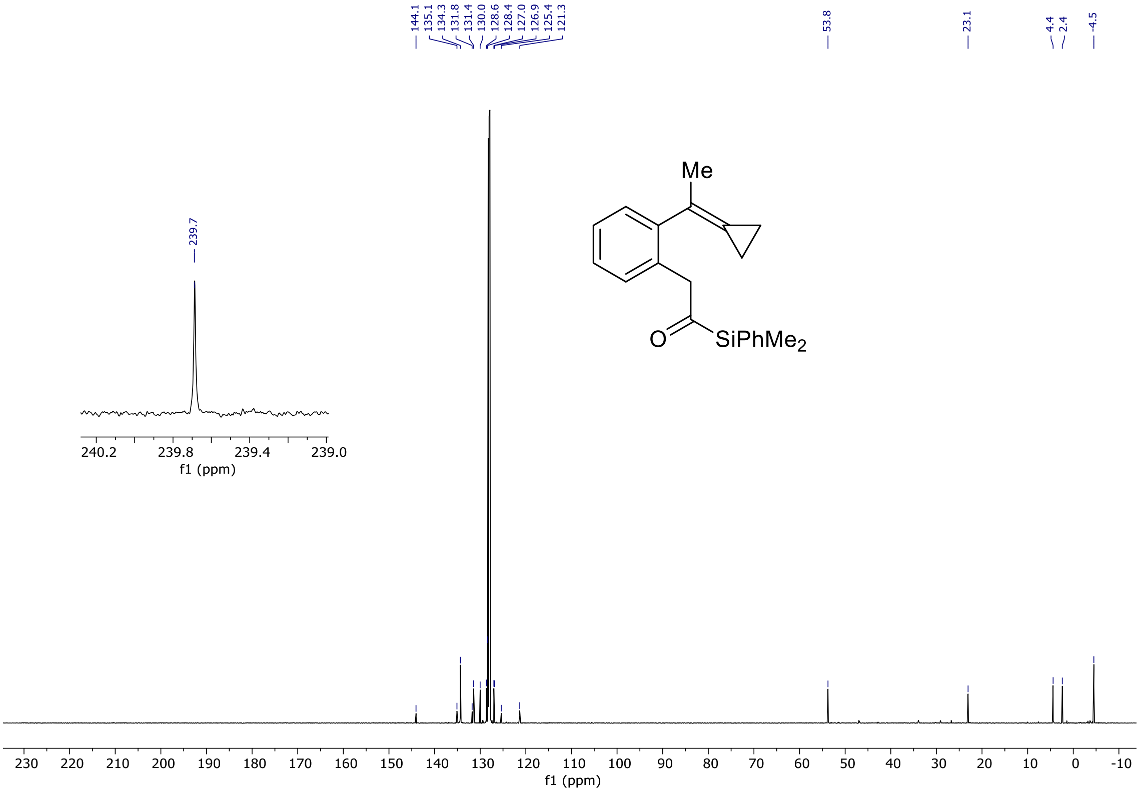

^{1}H NMR (500 MHz, C_6D_6) of **2i**

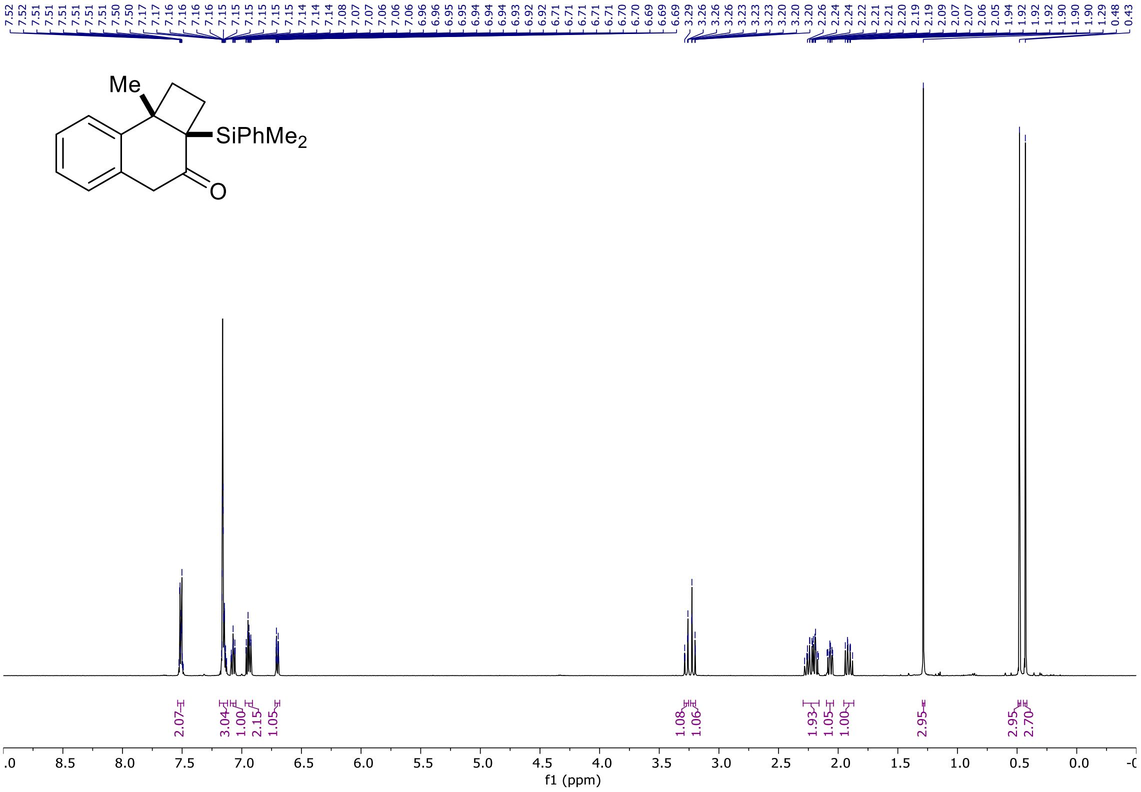

^{13}C NMR (126 MHz, C_6D_6) of **2i**

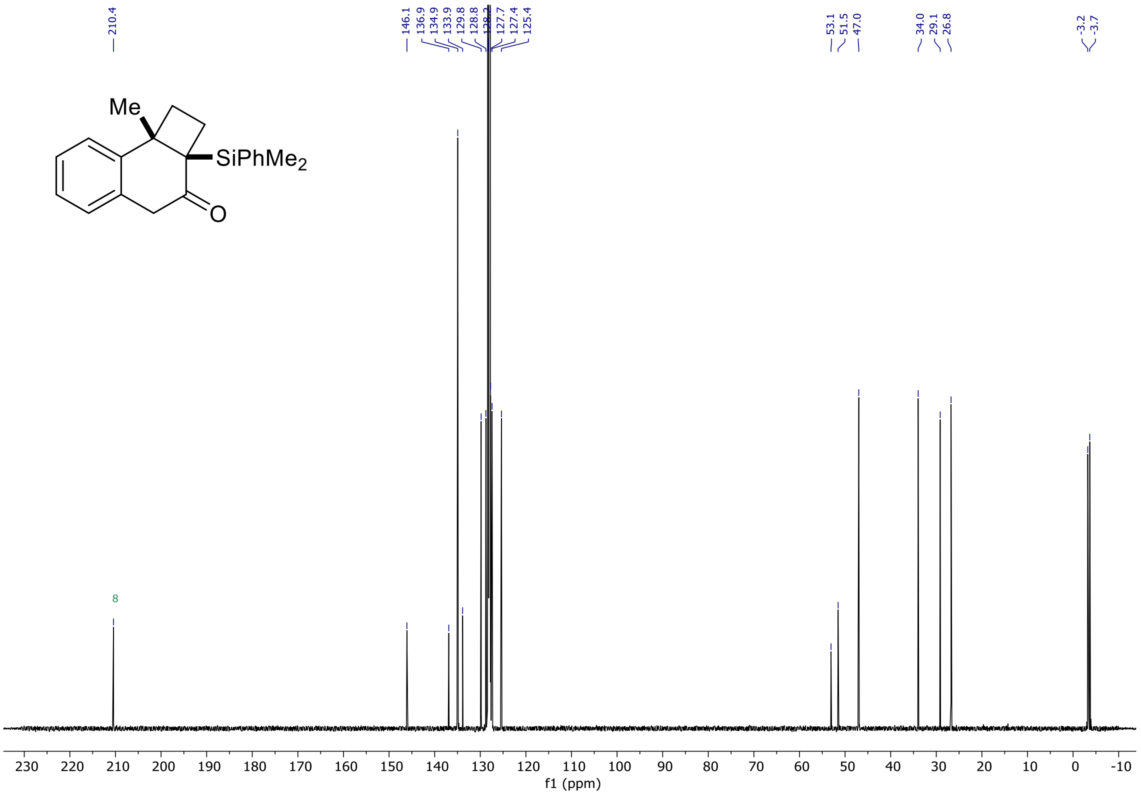

^{1}H NMR (500 MHz, $CDCl_3$) of **S2j**

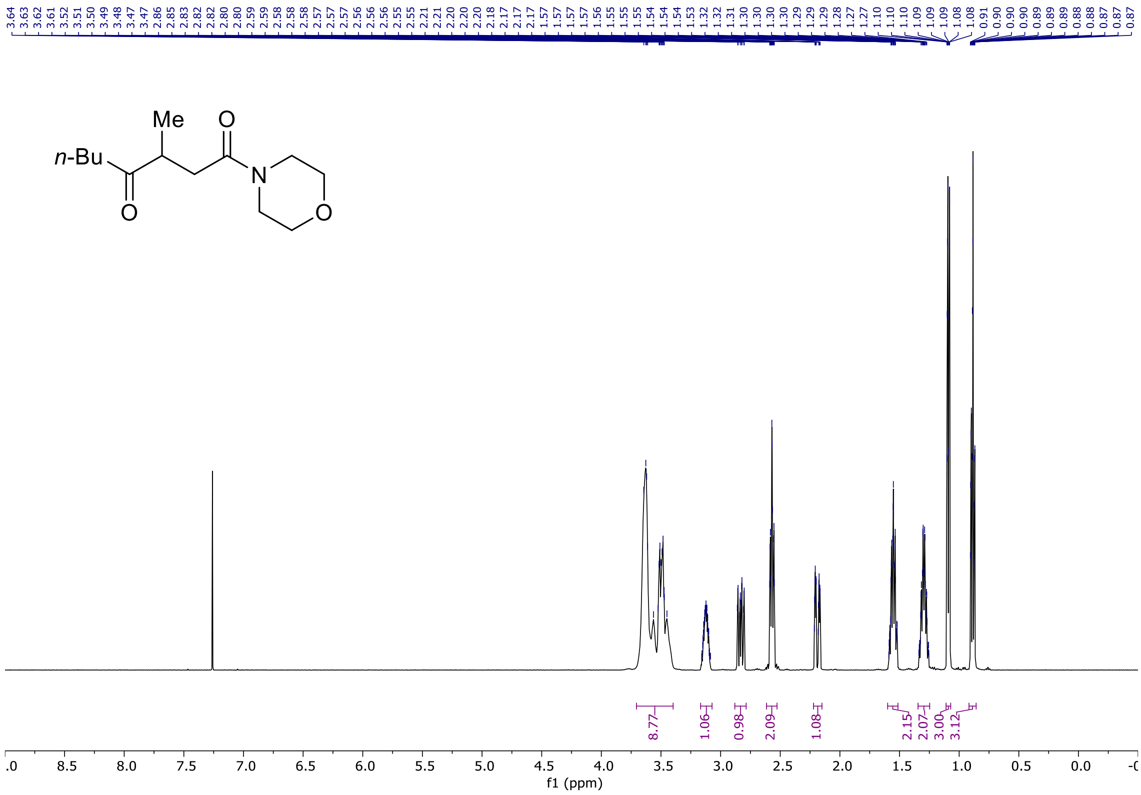

^{13}C NMR (126 MHz, $CDCl_3$) of **S2j**

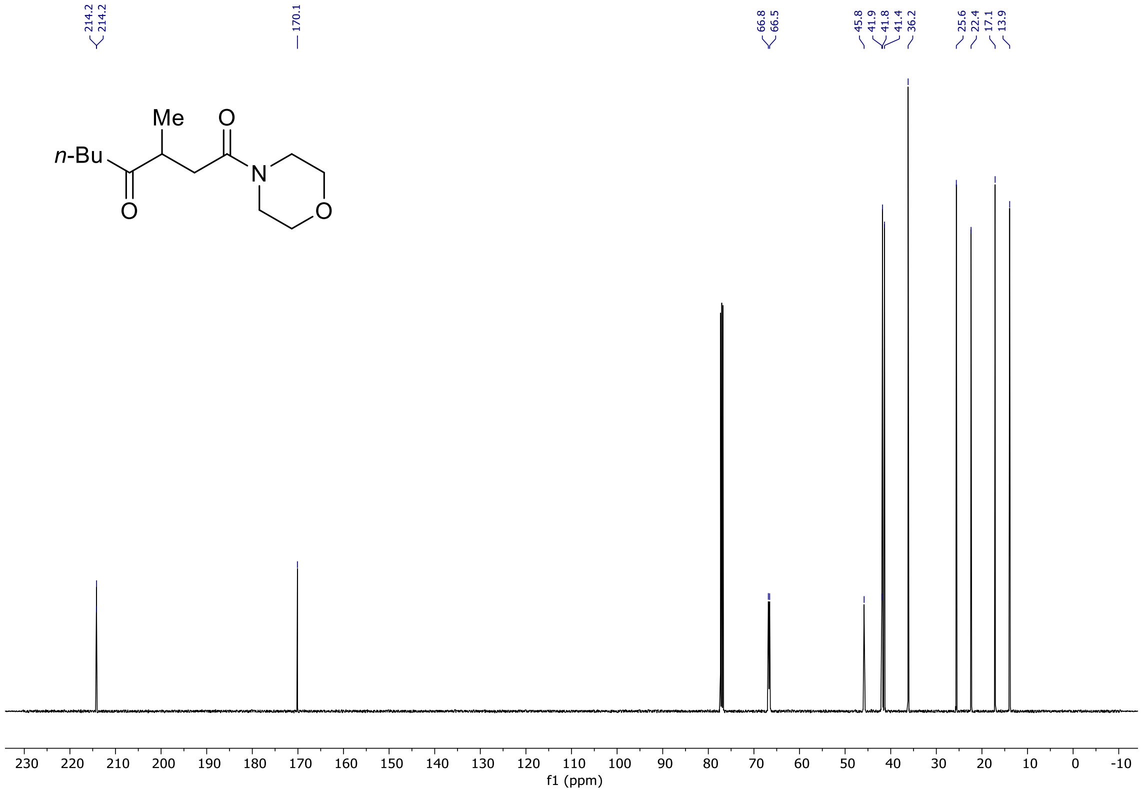

^1H NMR (500 MHz, C_6D_6) of **S3j**

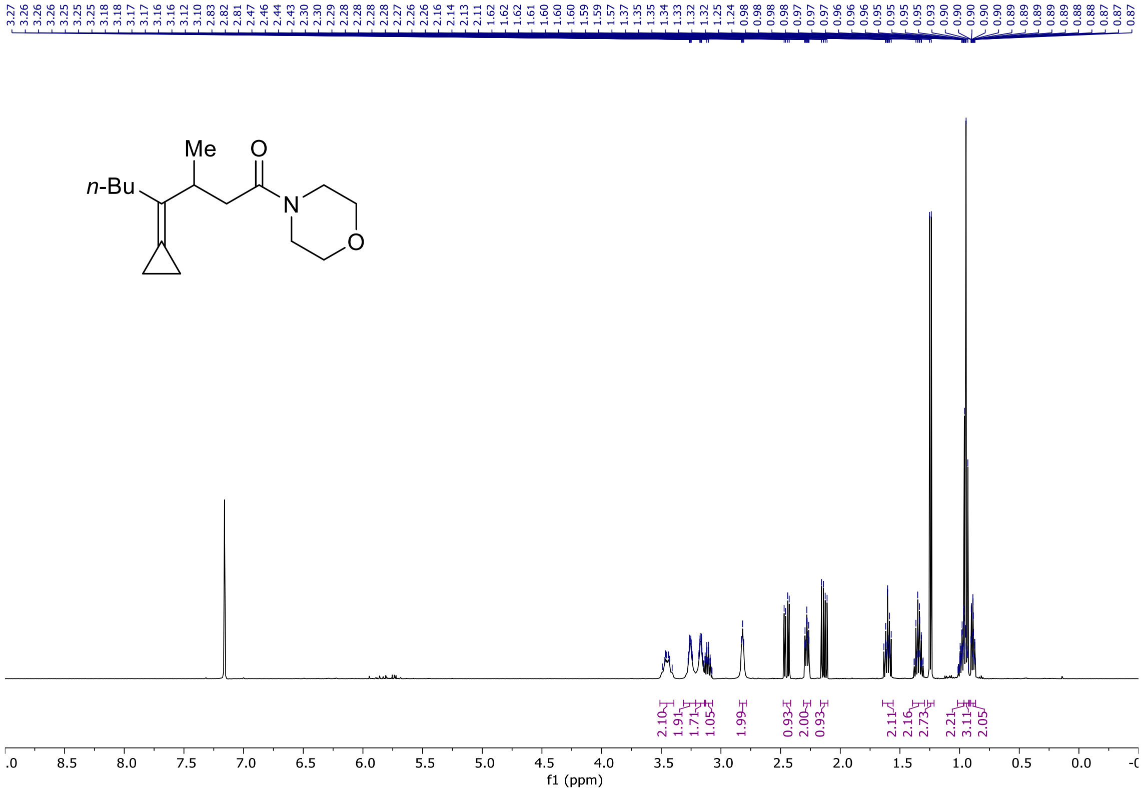

^{13}C NMR (126 MHz, C_6D_6) of **S3j**

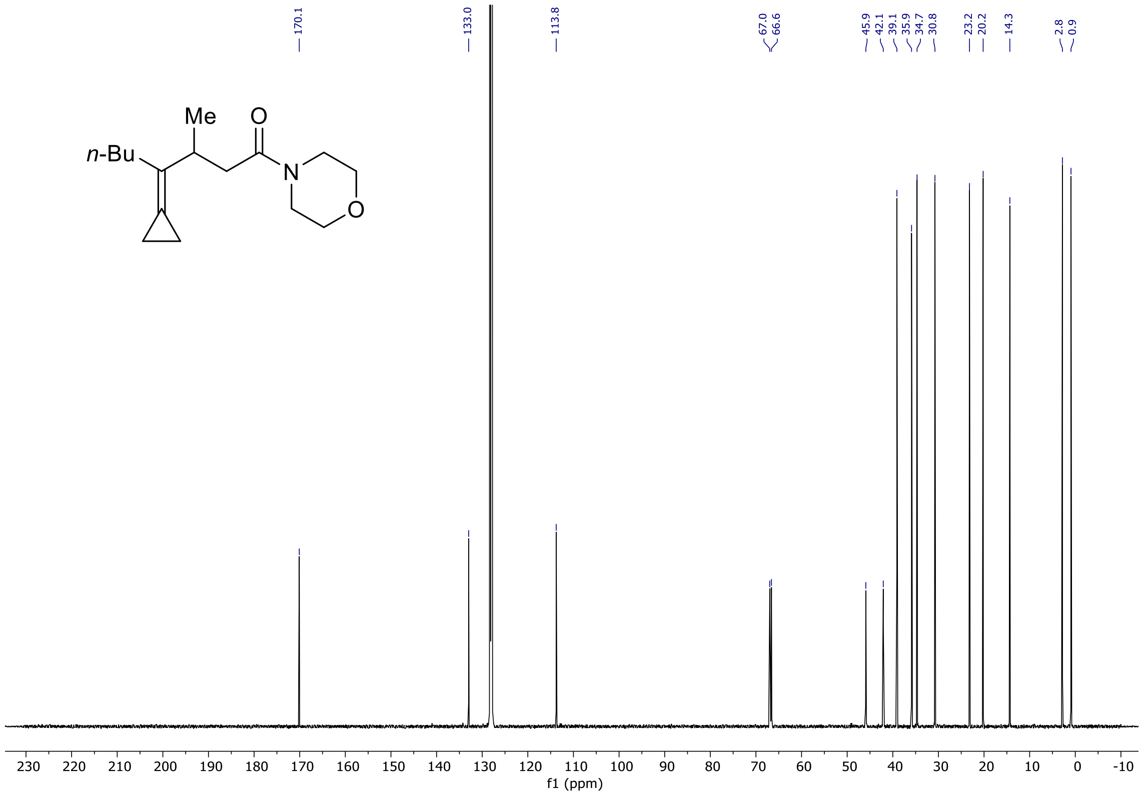

^{1}H NMR (500 MHz, C_6D_6) of **1j**

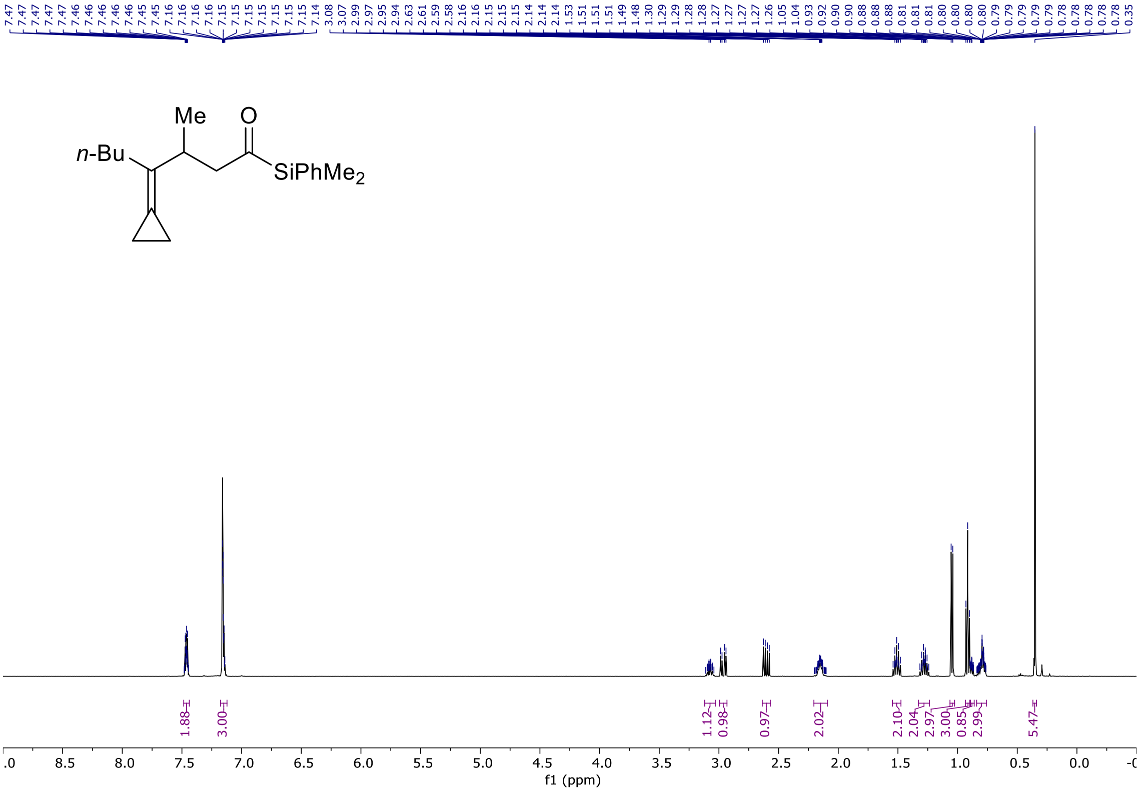

^{13}C NMR (126 MHz, C_6D_6) of **1j**

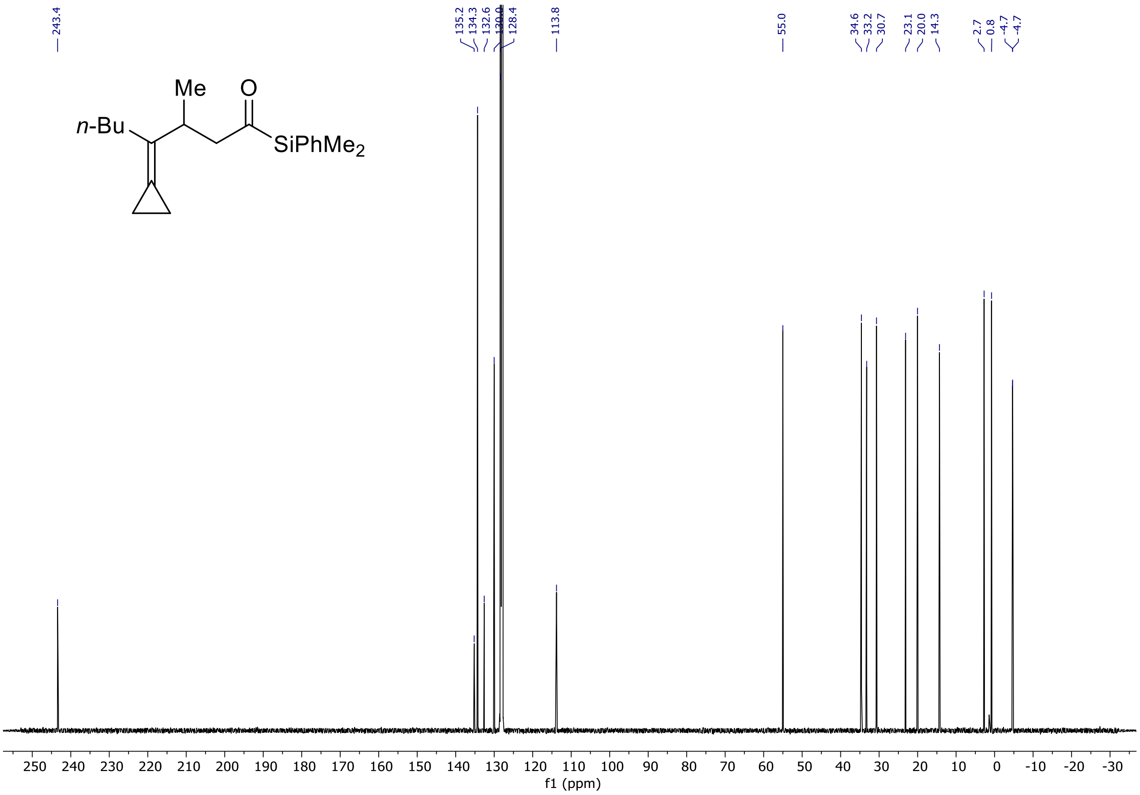

^{1}H NMR (400 MHz, C_6D_6) of **2j**

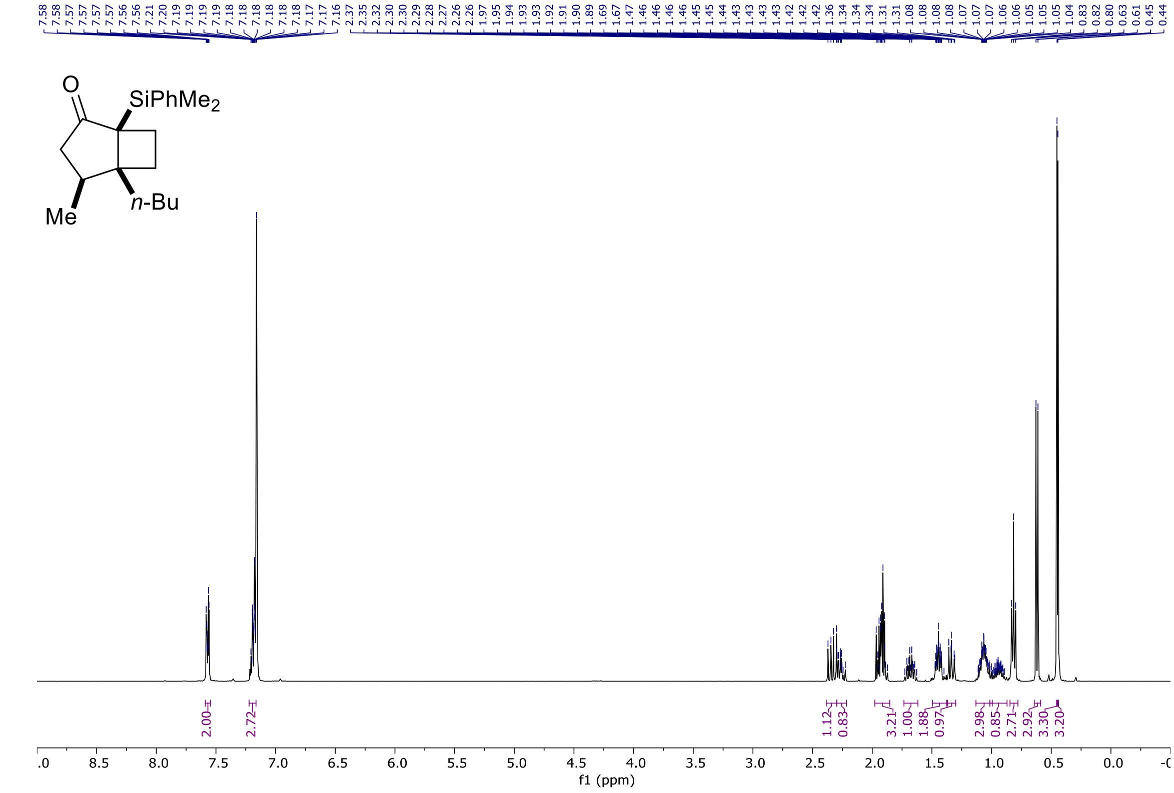

^{13}C NMR (151 MHz, C_6D_6) of **2j**

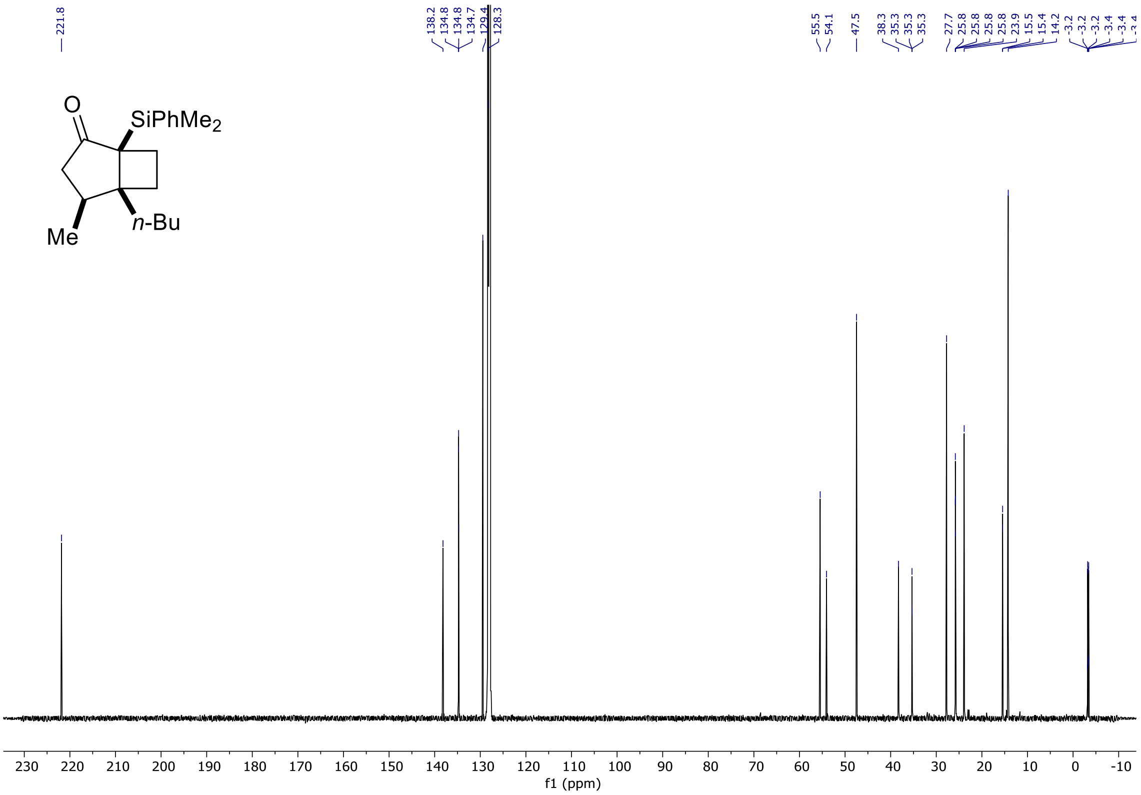

NOESY (600 MHz, C_6D_6) of **2j**

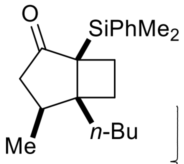

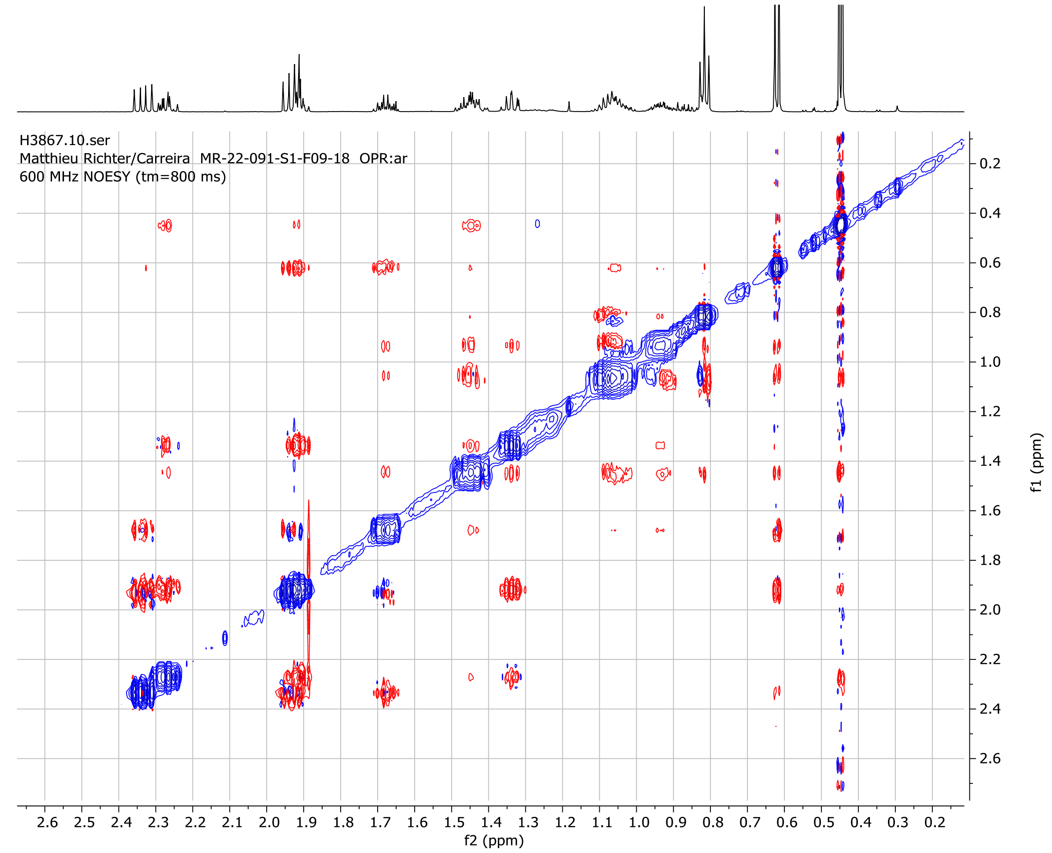

^{1}H NMR (500 MHz, $CDCl_3$) of **S5-3**

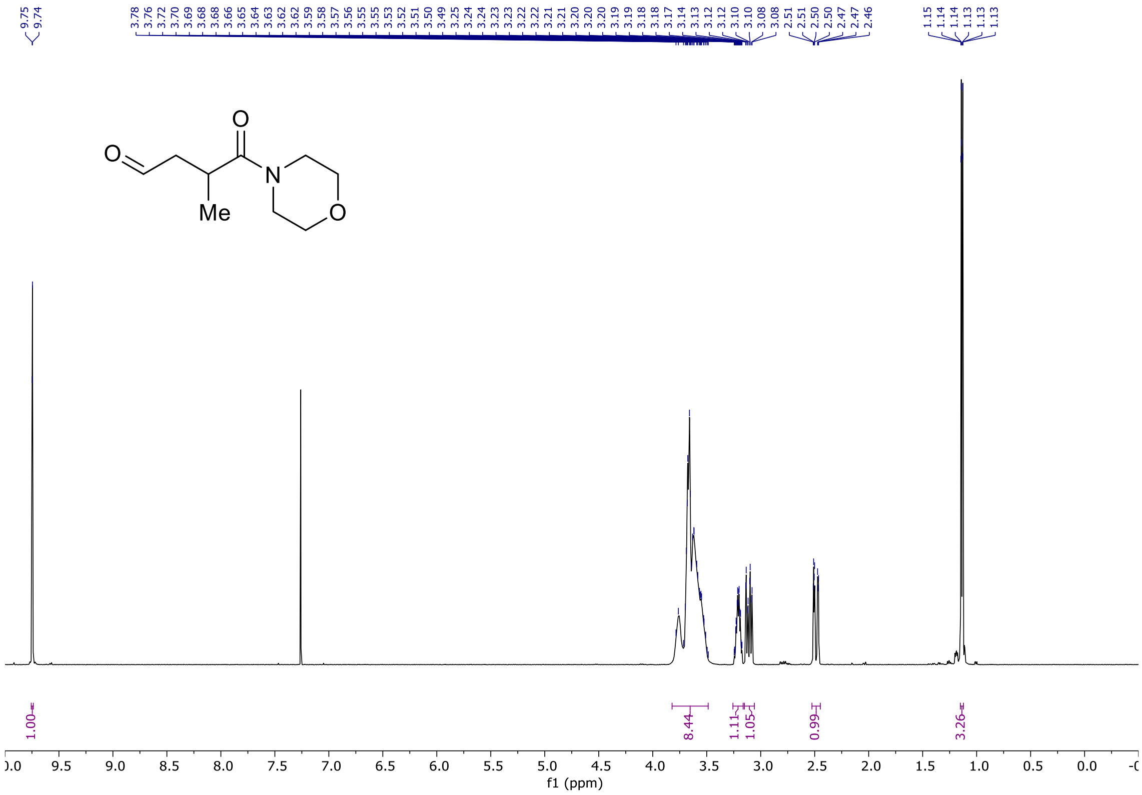

^{13}C NMR (126 MHz, $CDCl_3$) of **S5-3**

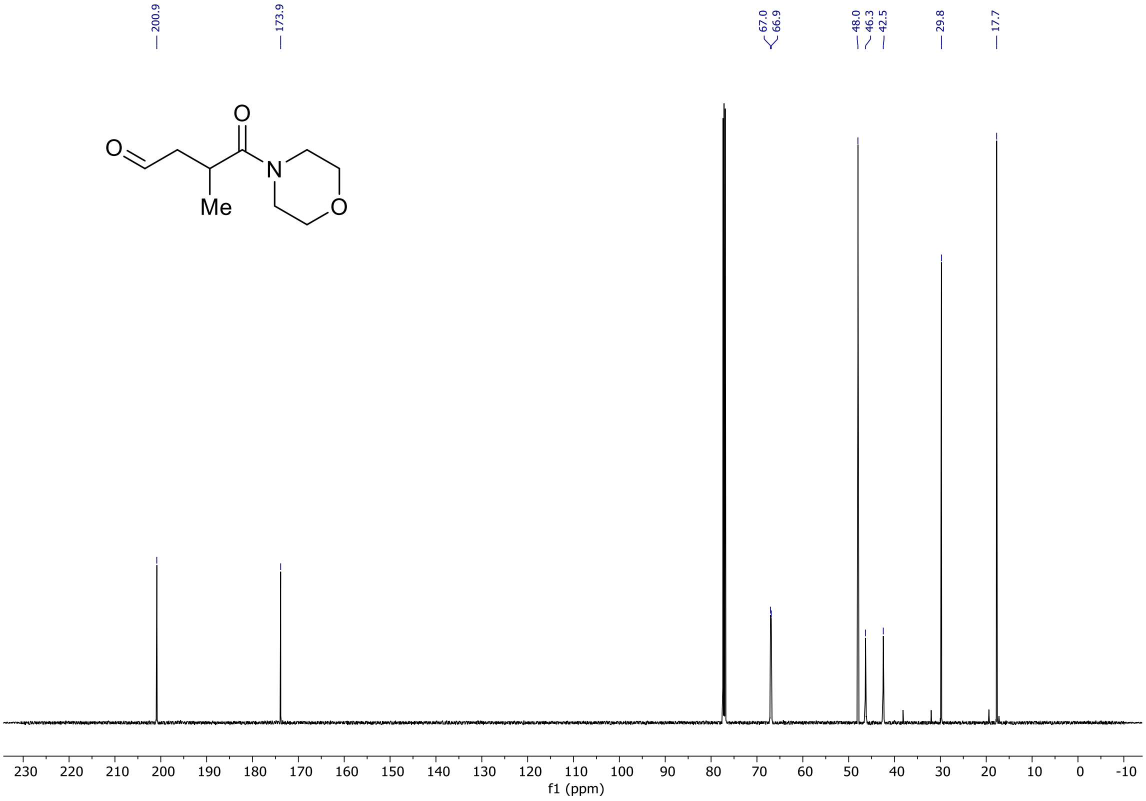

^{1}H NMR (500 MHz, $CDCl_3$) of **S2k**

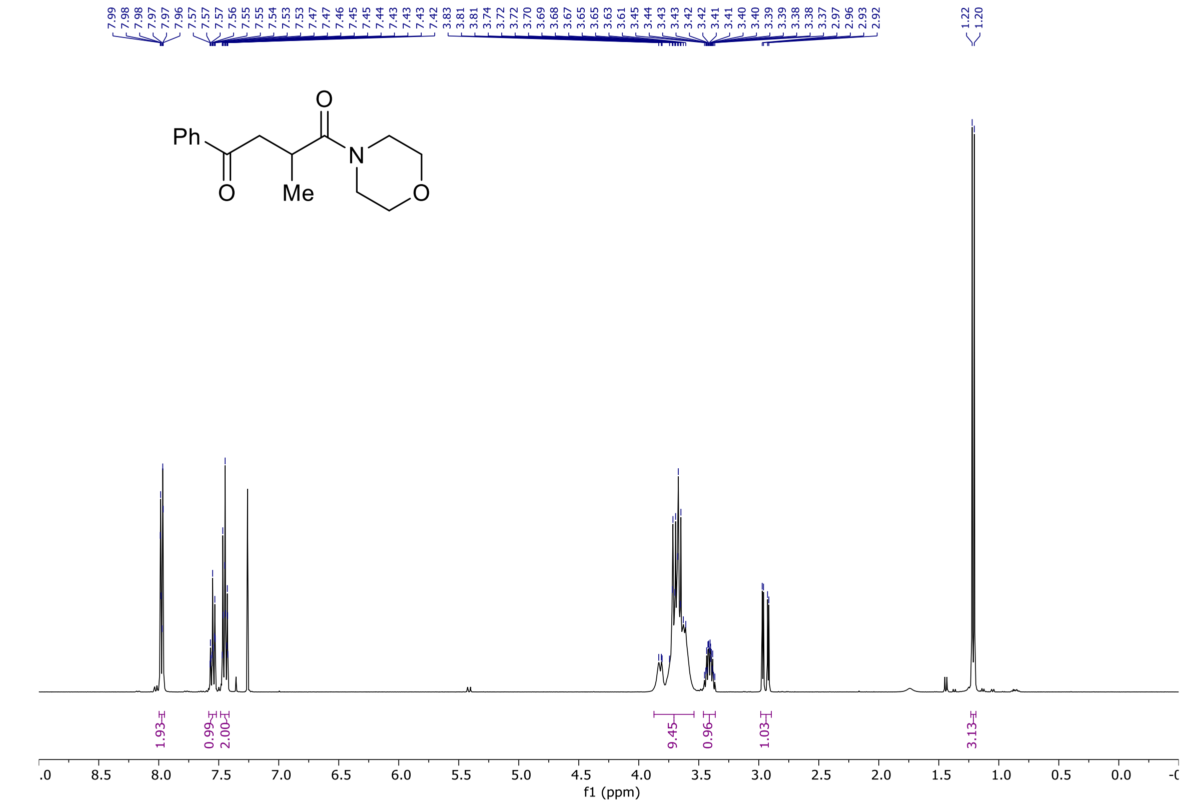

^{13}C NMR (126 MHz, $CDCl_3$) of **S2k**

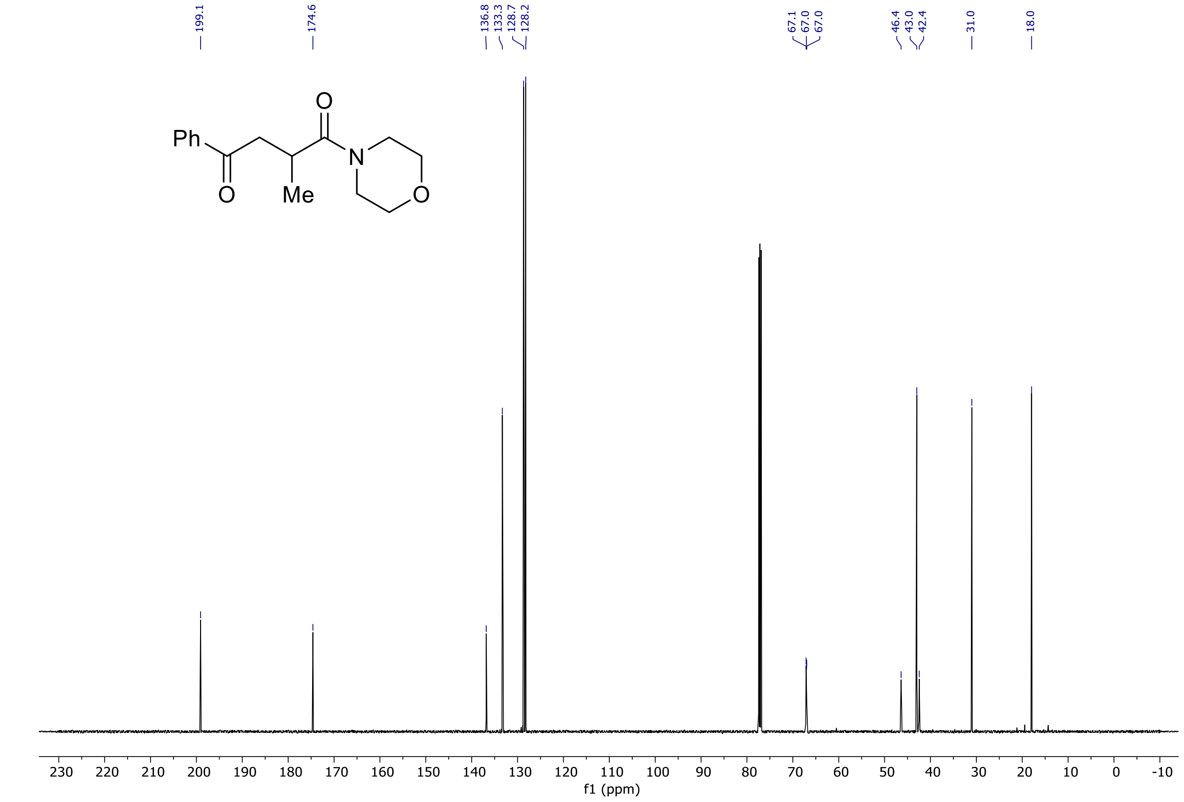

^{1}H NMR (400 MHz, C_6D_6) of **1k**

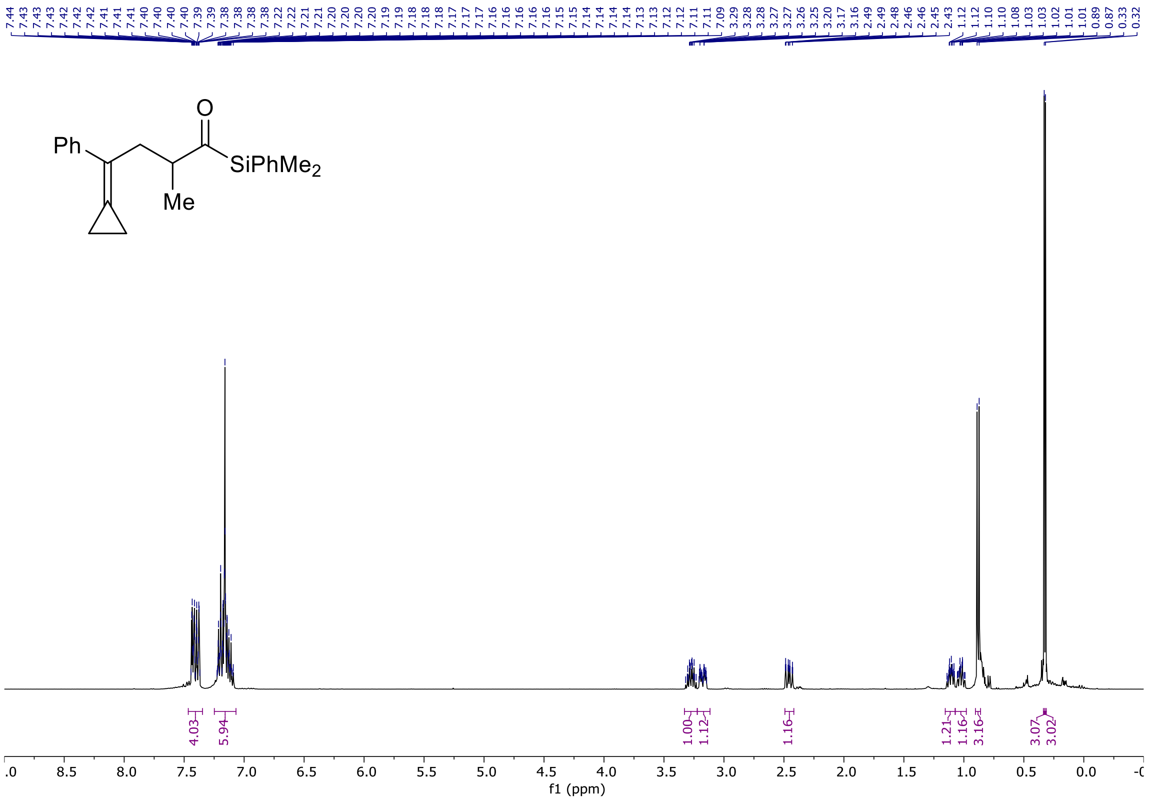

^{13}C NMR (126 MHz, C_6D_6) of **1k**

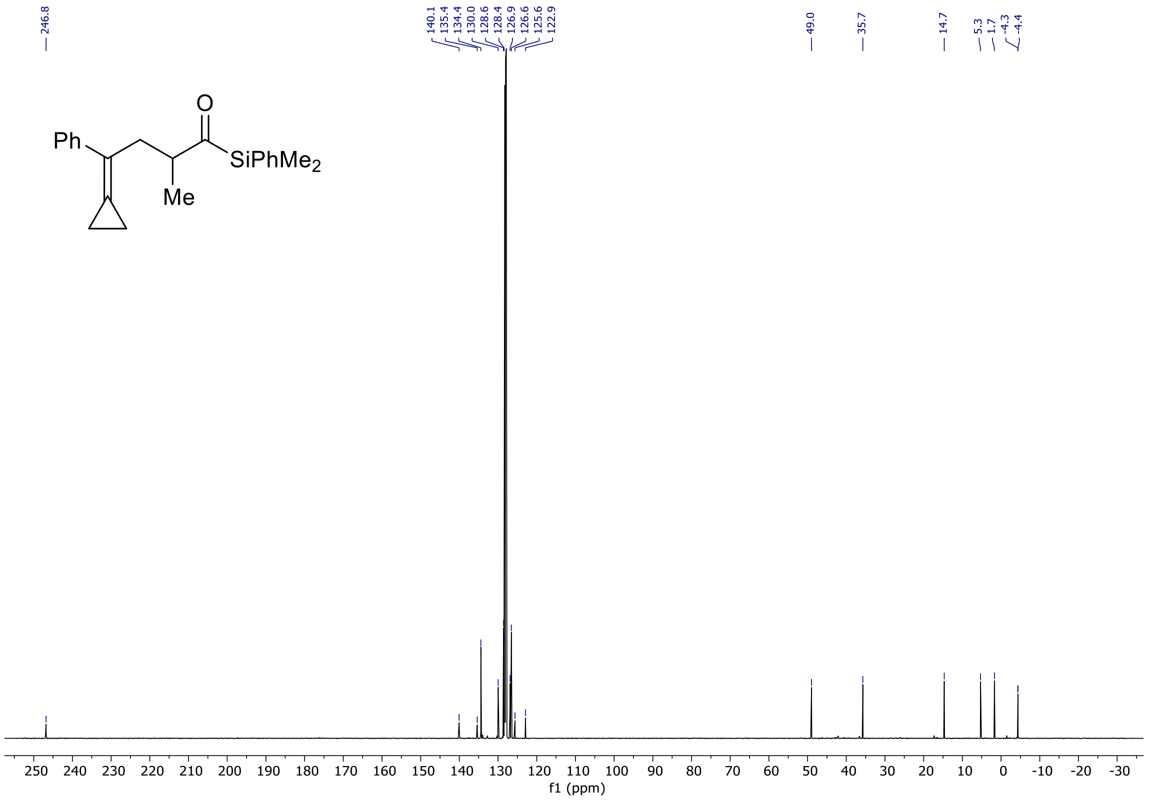

^{1}H NMR (400 MHz, C_6D_6) of **2k**

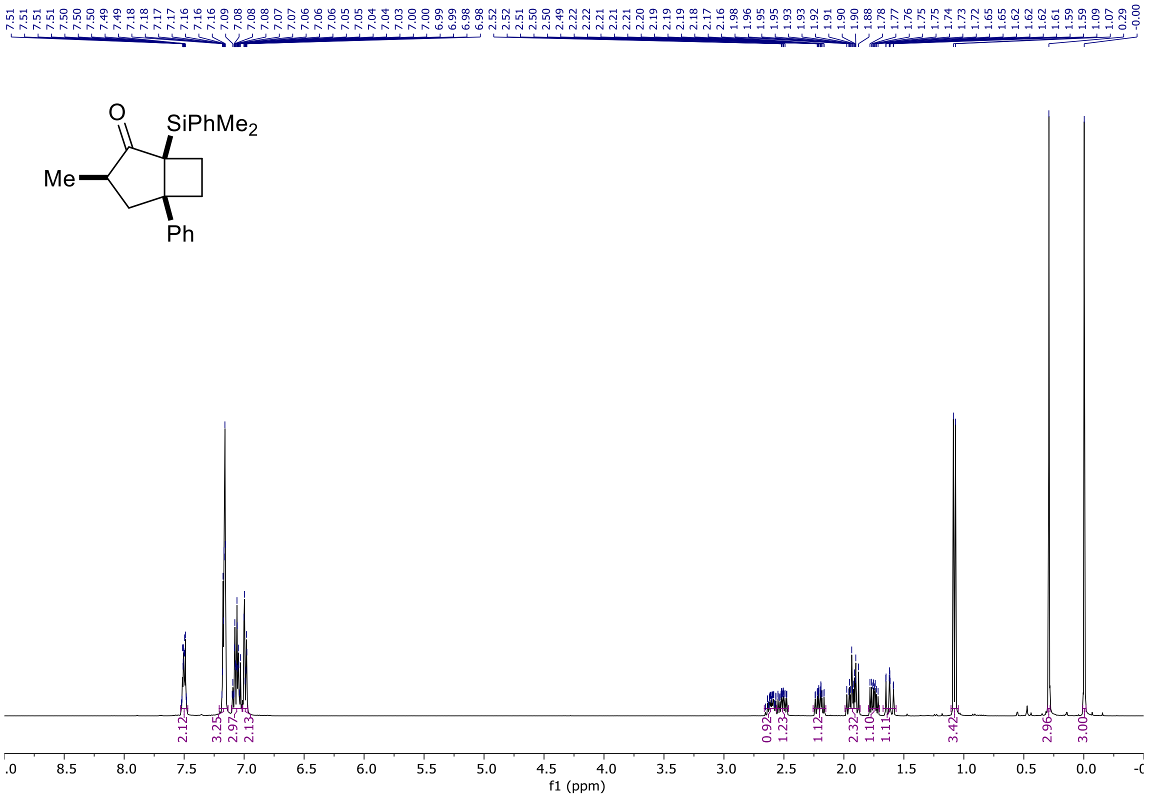

^{13}C NMR (101 MHz, C_6D_6) of **2k**

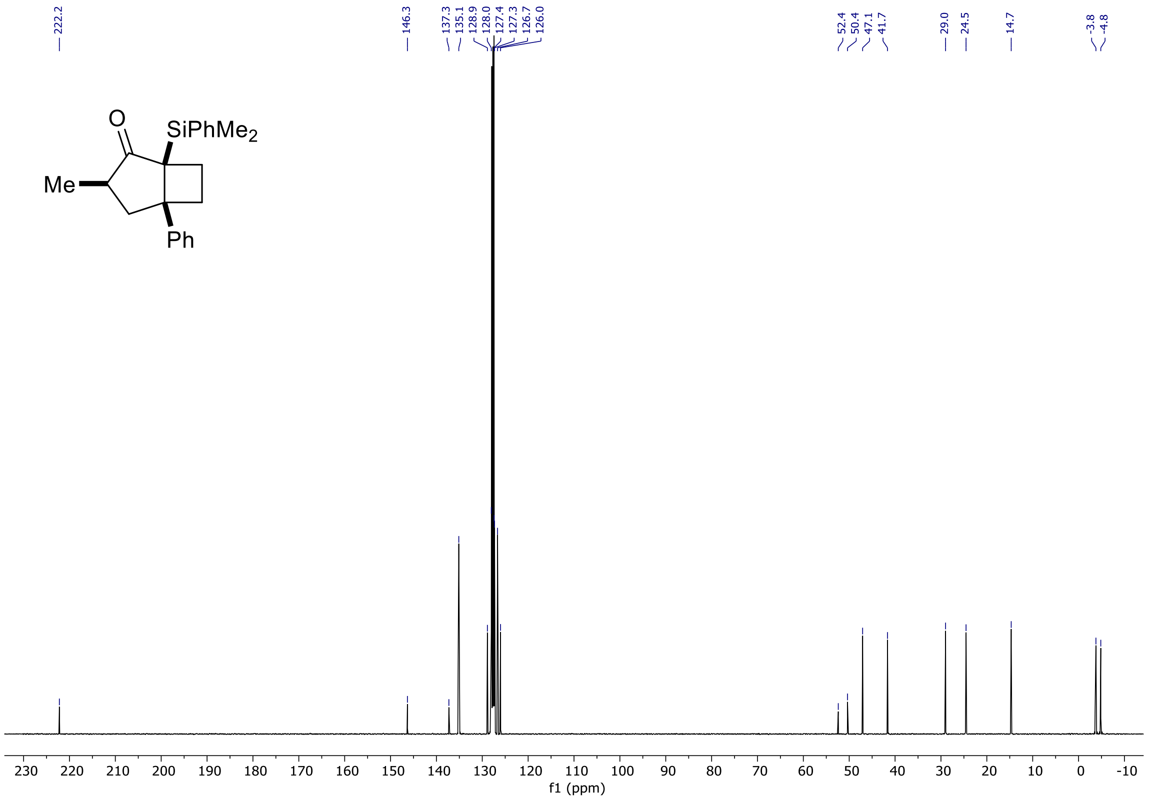

NOESY (600 MHz, C_6D_6) of **2k**

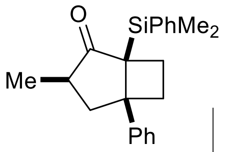

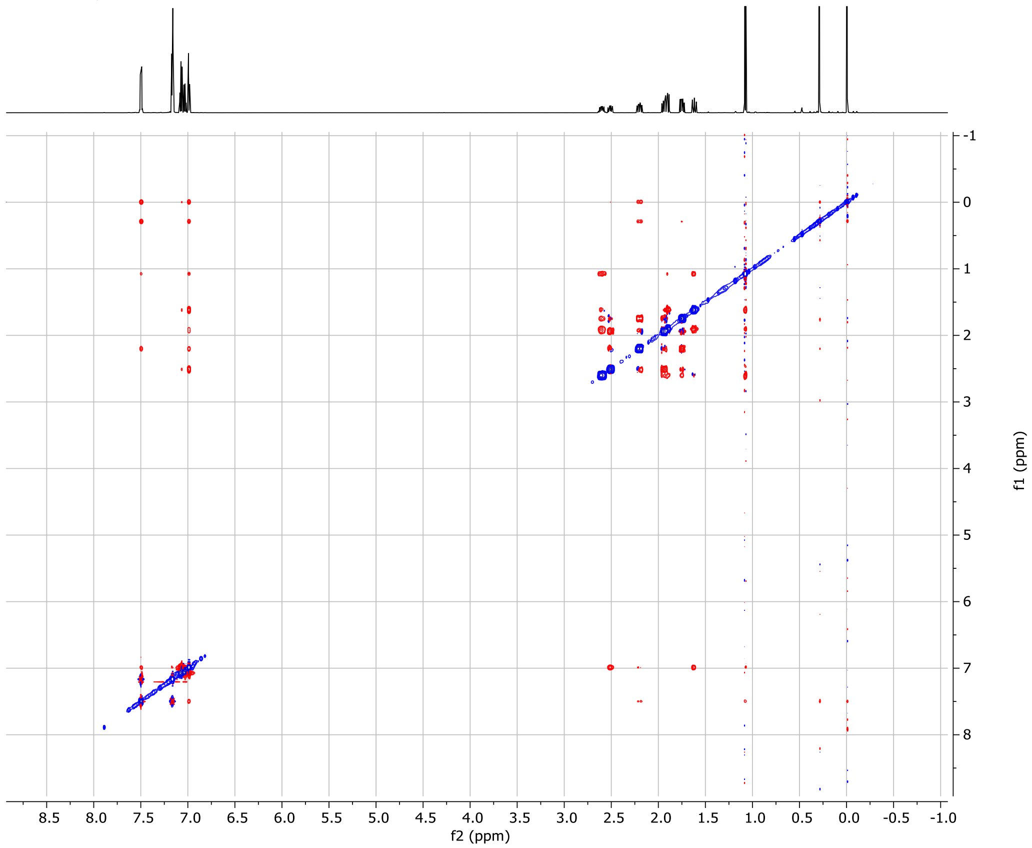

^{1}H NMR (400 MHz, $CDCl_3$) of **S6**

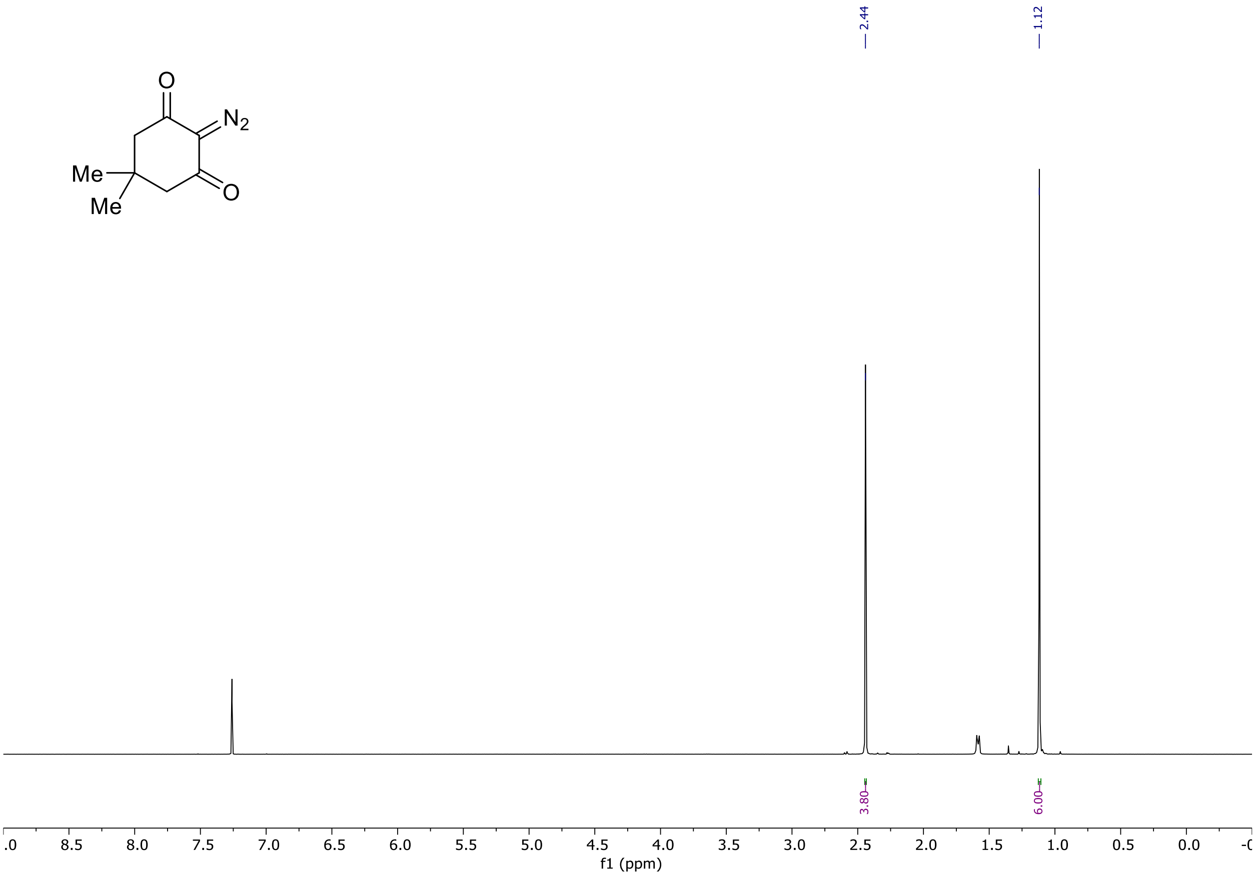

^{1}H NMR (400 MHz, $CDCl_3$) of **S7**

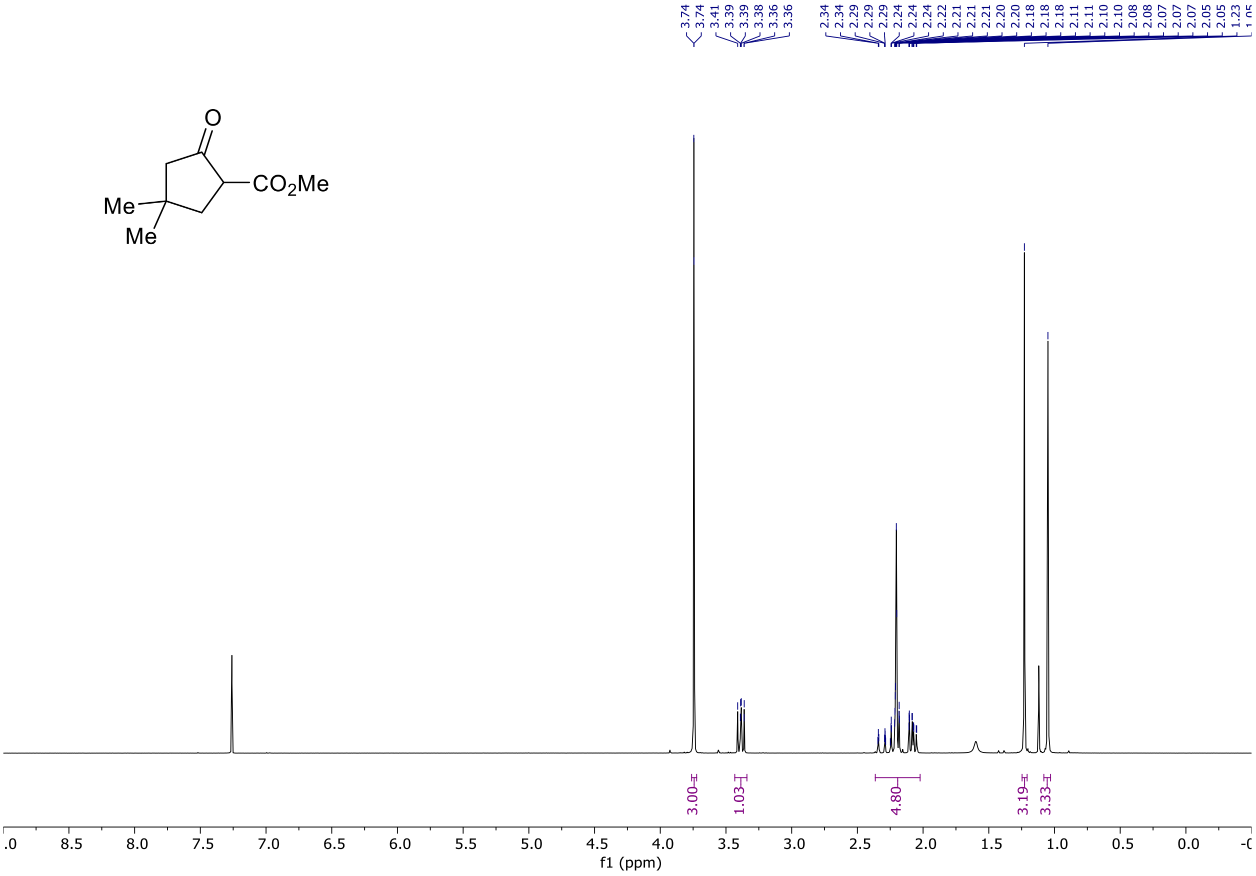

^{1}H NMR (500 MHz, $CDCl_3$) of **S8**

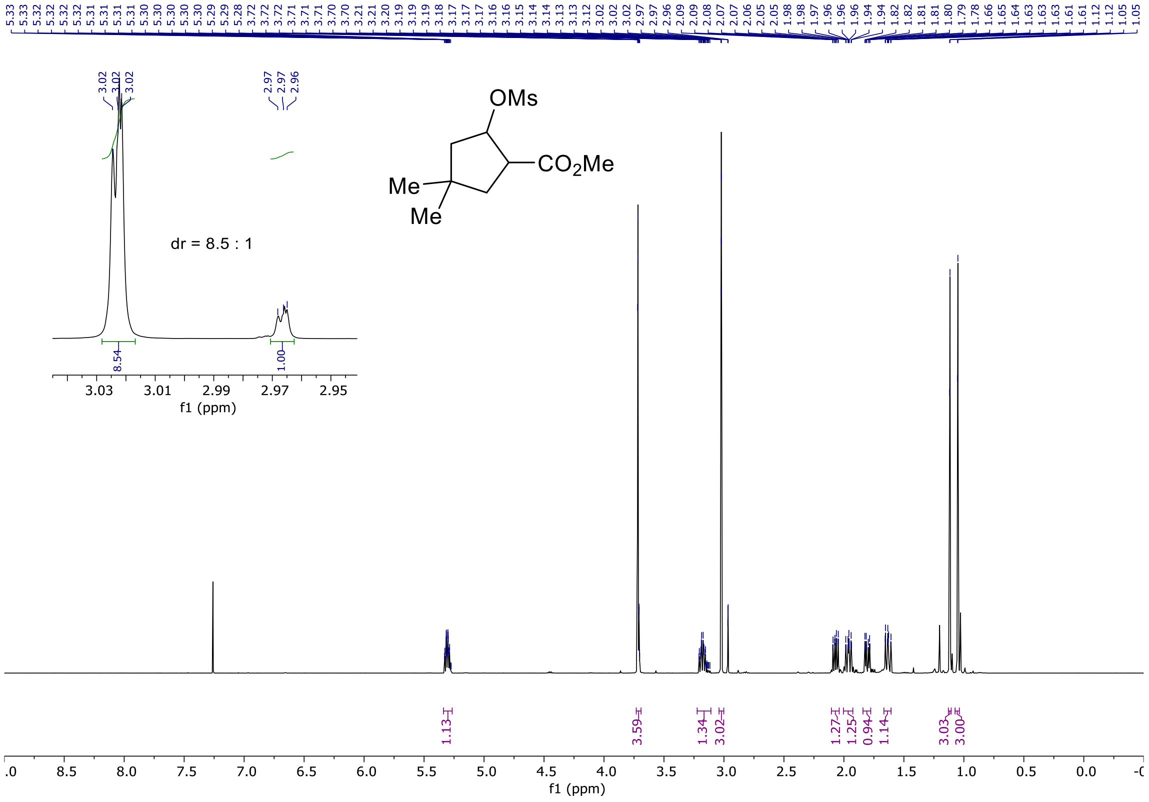

^{13}C NMR (126 MHz, $CDCl_3$) of **S8**

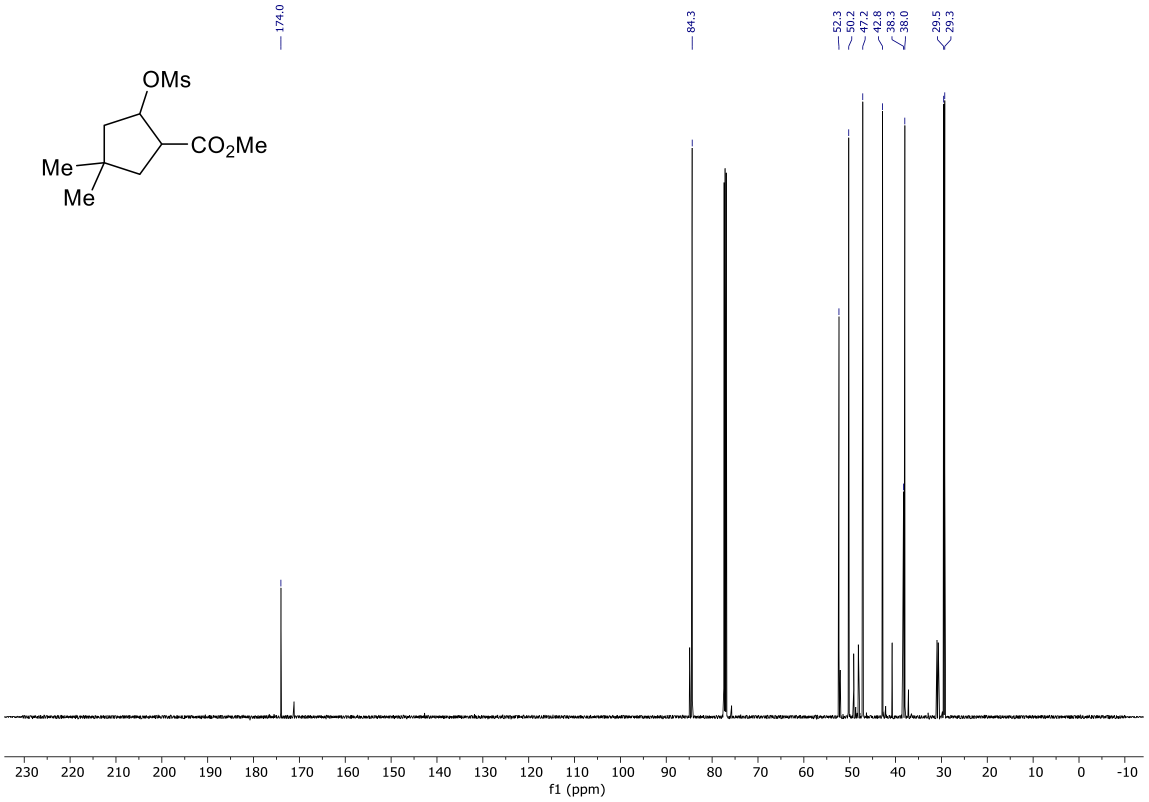

^{1}H NMR (500 MHz, $CDCl_3$) of **S9**

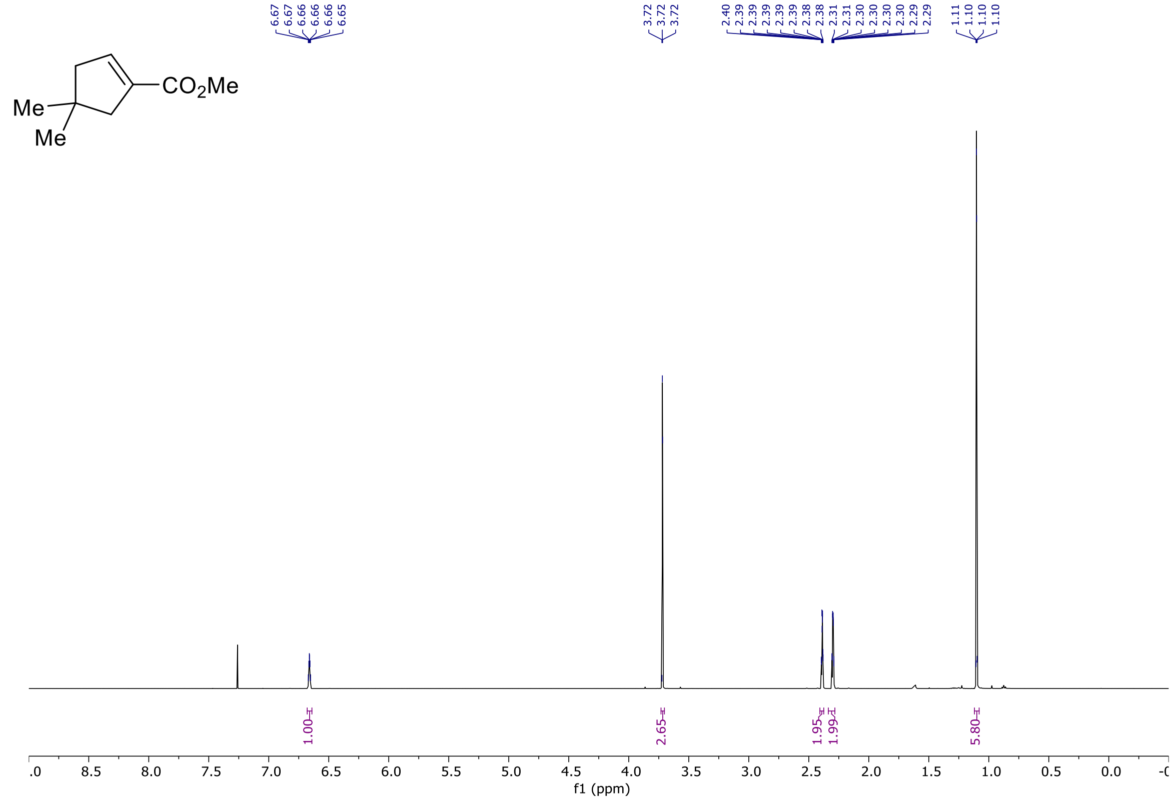

^{13}C NMR (126 MHz, $CDCl_3$) of **S9**

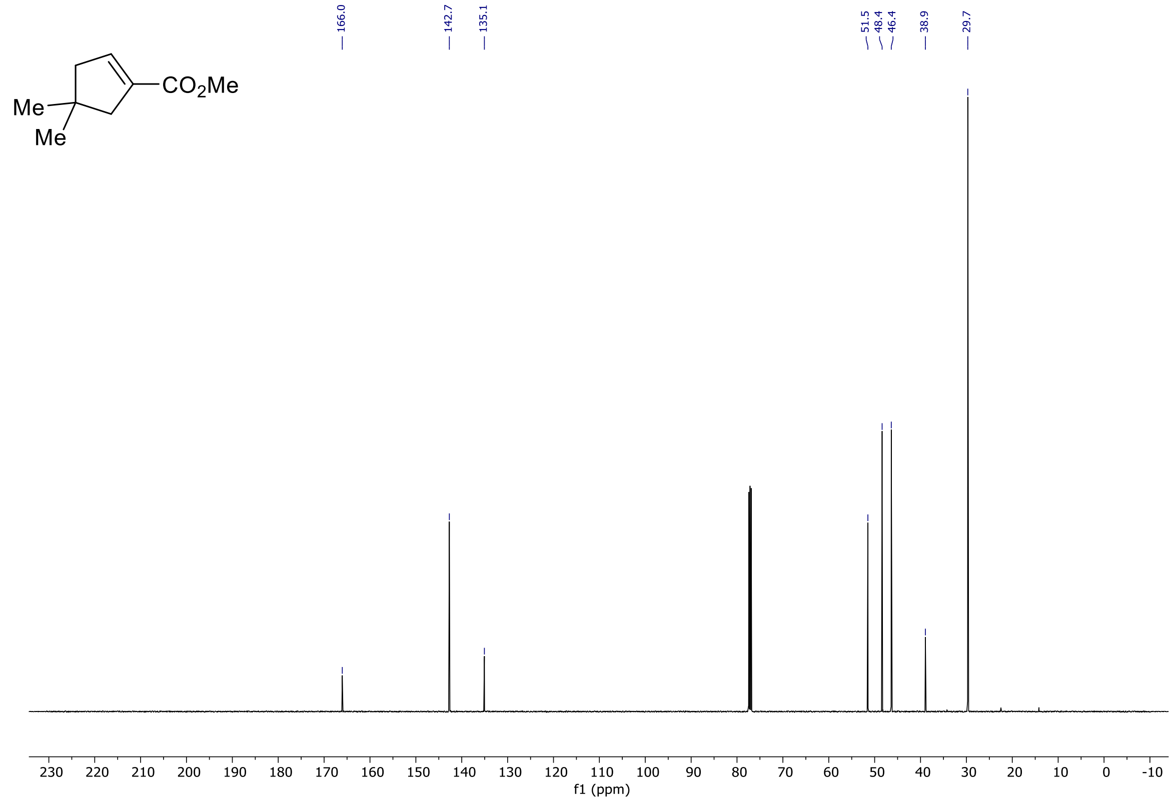

^{1}H NMR (500 MHz, $CDCl_3$) of **S10**

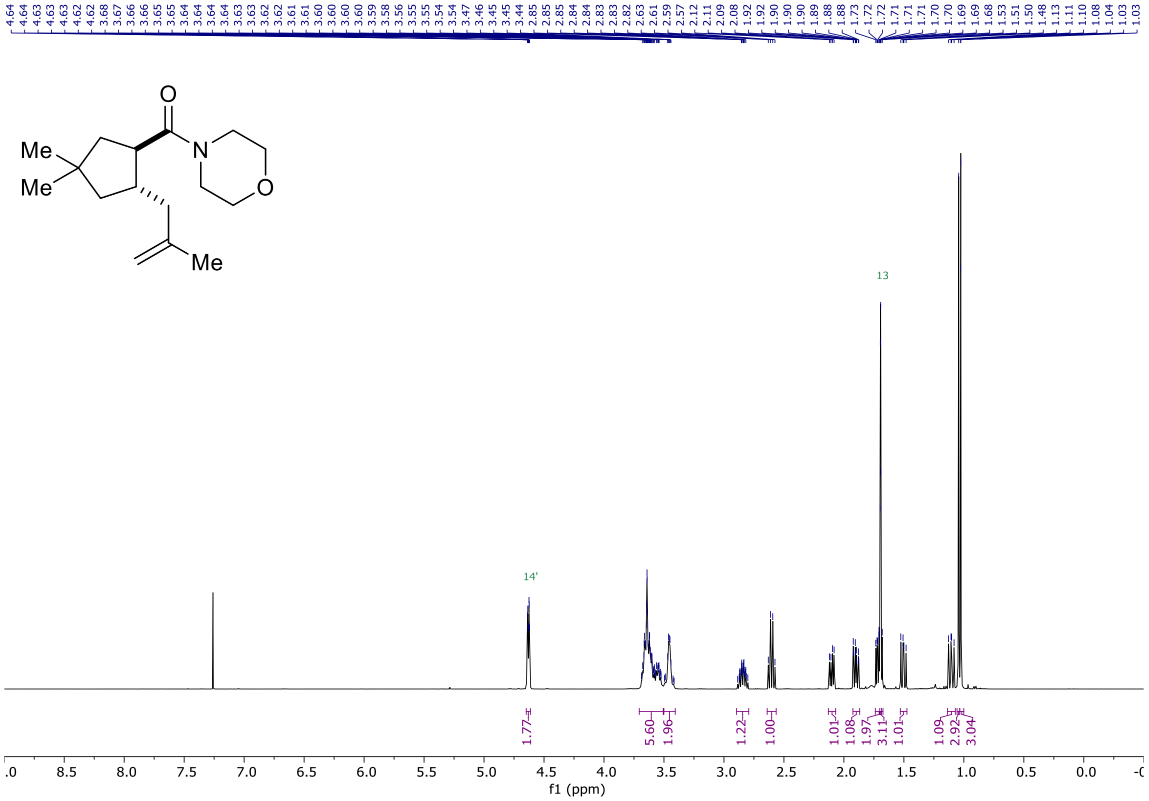

^{13}C NMR (126 MHz, $CDCl_3$) of **S10**

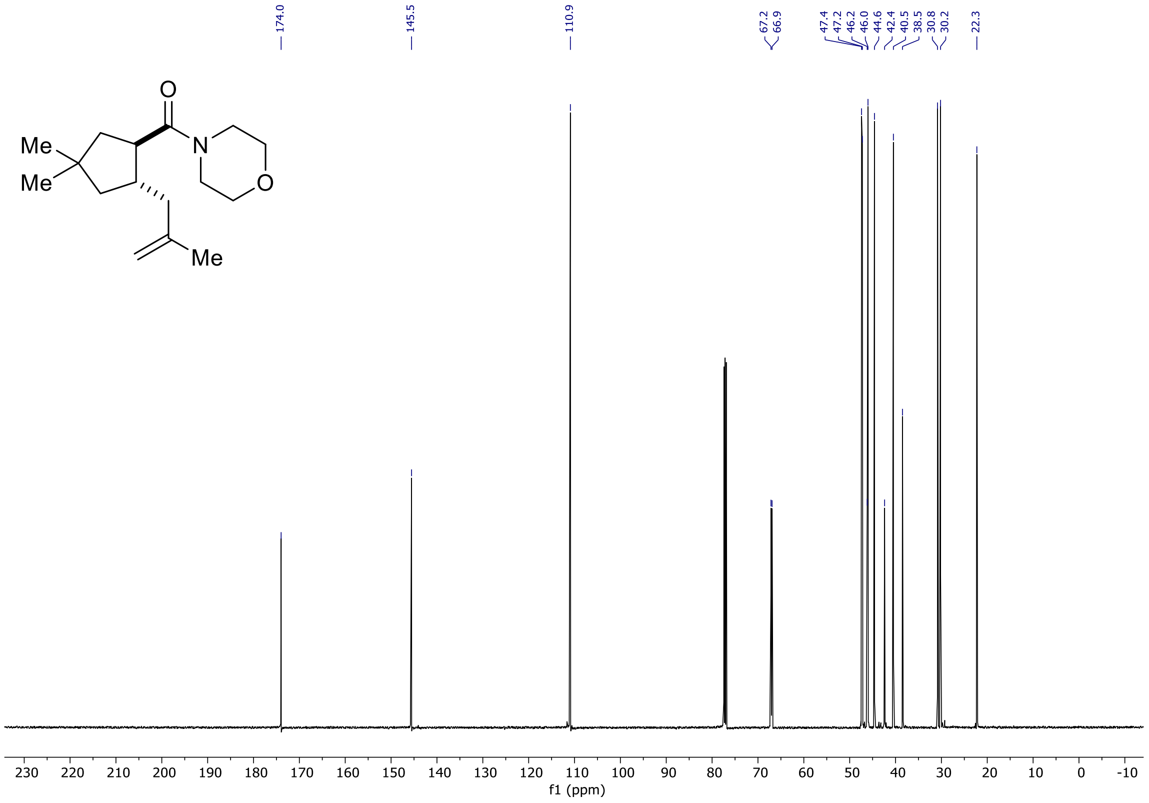

^{1}H NMR (500 MHz, $CDCl_3$) of **S2l**

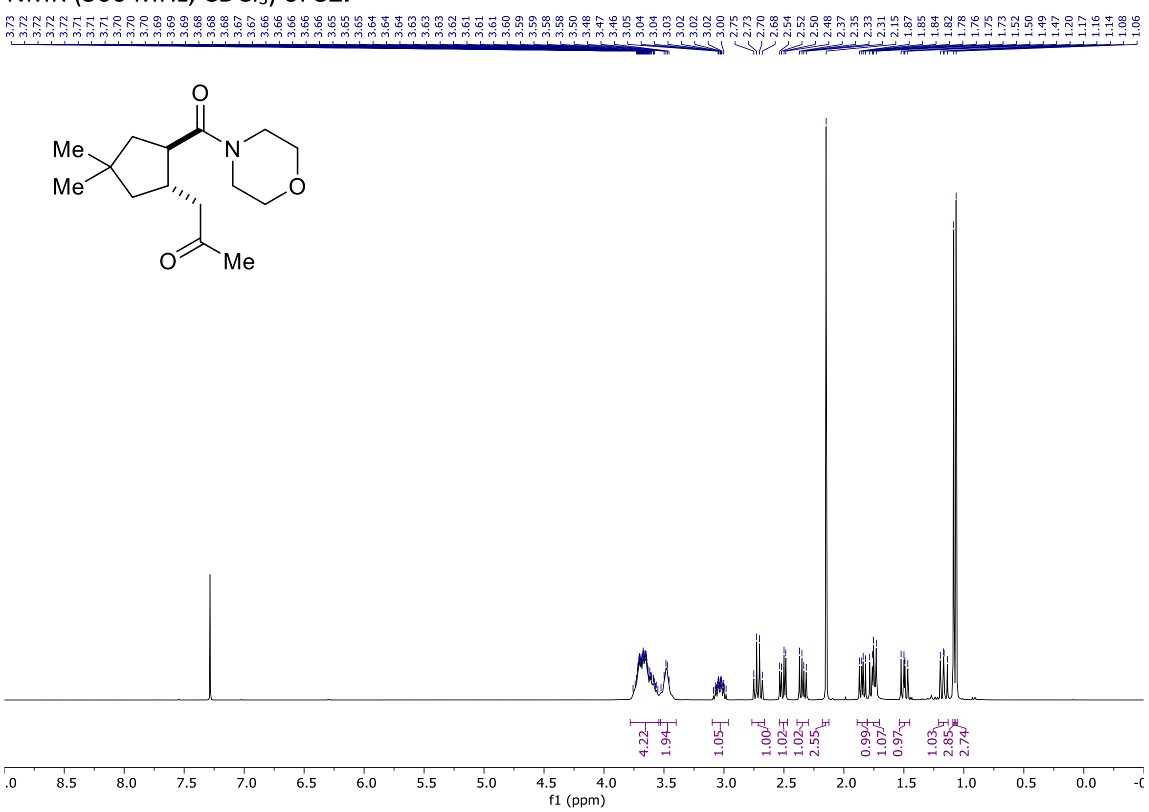

^{13}C NMR (126 MHz, $CDCl_3$) of **S2l**

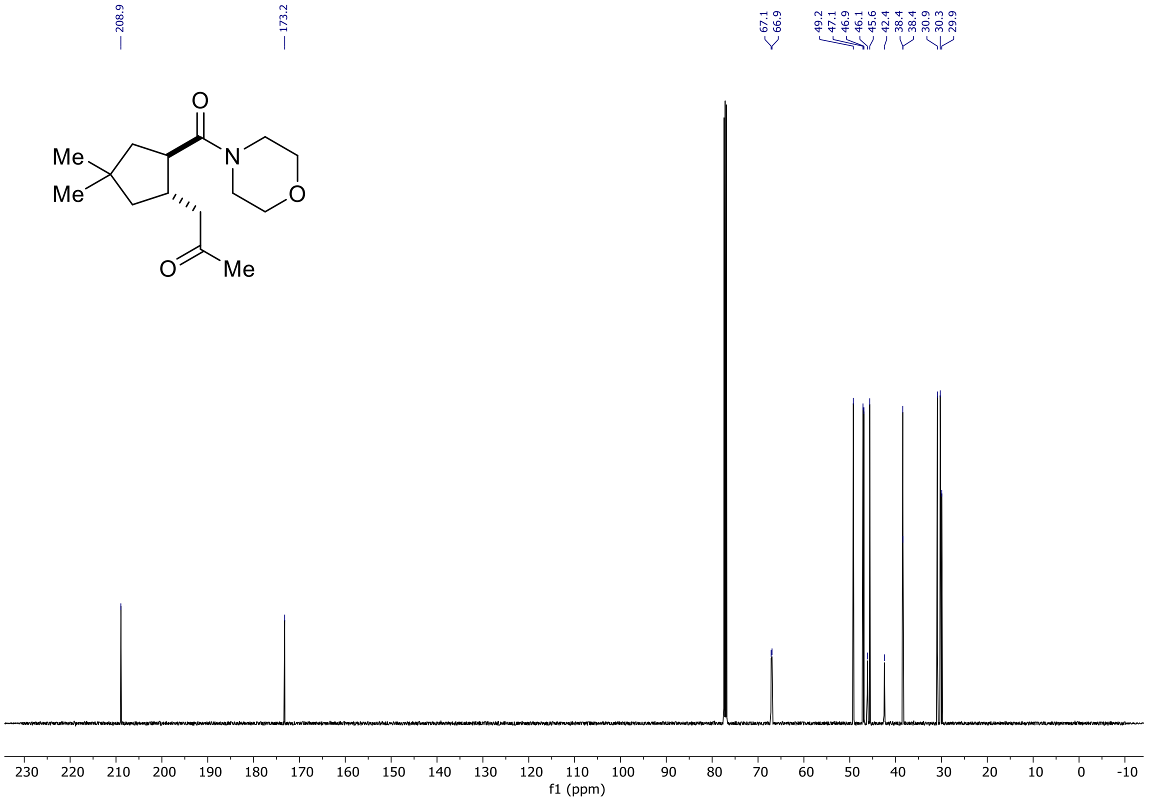

^{1}H NMR (500 MHz, C_6D_6) of **S3l**

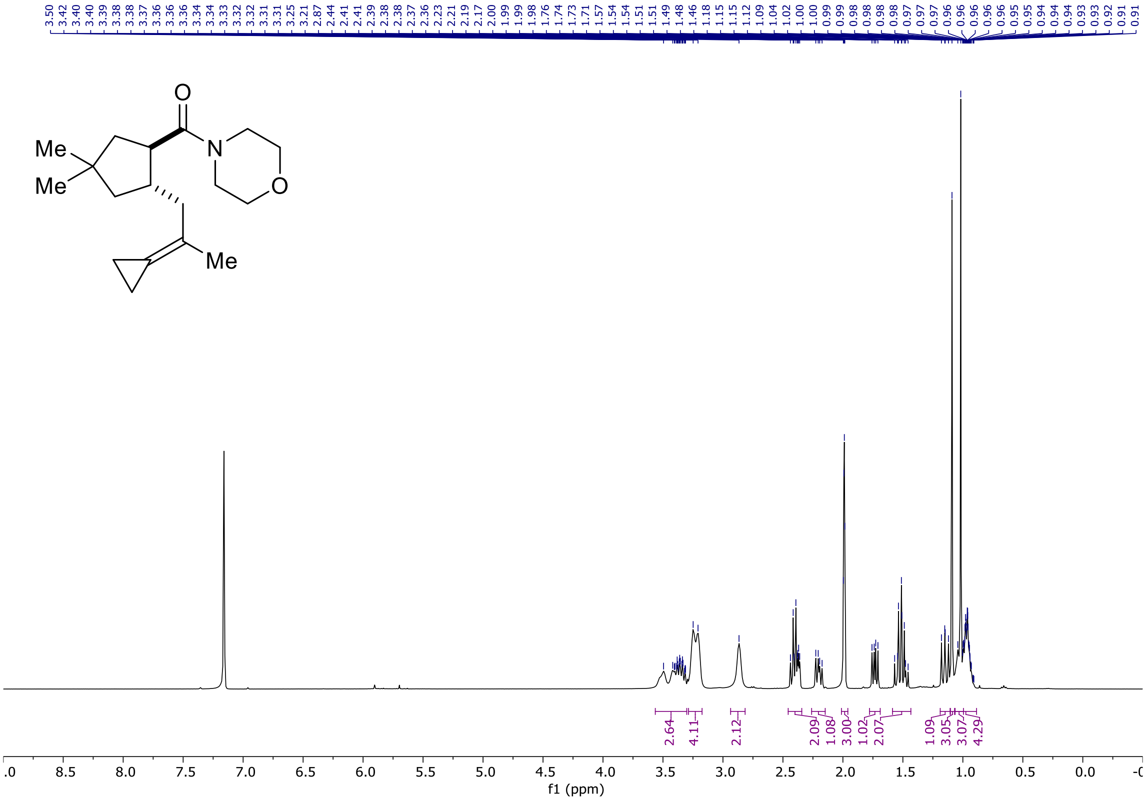

^{13}C NMR (126 MHz, $CDCl_3$) of **S3l**

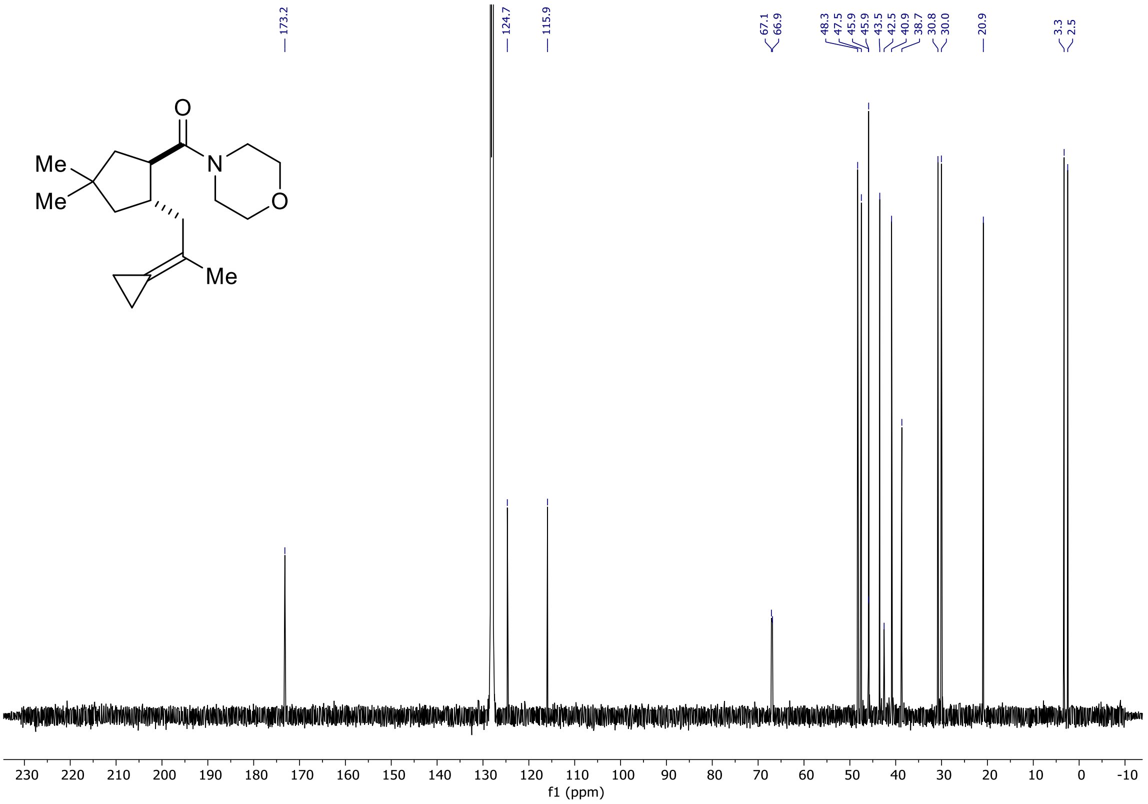

^{1}H NMR (500 MHz, C_6D_6) of **1l**

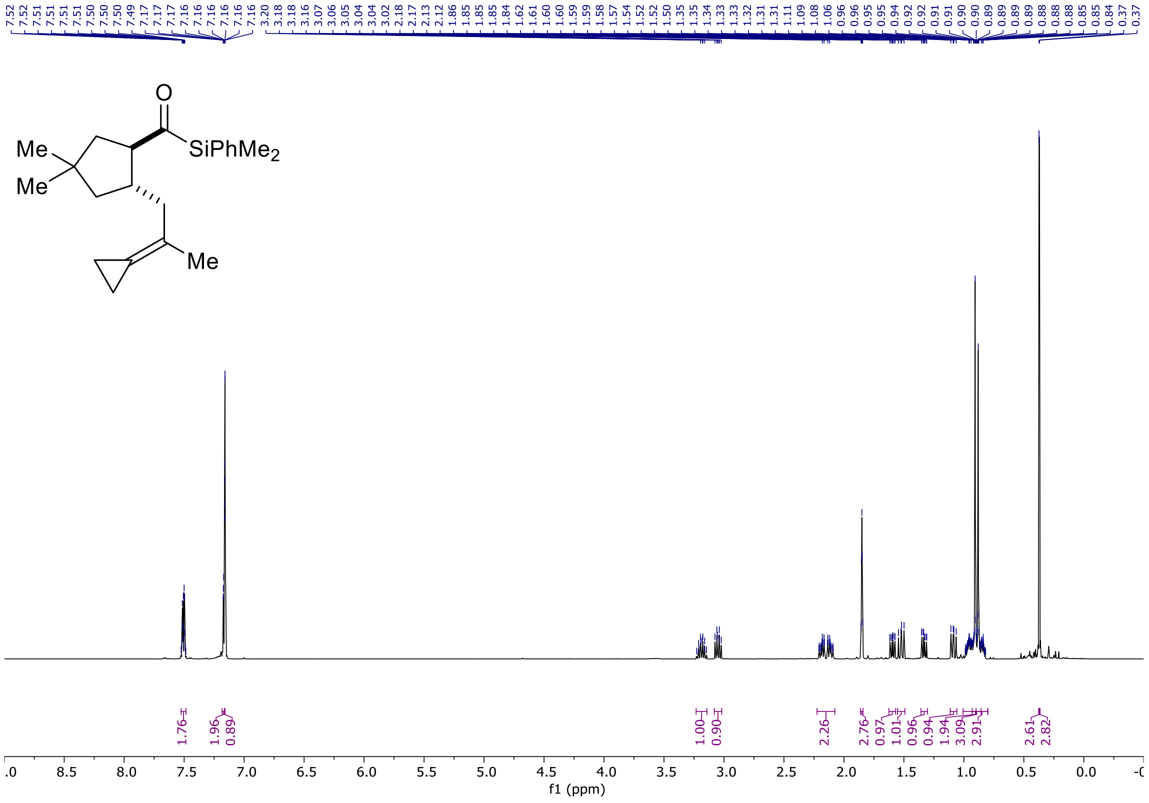

^{13}C NMR (126 MHz, C_6D_6) of **1l**

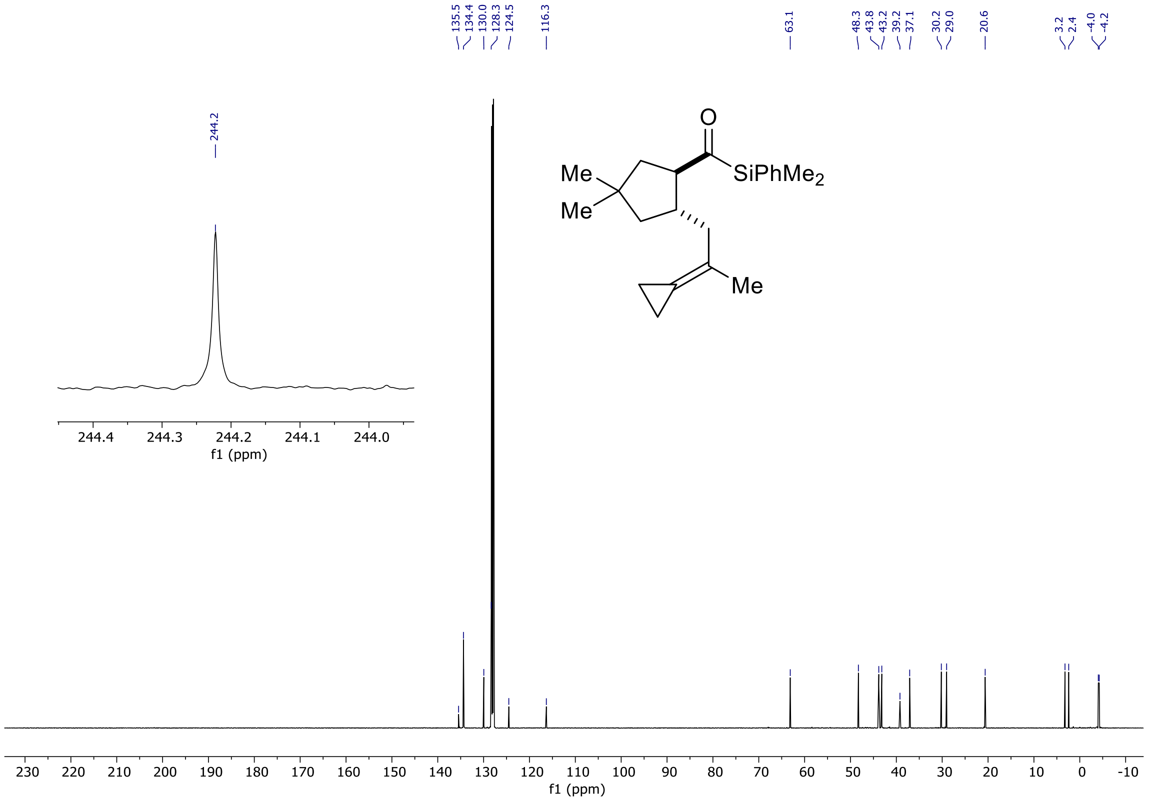

^{1}H NMR (500 MHz, C_6D_6) of **2l** (mixture of diastereomers, 3:1 dr)

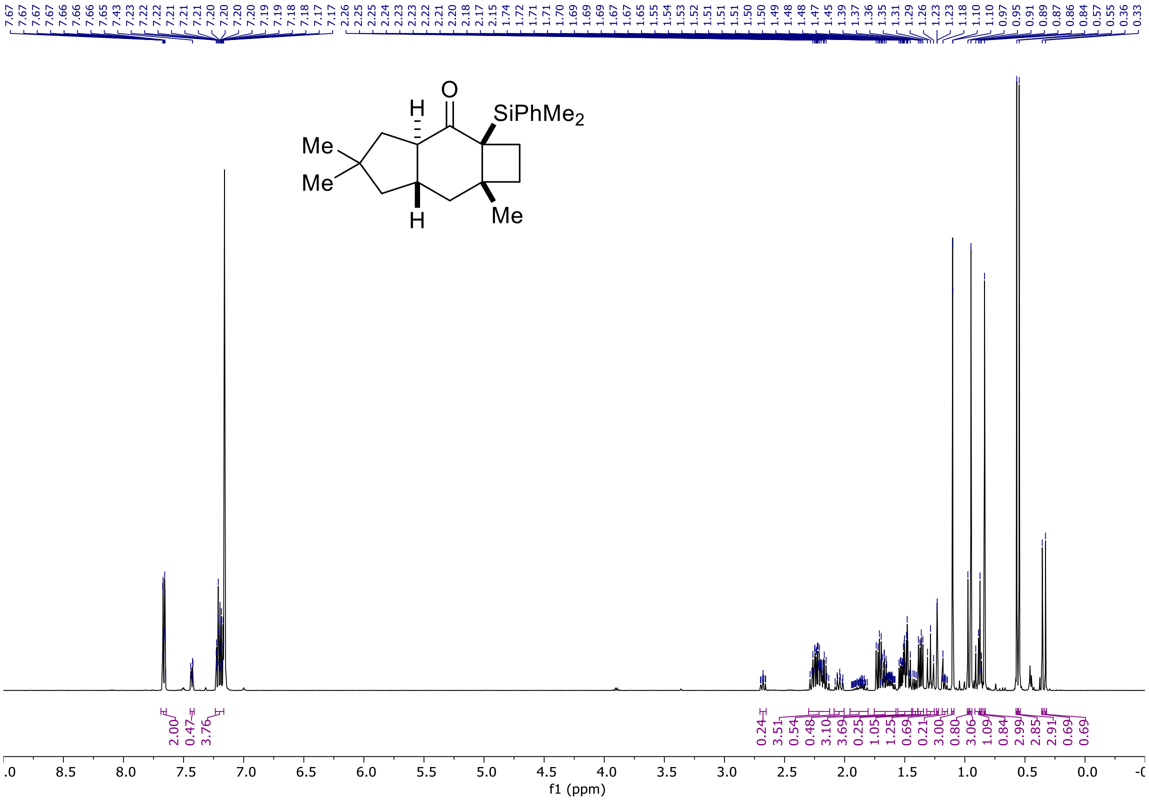

^{13}C NMR (126 MHz, C_6D_6) of **2l** (mixture of diastereomers, 3:1 dr)

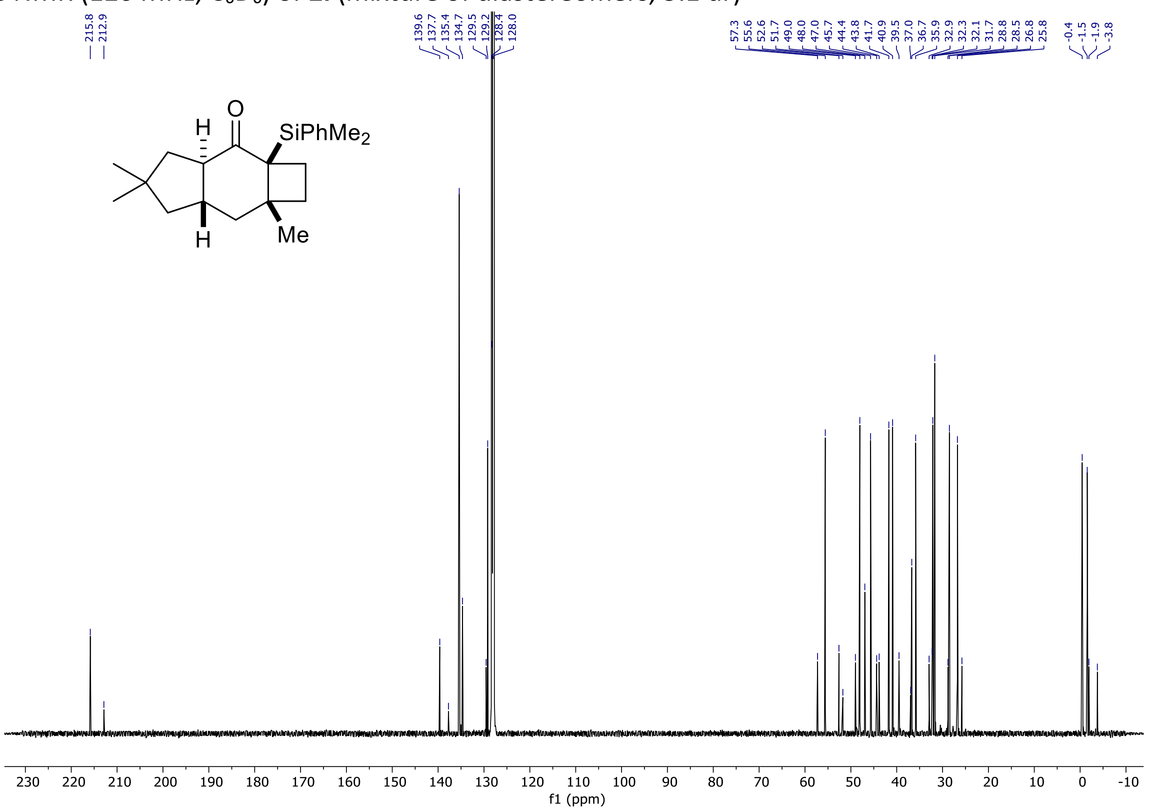

^{1}H NMR (600 MHz, $CDCl_3$) of **S11**

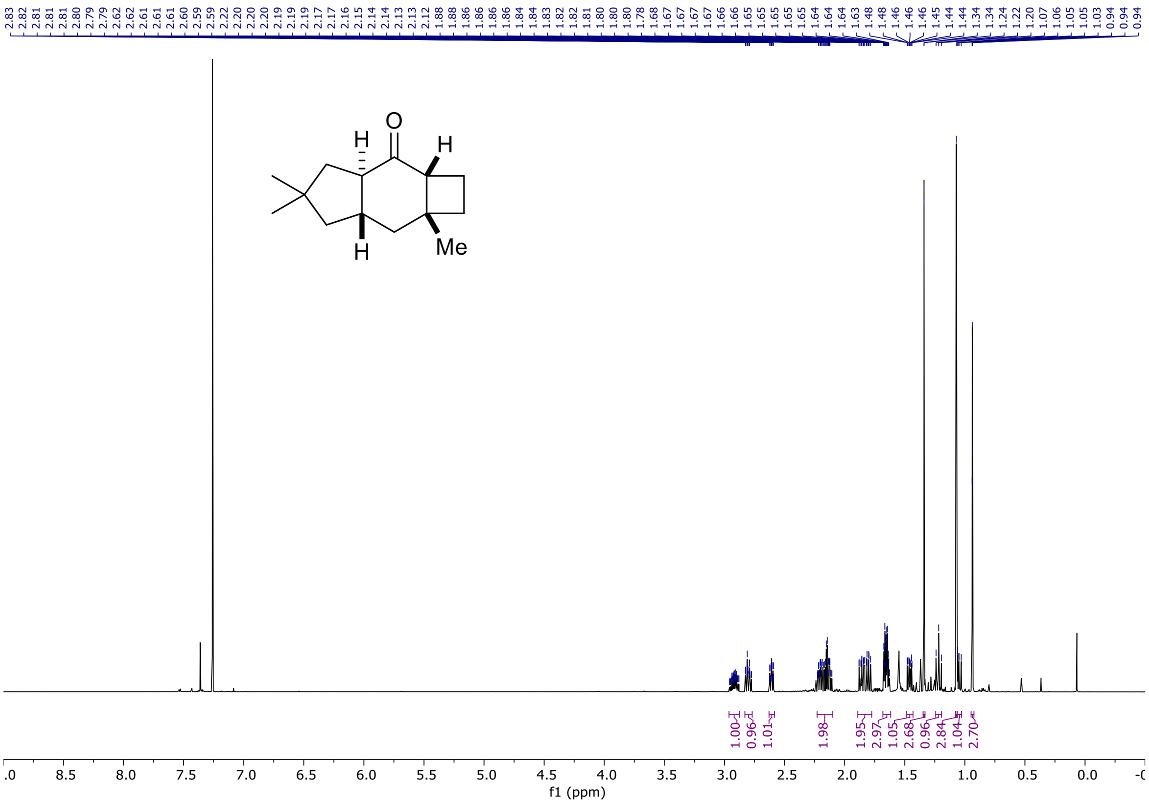

^{13}C NMR (151 MHz, $CDCl_3$) of **S11**

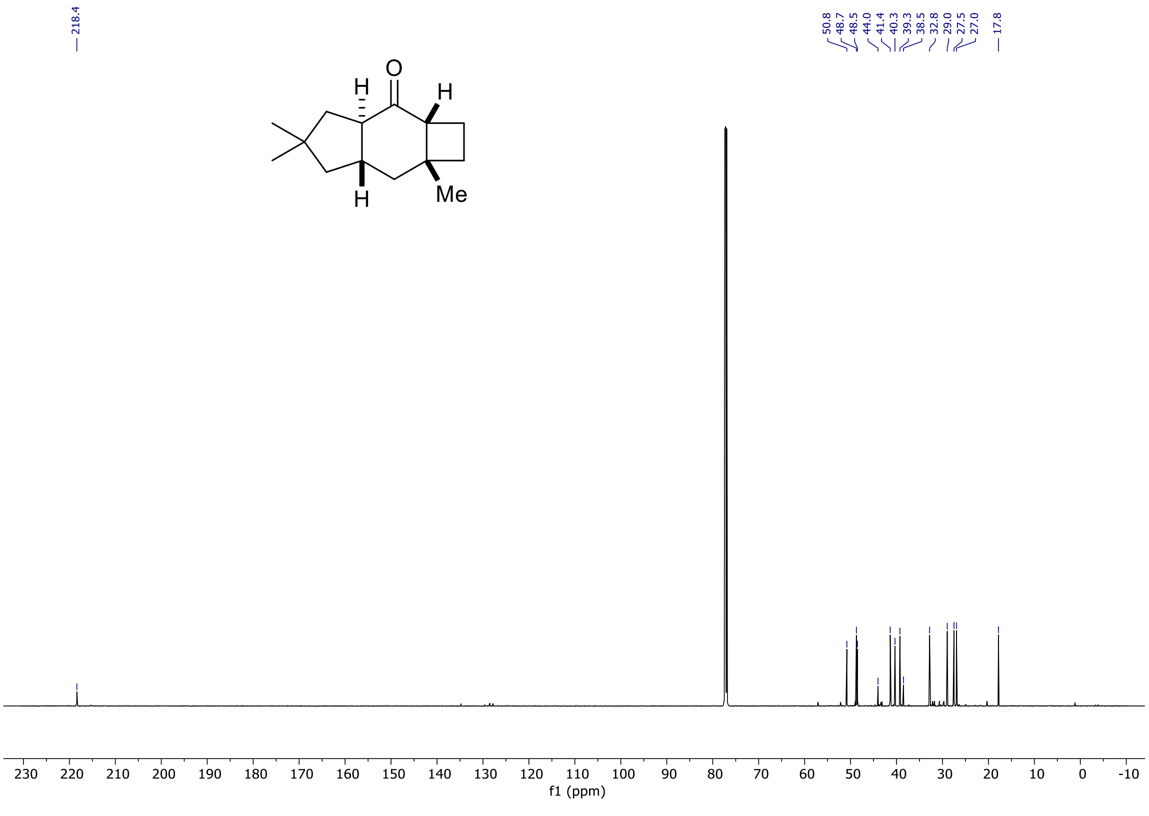

^{1}H NMR (500 MHz, C_6D_6) of **17**:

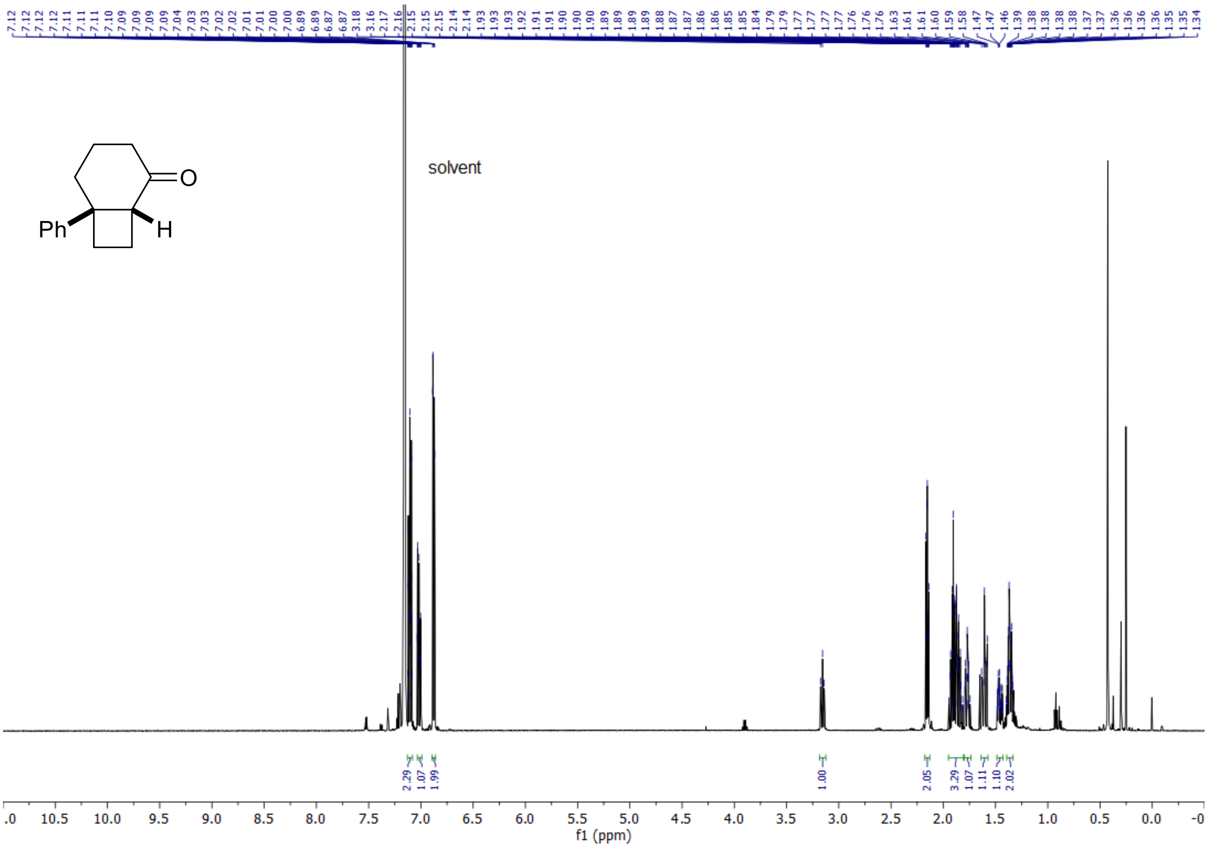

^{13}C NMR (126 MHz, C_6D_6) of **17**:

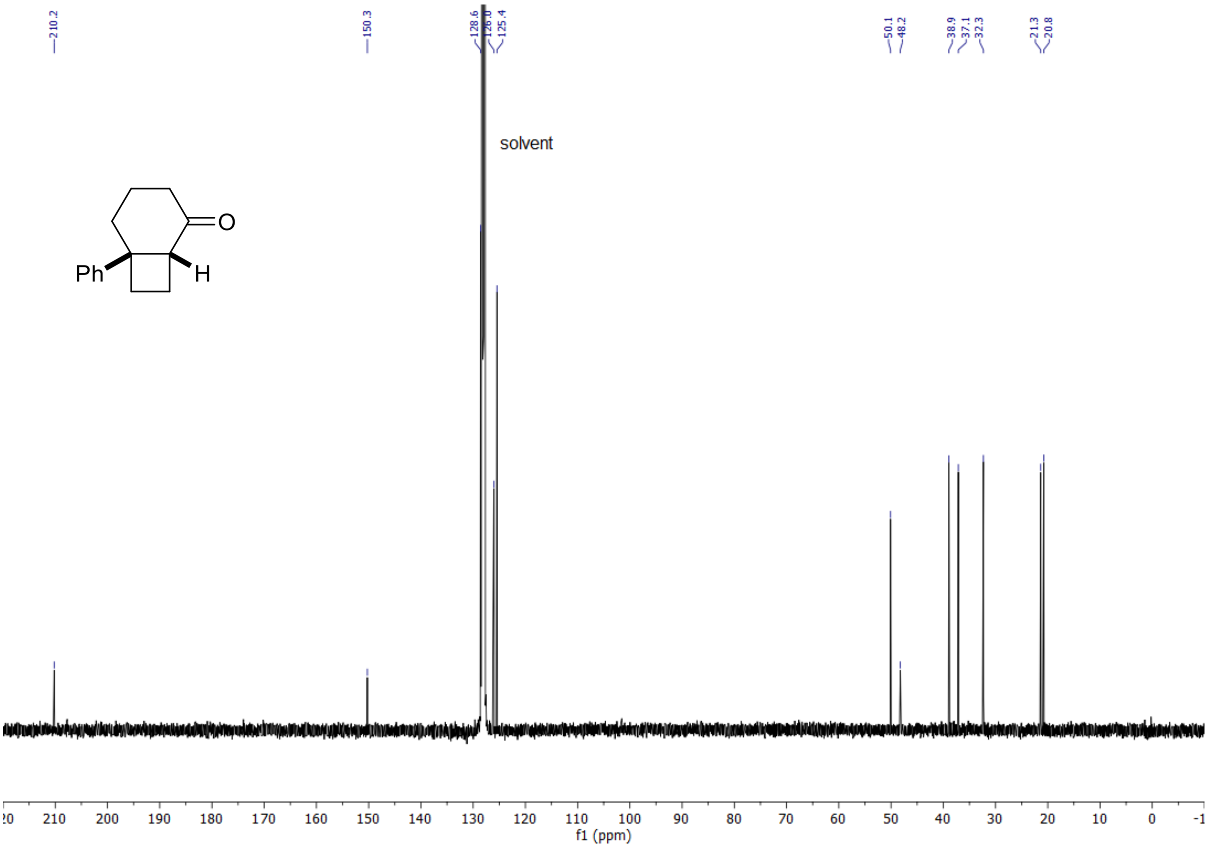

^{1}H NMR (500 MHz, C_6D_6) of **18**:

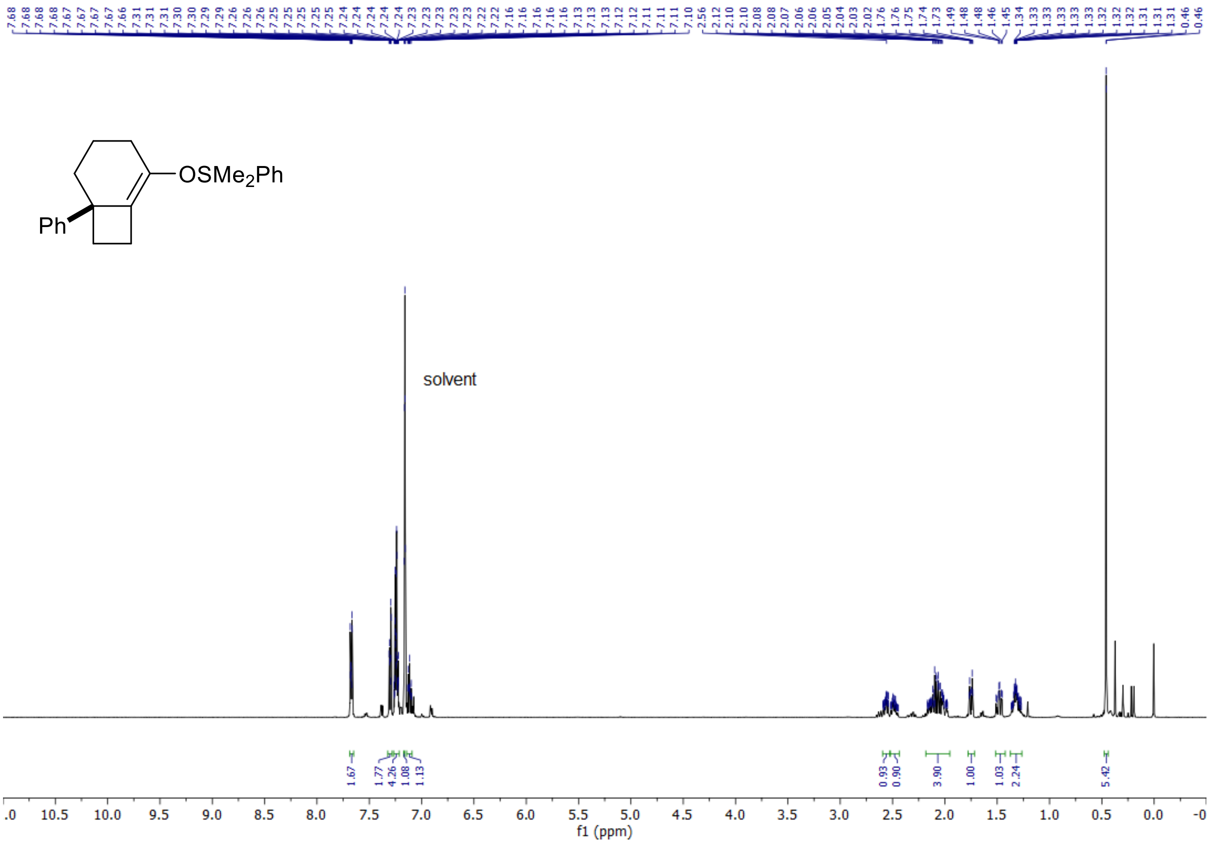

^{13}C NMR (126 MHz, C_6D_6) of **18**:

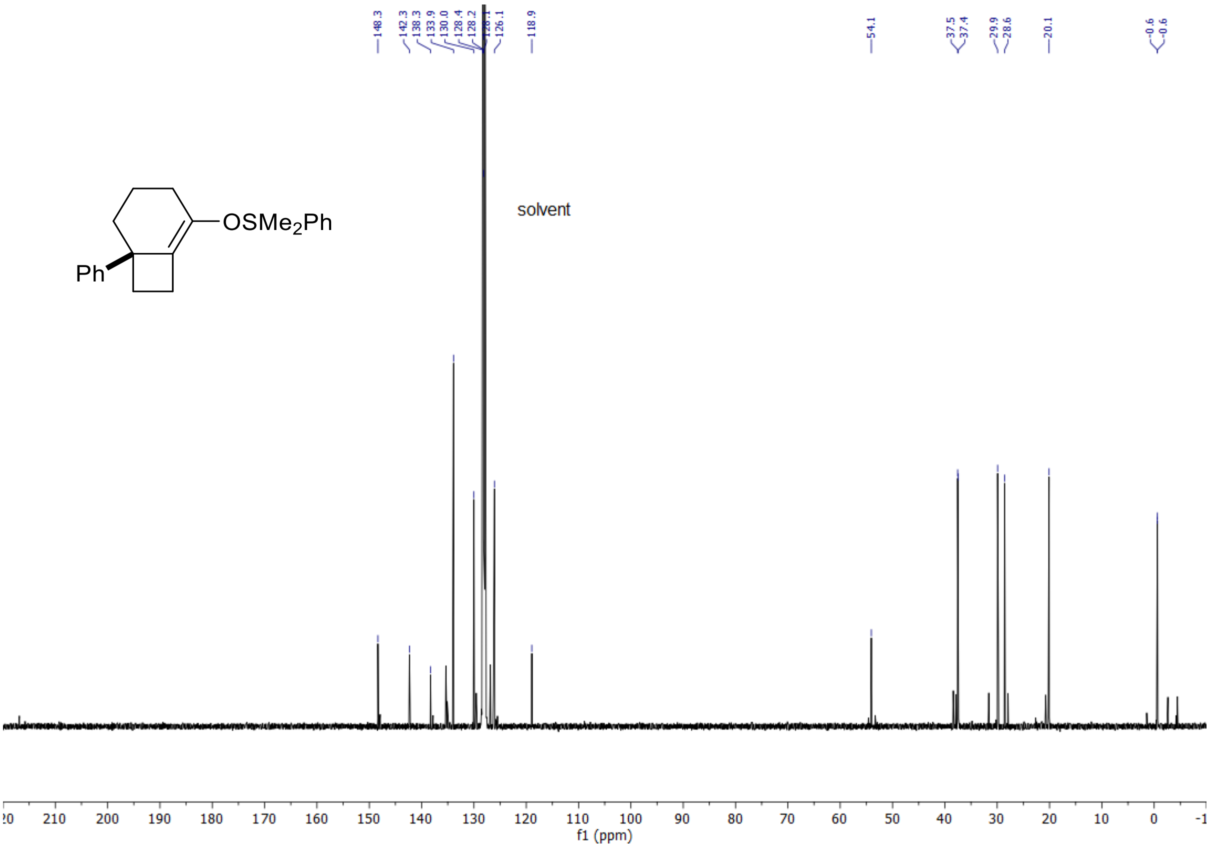

^{1}H NMR (500 MHz, C_6D_6) of **19**

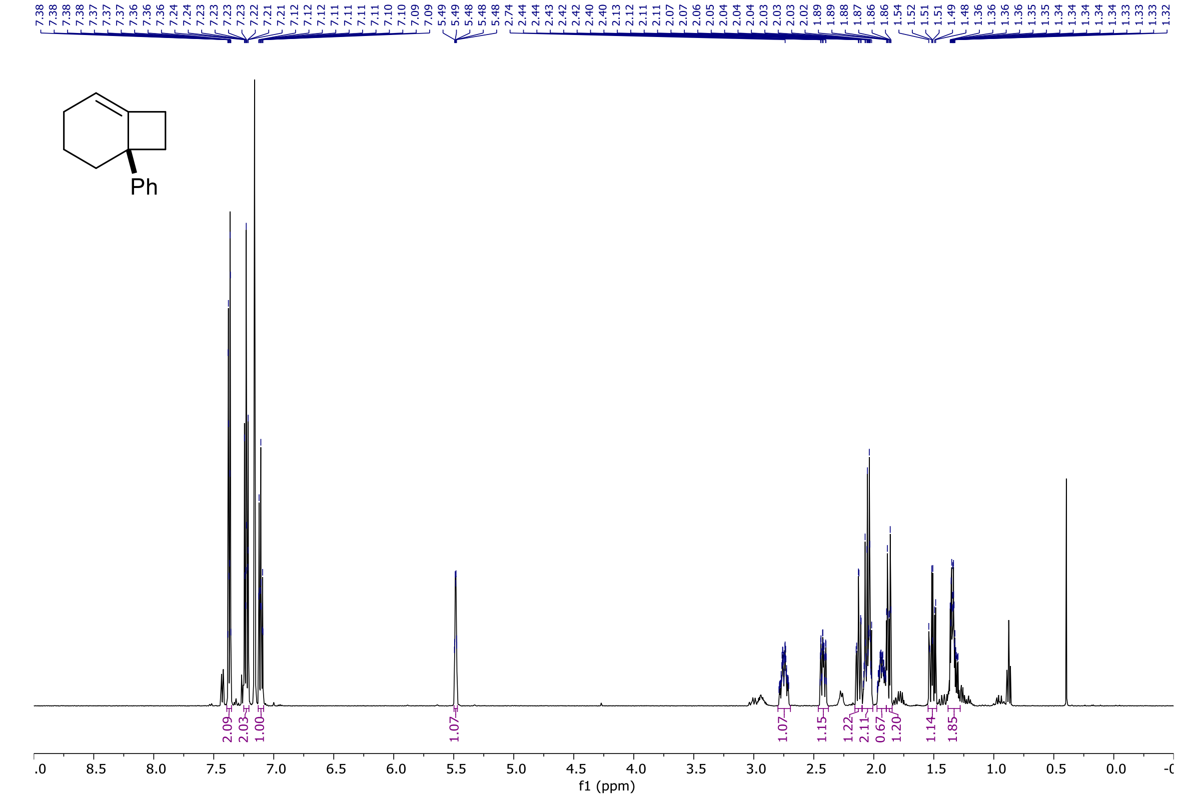

^{13}C NMR (126 MHz, C_6D_6) of **19**

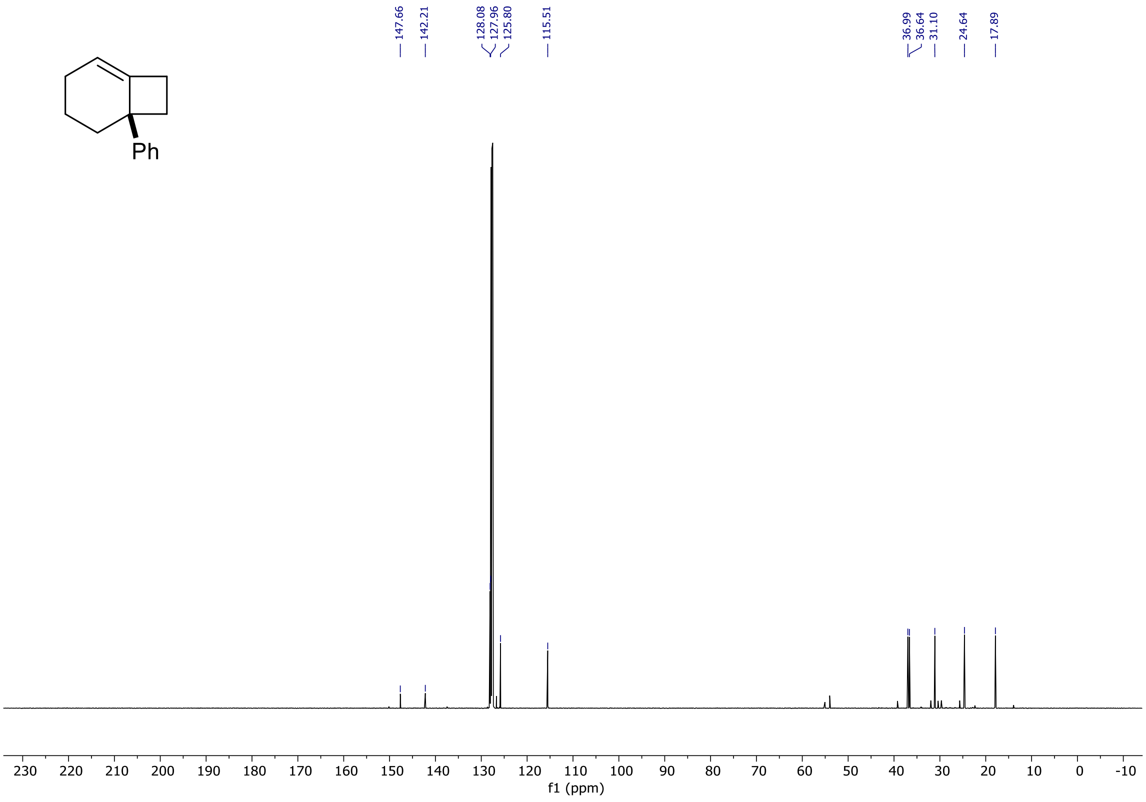

^{1}H NMR (500 MHz, C_6D_6) of **S12**:

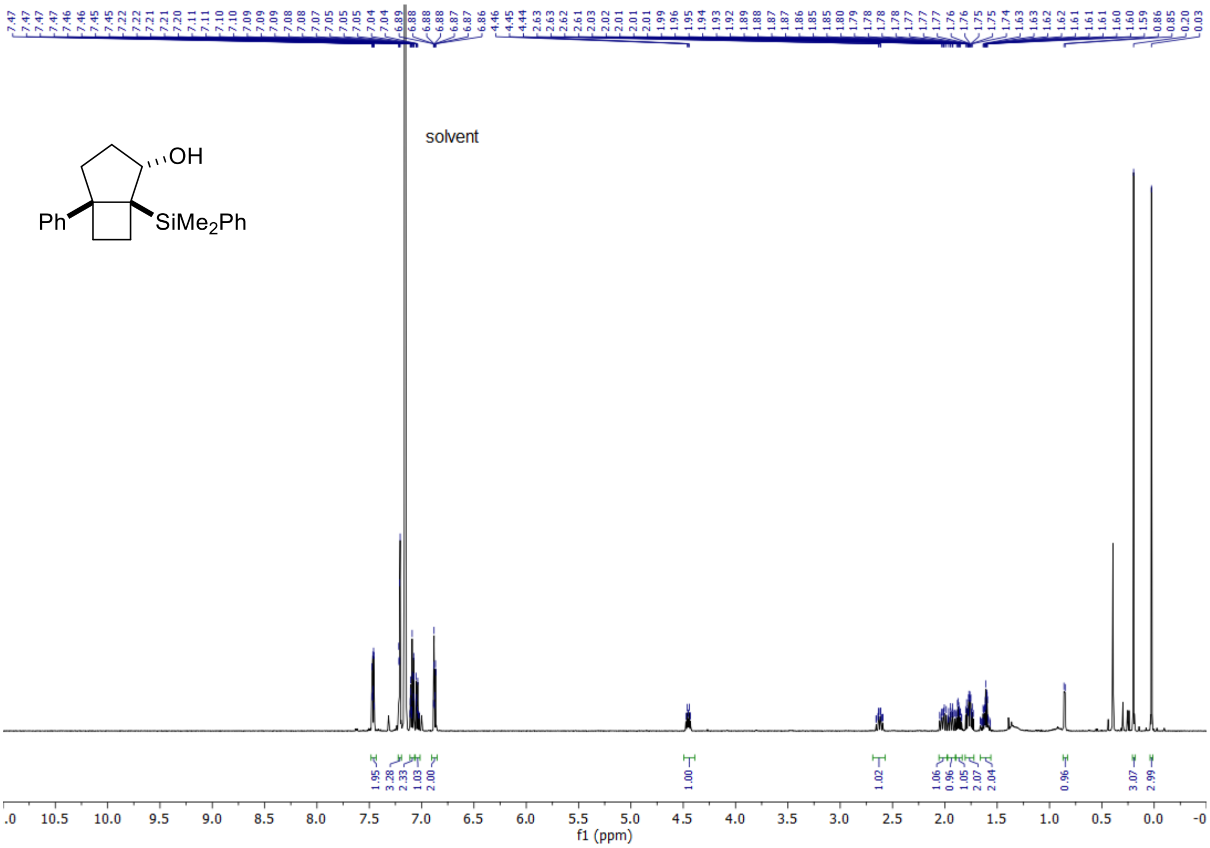

^{13}C NMR (126 MHz, C_6D_6) of **S12**:

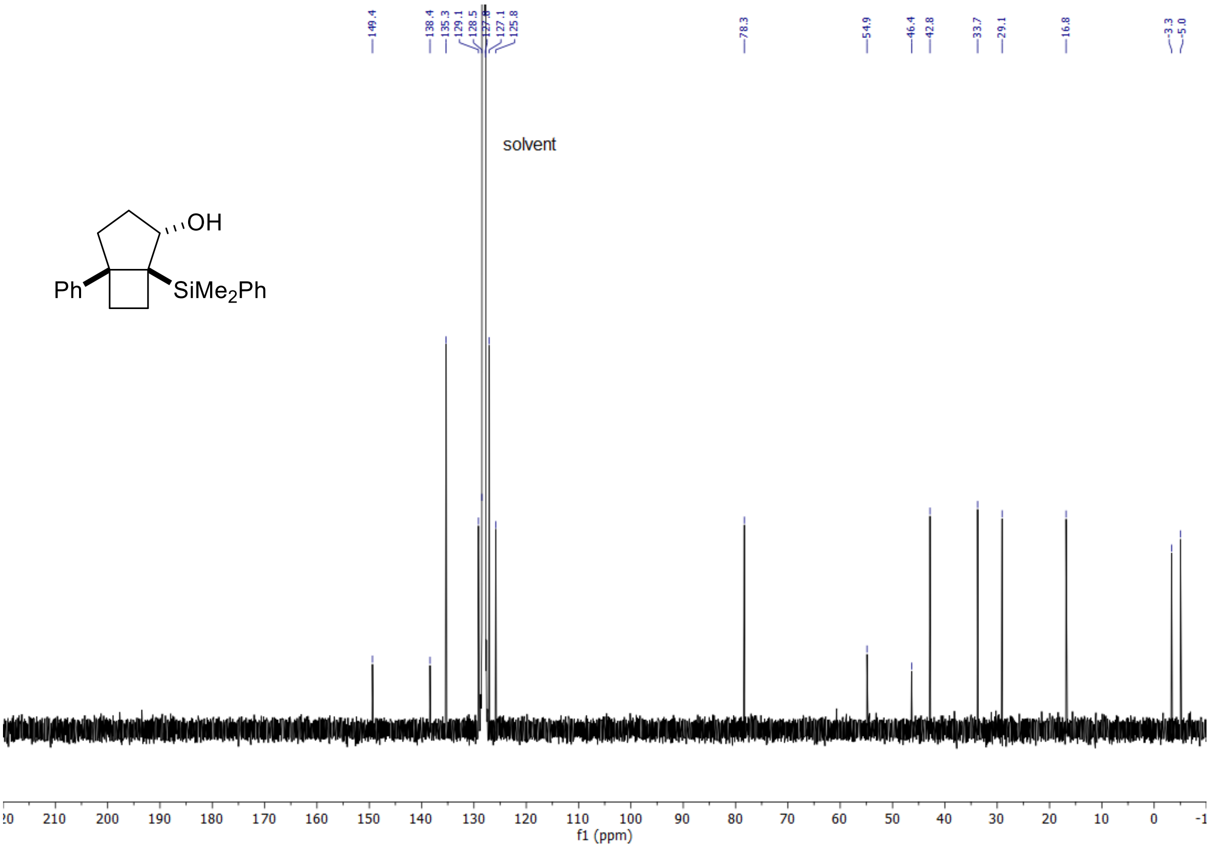

^{1}H NMR (500 MHz, CD_2Cl_2) of **13**:

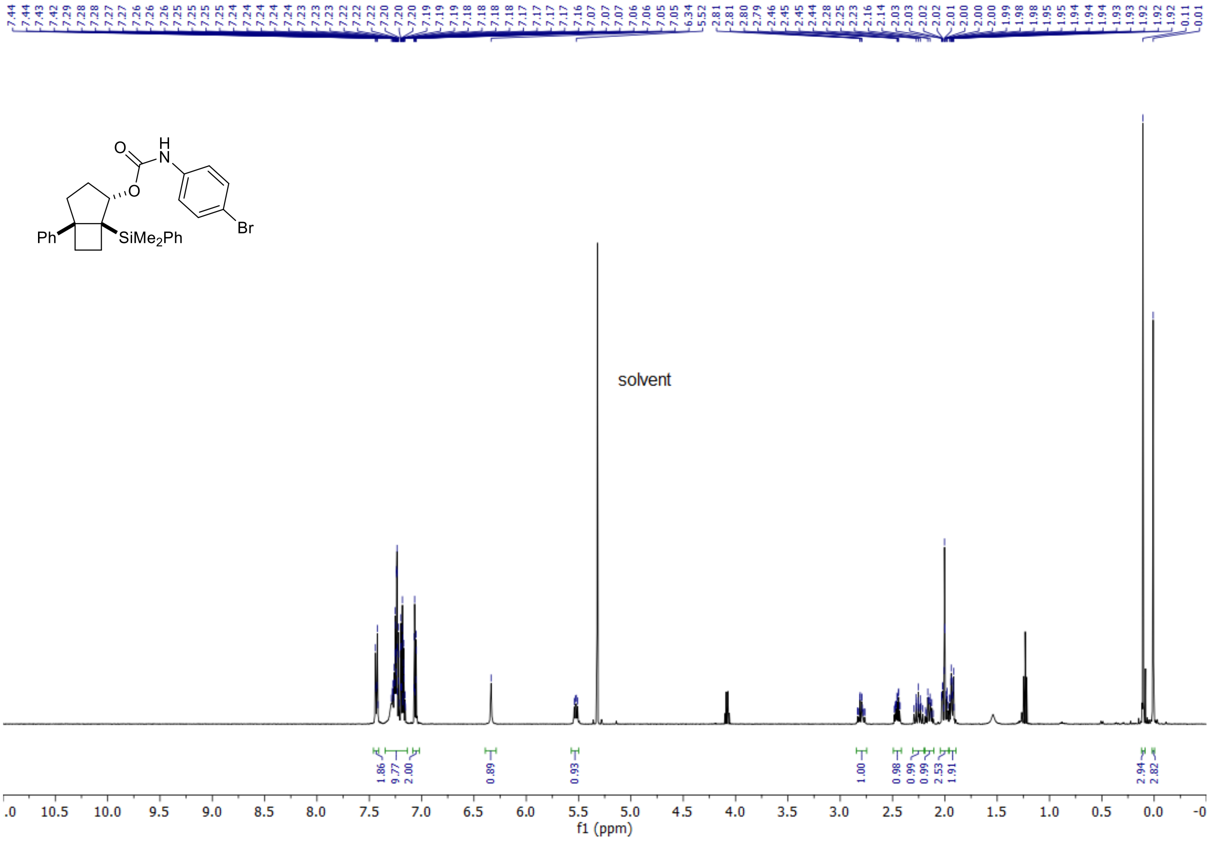

^{13}C NMR (126 MHz, CD_2Cl_2) of **13**:

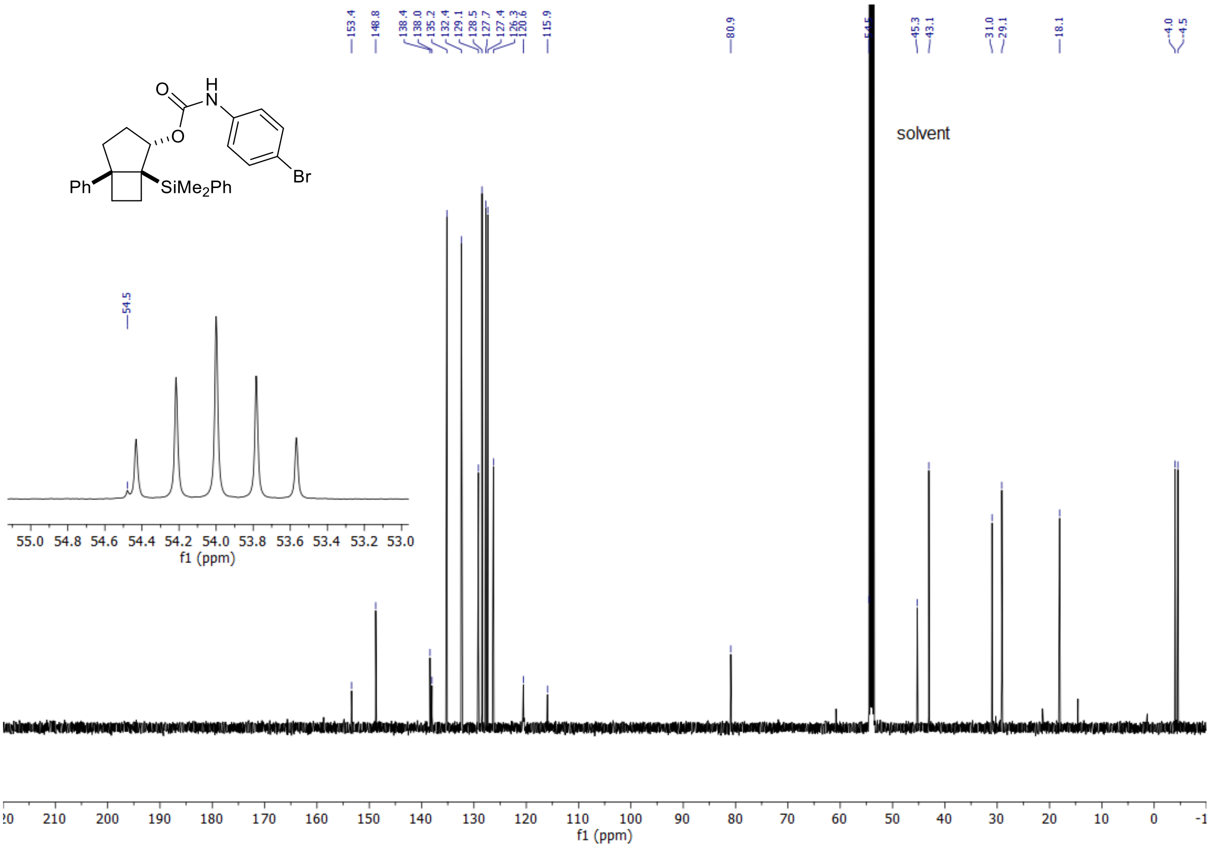

^{1}H NMR (500 MHz, $CDCl_3$) of **S14**:

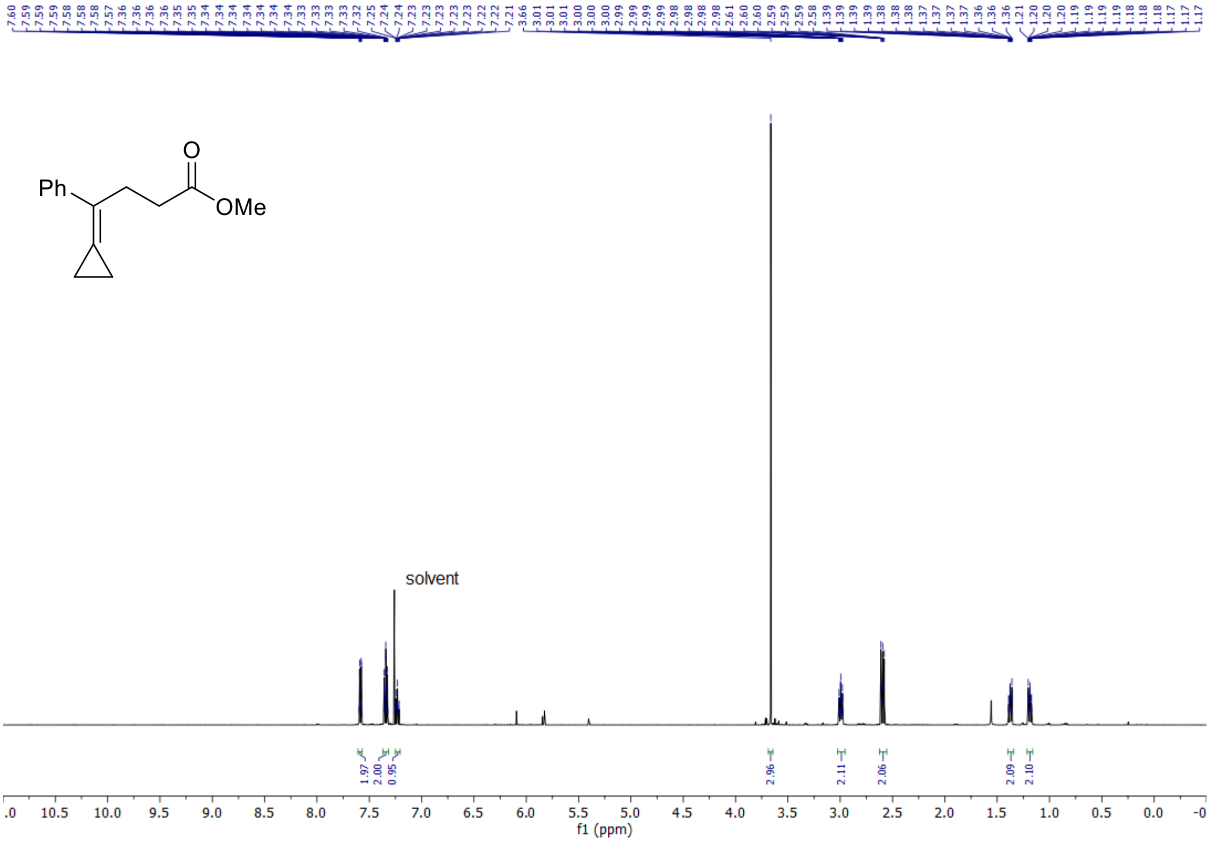

^{13}C NMR (126 MHz, $CDCl_3$) of **S14**:

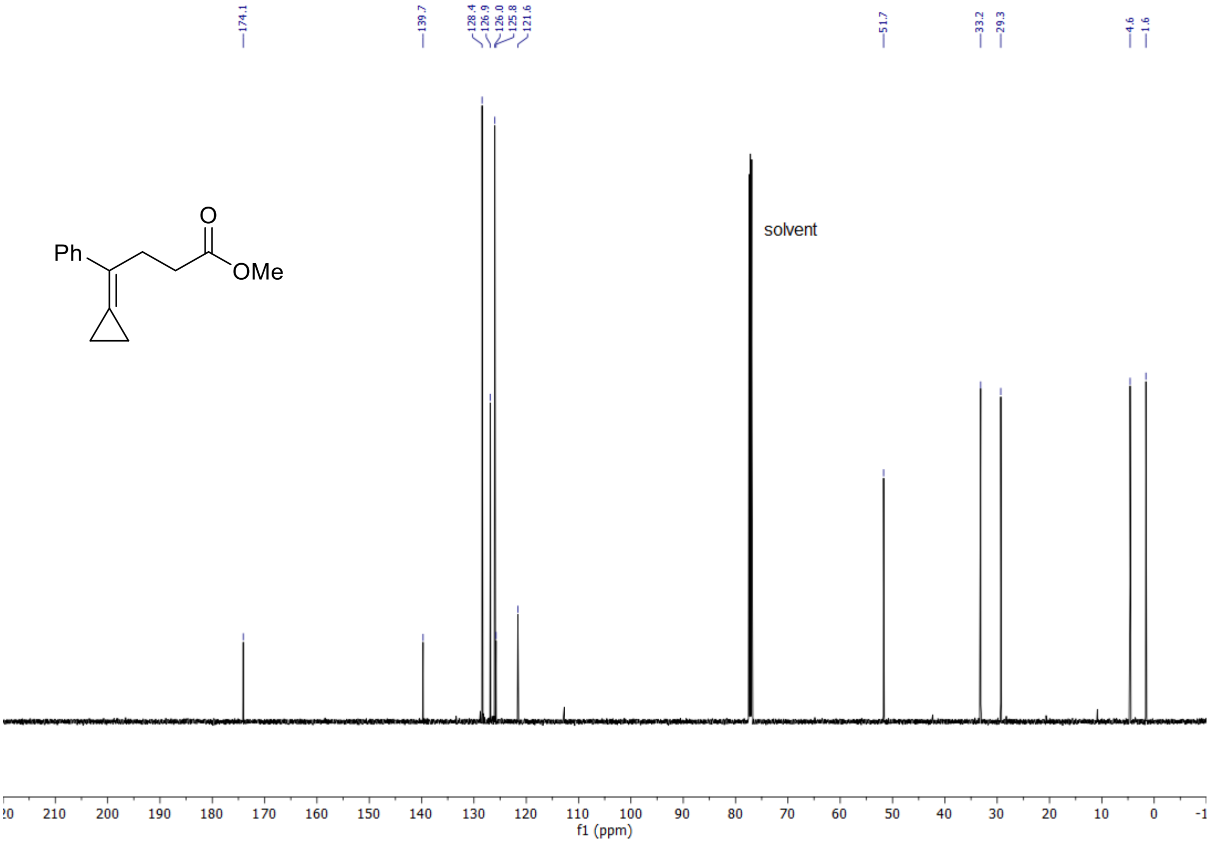

^{1}H NMR (400 MHz, $CDCl_3$) of **S15**:

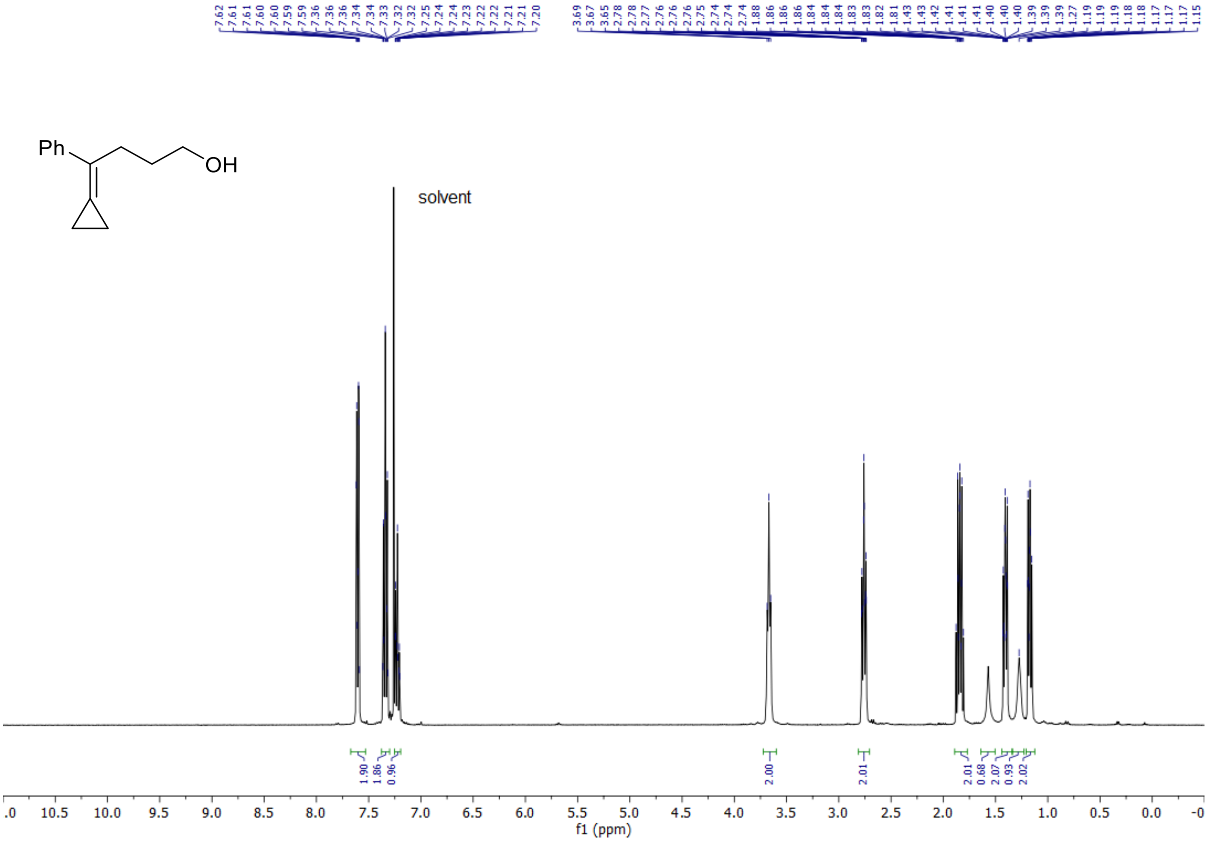

^{13}C NMR (101 MHz, $CDCl_3$) of **S15**:

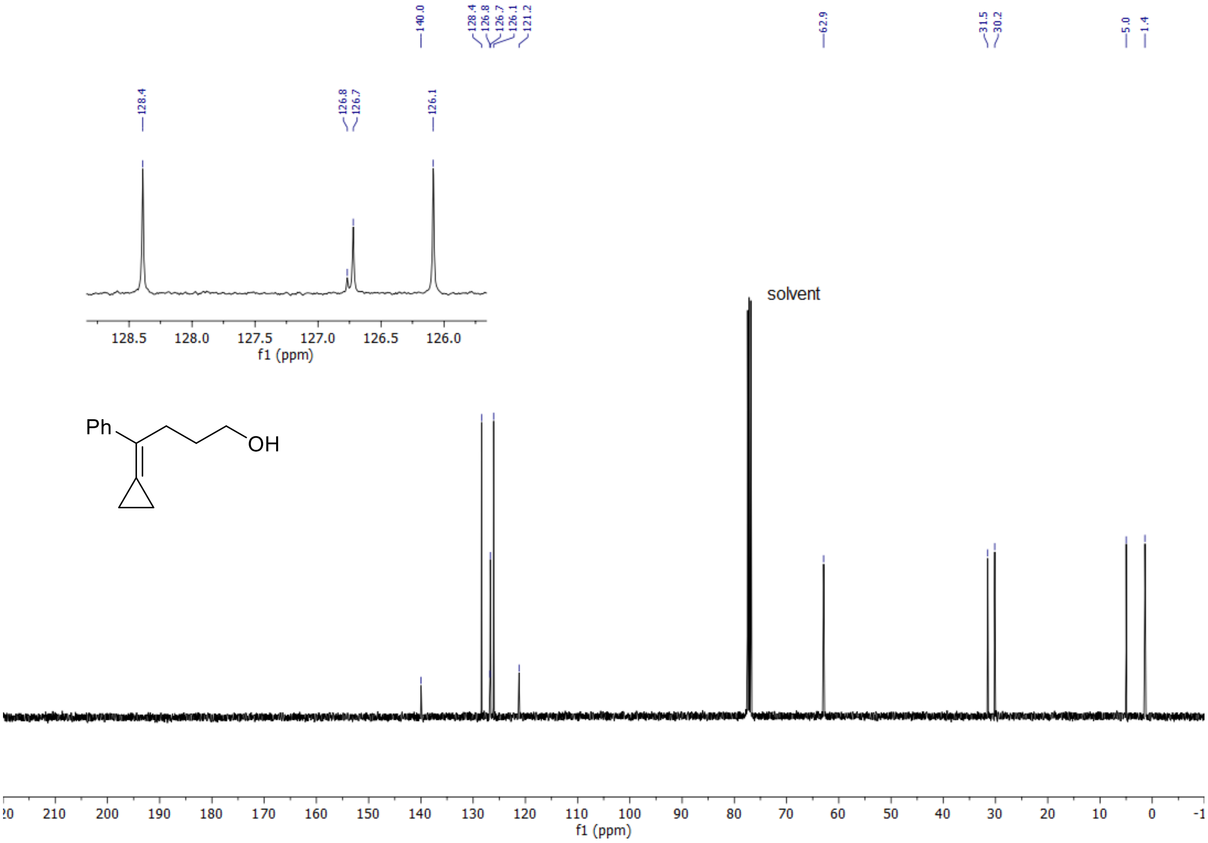

^{1}H NMR (500 MHz, $CDCl_3$) of **S16**:

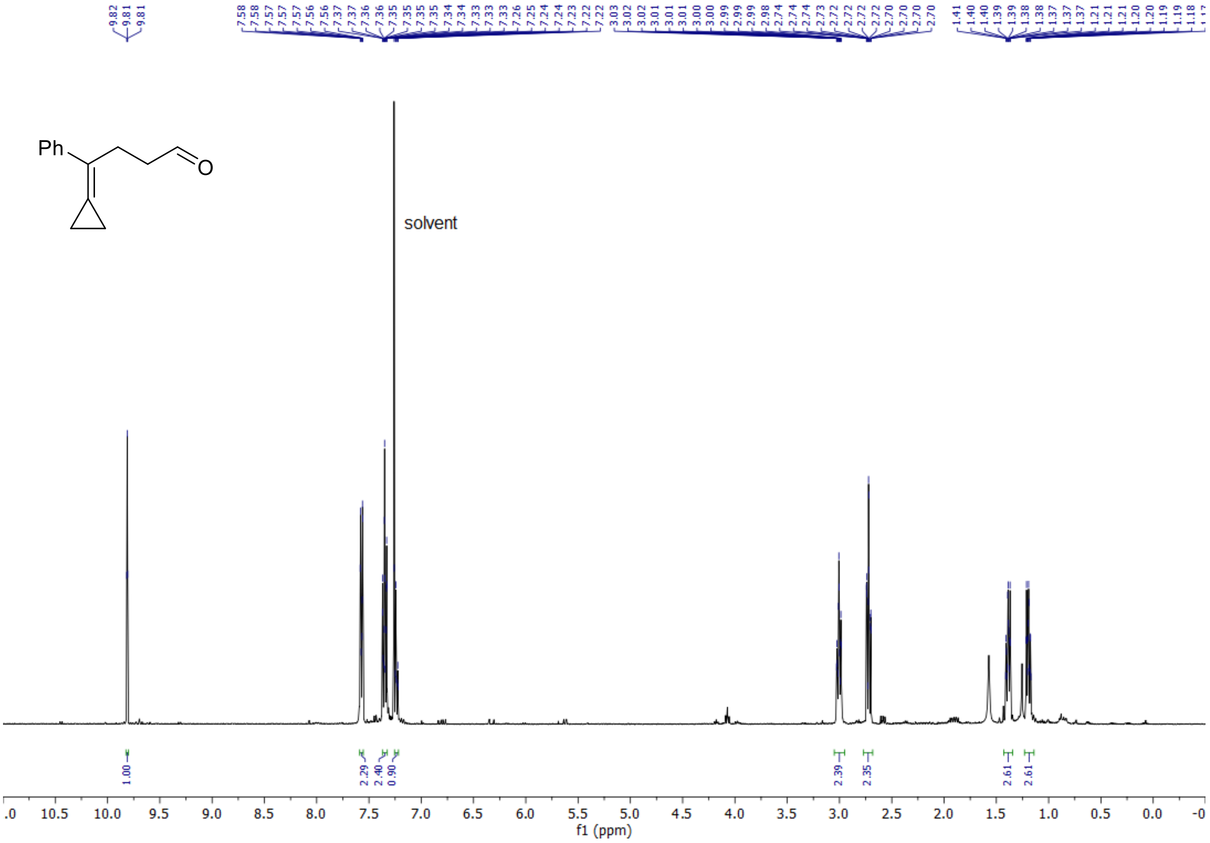

^{13}C NMR (126 MHz, $CDCl_3$) of **S16**:

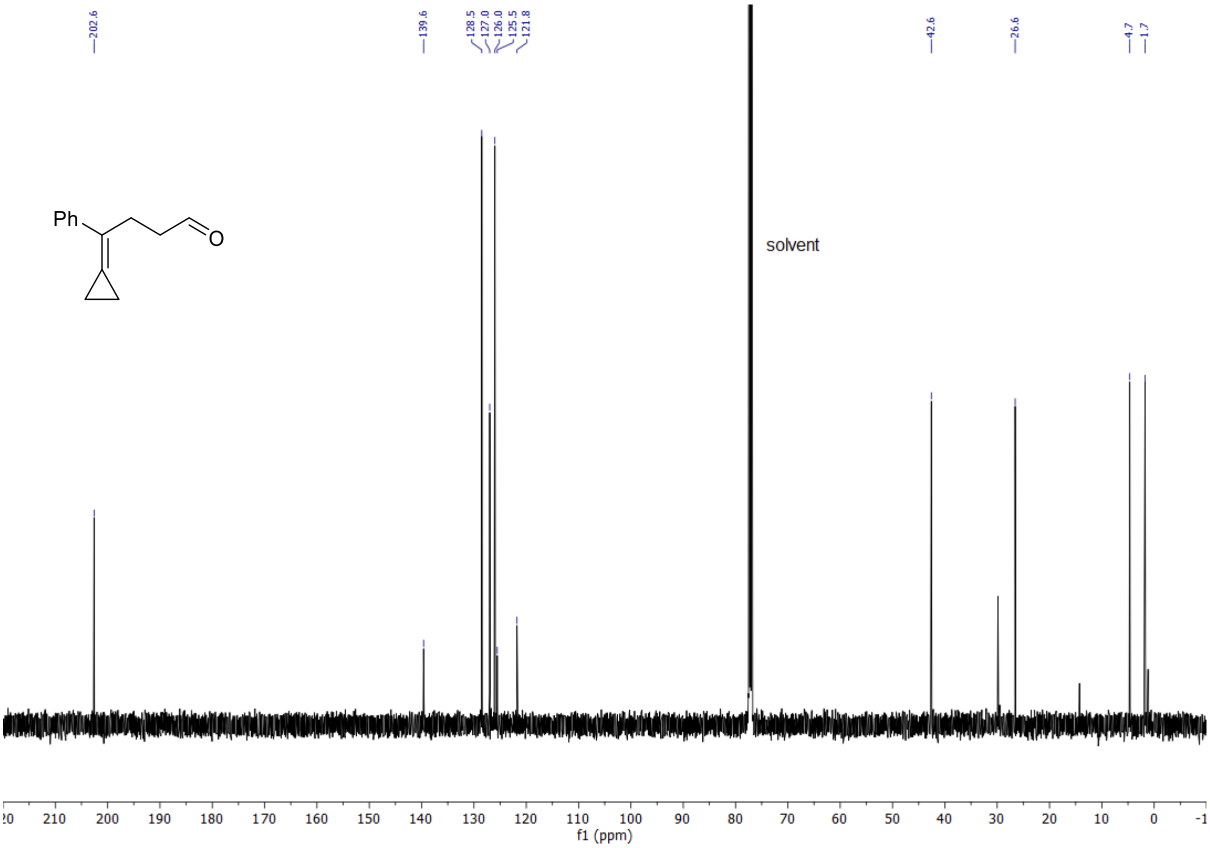

^{1}H NMR (500 MHz, $CDCl_3$) of **S17**:

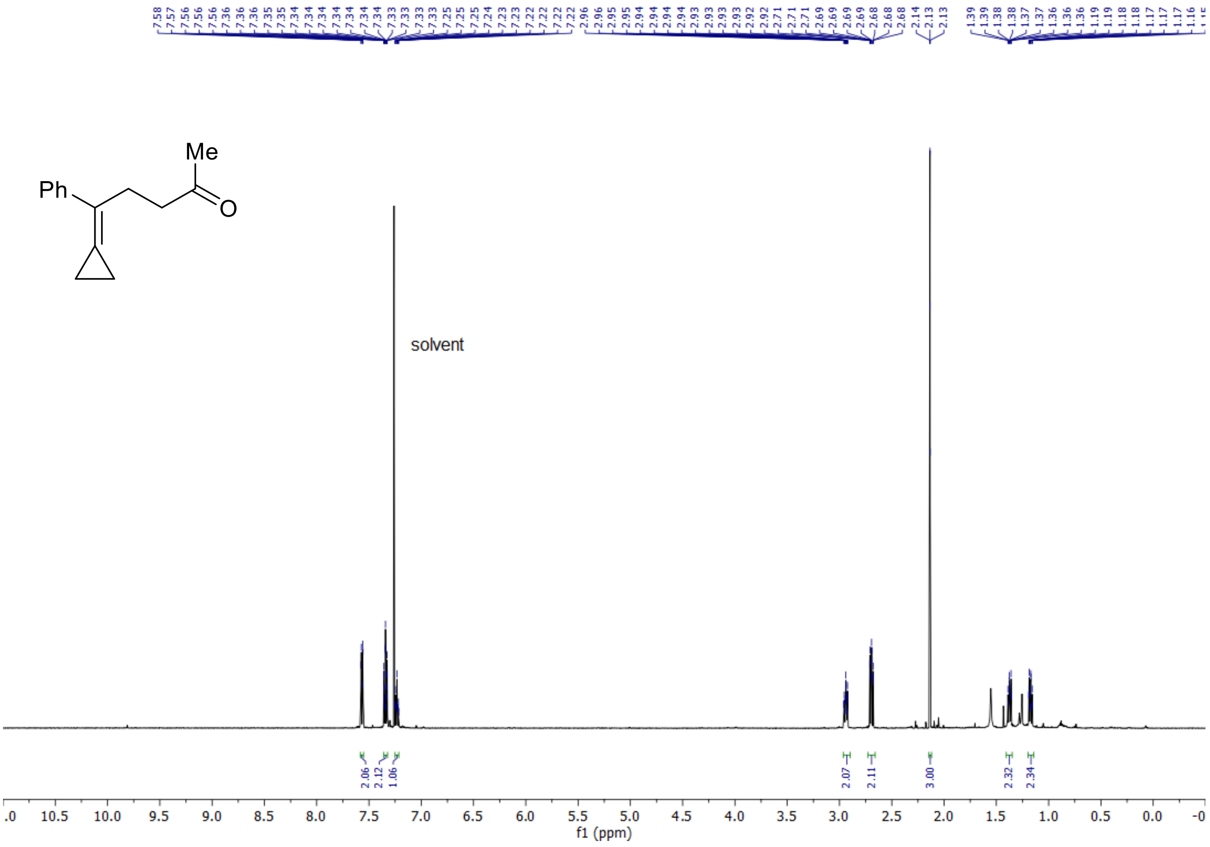

^{13}C NMR (126 MHz, $CDCl_3$) of **S17**:

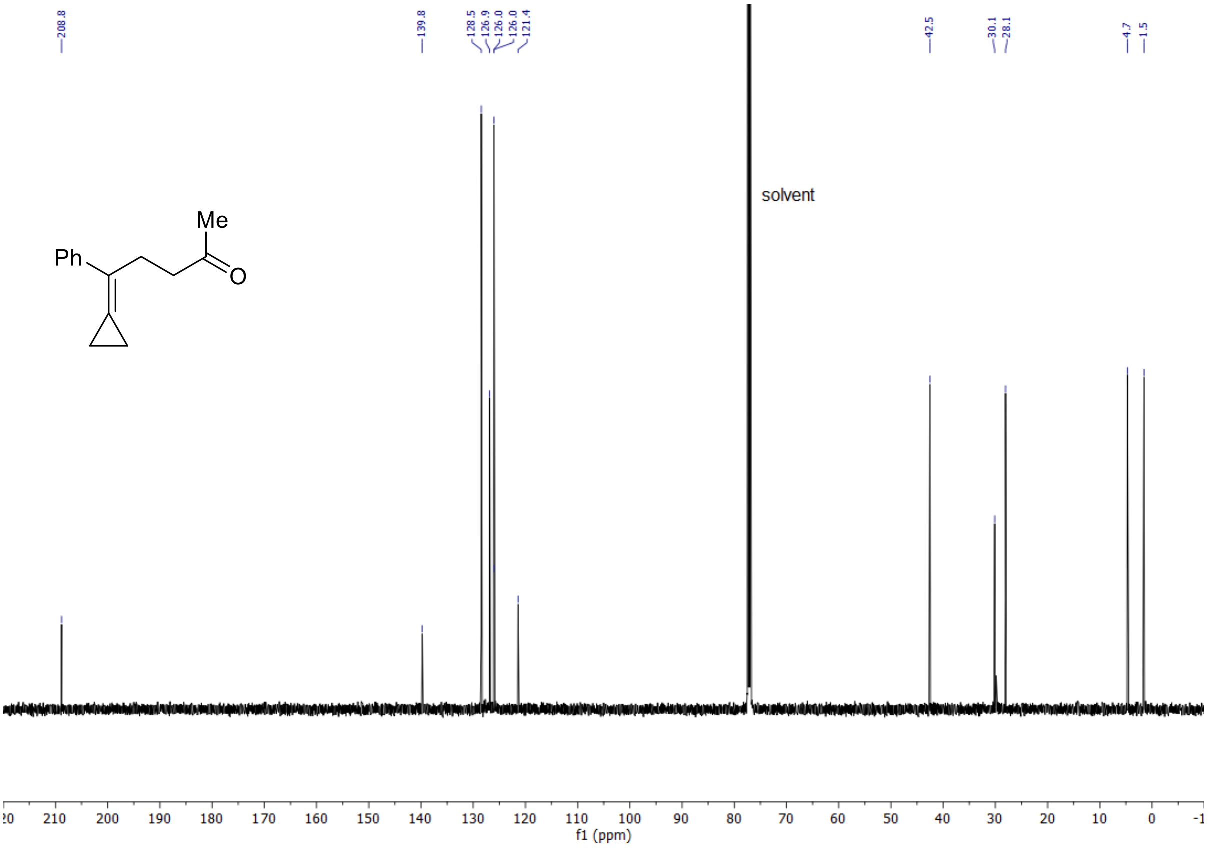

^{1}H NMR (500 MHz, $CDCl_3$) of **S18**:

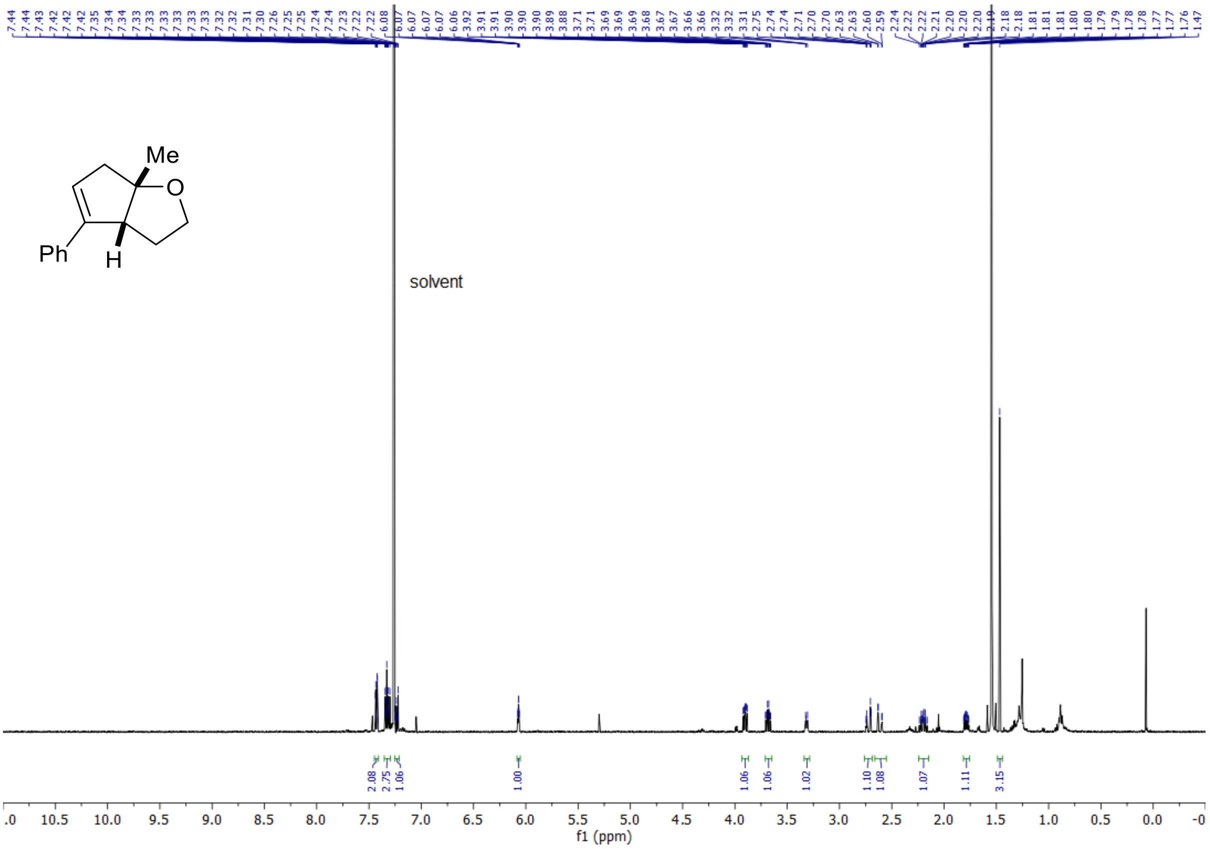

^{13}C NMR (126 MHz, $CDCl_3$) of **S18**:

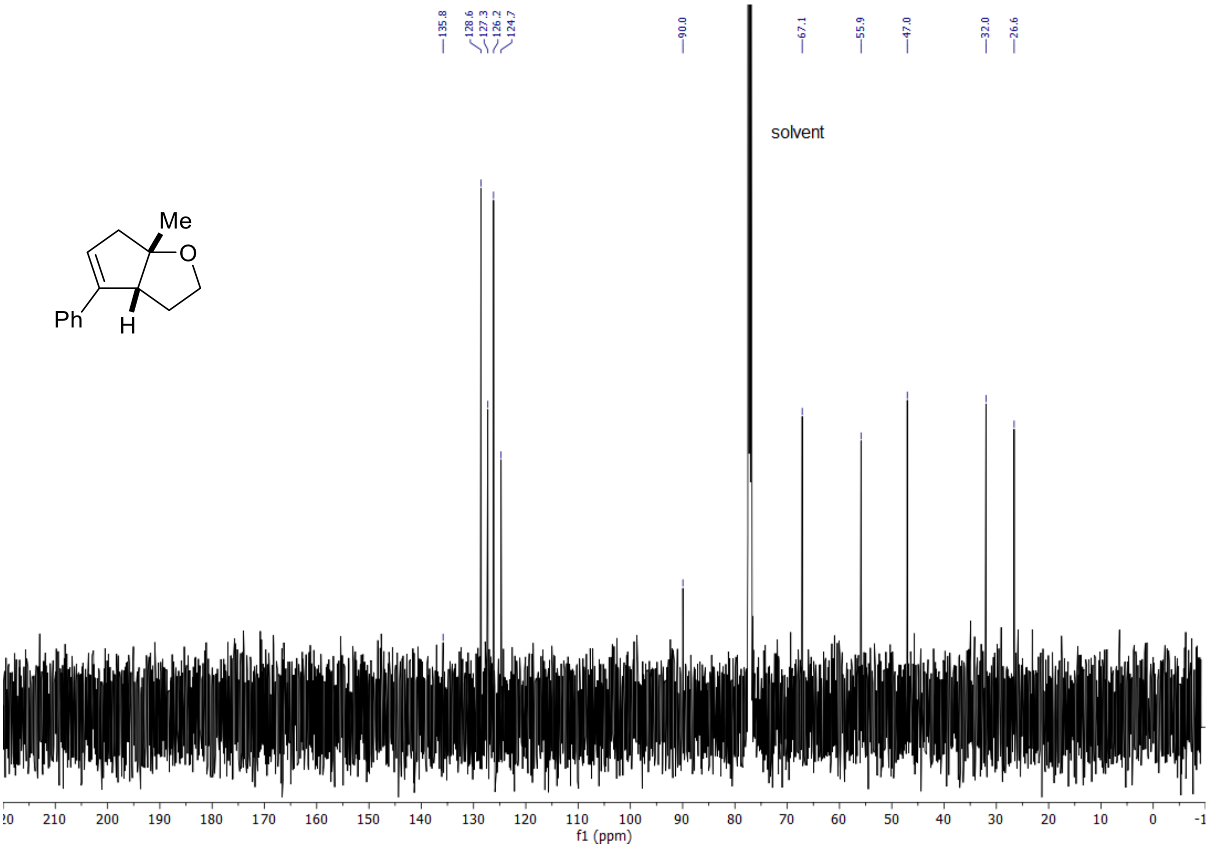

8. X-Ray Crystallographic Data of **13**

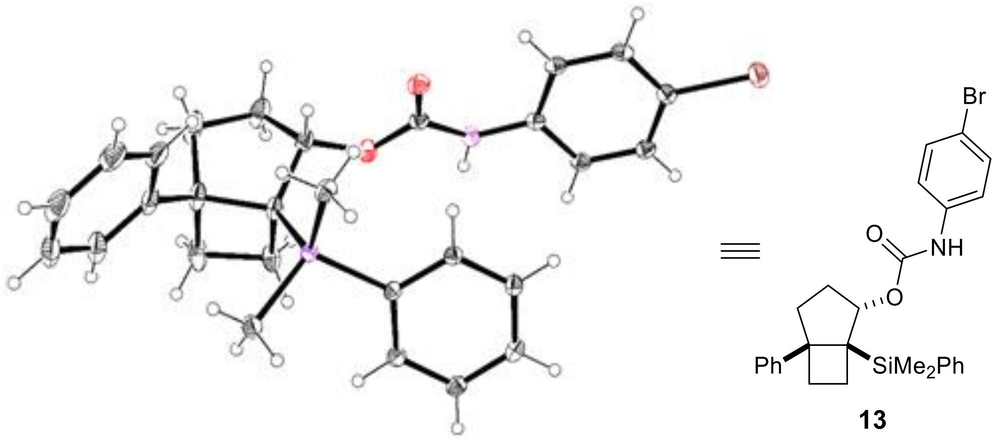

13

CCDC deposition number	To be inserted
Empirical formula	$C_{31}H_{37}BrNO_2Si$
Formula weight	563.61
Temperature/K	100.0(1)
Crystal system	monoclinic
Space group	$P2_1/c$
a/Å	19.00200(10)
b/Å	8.35050(10)
c/Å	18.65040(10)
α/°	90
β/°	106.5460(10)
γ/°	90
Volume/Å^3	2836.83(4)
Z	4
ρ_{calc}g/cm^3	1.320
μ/mm^{-1}	2.589
F(000)	1180.0
Crystal size/mm^3	0.225 × 0.082 × 0.042
Radiation	CuKα (λ = 1.54184)
2Θ range for data collection/°	4.852 to 159.542
Index ranges	-24 ≤ h ≤ 24, -10 ≤ k ≤ 8, -23 ≤ l ≤ 23
Reflections collected	84976
Independent reflections	6092 [R_{int} = 0.0475, R_{sigma} = 0.0173]
Data/restraints/parameters	6092/1/331
Goodness-of-fit on F^2	1.044
Final R indexes [I>=2σ (I)]	R_1 = 0.0347, wR_2 = 0.0922
Final R indexes [all data]	R_1 = 0.0362, wR_2 = 0.0933
Largest diff. peak/hole / e Å^{-3}	0.54/-0.65

Computational analysis

General

All calculations were performed with the Gaussian suite of programs, revision D. Geometries were optimized at the M06-2X/def2-TZVP level of approximation, with the D3 dispersion correction by Grimme and an implicit solvent model for CH2Cl2. All molecular orbitals are visualized at the isosurface value of α = 0.04. In all schemes and figure, hydrogens are omitted for clarity.

Molecular orbitals of 20 and 20'

As stated in the main text, the migration event that occurs in **20** but not in **20'** is enabled by the position of the cyclopropyl ring relative to the plane of the cyclopentane ring. In **20**, the cyclopropyl ring is "pushed upwards" from the cyclopentane, whereas in **20'** the two rings appear to bisect each other (See Scheme 6 in the main text for a visualization). This difference is reflected in the dihedral angle between the pseuso-axial substituent and the respective cyclopropyl carbon.

We believe this geometric effect is due to a destabilizing, out-of-phase interaction between the $\sigma_{C\text{-}Si}$ orbital and a Walsh-like molecular orbital on the cyclopropyl moiety. In order to minimize this interaction, the cyclopropyl ring is pushed upwards, and as a result, the Walsh-like orbital becomes more localized and the overlap with the neighboring empty p orbital increases. In other words, the distortion of the cyclopropyl ring positions C_β in the optimal position for migration to the neighboring carbocation. In contrast, **20'** does not undergo a similar migration and, accordingly, a similar interaction is not observed in **20'**. For this conformer, the $SiMe_3$ group is in the equatorial position and thus a similar overlap is geometrically precluded. Visual inspection of the HOMO of **20'** shows that it does not contain a similar interaction with the $\sigma_{C\text{-}O}$ orbital of the OBF3 group in the axial position (Figure S1).

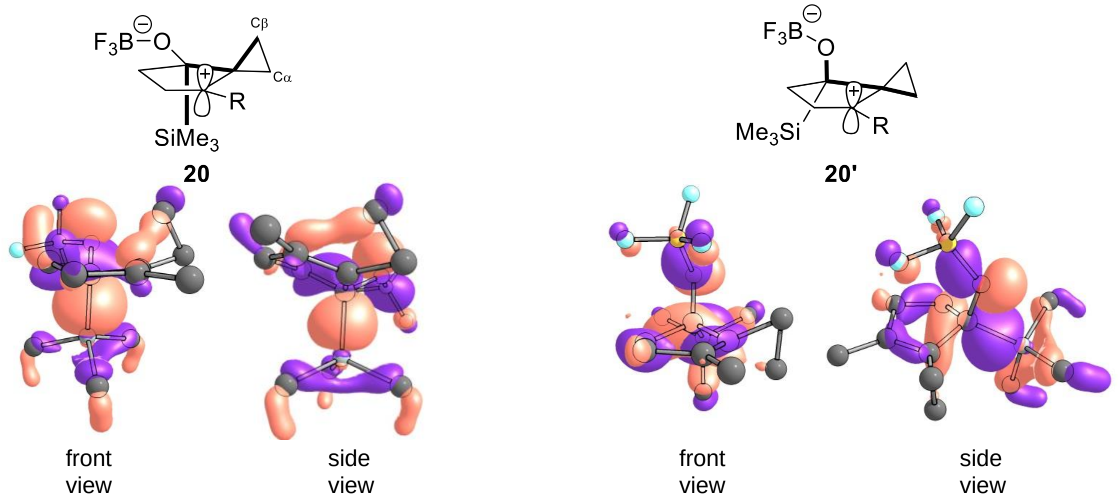

*Figure S1. HOMOs of conformers **20** and **20'**.*

Due to the lower energy of σ_{C-O} orbital, the preferred interaction is with the high-lying lone pairs on the oxygen in the pseudo-axial position. Thus, for **20'**, we find the Walsh-like orbitals in the HOMO-4 and HOMO-5, where they appear to interact with one of the lone pairs on the oxygen and with the two carbons neighboring the spiro carbon (Figure S2). As a result, there is no driving force for distortion of the cyclopropyl ring away from the pseudo=axial group.

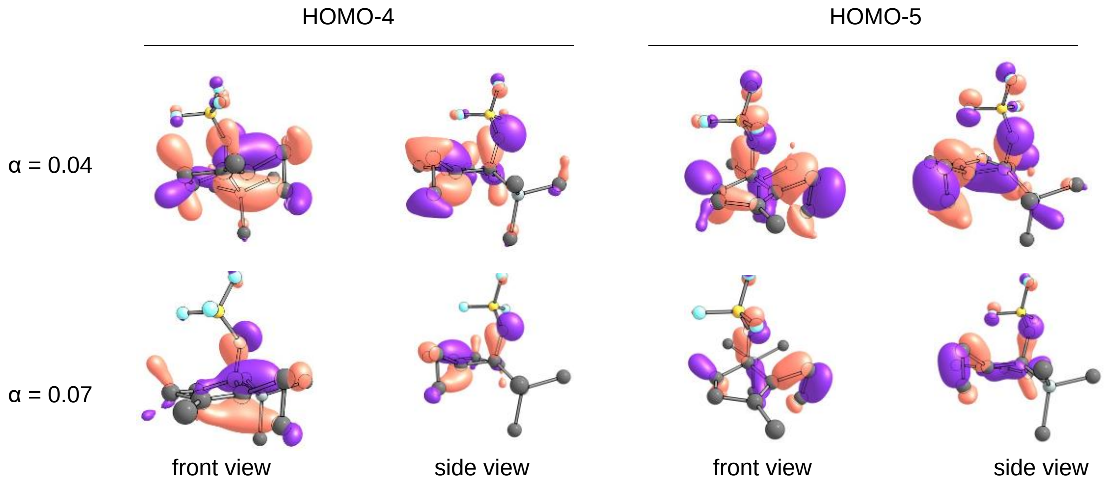

Figure S2. HOMO-4 and HOMO-5 of 20', visualized at two isosurface values, α = 0.04 (top) and α = 0.07 (bottom), for better clarity.

Model cases demonstrate geometric effect

To validate our interpretation of the orbital interactions taking place in **20** and **20'**, we studied two model conformers, where the $SiMe_3$ and OBF_3 groups were substituted with SiH_3 and OH, respectively. Model A, which is analogous to **20'**, has a dihedral angle of ~110° (Figure S3 left; compared to ~114° for **20'**). Model B has a dihedral angle of 129° (Figure S3 right; compared to ~157° for **20**). This indicates that model A and **20'** have similar orbital interaction, while model B does not have exactly the same orbital interactions as **20**. This is consistent with our hypothesis above, because it is expected that the σ_{C-Si} orbital would be higher in energy for $SiMe_3$ than it would be for SiH_3, due to the inductive effect of the methyls. Hence, in the $SiMe_3$ case, there would be a stronger interaction with the Walsh-like orbital of the cyclopropyl ring, causing the distortion we observe.

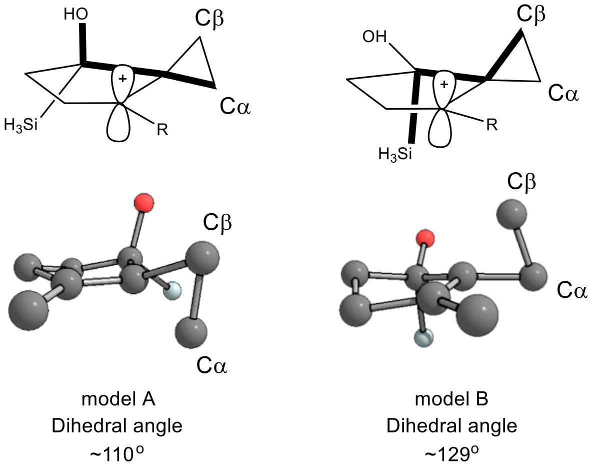

Figure S3. Top: Schematic depictions of model systems A and B and the relevant dihedral angles. Bottom: Optimized geometries of model systems A and B.

At the same time, model B allows us to probe the importance of the dihedral angle further. Though the system optimizes to a structure with a smaller dihedral angle, we could investigate what happens when this angle is forced to be greater. Thus, we subjected model B to a constrained scan, in which the dihedral angle was increased in increments of 5°, while allowing the rest of the molecule to relax at each step. As the dihedral angle approached 175°, the molecule underwent spontaneous rearrangement and ring opening to a cyclobutyl-containing system (Figure S4).

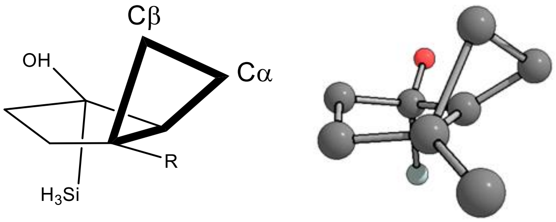

Figure S4. Left: Schematic depiction of model B following spontaneous rearrangement due to increased dihedral angle. Right: optimized geometry of the rearranged structure.

This corroborates our rationalization above, that the distortion of the cyclopropyl is the necessary condition for the migration to occur, and that this migration is very facile once the migrating carbon is in place.

The scan computations also allowed us to investigate the change to the HOMO of this model as the dihedral angle is increased. As the scan progressed, it showed again the orbital interactions that we believe are the main drivers: the Walsh-like orbital on the cyclopropyl moiety has an out-of-phase interaction with the $\sigma_{C\text{-}Si}$ orbital and an in-phase combination with the empty p orbital on the cationic carbon. As the aforementioned dihedral angle is increased, the orbital on the cyclopropyl ring appears to localize and become more sigma C-C in nature. Additionally, the interaction with the empty p orbital increases, which is also reflected in the movement of C_β close to this carbon. These are visualized in Figure S5.

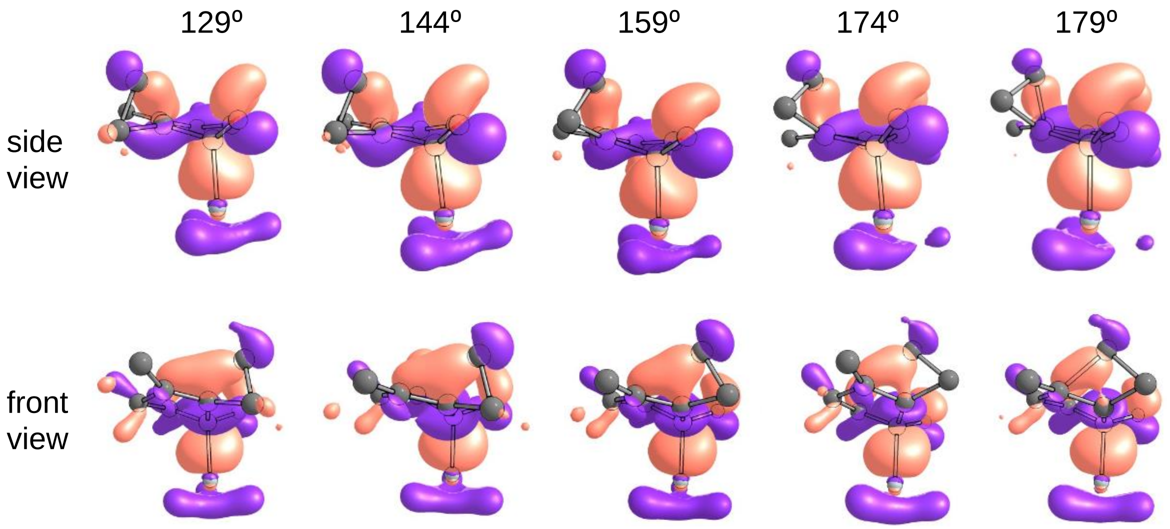

Figure S5. VIsualization of the HOMO of model B as the dihedral angle is increased. Top: side view. Bottom: front view. The visualizations show the change of the Walsh-like orbital to a more localized $\sigma_{C\text{-}C}$, concurrent with increasing interaction of C_β with the carbocationic center and breaking of the bond between C_β bond and the spiro cyclopropyl carbon.

Overall, the model systems highlight the importance of the dihedral angle in creating the necessary overlap with the empty p orbital, which drives the migration event. As mentioned above, model B has smaller dihedral angle, most likely because the destabilizing interaction with the $\sigma_{C\text{-}Si}$ orbital is not as strong when the group is SiH_3. To verify this, we studied another system, model B' (Figure S6), where we reintroduced only the $SiMe_3$ group. In doing so, the dihedral angle increased to 149°, significantly closer to the ~157° observed for **20**.

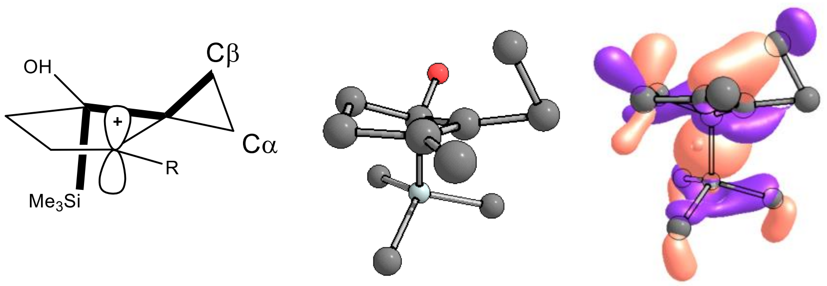

*Figure S6. Left: Schematic depiction of model B'. Middle: Optimized geometry of model B'. Right: Visualization of the HOMO of model B', showing the same interaction as described above for the actual system, **20**, and model B.*

Optimized geometries and energies

The geometries of all systems and their zero-point corrected energies are given below. All energies are zero-point corrected and given in Hartrees. All coordinates are given in Å.

20

ZPE	-1120.20228700	Hartree	
6	0.469731	-0.429312	-1.687082
6	1.986606	-0.220172	-1.782405
6	0.101047	-0.205547	-0.197335
6	1.429304	-0.490967	0.488645
6	2.498316	-0.428165	-0.39734
6	3.942777	-0.418619	-0.083888
8	-0.837733	-1.123486	0.28914
6	1.739648	-2.069178	0.815021
6	1.55539	-1.036139	1.862111
14	-0.327191	1.642925	0.214425
1	-0.087203	0.209923	-2.368769
1	0.219082	-1.464934	-1.926313
1	2.498872	-0.863305	-2.499167
1	2.250068	0.809108	-2.053421
1	4.485125	-1.09567	-0.744114
1	4.304715	0.591581	-0.300013
1	4.159439	-0.648515	0.956267
1	0.88165	-2.638452	0.484852
1	2.719837	-2.496301	0.666566
1	0.606077	-1.057954	2.380834
1	2.42286	-0.767977	2.44934
6	-1.771926	2.271582	-0.782688
1	-1.86975	3.347018	-0.613903
1	-1.612045	2.10868	-1.849729
1	-2.697645	1.777973	-0.496177
6	-0.626721	1.762327	2.055515
1	0.281391	1.54325	2.621138
1	-0.945454	2.773579	2.317374
1	-1.407577	1.064621	2.359652
6	1.16844	2.695903	-0.233914
1	2.098954	2.345391	0.218648
1	1.308425	2.766663	-1.314314
1	0.994	3.709707	0.135471
5	-2.239109	-1.083975	-0.091564
9	-2.969024	-0.218861	0.749336
9	-2.397464	-0.647975	-1.419672
9	-2.754446	-2.377371	0.045744

20'

ZPE	-1120.20394800	Hartree	
6	0.065229	0.04702	-1.767419
6	1.418853	0.764423	-1.804363
6	-0.333654	-0.02199	-0.27167
6	0.40326	1.208619	0.282448
6	1.46289	1.55811	-0.559481
6	2.54012	2.509178	-0.270534
8	0.127502	-1.183669	0.392107
6	-2.75151	-1.702615	-0.937667
6	-2.635955	-0.256095	1.765863
6	-3.107697	1.319882	-0.8538
6	0.592338	1.393361	1.812604
6	-0.237212	2.36334	1.143246
14	-2.230934	-0.138689	-0.058758
1	-0.666535	0.643616	-2.316498
1	0.101584	-0.94673	-2.204288
1	2.259388	0.060582	-1.745473
1	1.598143	1.391931	-2.679274
1	3.404575	1.888294	0.000687
1	2.820448	3.068517	-1.161996
1	2.32854	3.179207	0.558679
1	-3.823456	-1.870592	-0.815663
1	-2.541027	-1.637318	-2.007111
1	-2.217007	-2.563948	-0.535751
1	-3.689491	-0.513375	1.894402
1	-2.456481	0.671323	2.312814
1	-2.035788	-1.045065	2.222018
1	-2.973978	1.312704	-1.937258
1	-4.17952	1.238014	-0.657906
1	-2.78011	2.291802	-0.482814
1	0.126033	0.566756	2.332431
1	1.589602	1.656413	2.13221
1	-1.311647	2.275011	1.197625
1	0.140524	3.355232	0.942317
5	1.482607	-1.688336	0.310528
9	1.793764	-2.173942	-0.977297
9	2.445072	-0.685701	0.615464
9	1.606303	-2.725811	1.2339

model A

ZPE	-678.09671000	Hartree	
6	-0.343487	-1.744284	-0.344514
6	1.181797	-1.772958	-0.18303
6	-0.796689	-0.386069	0.235407
6	0.470699	0.459467	0.073155
6	1.586328	-0.350208	-0.127745
6	2.979005	0.07776	-0.285717
8	-1.15202	-0.472557	1.614148
1	-3.437533	-0.743427	-0.360654
1	-2.741063	1.5587	0.023121
1	-2.125018	0.436503	-2.032383
6	0.620732	1.823428	0.832073
6	0.52058	1.8991	-0.597259
14	-2.387497	0.26282	-0.589798
1	-0.586428	-1.802272	-1.406793
1	-0.848973	-2.563823	0.161437
1	1.508576	-2.215503	0.769515
1	1.72282	-2.318988	-0.957251
1	3.63364	-0.57844	0.290772
1	3.24559	-0.080292	-1.338019
1	3.155936	1.118109	-0.028422
1	-0.263	1.995063	1.432991
1	1.572099	1.980608	1.319061
1	-0.425331	2.147308	-1.058843
1	1.399284	2.090961	-1.195923
1	-0.483275	-0.960953	2.111159

model B

ZPE	-678.09925800	Hartree	
6	-0.530943	-1.36147	1.067627
6	0.699593	-1.845893	0.293722
6	-0.874267	-0.006078	0.422825
6	0.50964	0.473328	-0.034372
6	1.382333	-0.604669	-0.143392
6	2.771687	-0.591134	-0.608351
8	-1.529123	0.899524	1.286916
1	-3.310309	-0.801107	-0.559358
1	-2.301114	1.116684	-1.669932
1	-1.411463	-1.101558	-2.046172
6	1.217184	1.739563	0.650218
6	0.759635	1.831996	-0.714759
14	-2.061853	-0.206409	-1.065913
1	-1.352133	-2.075507	1.050522
1	-0.254224	-1.190752	2.113325
1	1.363625	-2.527531	0.824283
1	0.406233	-2.357169	-0.635973
1	3.4121	-0.885816	0.229602
1	2.895213	-1.371682	-1.364106
1	3.097944	0.366879	-1.003115
1	0.589241	2.120365	1.44345
1	2.268039	1.623153	0.869042
1	-0.175045	2.347743	-0.895537
1	1.476972	1.836279	-1.522743
1	-1.215322	0.779707	2.193045

model B'

ZPE	-795.98876600	Hartree	
6	0.28466	-0.912484	1.630588
6	1.325744	-1.671582	0.800808
6	-0.1599	0.267634	0.743994
6	1.076322	0.475688	-0.128359
6	1.867164	-0.668657	-0.15452
6	3.031739	-0.945637	-1.010517
8	-0.407615	1.399385	1.567564
6	2.20153	1.532334	0.402299
6	1.425099	1.75616	-0.82218
14	-1.691501	-0.154468	-0.354846
1	-0.537983	-1.5478	1.955148
1	0.755337	-0.492259	2.522667
1	2.117195	-2.155753	1.373559
1	0.871092	-2.458113	0.184449
1	3.879948	-1.266961	-0.403567
1	2.763549	-1.800158	-1.641055
1	3.312709	-0.110798	-1.64672
1	1.853012	1.951007	1.336425
1	3.245087	1.264476	0.335572
1	0.629504	2.488012	-0.762931
1	1.93965	1.694206	-1.770614
1	-1.138934	1.928381	1.232657
6	-3.138446	-0.380008	0.799824
1	-4.035937	-0.606691	0.220699
1	-2.972354	-1.202975	1.496375
1	-3.335363	0.522796	1.380409
6	-1.976412	1.283073	-1.516248
1	-1.162656	1.382377	-2.236779
1	-2.896264	1.1055	-2.077904
1	-2.096385	2.237752	-0.998885
6	-1.337561	-1.712439	-1.322981
1	-0.414833	-1.6418	-1.904084
1	-1.281069	-2.588404	-0.674406
1	-2.15302	-1.879818	-2.029932

21

ZPE	-1120.20522700	Hartree	
6	-0.036002	-0.338348	-1.605496
6	-1.370139	-1.096546	-1.699536
6	0.067772	0.108407	-0.126822
6	-0.797274	-0.807725	0.593421
6	-1.658725	-1.567407	-0.283206
6	-3.055646	-2.019455	0.054063
6	-0.450771	-1.604006	1.736996
6	-0.546907	-2.536777	0.440541
1	0.798848	-1.007892	-1.811273
1	0.032368	0.49133	-2.30766
1	-2.185201	-0.440799	-2.007664
1	-1.339686	-1.928151	-2.402825
1	-3.76628	-1.235686	-0.208752
1	-3.303579	-2.909514	-0.524488
1	-3.167145	-2.248584	1.113382
1	0.528891	-1.411438	2.156971
1	-1.218104	-1.892601	2.443955
1	0.386841	-2.672274	-0.08641
1	-1.080603	-3.445237	0.696214
14	-0.832256	1.852114	0.165583
6	0.303213	3.069067	-0.664345
1	-0.092421	4.078534	-0.53206
1	1.298966	3.018858	-0.225325
1	0.384977	2.875611	-1.734809
6	-2.536258	1.897922	-0.602057
1	-3.000712	2.854908	-0.351599
1	-2.494946	1.827	-1.689898
1	-3.186568	1.10828	-0.221521
6	-0.926784	2.093676	2.010795
1	0.046345	1.909484	2.467529
1	-1.227399	3.117528	2.241706
1	-1.657499	1.41945	2.462481
8	1.306032	0.356531	0.443454
5	2.523522	-0.299871	-0.020881
9	3.532539	0.004907	0.888344
9	2.88874	0.143485	-1.300368
9	2.34247	-1.702187	-0.073028

2

ZPE -1120.25221800 Hartree

6	-0.796395	-1.659562	0.870777
6	0.603997	-2.197166	1.169885
6	-0.577278	-0.478613	-0.024055
6	0.8277	-0.285299	-0.391747
6	1.511174	-1.607455	0.085465
6	2.96433	-1.538553	0.503076
6	1.092541	-0.717209	-1.871922
6	1.334492	-2.152371	-1.358738
1	-1.413152	-2.356062	0.293482
1	-1.39004	-1.385742	1.743614
1	0.938616	-1.843793	2.147729
1	0.637698	-3.28655	1.181626
1	3.090382	-0.945398	1.41152
1	3.330974	-2.547619	0.704524
1	3.587921	-1.107807	-0.282383
1	0.279361	-0.551661	-2.576036
1	1.998991	-0.249613	-2.254124
1	0.439435	-2.769092	-1.456966
1	2.184691	-2.690673	-1.775495
14	1.393234	1.482183	0.134959
6	1.008987	1.676177	1.953729
1	1.292508	2.672346	2.298916
1	-0.058743	1.547164	2.148224
1	1.55672	0.945444	2.552863
6	3.220706	1.693576	-0.195682
1	3.44686	2.760565	-0.126278
1	3.844182	1.170251	0.527644
1	3.499762	1.363733	-1.197877
6	0.442949	2.703291	-0.90796
1	-0.622706	2.692806	-0.681731
1	0.826942	3.70904	-0.723003
1	0.569165	2.485216	-1.970586
8	-1.481274	0.259772	-0.466921
5	-2.987886	0.167381	-0.055481
9	-3.424649	-1.094603	-0.38597
9	-3.598642	1.14916	-0.785454
9	-3.038313	0.401099	1.300393
